# How diverse are the mountain karst forests of Mexico?

**DOI:** 10.1371/journal.pone.0292352

**Published:** 2023-10-04

**Authors:** María Eugenia Molina-Paniagua, Pablo Hendrigo Alves de Melo, Santiago Ramírez-Barahona, Alexandre K. Monro, Carlos Manuel Burelo-Ramos, Héctor Gómez-Domínguez, Andrés Ernesto Ortiz-Rodriguez

**Affiliations:** 1 División Académica de Ciencias Biológicas, Universidad Juárez Autónoma de Tabasco, Villahermosa, Tabasco, México; 2 Departamento de Botánica, Instituto de Biología, UNAM, Ciudad Universitaria, Ciudad de México, México; 3 UNESP-Universidade Estadual Paulista “Julio de Mesquita Filho”, Río Claro, São Paulo, SP, Brazil; 4 Americas Team, The Herbarium, Royal Botanic Gardens Kew, United Kingdom; 5 Herbario UJAT, División Académica de Ciencias Biológicas, Universidad Juárez Autónoma de Tabasco, Villahermosa, Tabasco, México; 6 Senda Sustentable, A.C., Tuxtla Gutiérrez, Chiapas, México; Instituto Federal de Educacao Ciencia e Tecnologia Goiano - Campus Urutai, BRAZIL

## Abstract

Tropical forests on karstic relief (tropical karst forest) are among the most species-rich biomes. These forests play pivotal roles as global climate regulators and for human wellbeing. Their long-term conservation could be central to global climate mitigation and biodiversity conservation. In Mexico, karst landscapes occupy 20% of the total land surface and are distributed mainly in the southeast of the country, along the eastern slope, and in the Yucatan Peninsula. Within each of these areas, the following types of karst occur: coastal karst, plain karst, hill karst, and mountain karst (low, medium, high). Mountain karst cover 2.07% of Mexico’s land surface and are covered by tropical rainforests, montane cloud forests, and tropical deciduous forests. These are probably one of the most diverse biomes in Mexico. However, the mountain karst forests of Mexico have received little attention, and very little is known about their diversity. Here, we evaluated the vascular plant species richness within the mountain karst forests of Mexico. We assembled the first, largest, and most comprehensive datasets of Mexican mountain karst forest species, from different public databases (CONABIO, GBIF, IBdata-UNAM), which included a critical review of all data. We compiled a list of the families, genera, and species present within the mountain karst forests of Mexico. Taxa that best characterize these forests were identified based on their spatial correlation with this biome. We explored biodiversity patterns, identifying areas with the highest species richness, endemism centers, and areas of relatively low sampling intensity. We found that within the mountain karst forests of Mexico there are representatives of 11,771 vascular plant species (253 families and 2,254 genera), ca. 50% of the Mexican flora. We identified 372 species endemic to these forests. According to preliminary IUCN red list criteria, 2,477 species are under some category of conservation risk, of which 456 (3.8%) are endangered. Most of the Mexican mountain karst forests have been extensively explored and six allopatric, species-rich areas were identified. Compared to other regions in the world, the mountain karst forests of Mexico are one of the most diverse biomes. They contain more species than some entire montane systems in Mexico such as Sierra Madre Oriental, and Sierra Madre del Sur. Also, the mountain karst forests of Mexico are most diverse than similar forests of South America and Asia, even if considering the effect of different sampling areas. The fact that mountain karst forests are embedded in areas of high biotic diversity, probably contributes to their great floristic diversity. Thus, the mountain karst forests of Mexico are an important source of diversity and shelters a large percentage of the Mexican flora.

## Introduction

Tropical karst forests can be broadly defined as forest communities that growth in areas with karstic soils and are considered one of the most diverse biomes worldwide [1]. This high diversity is attributed to their soil conditions, high heterogeneity of microhabitats, and their archipelago-like distribution [2–8]. It is probable that many plant lineages that have colonized and diversified within these forests are specialized and characterized by their small and generally disjunct distribution ranges [9–12]. Thus, tropical karst forests are an ideal model to study the effects of their discontinuous distribution and extreme environmental conditions on their levels of endemism and biodiversity [2, 7, 11]. However, there are relatively few studies on the plant diversity of this biome [7].

Data published so far suggest that tropical karst forests in small islands harbor many endemic plant species, most associated with positive karst terrains (mountaintop hills and walls) [7, 8]. Within continental regions, studies have shown that tropical karst forests in Asia and Africa represent an important source of regional biodiversity and endemism, similar to that observed in islands [7]. For America, published data suggest that South American karst forests (Brazil) are a reservoir of the regional flora (high levels of representativeness of the surrounding flora) but have lower levels of endemism compared to the tropical forests of Asia and Africa [5]. However, for most regions in the Americas tha have large extensions of tropical karst forest, such as Mexico, Central America, and the Caribbean, it is unclear what proportions of their flora are represented by karst species [7, 10, 13].

**Fig 1:**
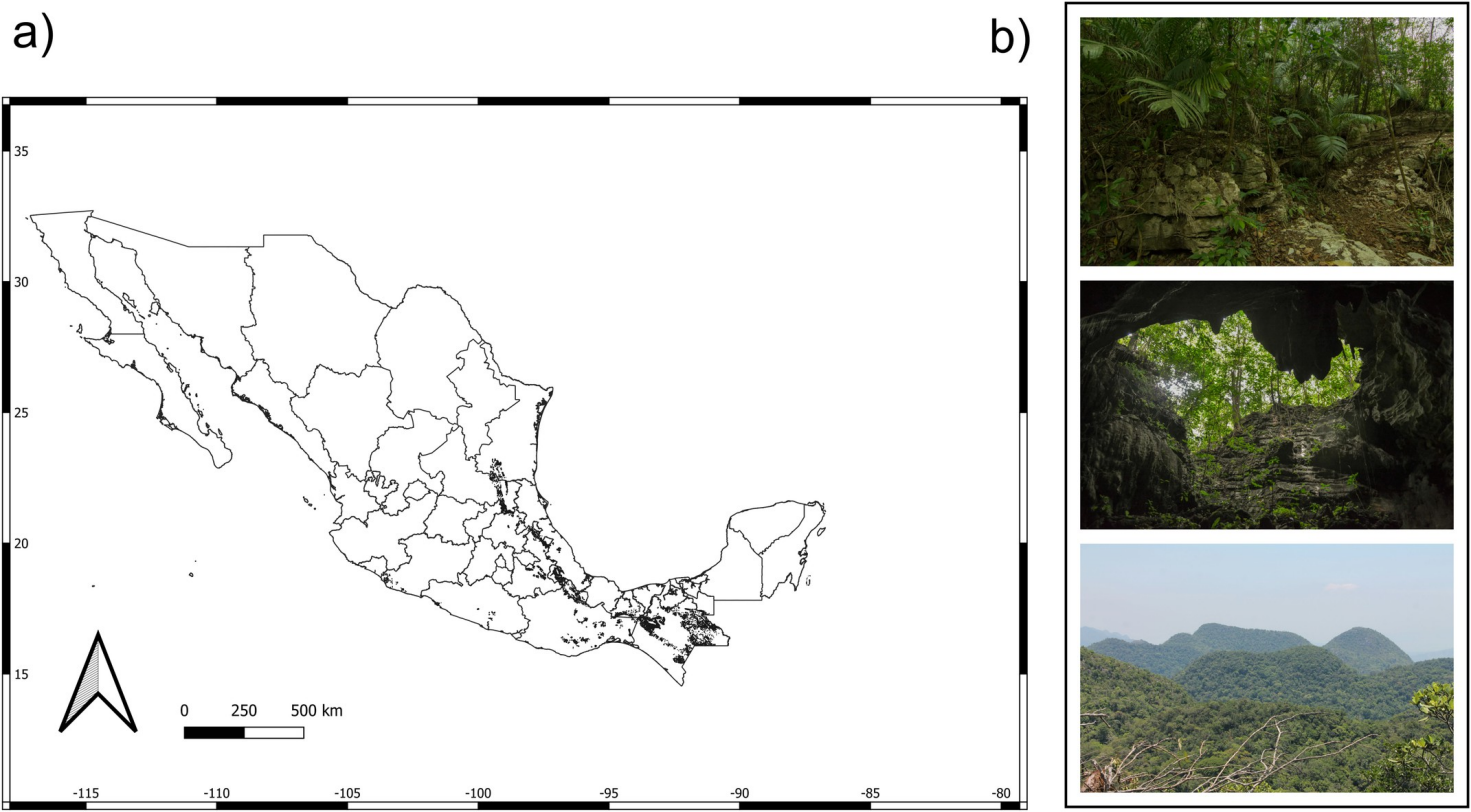
Mountain karst forests of Mexico. a) it is limited to small, archipelago like-distributed remnants along the mountain systems of western (Guerrero and Michoacán), eastern (Puebla, Querétaro, San Luis Potosí, Tamaulipas, and Veracruz), and southern Mexico (Chiapas, Oaxaca, and Tabasco), and occupying an area of 40,759.87 km^2^ (2.07% of Mexico surface). b) Most of the Mountain karst forest area is occupied by the tropical rain forest. In these forests, the chasms, cliffs, caves and limestone soils characterize the landscape. Forest pictures by Marcos Escobar. Background map: political division of Mexico provided by Comisión Nacional de Áreas Naturales Protegidas (CONANP) under CCBY 4.0 International License. The the karst forest polygons was obtained from the Atlas Nacional de Riesgos (http://www.atlasnacionalderiesgos.gob.mx/archivo/visor-capas.html), under a CC BY 4.0 license.

In Mexico, karst landscapes occupy 20% of the total land surface (~391 700 km^2^) and are distributed mainly in the southeast of the country (Chiapas, Guerrero and Oaxaca), along the eastern slope, and in the Yucatan Peninsula [14–17]. Within each of these areas the following types of karst occur: coastal karst, plain karst, hill karst, and mountain karst (low, medium, and high) [16]. Each type is determined by its structure, elevation, and the climatic conditions driving the karstification process [14–17]. Mountain karst (named hereafter as mountain karst forests) are distributed discontinuously through the Sierra Madre Oriental, from the southwest of Tamaulipas to southern Veracruz, in the north-eastern portion of Oaxaca, in southern Tabasco and several regions of the state of Chiapas (Fig 1). In contrast, coastal karst, plain karst, and hill karst are best represented and almost restricted to the Yucatan Peninsula (Karst of the Yucatan Peninsula or platform plains and hills karst [15–17]).

Mountain karst forests cover 2.07% of Mexico’s land surface, and include parts of some species-rich forest types in the country, such as tropical rain forest, montane cloud forest, and deciduous forest (Fig 1) [15–17]. Despite this, studies focused on the mountain karst forests of Mexico are scarce [2, 10, 18–21] and estimates of their total diversity have been based on low sampling effort and therefore likely to be underestimated. Moreover, mountain karst forests encompass biodiversity hotspots where new species are being described steadily [22], yet species extinction is increasing rapidly due mainly to loss of suitable habitats [23]. The availability of large and public databases that contain historical and contemporary taxonomic information collected over several decades in many parts of the world and often accompanied by ecological and geographic metadata [24, 25], offers an opportunity to more accurately estimate plant diversity within these forest communities.

Here, we assembled the first comprehensive dataset for Mexican mountain karst forests plant species from different public databases following a critical review of data. We sought to determine the number of species restricted to the mountain karst forest of Mexico and the conservation status of each of them. We also explored biodiversity patterns and identified species richness areas, endemism centers, and little-explored regions. Specifically, we aimed to answer the following questions: 1) How diverse is the mountain karst forest of Mexico? 2) What are the spatial patterns of species richness distribution? 3) Where are the centers of endemism? 4) How many species are restricted to mountain karst forest? and, 5) What is the conservation status of each of them?

## Methods

### Data acquisition

**Fig 2:**
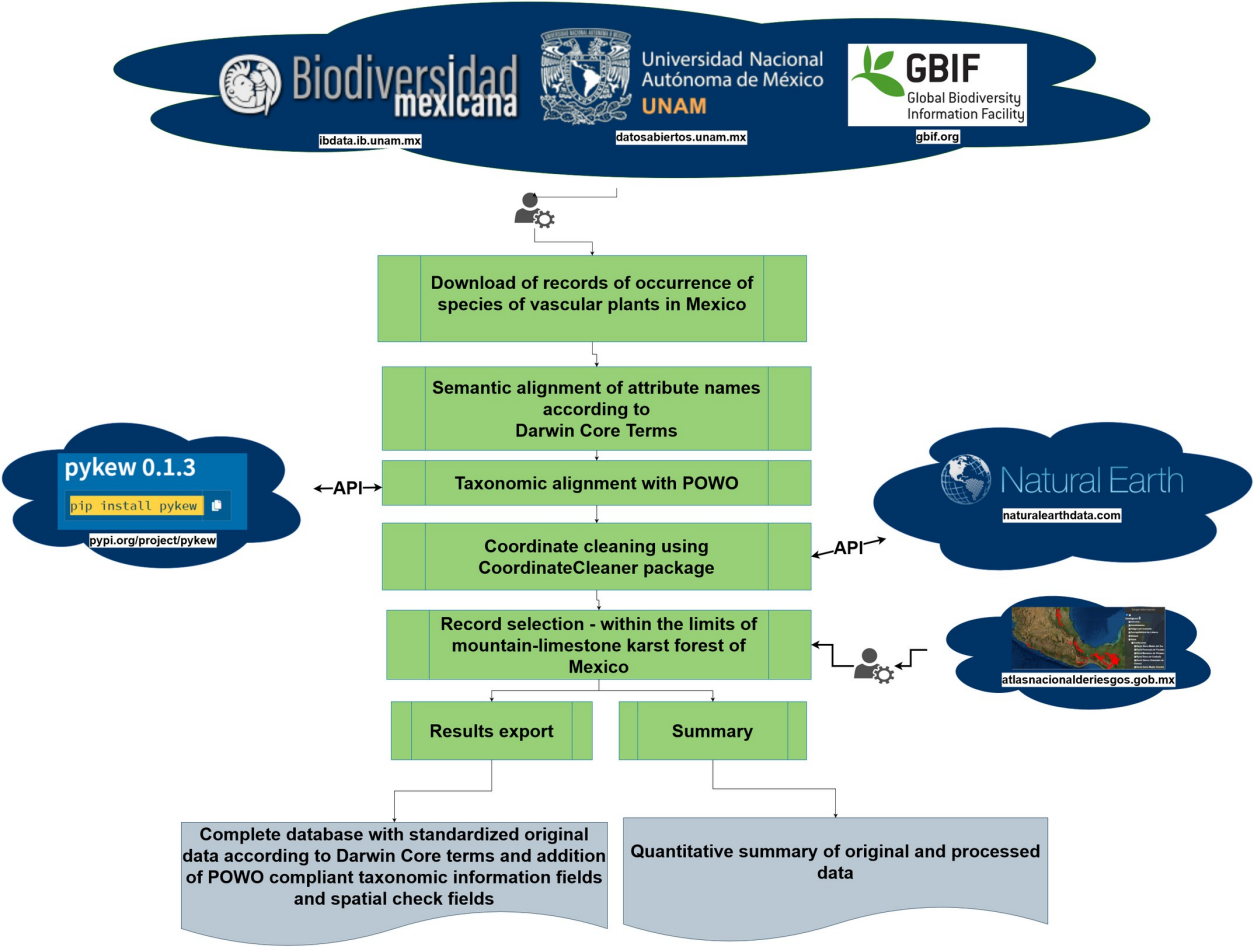
Workflow for composing vascular plants dataset for Mexico and extracting flora from mountain karst forests of Mexico. POWO = Plants of the World Online (https://powo.science.kew.org/).

Fig 2 summarizes the workflow used to obtain and validate the names that underpin the vascular plant dataset for Mexico, and which was used to extract occurrence data for mountain karst forests of Mexico.

We obtained 7,655,611 records of the Mexican vascular plant flora sourced from: 1) the Comisión Nacional para el Conocimiento y Uso de la Biodiversidad [26] (47.7% of total records), 2) the Global Biodiversity Information Facility [27] (36.8% of total records; (https://doi.org/10.15468/dl.af62vr)), and 3) the online database of the scientific collections of the Institute of Biology-UNAM [28] (IBdata v3 «Helia Bravo Hollis» (https://www.ibdata.abaco3.org); 15.5% of total records). To facilitate content comparison and manipulation between data sources, a semantic alignment of attribute names was undertaken according to Darwin Core Terms. This was followed by a harmonization of taxonomic names using Kew’s Plants of the World database [29] using the *pykew* 0.1.3 *R*-package [30]. We discarded records that were not identified to the rank of species. Synonyms, duplicate specimens, and invalid, unmatched or missing names were also discarded. This resulted in 6,492,388 records, representing a reduction of 16% of records.

We also discarded records lacking accurate geolocations according to the following criteria: a) non-numeric and not available coordinates (9.2% of total records); b) zero longitude or latitude (10 records flagged); c) coordinates outside the reference landmass (23.5% of records); d) coordinates in the vicinity of Country and province centroids (0.5% of total records); e) coordinates in the vicinity of country capitals (0.3% of total records); f) records inside urban areas (3.8% of total records); g) records in the vicinity of biodiversity institutions (0.1% of total records); and h) duplicated records (16.3% of total records). We did so using *CoordinateCleaner R*-package [31]. This resulted in a further reduction of 14% resulting in a dataset of 5,820,489 occurrence records representing 21,659 species of Mexican vascular plants [32]. We extracted the mountain karst forests species from the above dataset using the shape file depicted in Fig 1, where duplicate collections were eliminated based on the combination of cell (0.08 degrees), year, month, day, and scientific name of collections. These resulted in a first mountain karst vascular plant dataset comprising 176,884 occurrence records.

### Floristic characterization of mountain karst forests of Mexico and species affinity to karst

The families, genera and species present within the mountain karst forests of Mexico were compiled. Taxa that best characterize the mountain karst forests of Mexico were identified based on their spatial correlation to this biome. Specifically, we counted the number of ’karstic’ cells (0.5-degree latitude/longitude) that each species occupies (area of occupancy) throughout its entire geographic range, by homogenizing the sampling effort to one record per cell, and in cases of combined cells (karstic, non-karstic), one record considered for each category. Thus, three types of spatial correlation were identified: 1) karst endemic species, whose total number of grid cells are exclusive to mountain karst forests; 2) karst associated species, defined as those with more than half of occupied grid cells within mountain karsts forest; and 3) non-karst species, defined as species with more than half of occupied grid cells occurring outside mountain karst forests.

Species-richness and levels of endemism in Mexico were compared to other karst forests for which comparable information was available. This included karst forest from Brazil [5], China [33], and the Malayan Peninsula [34]. To compare species richness between areas of different sizes, a taxonomic biodiversity index was used [35]. This index calculates the number of observed species divided by the natural logarithm of the area in km2 (IB = E/lnA), where “E” is the number of recorded species and “A” the area [35]. Species-richness and endemism of the mountain karst forest of Mexico was also compared to non-karst areas surroundings the study area, such as the Sierra Madre Oriental [36, 37] and the Sierra Madre del Sur (SMS) [38, 39].

### Biodiversity distribution patterns and the conservation status of species

The mountain karst forests of Mexico was divided into 89 grid cells (0.5-degree latitude/longitude grid size) in order to map species richness, endemism, and conservation status. Each grid cell covered an area of approximately 256 km^2^, a medium-sized scale that avoids the effect of sampling artifacts, such as the occurrence of artificially empty grid squares [40, 41]. To assess whether the mountain karst forests of Mexico have a high degree of endemism, we estimated the weighted endemism (WE) [40] and corrected endemism (CWE) [41] indices using the Biodiverse v.1.1 software [42]. We defined endemic species as those restricted to the mountain karst forest (all georeferenced records distributed exclusively within this biome). For each species within the mountain karst forest of Mexico, we carried out a preliminary assessment of its conservation status by calculating the extent of occurrence (EOO) and the area of occupancy (AOO) using occurrence data, and applying the function *iucn*.*eval* implemented in the *conR R* package [43] and the IUCN Red List Categories and criteria. We also consulted which plant species are protected under Mexican laws (Norma Oficial Mexicana NOM-059-SEMARNAT [44]). Lastly, for each grid cell we measured and mapped separately the total number of species and those that are endemic and threatened. The resulting patterns (maps) were analysed by overlaying layers for each parameter, enabling us to detect areas with the highest species-richness, but also those that host the highest number of endemic and threatened species based on preliminary assessments. The maps were generated and projected in QGIS version 3.22.1-Białowieża [45] and Infomap Bioregions [46].

## Results

### Floristic characterization and species mountain karst forest affinity

A total of 11,771 species of vascular plants were recorded within the mountain karst forests of Mexico, representing 253 families and 2,254 genera (S1 Table). The 14 most diverse families in terms of number of species (more than 200 species) are presented in Table 1; these families contribute more than 50% of all recorded species. The Piperaceae, Polypodiaceae, and Orchidaceae stand out because 91%, 78%, and 62% of their known species in Mexico, respectively, were recorded within the mountain karst forests. Table 2 shows the plant families that characterize these forests. Among these, Gesneriaceae, Melastomataceae, Dioscoreaceae, Araceae, Violaceae, Urticaceae, Arecaceae, and Piperaceae have the highest proportion of their occurrence records in Mexico distributed exclusively within the mountain karst forests. The ten genera with the highest number of species within the mountain karst forests are *Piper* (119 species), *Salvia* (111), *Peperomia* (103), *Ipomoea* (101), *Solanum* (100), *Tillandsia* (100), *Euphorbia* (95), *Epidendrum* (81), *Senna* (86), and *Mimosa* (78).

**Table 1:** The most diverse vascular plant families [more than 200 species within the mountain karst forest of Mexico (MKF)]. The percentage of species with respect to the total number of species present in Mexico (^26,29^) is shown in parentheses.

Family	Species in Mexico	Species within the MKF
**Piperaceae**	245	222 (91%)
**Polypodiaceae**	293	230 (78%)
**Orchidaceae**	1213	760 (62%)
**Solanaceae**	407	240 (59%)
**Poaceae**	1047	606 (57%)
**Acanthaceae**	385	220 (57%)
**Fabaceae**	1903	1087 (57%)
**Rubiaceae**	707	396 (55%)
**Malvaceae**	527	284 (53%)
**Apocynaceae**	418	213 (50%)
**Cyperaceae**	416	210 (50%)
**Euphorbiaceae**	714	340 (47%)
**Lamiaceae**	601	229 (38%)
**Asteraceae**	3057	1081 (35%)

**Table 2:** Most common plant families with the highest proportion of records distributed exclusively within the mountain karst forest (MKF) of Mexico.

Families	Total records	Exclusive MKF records (%)
**Gesneriaceae**	1283	14.06
**Melastomataceae**	2432	12.64
**Dioscoreaceae**	1069	12.53
**Araceae**	4944	12.24
**Violaceae**	735	12.24
**Urticaceae**	1440	11.97
**Arecaceae**	2648	11.68
**Piperaceae**	3200	11.22
**Begoniaceae**	1437	11.03
**Orchidaceae**	13364	10.80
**Primulaceae**	2264	10.68
**Marantaceae**	689	10.17
**Lauraceae**	4115	10.10
**Myrtaceae**	2521	9.73
**Araliaceae**	1816	9.72
**Meliaceae**	2576	9.05
**Bromeliaceae**	7751	8.92
**Smilacaceae**	1056	8.44
**Annonaceae**	1238	8.07
**Sapotaceae**	1469	7.96
**Phyllanthaceae**	902	7.69
**Rubiaceae**	9749	7.54
**Celastraceae**	1561	7.36
**Moraceae**	4392	7.20
**Loranthaceae**	728	7.03
**Cannaceae**	921	6.95
**Malpighiaceae**	3919	6.88
**Passifloraceae**	3352	6.57
**Phytolaccaceae**	1444	6.57
**Acanthaceae**	7499	6.38
**Gentianaceae**	1120	6.28
**Caricaceae**	1149	6.09
**Iridaceae**	2433	6.07
**Salicaceae**	2928	6.04
**Polygalaceae**	1135	6.03
**Campanulaceae**	2290	5.96
**Burseraceae**	8152	5.93
**Vitaceae**	1453	5.86
**Crassulaceae**	3407	5.86
**Petiveriaceae**	1297	5.80
**Lythraceae**	3038	5.70
**Malvaceae**	19919	5.66
**Bixaceae**	694	5.48
**Sapindaceae**	4147	5.36
**Apocynaceae**	17736	5.35
**Oleaceae**	818	5.32
**Combretaceae**	2465	5.29
**Bignoniaceae**	6716	5.21
**Rosaceae**	2876	5.10

**Fig 3:**
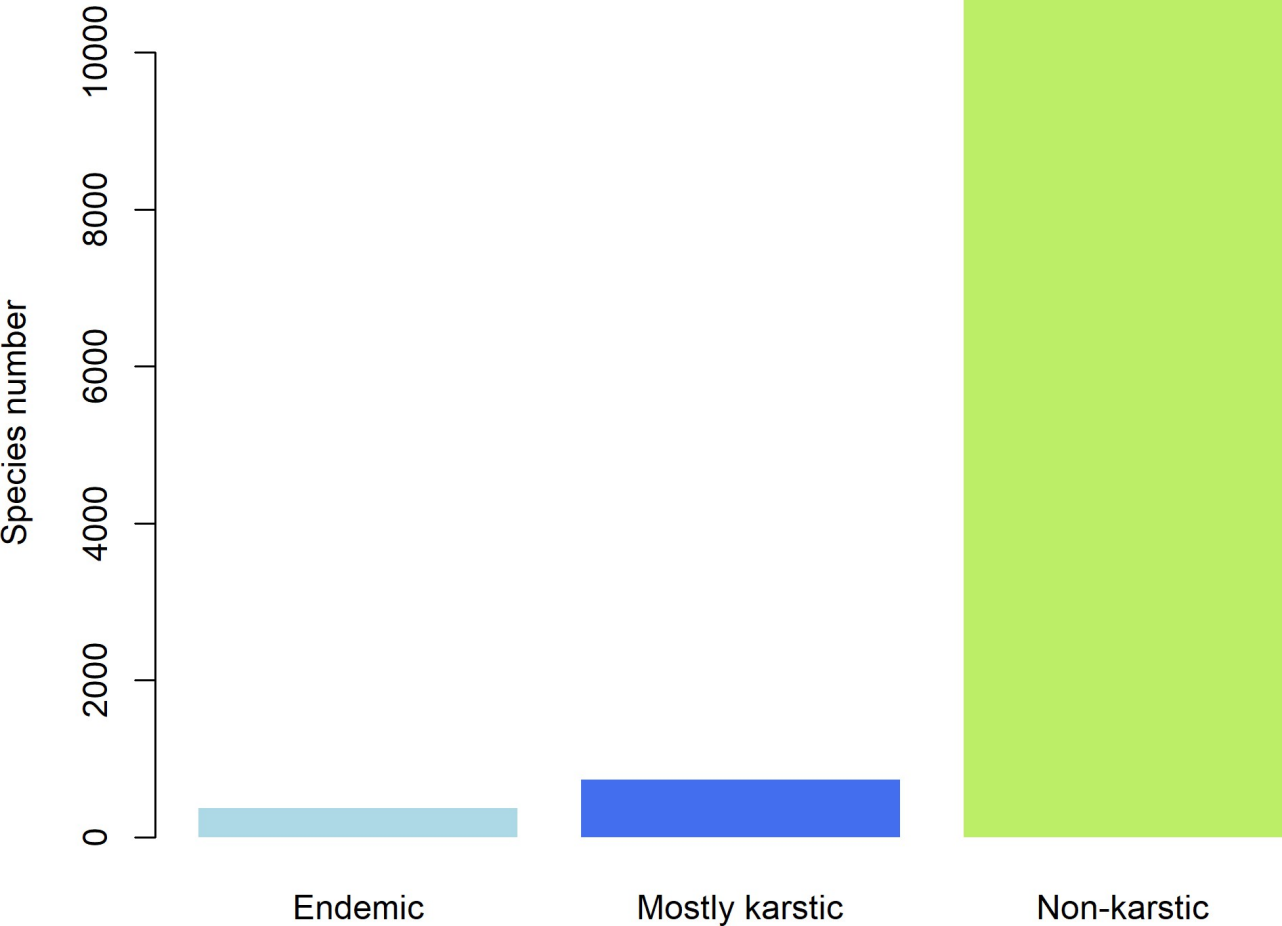
Number of recorded species distributed according to their spatial correlation to the mountain karst forests of Mexico.

We identified 372 endemic species (3.16% of the total species recorded in the mountain karst forest of Mexico), belonging to 84 families and 235 genera (S1 Table). The families with the highest number of endemic species are Orchidaceae (56 species), Fabaceae (26 species), Asteraceae (25 species), Piperaceae (19 species), Poaceae (13 species), Araceae (12 species), Aspleniaceae (12 species), Acanthaceae (10 species), and Polypodiaceae (10 especies). An additional 731 species had between 50% and 90% of their records in Mexico distributed exclusively within the mountain karst forests (the mostly karstic species), whilst the majority of the species recorded (10,668 species) had more than 50% of their records in Mexico outside the mountain karst forests (the non-karstic species) (Fig 3). The proportion of records found outside the mountain karst forest increased as a function of the total number of records (r^2^ = 0.99, pval = < 2.2e-16). While 84% of the non-karstic species are well represented in collections and known from more than 20 localities, species that are endemic to the mountain karst forests are known from a few scientific collections (mean = 2, max = 17, min = 1).

The taxonomic diversity index shows that the mountain karst forests of Mexico are highly diverse relative to similar biomes in South America and Asia (Table 3).

**Table 3:** Taxonomic diversity index calculated for the mountain karst forests of Mexico and similar biomes around the world. Mountain karst forests of Brazil, mountain karst forests of Guizhou, China, Malayan mountain karst forests, Southeast Asia.

Karst areas	Area (km^2^)	Species	Endemic species	Species/area	Taxonomic Biodiversity index
**Brazil**	318,126	9,592	468	0.0302	757
**Guizhou, China**	170,000	7,505	171	0.0441	623
**Malaysia, southeast Asia**	260	1,216	130	4.6769	219
**Mexico**	**40,759**	**11,771**	**372**	**0.2586**	**993**

### Biodiversity distribution pattern and conservation status of species

**Fig 4:**
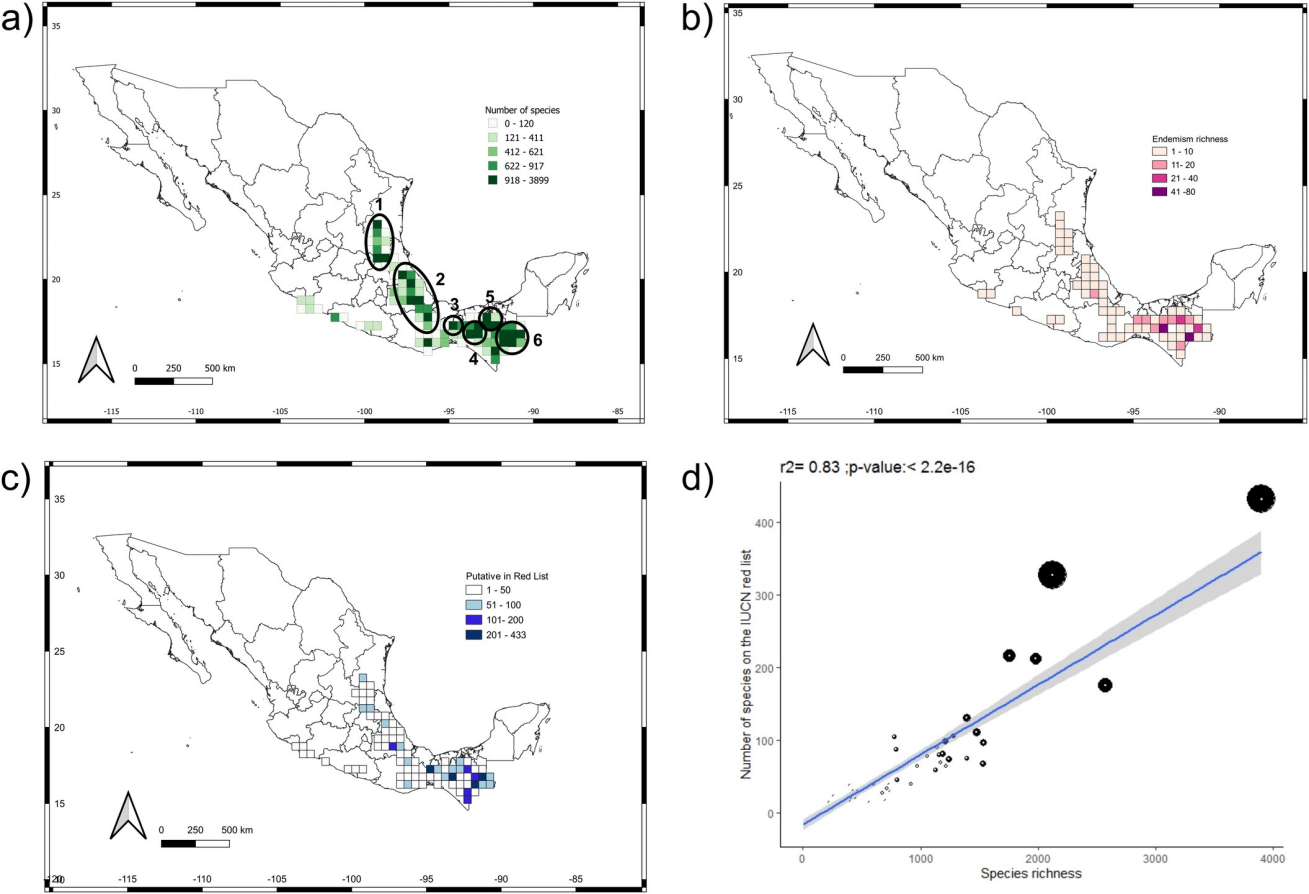
Biodiversity distribution pattern and conservation status of species. a) Species richness distribution, the regions with the highest number of species are marked in circles: 1) the Huasteca region, 2) the Zongolica region, 3) the Chimalapas-Uxpanapa region, 4) Central Chiapas, 5) Highlands of Chiapas, and 6) the Lacandon region. b) Distribution of endemism. c) Distribution of threatened species. d) Pearson’s correlation analysis between the species richness and the number of threatened species per cell, the size of the circles represents the number of endemic species. Background map: political division of Mexico provided by Comisión Nacional de Áreas Naturales Protegidas (CONANP) under CCBY 4.0 International License.

Species richness (the total number of recorded species in a cell) increased with sampling effort (Pearson’s correlation: r^2^ = 0.84, pval = < 2.2e-16). Although 38% of the grid cells within the mountain karst forests of Mexico have been poorly sampled [less than the median number of records (622) expected per cell), most forest grid cells (more than 60% of the grid cells analysed) had between 622 and 24,975 records. Moreover, sampling effort is not concentrated in a particular region but rather randomly distributed across the entire study region (S1 Fig). Accordingly, six regions were considered as species-rich: 1) the Huasteca region between Queretaro, Hidalgo and San Luis Potosí (1531 species), 2) the Zongolica region in Veracruz (2,574 species), 3) the Chimalapas-Uxpanapa region in Oaxaca and Veracruz (3,290 species), 4) Central Chiapas (3,899 species), 5) Highlands of Chiapas (2,124 species), and 6) the Lacandon region (1,985 species) (Fig 4).

We also identified ten areas of endemism with nine or more endemic species (Fig 4), distributed mainly in Southern Mexico (Chiapas, Oaxaca, and Veracruz) but also in East-Central Mexico (Veracruz and Puebla, the Zongolica region).

According to the IUCN red list criteria, there are 2,477 species in some risk category, most of them (2,021) under the Least Concern category (Table 4). Under Mexican laws, 376 species are protected, most of them (147) under the Endangered category (Table 4). The tentative conservation status assessment (using the *ConR* package) suggests that 2,704 species (22.9% of all registered species) could be under some risk category and most species (1,166) fall under the Vulnerable category (Table 4 and Fig 4).

**Table 4:** Number of species under some category of risk according to Mexican laws (NOM-059) and the IUCN red list. The results of the preliminary assessment of the conservation status using the *conR*, *R*-package are also shown.

Status	NOM-059	Red List	Red List preliminary assessment
**LC o NT**		2,021	
**VU**	136	186	1166
**EN**	147	221	1164
**CR**	91	45	374
**EW**	1	4	

Lastly, the overlay of richness, endemism, and threat category revealed that the most species-rich regions contain a high number of endangered species, many of them endemic, making these regions priority areas for conservation (Fig 4). The above is critical considering that only a small portion, 2% of the mountain karst forests, is protected within Mexican natural reserves (S2 Fig).

## Discussion

In this study, we estimated the number of species inhabiting the mountain karst forests of Mexico and the conservation status of each of them. We explored biodiversity patterns, identifying species richness areas, endemism centers, and little-explored regions.

### How diverse is the mountain karst forests of Mexico?

In Mexico, a wealth of 23,314 species of native vascular plants, 297 families and 2,854 genera have been estimated [32]. The species richness present in the mountain karst forests of Mexico (11,771 species, 2,254 genera, and 253 families) constitutes 50.49% of the plant species, 78.98% of genera, and 85.19% of the plant families known for Mexico. The fact that mountain karst forests includes some of the most species-rich forest types in Mexico, in addition to being embedded in areas of high biotic diversity, probably contributes to its great floristic diversity. Contrary to expectations, the most diverse plant families in Mexico (Asteraceae, Rubiaceae, Lamiaceae [32]) are not the most species-rich families within the mountain karst forests (Table 1), nor characterize these forests (Table 2). Instead, Gesneriaceae, Melastomataceae, Dioscoreaceae, Araceae, Violaceae, Urticaceae, Arecaceae, and Piperaceae, characterize the mountain karst forests of Mexico by having the highest number of species distributed exclusively within this biome (Table 2). Among these, the Piperaceae stands out with more than 90% of its species recorded for Mexico present in the mountain karst forest (Table 1). Interestingly, this study revealed that there are plant lineages such as Elatinaceae, Nelumbonaceae, Frankeniaceae, Juncaginaceae, Nitrariaceae, Setchellanthaceae, Simmondsiaceae that are not favored by karst soils and rather their diversity is concentrated in other biomes (coastal, aquatic, floodable, marshy or xeric habits).

We identified 372 species endemic to the mountain karst forests of Mexico where Orchidaceae, Fabaceae, Asteraceae, Piperaceae, Poaceae, Araceae, Aspleniaceae, and Acanthaceae are the plant families with the most endemic species. The first three families are species-rich lineages with a wide distribution in Mexico [32], and although they are not particularly diverse within the mountain karst forest, most of the recorded species are endemic to this biome. Other studies show similar results, where most of the recorded species of Asteraceae and Orchidaceae tend to be restricted to karst forests [36, 47]. For Araceae and Piperaceae, our results showed that a large proportion of their species in Mexico are associated with the mountain karst forest. Furthermore, from the total number of species endemic to Mexico (11,610 species) [32], 1,424 (11%) have representatives within the mountain karst forests, of which 3.2% (372 species) are fully restricted to this biome. Accordingly, the mountain karst forests of Mexico are an important source of diversity and shelters a large percentage of the Mexican flora.

Compared to other regions of Mexico and the world, the mountain karst forests (40, 759 km^2^_,_ 11,771 species) is one of the most diverse biomes. They contain more species than entire montane regions in Mexico, such as Sierra Madre Oriental (220,151 km^2^, 6,981 species [37]), and Sierra Madre del Sur (143,447 km^2^, 7,016 species [39]). The mountain karst forests of Mexico are more diverse than similar forests of Brazil, Guizhou (China), and Malaysia, even after considering the effect of different sampling areas (Table 3). Interestingly, our study revealed that the Neotropical karst forests (Brazil and Mexico) are more taxonomically diverse than the forests of Asia (Guizhou and Malaysia). However, Asian forests have a higher proportion of plant endemism than Neotropical forests (Table 3). Thus, it is probable that in the Neotropics these forest assemblages are phylogenetically more heterogeneous compared to Asia, but the speciation processes (species radiation) directly associated with karst habitats are more frequent in Asia than in America [11, 48–51]. This must be taken with caution as it is probably a generalized response that may not apply to particular families and genera in Mexico or the Neotropics as whole.

On the other hand, families with more endemic species within the mountain karst forests of Mexico are those with mainly herbaceous habits, such as Piperaceae, Araceae, Orchidaceae, and Asteraceae. This is probably associated with the restrictions that rocky soils represent for the establishment of trees and shrubs. It is probable, then, that shallow soils with little organic matter and water favor the growth of herbaceous plants and limit the development of arboreal species. Studies in herbs show that changes in genome size are associated with their presence in these habitats [49, 50].

### Biodiversity distribution pattern

Some authors based on the floristic pattern of particular regions considered the mountain karst forests of Mexico as centers of diversity and endemism, as a result of these being floristic refuges during periods of past climatic fluctuations [2, 52]. Our results support this hypothesis: the mountain karst forests of Mexico are characterized by a high number of species, many of them restricted to this biome, and where areas of high richness and endemism are allopatrically distributed throughout the study region (Fig 4), a pattern associated with the presence of floristic refuges. Six regions are here identified as areas of high species diversity and endemism, of which the Chimalapas-Uxpanapa, the Zongolica regions, and central Chiapas have been proposed by other authors as floristic refuges (S3 Fig) [2, 52].

In general, high species richness areas are confined to topographically, ecologically, and evolutionarily complex regions of Mexico, many being part of the biotic transition zone between the Holarctic and Neotropical regions of America. The development of mountain karst forests within mountain systems, corridors, and valleys in Mexico has driven, together with a complex climatic history, the evolutionary history of their biota.

### Conservation status

Our study shows that the mountain karst forest of Mexico harbor many species with a restricted distribution range and that are therefore at an elevated risk of extinction. The most diverse areas and with high levels of endemism are also those with the most endangered species (Fig 4). These regions are undoubtedly a priority for the conservation of the mountain karst forests in particular and for the Mexican flora in general. However, only 2% of the mountain karst forests of Mexico are within protected natural areas (S2 Fig). According to the criteria established by the IUCN, more than 2,000 species (more than 20% of the species recorded) could be under some risk category (Table 3), of which 1080 occur within protected areas. If least concern species are not considered, more than twice of the species currently assessed by IUCN may need protection. This without considering that a much smaller number of species is currently protected by Mexican law (Table 3).

### Experimental error sources

Species richness and sampling effort are correlated (S4 Fig). However, more than 60% of the forests under study show high levels of sampling effort and the areas of high richness and endemism are randomly distributed. In addition, most of the grid cells analysed fall below the regression line including two of the most species-rich sites, suggesting that these areas have been relatively well sampled. The fact that we observed a high degree of correlation between species richness and sampling effort suggests that species-richness in species-poor sites has been previously underestimated, possibly due to its geographical proximity to areas traditionally well explored or recognized as species-rich sites [5].

Concerning the usefulness of public databases, our occurrence dataset gathers geographic information for 21,659 species of vascular plants, a number very similar to the number of species estimated for the flora of Mexico (23,314 [32]; 24,360 [53]). We obtained a total of 7,655,611 records from three public data sources, in order of contribution CONABIO, GBIF and IB data. After filtering, as detailed in the methods section, 76% of the records originally obtained were used in the final analyses. Poorly georeferenced data, duplicate geo-references, and duplicate collections, were the most frequently deleted records. Thus, the distribution pattern of species richness in Mexico based on our database is consistent with the pattern described in previous studies (S5 Fig) [32, 53].

With respect to the taxonomic uncertainty, we standardize scientific names based on POWO [29]. From the scientific names originally obtained (56,302 species), 51% were retained as valid according to POWO. As detailed in the methods section, this cleaning also included the filtering of determinations above the species level, synonyms, duplicate collections, and missing names. Even so, our dataset probably includes “misdeterminations”, which directly influence the number of species estimated for Mexico and for the Mexican mountain karst forest. “Misdeterminations” have a complex origin, some probable factors are the little experience of botanists on the taxonomy of the species, the absence of local specialists, the absence of taxonomic keys or local and regional monographs, and of course, the species concept used (taxonomic issues). Taxonomic issues are ultimately due to the evolutionary nature of species, something hard to solve completely by standardization [54]. Interestingly, our species richness estimate and their distribution patterns for Mexico are quite similar to those inferred by other independent studies.

## Supporting information

S1 Table Plant species recorded within the mountain karst forest of Mexico, including their risk category according to the IUCN red list and their spatial correlation to the forest (endemism). (PDF)

S1 Fig Sampling effort distribution within the mountain karst forest of Mexico. Background map: political division of Mexico provided by Comisión Nacional de Áreas Naturales Protegidas (CONANP) under CCBY 4.0 International License.
(PNG)

S2 Fig Mountain karst forest distribution and location of the main protected natural areas in Mexico. Background maps: political division of Mexico and protected natural areas provided by Comisión Nacional de Áreas Naturales Protegidas (CONANP) under CCBY 4.0 International License.
(PNG)

S3 Fig Species richness distribution within the mountain karst forest. The colored polygons indicate the location of the floristic refuges in Mexico according to [2, 52]. Background map: political division of Mexico provided by Comisión Nacional de Áreas Naturales Protegidas (CONANP) under CCBY 4.0 International License.
(PNG)

S4 Fig Pearson’s correlation plot (r^2^ = 0.84, pval = < 2.2e-16) between species richness and sampling effort per cell (50 x 50). (JPEG)

S5 Fig Species richness distribution of vascular plant in Mexico. Background map: political division of Mexico provided by Comisión Nacional de Áreas Naturales Protegidas (CONANP) under CCBY 4.0 International License.
(PNG)
