# Supplementary material for: How diverse are the mountain karst forests of Mexico?
Source: PLoS One. 2023 Oct 4;18(10):e0292352. doi: 10.1371/journal.pone.0292352 (PMC10550121; doi:10.1371/journal.pone.0292352)
Supplement: S1 Table — (PDF) [file pone.0292352.s001.pdf]

Family	Species	NOM_059	Red List	Reld list-Preliminary status (ConR)	Endemic to karst
Acanthaceae	Anisacanthus quadrifidus				
Acanthaceae	Anisacanthus quadrifidus var. wrightii				
Acanthaceae	Aphelandra aurantiaca				
Acanthaceae	Aphelandra gigantiflora				
Acanthaceae	Aphelandra leariloba				
Acanthaceae	Aphelandra madrensis				
Acanthaceae	Aphelandra scabra				
Acanthaceae	Aphelandra schiedeana				
Acanthaceae	Aphelandra speciosa				
Acanthaceae	Aphelandra straminea			CR	YES
Acanthaceae	Aphelandra wendtii				
Acanthaceae	Avicennia germinans	VU			
Acanthaceae	Barleria oenotheroides				
Acanthaceae	Bravaisia berlandieriana				
Acanthaceae	Bravaisia grandiflora			EN	
Acanthaceae	Bravaisia integerrima	VU			
Acanthaceae	Carlowrightia arizonica				
Acanthaceae	Carlowrightia neesiana				
Acanthaceae	Chileranthemum pyramidatum				
Acanthaceae	Chileranthemum trifidum				
Acanthaceae	Crossandra infundibuliformis				
Acanthaceae	Dianthera breviflora				
Acanthaceae	Dianthera candelariae				
Acanthaceae	Dianthera candicans				
Acanthaceae	Dianthera glabra			EN	
Acanthaceae	Dianthera inaequalis				
Acanthaceae	Dianthera lindeniana				
Acanthaceae	Dianthera pectoralis				
Acanthaceae	Dianthera reptans			CR	YES
Acanthaceae	Dianthera sessilis				
Acanthaceae	Dicliptera acuminata				
Acanthaceae	Dicliptera acuminata var. spicata			VU	

Acanthaceae	Dicliptera anomala	VU
Acanthaceae	Dicliptera clinopodia	EN
Acanthaceae	Dicliptera imbricata	CR
Acanthaceae	Dicliptera membranacea	
Acanthaceae	Dicliptera peduncularis	
Acanthaceae	Dicliptera resupinata	
Acanthaceae	Dicliptera sciadephora	
Acanthaceae	Dicliptera sexangularis	
Acanthaceae	Dicliptera sumichrastii	
Acanthaceae	Dicliptera unguiculata	
Acanthaceae	Dyschoriste angustifolia	
Acanthaceae	Dyschoriste capitata	
Acanthaceae	Dyschoriste crenulata	
Acanthaceae	Dyschoriste hirsutissima	
Acanthaceae	Dyschoriste microphylla	
Acanthaceae	Dyschoriste ovata	
Acanthaceae	Dyschoriste quadrangularis	
Acanthaceae	Dyschoriste saltuensis	
Acanthaceae	Dyschoriste xylopoda	
Acanthaceae	Elytraria bromoides	
Acanthaceae	Elytraria imbricata	
Acanthaceae	Elytraria macrophylla	
Acanthaceae	Elytraria mexicana	
Acanthaceae	Henrya insularis	
Acanthaceae	Henrya scorpioides	
Acanthaceae	Holographis ehrenbergiana	
Acanthaceae	Holographis ilicifolia	
Acanthaceae	Holographis leticiana	VU
Acanthaceae	Holographis parayana	
Acanthaceae	Holographis tamaulipica	EN
Acanthaceae	Hygrophila costata	VU
Acanthaceae	Hygrophila polysperma	VU
Acanthaceae	Hypoestes phyllostachya	
Acanthaceae	Justicia aurea	

Acanthaceae	Justicia bartlettii		
Acanthaceae	Justicia borrerae		
Acanthaceae	Justicia bracteosa	CR	YES
Acanthaceae	Justicia brandegeeana		
Acanthaceae	Justicia breedlovei	EN	
Acanthaceae	Justicia brevifolia	CR	YES
Acanthaceae	Justicia campechiana		
Acanthaceae	Justicia canbyi		
Acanthaceae	Justicia carnea		
Acanthaceae	Justicia carthaginensis		
Acanthaceae	Justicia chol	VU	
Acanthaceae	Justicia chrysostephana	EN	
Acanthaceae	Justicia clinopodium		
Acanthaceae	Justicia comosa		
Acanthaceae	Justicia cuneata		
Acanthaceae	Justicia cymulifera	EN	YES
Acanthaceae	Justicia ensiflora	VU	
Acanthaceae	Justicia fimbriata		
Acanthaceae	Justicia flava	EN	
Acanthaceae	Justicia fragilis	EN	
Acanthaceae	Justicia fulvicoma		
Acanthaceae	Justicia gonzalezii		
Acanthaceae	Justicia herpetacanthoides	VU	
Acanthaceae	Justicia hilsenbeckii		
Acanthaceae	Justicia incana		
Acanthaceae	Justicia jitotolana	EN	
Acanthaceae	Justicia kanal		
Acanthaceae	Justicia karsticola	CR	YES
Acanthaceae	Justicia leonardii		
Acanthaceae	Justicia lundellii		
Acanthaceae	Justicia macrantha		
Acanthaceae	Justicia maya	VU	
Acanthaceae	Justicia metallica	CR	
Acanthaceae	Justicia micrantha	EN	YES

Acanthaceae	Justicia mirandae			
Acanthaceae	Justicia multicaulis		VU	
Acanthaceae	Justicia nevlingii		VU	
Acanthaceae	Justicia pacifica			
Acanthaceae	Justicia palmeri			
Acanthaceae	Justicia periplocifolia			
Acanthaceae	Justicia petiolaris		EN	
Acanthaceae	Justicia pilosella			
Acanthaceae	Justicia pringlei			
Acanthaceae	Justicia ramosa			
Acanthaceae	Justicia rothschuhii		CR	
Acanthaceae	Justicia salviiflora			
Acanthaceae	Justicia soliana		VU	
Acanthaceae	Justicia spicigera			
Acanthaceae	Justicia tinctoriella		VU	
Acanthaceae	Justicia torresii		EN	
Acanthaceae	Justicia turipachensis		EN	YES
Acanthaceae	Justicia uxpanapensis		CR	YES
Acanthaceae	Justicia valvata		EN	
Acanthaceae	Justicia veracruzana			
Acanthaceae	Justicia vitzliputzli		EN	
Acanthaceae	Justicia wendtii		EN	
Acanthaceae	Lepidagathis alopecuroidea			
Acanthaceae	Lepidagathis chiapensis			
Acanthaceae	Lepidagathis guatemalensis		VU	
Acanthaceae	Lepidagathis uxpanapensis			
Acanthaceae	Louteridium brevicalyx		EN	
Acanthaceae	Louteridium donnell-smithii	EN		
Acanthaceae	Louteridium mexicanum	NT		
Acanthaceae	Louteridium parayi	EN		
Acanthaceae	Louteridium tamaulipense		EN	
Acanthaceae	Megaskepasma erythrochlamys			
Acanthaceae	Mendoncia guatemalensis		VU	
Acanthaceae	Mendoncia litoralis		CR	YES

Acanthaceae	Mendoncia retusa	
Acanthaceae	Mirandea huastecensis	EN
Acanthaceae	Mirandea sylvatica	
Acanthaceae	Nicoteba betonica	EN
Acanthaceae	Odontonema albiflorum	
Acanthaceae	Odontonema auriculatum	
Acanthaceae	Odontonema callistachyum	
Acanthaceae	Odontonema cuspidatum	
Acanthaceae	Odontonema glaberrimum	
Acanthaceae	Odontonema glabrum	
Acanthaceae	Odontonema tubaeforme	
Acanthaceae	Pachystachys lutea	
Acanthaceae	Phaulopsis imbricata	EN
Acanthaceae	Poikilacanthus capitatus	VU
Acanthaceae	Poikilacanthus macranthus	
Acanthaceae	Pseuderanthemum alatum	
Acanthaceae	Pseuderanthemum cuspidatum	
Acanthaceae	Pseuderanthemum maculatum	
Acanthaceae	Pseuderanthemum praecox	
Acanthaceae	Pseuderanthemum standleyi	
Acanthaceae	Pseuderanthemum verapazense	
Acanthaceae	Ruellia amoena	
Acanthaceae	Ruellia blechum	
Acanthaceae	Ruellia bourgaei	
Acanthaceae	Ruellia breedlovei	
Acanthaceae	Ruellia donnell-smithii	
Acanthaceae	Ruellia foetida	
Acanthaceae	Ruellia galeottii	
Acanthaceae	Ruellia geminiflora	
Acanthaceae	Ruellia harveyana	
Acanthaceae	Ruellia hookeriana	
Acanthaceae	Ruellia inundata	
Acanthaceae	Ruellia jussieuoides	
Acanthaceae	Ruellia lactea	

Acanthaceae	Ruellia lanatoglandulosa	
Acanthaceae	Ruellia latibracteata	
Acanthaceae	Ruellia longepetiolata	
Acanthaceae	Ruellia macrosiphon	EN
Acanthaceae	Ruellia matagalpae	
Acanthaceae	Ruellia matudae	
Acanthaceae	Ruellia maya	EN
Acanthaceae	Ruellia megasphaera	
Acanthaceae	Ruellia nudiflora	
Acanthaceae	Ruellia oaxacana	VU
Acanthaceae	Ruellia occidentalis	
Acanthaceae	Ruellia palmeri	
Acanthaceae	Ruellia paniculata	
Acanthaceae	Ruellia parva	
Acanthaceae	Ruellia pedunculata	EN
Acanthaceae	Ruellia pereducta	
Acanthaceae	Ruellia petiolaris	
Acanthaceae	Ruellia pilosa	
Acanthaceae	Ruellia pringlei	
Acanthaceae	Ruellia puberula	
Acanthaceae	Ruellia simplex	
Acanthaceae	Ruellia stemonacanthoides	
Acanthaceae	Ruellia terminalis	CR
Acanthaceae	Ruellia tuberosa	
Acanthaceae	Ruellia tuxtlensis	VU
Acanthaceae	Sanchezia oblonga	EN
Acanthaceae	Sanchezia parvibracteata	
Acanthaceae	Spathacanthus hahnianus	
Acanthaceae	Spathacanthus parviflorus	
Acanthaceae	Stenandrium chameranthemoideum	
Acanthaceae	Stenandrium dulce	
Acanthaceae	Stenandrium pedunculatum	
Acanthaceae	Stenandrium subcordatum	
Acanthaceae	Stenostephanus glaber	VU

Acanthaceae	Stenostephanus gracilis			
Acanthaceae	Stenostephanus haematodes			
Acanthaceae	Stenostephanus leiorhachis		EN	
Acanthaceae	Stenostephanus monolophus			
Acanthaceae	Stenostephanus silvaticus			
Acanthaceae	Streblacanthus parviflorus			
Acanthaceae	Tabascina lindenii		EN	
Acanthaceae	Tetramerium glandulosum			
Acanthaceae	Tetramerium langlassei			
Acanthaceae	Tetramerium nemorum			
Acanthaceae	Tetramerium nervosum			
Acanthaceae	Tetramerium oaxacanum		VU	
Acanthaceae	Tetramerium rzedowskii		VU	
Acanthaceae	Tetramerium tenuissimum			
Acanthaceae	Thunbergia alata			
Acanthaceae	Thunbergia erecta			
Acanthaceae	Thunbergia fragrans			
Acanthaceae	Thunbergia grandiflora			
Achariaceae	Chiangiodendron mexicanum	EN	VU	
Achariaceae	Mayna suaveolens	EN	CR	YES
Achatocarpaceae	Achatocarpus nigricans			
Achatocarpaceae	Achatocarpus oaxacanus	EN		
Achatocarpaceae	Phaulothamnus spinescens			
Actinidiaceae	Saurauia angustifolia			
Actinidiaceae	Saurauia aspera			
Actinidiaceae	Saurauia comitis-rossei			
Actinidiaceae	Saurauia conzattii			
Actinidiaceae	Saurauia cuchumatanensis			
Actinidiaceae	Saurauia glabra		CR	YES
Actinidiaceae	Saurauia kegeliana			
Actinidiaceae	Saurauia laevigata			
Actinidiaceae	Saurauia leucocarpa	VU		
Actinidiaceae	Saurauia madrensis			
Actinidiaceae	Saurauia matudae			

Actinidiaceae	Saurauia oreophila		VU	
Actinidiaceae	Saurauia pedunculata			
Actinidiaceae	Saurauia pringlei			
Actinidiaceae	Saurauia pustulata			VU
Actinidiaceae	Saurauia rubiformis			VU
Actinidiaceae	Saurauia scabrida			
Actinidiaceae	Saurauia selerorum			
Actinidiaceae	Saurauia serrata	NT	EN	
Actinidiaceae	Saurauia villosa		VU	
Actinidiaceae	Saurauia yasicae			
Actinidiaceae	Saurauia zahlbruckneri			EN
Aizoaceae	Mesembryanthemum cordifolium			
Aizoaceae	Sesuvium portulacastrum			
Aizoaceae	Trianthema portulacastrum			
Alismataceae	Aquarius grandiflorus			
Alismataceae	Aquarius subulatus			
Alismataceae	Echinodorus berteroi			
Alismataceae	Helanthium bolivianum			CR
Alismataceae	Helanthium tenellum			EN
Alismataceae	Hydrocleys parviflora	NT		EN
Alismataceae	Limnocharis laforestii			
Alismataceae	Sagittaria demersa			
Alismataceae	Sagittaria guayanensis subsp. guayanensis			
Alismataceae	Sagittaria lancifolia			
Alismataceae	Sagittaria lancifolia subsp. lancifolia			
Alismataceae	Sagittaria lancifolia subsp. media			
Alismataceae	Sagittaria longiloba			
Alismataceae	Sagittaria macrophylla	VU		
Alismataceae	Sagittaria montevidensis subsp. calycina			
Alstroemeriaceae	Bomarea acutifolia			
Alstroemeriaceae	Bomarea edulis			
Altingiaceae	Liquidambar styraciflua			
Amaranthaceae	Achyranthes aspera			
Amaranthaceae	Achyranthes aspera var. indica			

Amaranthaceae	Alternanthera bettzickiana		
Amaranthaceae	Alternanthera brasiliana		
Amaranthaceae	Alternanthera caracasana		
Amaranthaceae	Alternanthera flava		
Amaranthaceae	Alternanthera jacquinii	EN	
Amaranthaceae	Alternanthera laguroides		
Amaranthaceae	Alternanthera lanceolata		
Amaranthaceae	Alternanthera obovata		
Amaranthaceae	Alternanthera pungens		
Amaranthaceae	Alternanthera pycnantha		
Amaranthaceae	Alternanthera ramosissima		
Amaranthaceae	Alternanthera sessilis		
Amaranthaceae	Amaranthus caudatus		
Amaranthaceae	Amaranthus cruentus		
Amaranthaceae	Amaranthus hybridus		
Amaranthaceae	Amaranthus hypochondriacus		
Amaranthaceae	Amaranthus retroflexus		
Amaranthaceae	Amaranthus scariosus		
Amaranthaceae	Amaranthus spinosus		
Amaranthaceae	Amaranthus tricolor	CR	YES
Amaranthaceae	Atriplex semibaccata		
Amaranthaceae	Celosia argentea		
Amaranthaceae	Celosia nitida		
Amaranthaceae	Celosia virgata		
Amaranthaceae	Chamissoa acuminata var. swansonii		
Amaranthaceae	Chamissoa altissima		
Amaranthaceae	Chenopodiastrum murale		
Amaranthaceae	Chenopodium berlandieri		
Amaranthaceae	Cyathula achyranthoides		
Amaranthaceae	Dysphania ambrosioides		
Amaranthaceae	Dysphania incisa		
Amaranthaceae	Dysphania schraderiana		
Amaranthaceae	Froelichia interrupta		
Amaranthaceae	Gomphrena boliviana		

Amaranthaceae	Gomphrena filaginoides		
Amaranthaceae	Gomphrena globosa		
Amaranthaceae	Gomphrena pringlei		
Amaranthaceae	Gomphrena serrata		
Amaranthaceae	Hebanthe erianthos		
Amaranthaceae	Hebanthe grandiflora		
Amaranthaceae	Iresine angustifolia		
Amaranthaceae	Iresine arbuscula		
Amaranthaceae	Iresine borschii	CR	YES
Amaranthaceae	Iresine cassiniiformis		
Amaranthaceae	Iresine diffusa		
Amaranthaceae	Iresine diffusa f. herbstii		
Amaranthaceae	Iresine flavescens		
Amaranthaceae	Iresine hartmanii		
Amaranthaceae	Iresine hebanthoides		
Amaranthaceae	Iresine heterophylla		
Amaranthaceae	Iresine interrupta		
Amaranthaceae	Iresine latifolia		
Amaranthaceae	Iresine nigra		
Amaranthaceae	Iresine palmeri		
Amaranthaceae	Iresine pringlei		
Amaranthaceae	Iresine rhizomatosa		
Amaranthaceae	Iresine rotundifolia		
Amaranthaceae	Iresine schaffneri		
Amaranthaceae	Iresine valdesii	EN	
Amaranthaceae	Lagrezia monosperma		
Amaranthaceae	Oxybasis macrosperma		
Amaranthaceae	Oxybasis mexicana		
Amaranthaceae	Pfaffia iresinoides		
Amaranthaceae	Pleuropetalum sprucei		
Amaranthaceae	Salsola tragus		
Amaryllidaceae	Agapanthus africanus		
Amaryllidaceae	Agapanthus praecox		
Amaryllidaceae	Allium cepa		

Amaryllidaceae	Allium fistulosum		EN
Amaryllidaceae	Allium glandulosum		
Amaryllidaceae	Allium longifolium		
Amaryllidaceae	Allium neapolitanum		EN
Amaryllidaceae	Allium potosiense		VU
Amaryllidaceae	Amaryllis belladonna		
Amaryllidaceae	Crinum × amabile		EN
Amaryllidaceae	Crinum americanum		
Amaryllidaceae	Crinum erubescens		
Amaryllidaceae	Crinum moorei		
Amaryllidaceae	Hippeastrum elegans		EN
Amaryllidaceae	Hippeastrum puniceum		
Amaryllidaceae	Hymenocallis acutifolia		
Amaryllidaceae	Hymenocallis littoralis		
Amaryllidaceae	Hymenocallis partita		CR
Amaryllidaceae	Nothoscordum bivalve		
Amaryllidaceae	Nothoscordum gracile		
Amaryllidaceae	Scadoxus multiflorus		
Amaryllidaceae	Sprekelia formosissima		
Amaryllidaceae	Urceolina × grandiflora		EN
Amaryllidaceae	Zephyranthes brevipes		
Amaryllidaceae	Zephyranthes carinata		
Amaryllidaceae	Zephyranthes chlorosolen		
Amaryllidaceae	Zephyranthes clintiae		
Amaryllidaceae	Zephyranthes conzattii		EN
Amaryllidaceae	Zephyranthes drummondii		
Amaryllidaceae	Zephyranthes fosteri		
Amaryllidaceae	Zephyranthes lindleyana		
Amaryllidaceae	Zephyranthes macrosiphon		VU
Amaryllidaceae	Zephyranthes nelsonii		VU
Anacardiaceae	Actinocheita filicina		
Anacardiaceae	Amphipterygium adstringens	VU	
Anacardiaceae	Amphipterygium simplicifolium		
Anacardiaceae	Anacardium occidentale		

Anacardiaceae	Astronium graveolens	VU		
Anacardiaceae	Comocladia guatemalensis			
Anacardiaceae	Comocladia mollissima			
Anacardiaceae	Comocladia palmeri			
Anacardiaceae	Cyrtocarpa kruseana			
Anacardiaceae	Cyrtocarpa procera			
Anacardiaceae	Mangifera indica			
Anacardiaceae	Mauria heterophylla			EN
Anacardiaceae	Metopium brownei			
Anacardiaceae	Mosquitoxylum jamaicense			
Anacardiaceae	Pistacia mexicana			
Anacardiaceae	Pseudosmodingium andrieuxii			
Anacardiaceae	Rhus allophyloides			
Anacardiaceae	Rhus andrieuxii			
Anacardiaceae	Rhus aromatica			
Anacardiaceae	Rhus barclayi			
Anacardiaceae	Rhus chondroloma			
Anacardiaceae	Rhus chondroloma subsp. huajuapanensis			
Anacardiaceae	Rhus oaxacana			
Anacardiaceae	Rhus pachyrrhachis			
Anacardiaceae	Rhus rubifolia			
Anacardiaceae	Rhus schiedeana			
Anacardiaceae	Rhus schmidelioides			
Anacardiaceae	Rhus standleyi			
Anacardiaceae	Rhus terebinthifolia			
Anacardiaceae	Rhus trilobata			
Anacardiaceae	Rhus vestita			VU
Anacardiaceae	Rhus virens			
Anacardiaceae	Schinus molle			
Anacardiaceae	Schinus terebinthifolia			
Anacardiaceae	Spondias mombin			
Anacardiaceae	Spondias purpurea			
Anacardiaceae	Spondias radlkoferi	VU		
Anacardiaceae	Tapirira chimalapana		CR	VU

Anacardiaceae	Tapirira mexicana		
Anacardiaceae	Toxicodendron pubescens		
Anacardiaceae	Toxicodendron radicans		
Anacardiaceae	Toxicodendron radicans subsp. barkleyi		
Anacardiaceae	Toxicodendron striatum		
Annonaceae	Anaxagorea guatemalensis		
Annonaceae	Annona cherimola		
Annonaceae	Annona glabra		
Annonaceae	Annona globiflora		
Annonaceae	Annona liebmanniana		VU
Annonaceae	Annona longiflora		
Annonaceae	Annona macroprophyllata		
Annonaceae	Annona mucosa		
Annonaceae	Annona muricata		
Annonaceae	Annona palmeri		
Annonaceae	Annona purpurea		
Annonaceae	Annona rensoniana		
Annonaceae	Annona reticulata		
Annonaceae	Annona scleroderma		
Annonaceae	Annona squamosa		
Annonaceae	Cymbopetalum baillonii		
Annonaceae	Cymbopetalum gracile	EN	VU
Annonaceae	Cymbopetalum hintonii	EN	VU
Annonaceae	Cymbopetalum mayanum		
Annonaceae	Cymbopetalum penduliflorum		
Annonaceae	Cymbopetalum stenophyllum		
Annonaceae	Desmopsis lanceolata		
Annonaceae	Desmopsis mexicana	CR	EN
Annonaceae	Desmopsis trunciflora	VU	
Annonaceae	Desmopsis uxpanapensis	EN	EN
Annonaceae	Guatteria amplifolia		
Annonaceae	Guatteria costaricensis		CR
Annonaceae	Guatteria galeottiana	VU	
Annonaceae	Guatteria grandiflora	VU	

Annonaceae	Guatteria oliviformis		CR	
Annonaceae	Mosannona depressa			
Annonaceae	Mosannona depressa subsp. abscondita		VU	
Annonaceae	Oxandra lanceolata			
Annonaceae	Oxandra maya	VU	EN	
Annonaceae	Sapranthus campechianus			
Annonaceae	Sapranthus microcarpus			
Annonaceae	Sapranthus palanga		EN	
Annonaceae	Stenanona cauliflora	EN	CR	YES
Annonaceae	Stenanona flagelliflora	CR	EN	
Annonaceae	Stenanona humilis	EN	VU	
Annonaceae	Stenanona migueliana	CR	CR	YES
Annonaceae	Stenanona monticola	CR	EN	YES
Annonaceae	Stenanona stenopetala		EN	
Annonaceae	Stenanona zoque		CR	YES
Annonaceae	Tridimeris chiapensis	EN	CR	YES
Annonaceae	Tridimeris hahniana	EN	VU	
Annonaceae	Unonopsis mexicana		CR	YES
Annonaceae	Xylopia frutescens			
Apiaceae	Ammi majus			
Apiaceae	Anethum graveolens			
Apiaceae	Angelica nelsonii			
Apiaceae	Arracacia aegopodioides			
Apiaceae	Arracacia atropurpurea			
Apiaceae	Arracacia bracteata			
Apiaceae	Arracacia donnell-smithii		EN	
Apiaceae	Arracacia ebracteata			
Apiaceae	Arracacia filipes		VU	
Apiaceae	Arracacia hemsleyana		VU	
Apiaceae	Arracacia nelsonii			
Apiaceae	Arracacia pringlei			
Apiaceae	Arracacia ravenii		EN	
Apiaceae	Arracacia rigida			
Apiaceae	Arracacia ternata			

Apiaceae	Arracacia tolucensis		
Apiaceae	Arracacia tolucensis var. multifida		
Apiaceae	Berula erecta		
Apiaceae	Centella asiatica		
Apiaceae	Cicuta maculata		
Apiaceae	Conium maculatum		
Apiaceae	Coriandrum sativum		
Apiaceae	Coulterophytum laxum		VU
Apiaceae	Cyclospermum leptophyllum		
Apiaceae	Daucus carota		
Apiaceae	Daucus montanus		
Apiaceae	Donnellsmithia juncea		
Apiaceae	Donnellsmithia mexicana		
Apiaceae	Donnellsmithia serrata		
Apiaceae	Donnellsmithia ternata		
Apiaceae	Enantiophylla heydeana		
Apiaceae	Eryngium alternatum		
Apiaceae	Eryngium carlinae		
Apiaceae	Eryngium cymosum		
Apiaceae	Eryngium deppeanum		
Apiaceae	Eryngium foetidum		
Apiaceae	Eryngium ghiesbreghtii		
Apiaceae	Eryngium gracile		
Apiaceae	Eryngium guatemalense		EN
Apiaceae	Eryngium hemsleyanum		
Apiaceae	Eryngium longifolium		
Apiaceae	Eryngium monocephalum		
Apiaceae	Eryngium nasturtiifolium		
Apiaceae	Eryngium pectinatum		
Apiaceae	Eryngium proteiflorum	NT	
Apiaceae	Eryngium purpusii		
Apiaceae	Eryngium scaposum		
Apiaceae	Eryngium serratum		
Apiaceae	Eryngium strotheri		CR

Apiaceae	Eryngium tzeltal	EN
Apiaceae	Eryngium venustum	
Apiaceae	Foeniculum vulgare	
Apiaceae	Micropleura renifolia	
Apiaceae	Myrrhidendron donnellsmithii	VU
Apiaceae	Neogoezia minor	
Apiaceae	Osmorhiza mexicana	
Apiaceae	Ottoa oenanthoides	
Apiaceae	Petroselinum crispum	
Apiaceae	Prionosciadium nelsonii	
Apiaceae	Prionosciadium thapsoides	
Apiaceae	Prionosciadium watsonii	
Apiaceae	Rhodosciadium diffusum	
Apiaceae	Rhodosciadium glaucum	
Apiaceae	Rhodosciadium glaucum var. lineare	EN
Apiaceae	Rhodosciadium nelsonii	VU
Apiaceae	Rhodosciadium pringlei	
Apiaceae	Rhodosciadium tolucense	
Apiaceae	Sanicula liberta	
Apiaceae	Spananthe paniculata	
Apiaceae	Tauschia edulis	
Apiaceae	Tauschia filiformis	VU
Apiaceae	Tauschia seatonii	
Apiaceae	Tauschia vaginata	
Apiaceae	Trachyspermum ammi	VU
Apocynaceae	Allamanda blanchetii	
Apocynaceae	Allamanda cathartica	
Apocynaceae	Alstonia longifolia	
Apocynaceae	Ampelamus ligulatus	
Apocynaceae	Asclepias angustifolia	
Apocynaceae	Asclepias asperula	
Apocynaceae	Asclepias auriculata	
Apocynaceae	Asclepias coulteri	
Apocynaceae	Asclepias curassavica	

Apocynaceae	Asclepias elata		
Apocynaceae	Asclepias emoryi		
Apocynaceae	Asclepias fournieri		
Apocynaceae	Asclepias glaucescens		
Apocynaceae	Asclepias jaliscana		
Apocynaceae	Asclepias jorgeana		
Apocynaceae	Asclepias linaria		
Apocynaceae	Asclepias melantha		
Apocynaceae	Asclepias mexicana		
Apocynaceae	Asclepias oenotheroides		
Apocynaceae	Asclepias otarioides		
Apocynaceae	Asclepias ovata		
Apocynaceae	Asclepias scheryi		
Apocynaceae	Asclepias senecionifolia		
Apocynaceae	Asclepias similis		
Apocynaceae	Asclepias woodsoniana		
Apocynaceae	Aspidosperma desmanthum		
Apocynaceae	Aspidosperma megalocarpon		
Apocynaceae	Aspidosperma megalocarpon subsp. megalocarpon		
Apocynaceae	Aspidosperma spruceanum		
Apocynaceae	Aspidosperma steyermarkii	EN	
Apocynaceae	Blepharodon mucronatum		
Apocynaceae	Calotropis procera		
Apocynaceae	Cameraria latifolia		
Apocynaceae	Carissa macrocarpa		
Apocynaceae	Cascabela ovata		
Apocynaceae	Cascabela thevetia		
Apocynaceae	Cascabela thevetioides		
Apocynaceae	Catharanthus roseus		
Apocynaceae	Cynanchum chinense	CR	YES
Apocynaceae	Cynanchum cubense	EN	
Apocynaceae	Cynanchum foetidum		
Apocynaceae	Cynanchum jaliscanum		
Apocynaceae	Cynanchum peraffine		

Apocynaceae	Cynanchum racemosum		
Apocynaceae	Cynanchum racemosum var. unifarium		
Apocynaceae	Cynanchum surrubriflorum	EN	
Apocynaceae	Echites panduratus		
Apocynaceae	Echites tuxtlensis		
Apocynaceae	Echites umbellatus		
Apocynaceae	Echites woodsonianus		
Apocynaceae	Echites yucatanensis		
Apocynaceae	Fischeria scandens		
Apocynaceae	Fischeria stellata		
Apocynaceae	Forsteronia acouci		
Apocynaceae	Forsteronia myriantha		
Apocynaceae	Forsteronia spicata		
Apocynaceae	Funastrum bilobum		
Apocynaceae	Funastrum clausum		
Apocynaceae	Funastrum elegans		
Apocynaceae	Funastrum odoratum		
Apocynaceae	Funastrum pannosum		
Apocynaceae	Gomphocarpus physocarpus		
Apocynaceae	Gonolobus ancorifer	EN	
Apocynaceae	Gonolobus barbatus		
Apocynaceae	Gonolobus breedlovei	VU	
Apocynaceae	Gonolobus chloranthus		
Apocynaceae	Gonolobus cteniophorus		
Apocynaceae	Gonolobus dasystephanus	CR	YES
Apocynaceae	Gonolobus erianthus		
Apocynaceae	Gonolobus fraternus		
Apocynaceae	Gonolobus grandiflorus		
Apocynaceae	Gonolobus incerianus		
Apocynaceae	Gonolobus leianthus		
Apocynaceae	Gonolobus luridus	EN	
Apocynaceae	Gonolobus macranthus		
Apocynaceae	Gonolobus nemorosus		
Apocynaceae	Gonolobus niger		

Apocynaceae	Gonolobus pallidus	EN
Apocynaceae	Gonolobus pectinatus	
Apocynaceae	Gonolobus sororius	
Apocynaceae	Gonolobus stenosepalus	
Apocynaceae	Gonolobus uniflorus	
Apocynaceae	Gonolobus versicolor	EN
Apocynaceae	Gonolobus xanthotrichus	
Apocynaceae	Haplophyton cimicidum	
Apocynaceae	Hoya carnosa	
Apocynaceae	Irmischia angustifolia	
Apocynaceae	Laubertia contorta	
Apocynaceae	Macroscepis diademata	
Apocynaceae	Mandevilla acutiloba	
Apocynaceae	Mandevilla convolvulacea	
Apocynaceae	Mandevilla hirsuta	
Apocynaceae	Mandevilla hypoleuca	
Apocynaceae	Mandevilla mexicana	
Apocynaceae	Mandevilla oaxacana	
Apocynaceae	Mandevilla subsagittata	
Apocynaceae	Mandevilla subsessilis	
Apocynaceae	Mandevilla torosa	
Apocynaceae	Mandevilla tubiflora	
Apocynaceae	Mandevilla villosa	
Apocynaceae	Marsdenia astephanoides	
Apocynaceae	Marsdenia bourgaeana	
Apocynaceae	Marsdenia callosa	
Apocynaceae	Marsdenia coulteri	
Apocynaceae	Marsdenia gallardoae	
Apocynaceae	Marsdenia gualanensis	
Apocynaceae	Marsdenia gymnemoides	
Apocynaceae	Marsdenia hiriartiana	EN
Apocynaceae	Marsdenia lanata	
Apocynaceae	Marsdenia laxiflora	
Apocynaceae	Marsdenia mayana	

Apocynaceae	Marsdenia mexicana		
Apocynaceae	Marsdenia neriifolia		
Apocynaceae	Marsdenia parvifolia		
Apocynaceae	Marsdenia popoluca	EN	
Apocynaceae	Marsdenia pringlei		
Apocynaceae	Marsdenia propinqua		
Apocynaceae	Marsdenia rzedowskiana		
Apocynaceae	Marsdenia variifolia	EN	
Apocynaceae	Marsdenia zimapanica		
Apocynaceae	Matelea aspera		
Apocynaceae	Matelea calcarata	VU	
Apocynaceae	Matelea campechiana		
Apocynaceae	Matelea castanea	EN	
Apocynaceae	Matelea chrysantha		
Apocynaceae	Matelea crassifolia		
Apocynaceae	Matelea cyclophylla		
Apocynaceae	Matelea emmartinezii	EN	YES
Apocynaceae	Matelea gentlei		
Apocynaceae	Matelea gonoloboides		
Apocynaceae	Matelea guatemalensis	EN	
Apocynaceae	Matelea hamata		
Apocynaceae	Matelea inconspicua		
Apocynaceae	Matelea inops		
Apocynaceae	Matelea johnstonii	EN	
Apocynaceae	Matelea lanceolata		
Apocynaceae	Matelea magnifolia		
Apocynaceae	Matelea micrantha	VU	
Apocynaceae	Matelea molinarum	CR	YES
Apocynaceae	Matelea nummularia		
Apocynaceae	Matelea ocellata	VU	
Apocynaceae	Matelea pavonii		
Apocynaceae	Matelea pedunculata		
Apocynaceae	Matelea pilosa		
Apocynaceae	Matelea prosthecidiscus		

Apocynaceae	Matelea prostrata	
Apocynaceae	Matelea pusilliflora	VU
Apocynaceae	Matelea reticulata	
Apocynaceae	Matelea standleyana	
Apocynaceae	Matelea suberifera	
Apocynaceae	Matelea tezcatlipocantha	EN
Apocynaceae	Matelea tuerckheimii	EN
Apocynaceae	Matelea velutina	
Apocynaceae	Matelea velutinoides	
Apocynaceae	Mesechites trifidus	
Apocynaceae	Metastelma brachymischum	EN
Apocynaceae	Metastelma chiapense	
Apocynaceae	Metastelma lanceolatum	
Apocynaceae	Metastelma longicoronatum	VU
Apocynaceae	Metastelma palmeri	
Apocynaceae	Metastelma pedunculare	
Apocynaceae	Metastelma schlechtendalii	
Apocynaceae	Metastelma thalamosiphon	
Apocynaceae	Metastelma trichophyllum	
Apocynaceae	Nerium oleander	
Apocynaceae	Odontadenia semidigyna	VU
Apocynaceae	Orthosia bonplandiana	
Apocynaceae	Orthosia cynanchoides	EN
Apocynaceae	Orthosia glaberrima	VU
Apocynaceae	Orthosia misera	VU
Apocynaceae	Orthosia pubescens	
Apocynaceae	Oxypetalum cordifolium	
Apocynaceae	Pachypodium lamerei	
Apocynaceae	Pentalinon andrieuxii	
Apocynaceae	Pentalinon luteum	
Apocynaceae	Pherotrichis villosa	
Apocynaceae	Plumeria alba	
Apocynaceae	Plumeria obtusa	
Apocynaceae	Plumeria rubra	

Apocynaceae	Polystemma guatemalense			
Apocynaceae	Polystemma scopulorum			
Apocynaceae	Prestonia longifolia			
Apocynaceae	Prestonia mexicana			
Apocynaceae	Prestonia portobellensis			
Apocynaceae	Prestonia quinquangularis		EN	
Apocynaceae	Prestonia speciosa			
Apocynaceae	Rauvolfia ligustrina			
Apocynaceae	Rauvolfia tetraphylla			
Apocynaceae	Ruehssia macrophylla			
Apocynaceae	Tabernaemontana alba			
Apocynaceae	Tabernaemontana amygdalifolia			
Apocynaceae	Tabernaemontana arborea			
Apocynaceae	Tabernaemontana citrifolia			
Apocynaceae	Tabernaemontana divaricata			
Apocynaceae	Tabernaemontana donnell-smithii			
Apocynaceae	Tabernaemontana elegans		CR	YES
Apocynaceae	Tabernaemontana eubracteata			
Apocynaceae	Tabernaemontana glabra			
Apocynaceae	Tabernaemontana grandiflora			
Apocynaceae	Tabernaemontana hannae	VU		
Apocynaceae	Tabernaemontana litoralis			
Apocynaceae	Tabernaemontana ochoterenae		VU	
Apocynaceae	Tabernaemontana robinsonii		VU	
Apocynaceae	Tabernaemontana tomentosa			
Apocynaceae	Tabernaemontana venusta		CR	YES
Apocynaceae	Tassadia obovata		EN	
Apocynaceae	Thenardia chiapensis			
Apocynaceae	Thenardia galeottiana			
Apocynaceae	Thevetia ahouai			
Apocynaceae	Tintinnabularia mortonii		EN	
Apocynaceae	Vallesia aurantiaca			
Apocynaceae	Vinca major			
Apocynaceae	Wrightia tinctoria		VU	

Apodanthaceae	Pilostyles mexicana			
Aquifoliaceae	Ilex aquifolium		CR	YES
Aquifoliaceae	Ilex belizensis			
Aquifoliaceae	Ilex brandegeeana			
Aquifoliaceae	Ilex condensata	EN		
Aquifoliaceae	Ilex costaricensis		VU	
Aquifoliaceae	Ilex decidua			
Aquifoliaceae	Ilex discolor			
Aquifoliaceae	Ilex discolor var. tolucana			
Aquifoliaceae	Ilex guianensis			
Aquifoliaceae	Ilex liebmannii			
Aquifoliaceae	Ilex mitis		EN	
Aquifoliaceae	Ilex pringlei			
Aquifoliaceae	Ilex quercetorum	VU		
Aquifoliaceae	Ilex rubra			
Aquifoliaceae	Ilex servinii	CR	CR	
Aquifoliaceae	Ilex vomitoria			
Araceae	Alocasia cucullata		EN	
Araceae	Alocasia cuprea		CR	YES
Araceae	Alocasia macrorrhizos			
Araceae	Alocasia odora		VU	
Araceae	Anthurium × macrolobum		CR	YES
Araceae	Anthurium × robustum		CR	YES
Araceae	Anthurium andicola			
Araceae	Anthurium armeniense		EN	
Araceae	Anthurium bakeri		VU	
Araceae	Anthurium berriozabalense			
Araceae	Anthurium cerrobaulense			
Araceae	Anthurium cerropelonense		VU	
Araceae	Anthurium chamulense			
Araceae	Anthurium chamulense subsp. chamulense			
Araceae	Anthurium chiapasense			
Araceae	Anthurium chiapasense subsp. chiapasense			
Araceae	Anthurium chiapasense subsp. tlaxiacense			

Araceae	Anthurium clarinervium			
Araceae	Anthurium clavigerum		CR	
Araceae	Anthurium crassinervium			
Araceae	Anthurium cubense		EN	
Araceae	Anthurium davidsoniae		EN	YES
Araceae	Anthurium faustomirandae		VU	
Araceae	Anthurium flexile			
Araceae	Anthurium flexile subsp. flexile			
Araceae	Anthurium flexile subsp. muelleri			
Araceae	Anthurium halmoorei			
Araceae	Anthurium hookeri		CR	YES
Araceae	Anthurium huixtlense			
Araceae	Anthurium laciniosum		CR	YES
Araceae	Anthurium leuconeurum		EN	
Araceae	Anthurium lezamae			
Araceae	Anthurium longipeltatum			
Araceae	Anthurium lucens			
Araceae	Anthurium machetioides		EN	
Araceae	Anthurium macrospadix		CR	YES
Araceae	Anthurium microspadix			
Araceae	Anthurium montanum			
Araceae	Anthurium myosuroides			
Araceae	Anthurium nakamurae		VU	
Araceae	Anthurium nizandense		EN	
Araceae	Anthurium nymphaeifolium			
Araceae	Anthurium obtusum		EN	
Araceae	Anthurium parvispathum		VU	
Araceae	Anthurium pedatoradiatum			
Araceae	Anthurium pedatoradiatum subsp. helleborifolium			
Araceae	Anthurium pedatoradiatum subsp. pedatoradiatum			
Araceae	Anthurium pentaphyllum			
Araceae	Anthurium pentaphyllum var. bombacifolium			
Araceae	Anthurium podophyllum	VU		
Araceae	Anthurium protensum		EN	

Araceae	Anthurium rionegrense		EN
Araceae	Anthurium rzedowskii		
Araceae	Anthurium salvinii		
Araceae	Anthurium sarukhanianum		VU
Araceae	Anthurium scandens		
Araceae	Anthurium scandens subsp. pusillum		EN
Araceae	Anthurium scandens subsp. scandens		
Araceae	Anthurium schlechtendalii		
Araceae	Anthurium schlechtendalii subsp. jimenezii		
Araceae	Anthurium schlechtendalii subsp. schlechtendalii		
Araceae	Anthurium seleri		
Araceae	Anthurium titanium		
Araceae	Anthurium umbrosum		
Araceae	Anthurium verapazense		
Araceae	Arisaema dracontium		
Araceae	Arisaema macrospathum		
Araceae	Caladium bicolor		
Araceae	Colocasia esculenta		
Araceae	Dieffenbachia oerstedii		
Araceae	Dieffenbachia seguine	VU	
Araceae	Dieffenbachia wendlandii		
Araceae	Dracontium soconuscum		EN
Araceae	Epipremnum aureum		
Araceae	Lemna aequinoctialis		
Araceae	Lemna gibba		
Araceae	Lemna valdiviana		
Araceae	Monstera acacoyaguensis		
Araceae	Monstera acuminata		
Araceae	Monstera adansonii	VU	
Araceae	Monstera adansonii subsp. laniata		EN
Araceae	Monstera deliciosa		
Araceae	Monstera dubia		
Araceae	Monstera egregia		
Araceae	Monstera florescanoana		EN

Araceae	Monstera lechleriana			
Araceae	Monstera obliqua		VU	
Araceae	Monstera punctulata	VU		
Araceae	Monstera siltepecana			
Araceae	Monstera tuberculata	VU		
Araceae	Monstera tuberculata var. tuberculata		VU	
Araceae	Peltandra virginica		CR	
Araceae	Philodendron advena			
Araceae	Philodendron anisotomum			
Araceae	Philodendron aurantiifolium		VU	
Araceae	Philodendron aurantiifolium subsp. calderense		VU	
Araceae	Philodendron basii		VU	
Araceae	Philodendron breedlovei		CR	YES
Araceae	Philodendron glanduliferum		VU	
Araceae	Philodendron guttiferum			
Araceae	Philodendron hebetatum		VU	
Araceae	Philodendron hederaceum			
Araceae	Philodendron hederaceum var. hederaceum			
Araceae	Philodendron hederaceum var. kirkbridei		CR	YES
Araceae	Philodendron hederaceum var. oxycardium		VU	
Araceae	Philodendron inaequilaterum			
Araceae	Philodendron jacquinii			
Araceae	Philodendron jodavisianum		EN	
Araceae	Philodendron mexicanum			
Araceae	Philodendron purulhense		EN	
Araceae	Philodendron radiatum			
Araceae	Philodendron radiatum var. radiatum			
Araceae	Philodendron rojasianum		EN	
Araceae	Philodendron sagittifolium			
Araceae	Philodendron schottianum		CR	
Araceae	Philodendron schottii subsp. talamancae		VU	
Araceae	Philodendron seguine			
Araceae	Philodendron seguine subsp. lingua-bovis			
Araceae	Philodendron seguine subsp. seguine			

Araceae	Philodendron smithii			
Araceae	Philodendron sousae		VU	
Araceae	Philodendron standleyi			
Araceae	Philodendron tripartitum			
Araceae	Philodendron tuerckheimii		VU	
Araceae	Philodendron verapazense		CR	YES
Araceae	Philodendron warszewiczii			
Araceae	Pistia stratiotes			
Araceae	Rhodospatha wendlandii			
Araceae	Spathiphyllum blandum			
Araceae	Spathiphyllum brevirostre			
Araceae	Spathiphyllum cannifolium		CR	YES
Araceae	Spathiphyllum cochlearispathum			
Araceae	Spathiphyllum friedrichsthalii	VU		
Araceae	Spathiphyllum matudae			
Araceae	Spathiphyllum montanum		CR	YES
Araceae	Spathiphyllum ortgiesii			
Araceae	Spathiphyllum phryniifolium			
Araceae	Spathiphyllum wallisii			
Araceae	Stenospermation marantifolium		EN	
Araceae	Syngonium angustatum			
Araceae	Syngonium chiapense			
Araceae	Syngonium hoffmannii		EN	
Araceae	Syngonium macrophyllum			
Araceae	Syngonium neglectum			
Araceae	Syngonium podophyllum			
Araceae	Syngonium podophyllum var. podophyllum		VU	
Araceae	Syngonium sagittatum		VU	
Araceae	Syngonium salvadorense			
Araceae	Thaumatophyllum bipinnatifidum			
Araceae	Thaumatophyllum undulatum		EN	
Araceae	Wolffia brasiliensis			
Araceae	Xanthosoma mexicanum			
Araceae	Xanthosoma robustum			

Araceae	Xanthosoma sagittifolium		
Araceae	Zantedeschia aethiopica		
Araliaceae	Aralia humilis		
Araliaceae	Dendropanax arboreus		
Araliaceae	Dendropanax capillaris		EN
Araliaceae	Dendropanax gonatopodus		EN
Araliaceae	Dendropanax hondurensis	CR	EN
Araliaceae	Dendropanax leptopodus		
Araliaceae	Dendropanax oliganthus		EN
Araliaceae	Dendropanax pallidus		VU
Araliaceae	Dendropanax populifolius	EN	
Araliaceae	Didymopanax morototoni		
Araliaceae	Hedera helix		
Araliaceae	Heptapleurum arboricola		
Araliaceae	Hydrocotyle bonariensis		
Araliaceae	Hydrocotyle leucocephala		EN
Araliaceae	Hydrocotyle mexicana		
Araliaceae	Hydrocotyle ranunculoides		
Araliaceae	Hydrocotyle umbellata		
Araliaceae	Hydrocotyle verticillata		
Araliaceae	Oreopanax arcanus	VU	
Araliaceae	Oreopanax capitatus		
Araliaceae	Oreopanax echinops	VU	
Araliaceae	Oreopanax geminatus		
Araliaceae	Oreopanax guatemalensis		
Araliaceae	Oreopanax peltatus	VU	
Araliaceae	Oreopanax platyphyllus		VU
Araliaceae	Oreopanax sanderianus	VU	
Araliaceae	Oreopanax sandianus		EN
Araliaceae	Oreopanax xalapensis		
Arecaceae	Acrocomia aculeata		
Arecaceae	Adonidia merrillii	VU	
Arecaceae	Astrocaryum mexicanum		
Arecaceae	Attalea butyracea		

Arecaceae	Attalea rostrata				
Arecaceae	Bactris major	NT			
Arecaceae	Bactris major var. major				
Arecaceae	Bactris mexicana				
Arecaceae	Bactris mexicana var. mexicana			EN	
Arecaceae	Bactris mexicana var. trichophylla				
Arecaceae	Brahea calcarea				
Arecaceae	Brahea dulcis				
Arecaceae	Brahea moorei	NT			
Arecaceae	Brahea salvadorensis		CR	VU	
Arecaceae	Calyptrogyne ghiesbreghtiana			VU	
Arecaceae	Chamaedorea adscendens		VU	CR	YES
Arecaceae	Chamaedorea alternans	VU			
Arecaceae	Chamaedorea arenbergiana	VU			
Arecaceae	Chamaedorea carchensis	VU		EN	
Arecaceae	Chamaedorea cataractarum	VU			
Arecaceae	Chamaedorea costaricana				
Arecaceae	Chamaedorea elatior	VU			
Arecaceae	Chamaedorea elegans				
Arecaceae	Chamaedorea ernesti-augusti	VU			
Arecaceae	Chamaedorea geonomiformis	EN			
Arecaceae	Chamaedorea glaucifolia	EN	EN		
Arecaceae	Chamaedorea graminifolia	VU			
Arecaceae	Chamaedorea klotzschiana	NT	EN		
Arecaceae	Chamaedorea liebmannii	VU			
Arecaceae	Chamaedorea macrospadix			CR	YES
Arecaceae	Chamaedorea metallica	EN			
Arecaceae	Chamaedorea microspadix	VU			
Arecaceae	Chamaedorea neurochlamys				
Arecaceae	Chamaedorea nubium	VU			
Arecaceae	Chamaedorea oblongata	VU	VU		
Arecaceae	Chamaedorea oreophila	VU			
Arecaceae	Chamaedorea parvisecta	VU		CR	YES
Arecaceae	Chamaedorea pinnatifrons	VU			

Arecaceae	Chamaedorea plumosa		CR	EN
Arecaceae	Chamaedorea pochutlensis	VU		
Arecaceae	Chamaedorea radicalis			
Arecaceae	Chamaedorea rojasiana	VU		
Arecaceae	Chamaedorea sartorii	VU		
Arecaceae	Chamaedorea schiedeana	VU		
Arecaceae	Chamaedorea simplex	VU		EN
Arecaceae	Chamaedorea stolonifera	VU		VU
Arecaceae	Chamaedorea tepejilote			
Arecaceae	Chamaedorea tuerckheimii	EN		
Arecaceae	Chamaedorea warscewiczii			EN
Arecaceae	Chamaedorea whitelockiana	VU		EN
Arecaceae	Chamaedorea woodsoniana	VU		
Arecaceae	Cocos nucifera			
Arecaceae	Cryosophila nana	VU		
Arecaceae	Cryosophila stauracantha			
Arecaceae	Desmoncus chinantlensis			
Arecaceae	Desmoncus orthacanthos			
Arecaceae	Desmoncus polyacanthos			EN
Arecaceae	Dypsis lutescens			
Arecaceae	Elaeis guineensis			VU
Arecaceae	Gaussia gomez-pompae	VU	VU	VU
Arecaceae	Gaussia maya	VU	VU	
Arecaceae	Geonoma interrupta			
Arecaceae	Geonoma interrupta subsp. magnifica	VU		EN
Arecaceae	Geonoma pinnatifrons subsp. membranacea	VU		VU
Arecaceae	Geonoma pinnatifrons subsp. mexicana			VU
Arecaceae	Geonoma pinnatifrons subsp. oxycarpa			
Arecaceae	Geonoma undata subsp. edulis			
Arecaceae	Phoenix roebelenii			
Arecaceae	Reinhardtia elegans	VU		VU
Arecaceae	Reinhardtia gracilis	NT		
Arecaceae	Reinhardtia gracilis var. gracilior			
Arecaceae	Reinhardtia simplex			EN

Arecaceae	Roystonea dunlapiana	NT	EN		
Arecaceae	Roystonea regia	NT			
Arecaceae	Sabal mauritiiformis				
Arecaceae	Sabal mexicana				
Arecaceae	Sabal minor			VU	
Arecaceae	Syagrus romanzoffiana				
Arecaceae	Synechanthus fibrosus	EN			
Arecaceae	Washingtonia robusta				
Aristolochiaceae	Aristolochia amara			CR	YES
Aristolochiaceae	Aristolochia arborea				
Aristolochiaceae	Aristolochia asclepiadifolia				
Aristolochiaceae	Aristolochia cordiflora			CR	YES
Aristolochiaceae	Aristolochia grandiflora				
Aristolochiaceae	Aristolochia inflata				
Aristolochiaceae	Aristolochia littoralis				
Aristolochiaceae	Aristolochia malacophylla				
Aristolochiaceae	Aristolochia maxima				
Aristolochiaceae	Aristolochia nelsonii				
Aristolochiaceae	Aristolochia odoratissima				
Aristolochiaceae	Aristolochia orbicularis				
Aristolochiaceae	Aristolochia ovalifolia				
Aristolochiaceae	Aristolochia paracleta				
Aristolochiaceae	Aristolochia pentandra				
Aristolochiaceae	Aristolochia pilosa				
Aristolochiaceae	Aristolochia pueblana			VU	
Aristolochiaceae	Aristolochia rhizantha			EN	
Aristolochiaceae	Aristolochia schippii				
Aristolochiaceae	Aristolochia sericea			VU	
Aristolochiaceae	Aristolochia taliscana				
Aristolochiaceae	Aristolochia tentaculata				
Aristolochiaceae	Aristolochia tricaudata			VU	
Aristolochiaceae	Aristolochia veracruzana				
Asparagaceae	Agave americana				
Asparagaceae	Agave angustiarum				

Asparagaceae	Agave angustifolia				
Asparagaceae	Agave angustifolia var. angustifolia				
Asparagaceae	Agave angustifolia var. rubescens				
Asparagaceae	Agave applanata				
Asparagaceae	Agave atrovirens				
Asparagaceae	Agave attenuata				
Asparagaceae	Agave chiapensis	NT	VU		
Asparagaceae	Agave coetocapnia subsp. coetocapnia				
Asparagaceae	Agave congesta	NT		VU	
Asparagaceae	Agave convallis		VU		
Asparagaceae	Agave funkiana				
Asparagaceae	Agave ghiesbreghtii				
Asparagaceae	Agave gomezpompae		VU	CR	YES
Asparagaceae	Agave gracielae			EN	YES
Asparagaceae	Agave guiengola	EN	EN	VU	
Asparagaceae	Agave guttata				
Asparagaceae	Agave gypsophila	VU	CR	VU	
Asparagaceae	Agave hauniensis			VU	
Asparagaceae	Agave hiemiflora				
Asparagaceae	Agave horrida subsp. perotensis			EN	
Asparagaceae	Agave inaequidens				
Asparagaceae	Agave isthmensis		VU	EN	
Asparagaceae	Agave kerchovei		VU		
Asparagaceae	Agave kewensis	EN			
Asparagaceae	Agave maculata				
Asparagaceae	Agave marmorata				
Asparagaceae	Agave mitis				
Asparagaceae	Agave nizandensis	EN	CR	VU	
Asparagaceae	Agave obscura				
Asparagaceae	Agave pendula				
Asparagaceae	Agave polyacantha				
Asparagaceae	Agave potatorum		VU		
Asparagaceae	Agave salmiana				
Asparagaceae	Agave salmiana var. ferox			VU	

Asparagaceae	Agave scabra				
Asparagaceae	Agave scaposa				
Asparagaceae	Agave seemanniana				
Asparagaceae	Agave sisalana				
Asparagaceae	Agave striata				
Asparagaceae	Agave tenuifolia				
Asparagaceae	Agave titanota	VU	EN		
Asparagaceae	Agave triangularis		VU		
Asparagaceae	Agave variegata				
Asparagaceae	Agave vivipara		VU		
Asparagaceae	Agave warelliana		EN		
Asparagaceae	Agave xylonacantha				
Asparagaceae	Asparagus aethiopicus				
Asparagaceae	Asparagus declinatus			CR	YES
Asparagaceae	Asparagus setaceus				
Asparagaceae	Beaucarnea goldmanii	VU	VU		
Asparagaceae	Beaucarnea gracilis	VU	EN		
Asparagaceae	Beaucarnea purpusii		EN		
Asparagaceae	Beaucarnea recurvata	VU	CR		
Asparagaceae	Beaucarnea stricta	EN	VU		
Asparagaceae	Beschorneria albiflora	NT			
Asparagaceae	Beschorneria calcicola	VU			
Asparagaceae	Beschorneria rigida				
Asparagaceae	Beschorneria yuccoides				
Asparagaceae	Beschorneria yuccoides subsp. dekosteriana				
Asparagaceae	Chlorophytum comosum				
Asparagaceae	Cordyline fruticosa				
Asparagaceae	Cordyline stricta			VU	
Asparagaceae	Dasylirion acrotrichum	VU			
Asparagaceae	Dasylirion berlandieri				
Asparagaceae	Dasylirion lucidum				
Asparagaceae	Dasylirion quadrangulatum	VU			
Asparagaceae	Dracaena fragrans				
Asparagaceae	Dracaena trifasciata				

Asparagaceae	Echeandia albiflora		
Asparagaceae	Echeandia breedlovei		
Asparagaceae	Echeandia chiapensis		EN
Asparagaceae	Echeandia ciliata		EN
Asparagaceae	Echeandia echeandioides		
Asparagaceae	Echeandia flavescens		
Asparagaceae	Echeandia formosa		
Asparagaceae	Echeandia hallbergii		EN
Asparagaceae	Echeandia longipedicellata		
Asparagaceae	Echeandia matudae		EN
Asparagaceae	Echeandia mexicana		
Asparagaceae	Echeandia montealbanensis		EN
Asparagaceae	Echeandia nana		
Asparagaceae	Echeandia paniculata		
Asparagaceae	Echeandia parviflora		
Asparagaceae	Echeandia reflexa		
Asparagaceae	Echeandia skinneri		
Asparagaceae	Echeandia vestita		
Asparagaceae	Furcraea cabuya		
Asparagaceae	Furcraea guatemalensis		
Asparagaceae	Furcraea longaeva		
Asparagaceae	Furcraea parmentieri	VU	
Asparagaceae	Furcraea pubescens		
Asparagaceae	Furcraea quicheensis		VU
Asparagaceae	Furcraea samalana		
Asparagaceae	Hemiphylacus alatostylus		
Asparagaceae	Hemiphylacus latifolius		
Asparagaceae	Maianthemum amoenum		
Asparagaceae	Maianthemum flexuosum		
Asparagaceae	Maianthemum gigas		EN
Asparagaceae	Maianthemum gigas var. crassipes		EN
Asparagaceae	Maianthemum macrophyllum		
Asparagaceae	Maianthemum paniculatum		
Asparagaceae	Maianthemum scilloideum		

Asparagaceae	Milla biflora				
Asparagaceae	Nolina nelsonii				
Asparagaceae	Nolina parviflora		VU		
Asparagaceae	Nolina watsonii				
Asparagaceae	Yucca aloifolia				
Asparagaceae	Yucca carnerosana				
Asparagaceae	Yucca filamentosa			CR	YES
Asparagaceae	Yucca filifera				
Asparagaceae	Yucca gigantea				
Asparagaceae	Yucca lacandonica	EN	EN		
Asparagaceae	Yucca mixtecana		VU		
Asparagaceae	Yucca periculosa				
Asparagaceae	Yucca queretaroensis	EN	EN		
Asparagaceae	Yucca schidigera				
Asparagaceae	Yucca treculeana		VU		
Aspleniaceae	Asplenium abscissum				
Aspleniaceae	Asplenium aethiopicum				
Aspleniaceae	Asplenium africanum			CR	YES
Aspleniaceae	Asplenium alatum				
Aspleniaceae	Asplenium athyrioides				
Aspleniaceae	Asplenium auriculatum				
Aspleniaceae	Asplenium auritum	VU			
Aspleniaceae	Asplenium barbaense			VU	
Aspleniaceae	Asplenium breedlovei			VU	
Aspleniaceae	Asplenium castaneum				
Aspleniaceae	Asplenium cirrhatum			VU	
Aspleniaceae	Asplenium cristatum				
Aspleniaceae	Asplenium cuspidatum				
Aspleniaceae	Asplenium dentatum subsp. dentatum			EN	
Aspleniaceae	Asplenium dissectum			EN	
Aspleniaceae	Asplenium feei			VU	
Aspleniaceae	Asplenium flabellulatum				
Aspleniaceae	Asplenium formosum				
Aspleniaceae	Asplenium fragrans				

Aspleniaceae	Asplenium ghiesbreghtii			
Aspleniaceae	Asplenium hallbergii			
Aspleniaceae	Asplenium harpeodes			
Aspleniaceae	Asplenium hastatum		EN	
Aspleniaceae	Asplenium heterochroum		VU	
Aspleniaceae	Asplenium insolitum		EN	
Aspleniaceae	Asplenium juglandifolium		VU	
Aspleniaceae	Asplenium lamprocaulon		VU	
Aspleniaceae	Asplenium minimum			
Aspleniaceae	Asplenium miradorense			
Aspleniaceae	Asplenium monanthes			
Aspleniaceae	Asplenium monodon			
Aspleniaceae	Asplenium munchii		VU	
Aspleniaceae	Asplenium myriophyllum			
Aspleniaceae	Asplenium nigripes			
Aspleniaceae	Asplenium oblongatum		CR	YES
Aspleniaceae	Asplenium oligosorum		CR	YES
Aspleniaceae	Asplenium otites		EN	
Aspleniaceae	Asplenium palmeri			
Aspleniaceae	Asplenium perplexum			
Aspleniaceae	Asplenium polyphyllum			
Aspleniaceae	Asplenium potosinum			
Aspleniaceae	Asplenium praemorsum			
Aspleniaceae	Asplenium pteropus			
Aspleniaceae	Asplenium pulchellum		EN	YES
Aspleniaceae	Asplenium pumilum			
Aspleniaceae	Asplenium radicans			
Aspleniaceae	Asplenium resiliens			
Aspleniaceae	Asplenium rutaceum			
Aspleniaceae	Asplenium salicifolium			
Aspleniaceae	Asplenium serra			
Aspleniaceae	Asplenium serratum	VU		
Aspleniaceae	Asplenium sessilifolium			
Aspleniaceae	Asplenium sphaerosporum			

Aspleniaceae	Asplenium tenerrimum	VU	
Aspleniaceae	Asplenium tricholepis	CR	YES
Aspleniaceae	Asplenium tuerckheimii		
Aspleniaceae	Asplenium uniseriale		
Aspleniaceae	Asplenium virillae	EN	
Aspleniaceae	Athyrium filix-femina		
Aspleniaceae	Athyrium filix-femina subsp. filix-femina	CR	YES
Aspleniaceae	Athyrium palmense		
Aspleniaceae	Athyrium skinneri		
Aspleniaceae	Blechnum appendiculatum		
Aspleniaceae	Blechnum capense	EN	
Aspleniaceae	Blechnum divergens	VU	
Aspleniaceae	Blechnum ensiforme		
Aspleniaceae	Blechnum falciforme		
Aspleniaceae	Blechnum fragile		
Aspleniaceae	Blechnum ghiesbreghtii		
Aspleniaceae	Blechnum gracile		
Aspleniaceae	Blechnum occidentale		
Aspleniaceae	Blechnum polypodioides		
Aspleniaceae	Blechnum schiedeanum		
Aspleniaceae	Blechnum wardiae	VU	
Aspleniaceae	Cystopteris fragilis		
Aspleniaceae	Cystopteris membranifolia	VU	
Aspleniaceae	Diplazium altissimum	VU	
Aspleniaceae	Diplazium cristatum		
Aspleniaceae	Diplazium diplazioides	VU	
Aspleniaceae	Diplazium donnell-smithii		
Aspleniaceae	Diplazium drepanolobium		
Aspleniaceae	Diplazium expansum	VU	
Aspleniaceae	Diplazium franconis		
Aspleniaceae	Diplazium lindbergii		
Aspleniaceae	Diplazium lonchophyllum		
Aspleniaceae	Diplazium obscurum	VU	
Aspleniaceae	Diplazium plantaginifolium		

Aspleniaceae	Diplazium striatastrum		
Aspleniaceae	Diplazium striatum		
Aspleniaceae	Diplazium ternatum		
Aspleniaceae	Diplazium urticifolium		
Aspleniaceae	Diplazium werckleanum		
Aspleniaceae	Hemidictyum marginatum	VU	
Aspleniaceae	Hymenasplenium delitescens		
Aspleniaceae	Hymenasplenium hoffmannii		
Aspleniaceae	Hymenasplenium laetum	CR	YES
Aspleniaceae	Hymenasplenium repandulum	EN	
Aspleniaceae	Hymenasplenium riparium	EN	
Aspleniaceae	Hymenasplenium volubile	CR	YES
Aspleniaceae	Macrothelypteris torresiana		
Aspleniaceae	Telmatoblechnum serrulatum		
Aspleniaceae	Thelypteris albicaulis		
Aspleniaceae	Thelypteris atrovirens	VU	
Aspleniaceae	Thelypteris blanda		
Aspleniaceae	Thelypteris blepharis	EN	
Aspleniaceae	Thelypteris cinerea	VU	
Aspleniaceae	Thelypteris concinna		
Aspleniaceae	Thelypteris cretacea	VU	
Aspleniaceae	Thelypteris deflexa		
Aspleniaceae	Thelypteris dentata		
Aspleniaceae	Thelypteris ghiesbreghtii		
Aspleniaceae	Thelypteris glandulosa	CR	YES
Aspleniaceae	Thelypteris hatchii	VU	
Aspleniaceae	Thelypteris hispidula		
Aspleniaceae	Thelypteris hondurensis	CR	YES
Aspleniaceae	Thelypteris imbricata		
Aspleniaceae	Thelypteris interrupta		
Aspleniaceae	Thelypteris invisa	EN	
Aspleniaceae	Thelypteris lanosa	VU	
Aspleniaceae	Thelypteris meniscioides		
Aspleniaceae	Thelypteris minor	EN	

Aspleniaceae	Thelypteris nicaraguensis	EN	YES
Aspleniaceae	Thelypteris normalis		
Aspleniaceae	Thelypteris obliterata	VU	
Aspleniaceae	Thelypteris oligocarpa		
Aspleniaceae	Thelypteris ovata		
Aspleniaceae	Thelypteris patens		
Aspleniaceae	Thelypteris paucipinnata		
Aspleniaceae	Thelypteris pilosa		
Aspleniaceae	Thelypteris pilosohispida		
Aspleniaceae	Thelypteris pilosula		
Aspleniaceae	Thelypteris poiteana	VU	
Aspleniaceae	Thelypteris puberula		
Aspleniaceae	Thelypteris reptans		
Aspleniaceae	Thelypteris resiliens		
Aspleniaceae	Thelypteris resinifera		
Aspleniaceae	Thelypteris reticulata	EN	
Aspleniaceae	Thelypteris rudis		
Aspleniaceae	Thelypteris scalaris	VU	
Aspleniaceae	Thelypteris schaffneri		
Aspleniaceae	Thelypteris serrata		
Aspleniaceae	Thelypteris stolzeana	CR	YES
Aspleniaceae	Thelypteris tetragona		
Aspleniaceae	Thelypteris thomsonii	EN	
Aspleniaceae	Thelypteris toganetra	VU	
Aspleniaceae	Thelypteris tuerckheimii	VU	
Aspleniaceae	Woodsia mexicana		
Aspleniaceae	Woodsia mollis		
Aspleniaceae	Woodwardia × semicordata		
Aspleniaceae	Woodwardia martinezii		
Aspleniaceae	Woodwardia radicans	EN	
Aspleniaceae	Woodwardia spinulosa		
Asteraceae	Achillea millefolium		
Asteraceae	Achyrocline deflexa		
Asteraceae	Achyrocline vargasiana	VU	

Asteraceae	Acmella alba		
Asteraceae	Acmella brachyglossa	VU	
Asteraceae	Acmella decumbens	CR	YES
Asteraceae	Acmella pilosa		
Asteraceae	Acmella radicans		
Asteraceae	Acmella repens		
Asteraceae	Acourtia caltepecana	VU	
Asteraceae	Acourtia cordata		
Asteraceae	Acourtia coulteri		
Asteraceae	Acourtia cuernavacana	VU	
Asteraceae	Acourtia dugesii		
Asteraceae	Acourtia dugesii var. veracruzana		
Asteraceae	Acourtia glandulifera	EN	
Asteraceae	Acourtia gracilis	CR	YES
Asteraceae	Acourtia lepidopoda	VU	
Asteraceae	Acourtia lobulata	VU	
Asteraceae	Acourtia nudicaulis		
Asteraceae	Acourtia reticulata		
Asteraceae	Acourtia scapiformis		
Asteraceae	Acourtia scaposa	CR	
Asteraceae	Adenocaulon lyratum	VU	
Asteraceae	Adenophyllum appendiculatum		
Asteraceae	Adenophyllum aurantium		
Asteraceae	Adenophyllum glandulosum		
Asteraceae	Adenophyllum porophyllum		
Asteraceae	Adenophyllum porophyllum var. radiatum		
Asteraceae	Adenophyllum speciosum		
Asteraceae	Ageratina adenophora		
Asteraceae	Ageratina altissima		
Asteraceae	Ageratina areolaris		
Asteraceae	Ageratina arsenei		
Asteraceae	Ageratina atrocordata		
Asteraceae	Ageratina brevipes		
Asteraceae	Ageratina calaminthifolia		

Asteraceae	Ageratina calophylla	
Asteraceae	Ageratina capillipes	VU
Asteraceae	Ageratina choricephala	
Asteraceae	Ageratina collodes	
Asteraceae	Ageratina crassiramea	
Asteraceae	Ageratina deltoidea	
Asteraceae	Ageratina espinosarum	
Asteraceae	Ageratina glabrata	
Asteraceae	Ageratina glauca	VU
Asteraceae	Ageratina grandifolia	
Asteraceae	Ageratina havanensis	
Asteraceae	Ageratina hidalgensis	
Asteraceae	Ageratina liebmannii	
Asteraceae	Ageratina ligustrina	
Asteraceae	Ageratina macdonaldii	EN
Asteraceae	Ageratina mairetiana	
Asteraceae	Ageratina malacolepis	
Asteraceae	Ageratina miahuatlana	VU
Asteraceae	Ageratina muelleri	
Asteraceae	Ageratina oligocephala	
Asteraceae	Ageratina ovilla	VU
Asteraceae	Ageratina parayana	
Asteraceae	Ageratina pazcuarensis	
Asteraceae	Ageratina petiolaris	
Asteraceae	Ageratina pichinchensis	
Asteraceae	Ageratina pichinchensis var. bustamenta	
Asteraceae	Ageratina pringlei	
Asteraceae	Ageratina rhomboidea	
Asteraceae	Ageratina riparia	
Asteraceae	Ageratina rivalis	
Asteraceae	Ageratina saltillensis	
Asteraceae	Ageratina saxorum	EN
Asteraceae	Ageratina schaffneri	
Asteraceae	Ageratina scorodonioides	

Asteraceae	Ageratina subinclusa		
Asteraceae	Ageratina tomentella		
Asteraceae	Ageratina vernalis		
Asteraceae	Ageratina zunilana	EN	
Asteraceae	Ageratum conyzoides		
Asteraceae	Ageratum corymbosum		
Asteraceae	Ageratum echioides		
Asteraceae	Ageratum elassocarpum		
Asteraceae	Ageratum houstonianum		
Asteraceae	Ageratum microcephalum		
Asteraceae	Ageratum paleaceum		
Asteraceae	Ageratum rugosum		
Asteraceae	Ageratum scorpioideum	CR	YES
Asteraceae	Ageratum tehuacanum		
Asteraceae	Ageratum tomentosum		
Asteraceae	Aldama apiculata	EN	
Asteraceae	Aldama canescens		
Asteraceae	Aldama dentata		
Asteraceae	Aldama excelsa		
Asteraceae	Aldama ghiesbreghtii		
Asteraceae	Aldama michoacana		
Asteraceae	Alepidocline annua	EN	
Asteraceae	Alepidocline breedlovei	EN	
Asteraceae	Alepidocline trifida		
Asteraceae	Alloispermum integrifolium		
Asteraceae	Alloispermum longiradiatum		
Asteraceae	Alloispermum michoacanum		
Asteraceae	Alloispermum michoacanum var. liebmannii		
Asteraceae	Alloispermum scabrum		
Asteraceae	Ambrosia ambrosioides		
Asteraceae	Ambrosia artemisiifolia		
Asteraceae	Ambrosia bryantii		
Asteraceae	Ambrosia cumanensis		
Asteraceae	Ambrosia peruviana		

Asteraceae	Amolinia heydeana	VU	
Asteraceae	Anaphalis bicolor		
Asteraceae	Anaphalis margaritacea subsp. margaritacea		
Asteraceae	Aphanostephus ramosissimus		
Asteraceae	Aphanostephus ramosissimus var. humilis		
Asteraceae	Aphanostephus ramosissimus var. ramosus		
Asteraceae	Aquilula riskindii		
Asteraceae	Archibaccharis androgyna		
Asteraceae	Archibaccharis asperifolia		
Asteraceae	Archibaccharis auriculata		
Asteraceae	Archibaccharis flexilis		
Asteraceae	Archibaccharis hirtella		
Asteraceae	Archibaccharis intermedia		
Asteraceae	Archibaccharis macdonaldii	EN	
Asteraceae	Archibaccharis salmeoides	VU	
Asteraceae	Archibaccharis schiedeana		
Asteraceae	Archibaccharis serratifolia		
Asteraceae	Archibaccharis simplex	VU	
Asteraceae	Archibaccharis subsessilis		
Asteraceae	Archibaccharis taeniotricha	VU	
Asteraceae	Archibaccharis trichotoma		
Asteraceae	Argyranthemum frutescens		
Asteraceae	Artemisia absinthium		
Asteraceae	Artemisia klotzschiana		
Asteraceae	Artemisia ludoviciana		
Asteraceae	Artemisia ludoviciana subsp. mexicana		
Asteraceae	Astranthium purpurascens		
Asteraceae	Astranthium xanthocomoides		
Asteraceae	Baccharis conferta		
Asteraceae	Baccharis glandulifera		
Asteraceae	Baccharis glutinosa		
Asteraceae	Baccharis heterophylla		
Asteraceae	Baccharis lancifolia		
Asteraceae	Baccharis ligustrina	CR	YES

Asteraceae	Baccharis mexicana	
Asteraceae	Baccharis multiflora	
Asteraceae	Baccharis pedunculata	
Asteraceae	Baccharis pseudovaccinioides	
Asteraceae	Baccharis pteronioides	
Asteraceae	Baccharis salicifolia	
Asteraceae	Baccharis salicifolia subsp. salicifolia	
Asteraceae	Baccharis serrifolia	
Asteraceae	Baccharis sordescens	
Asteraceae	Baccharis thesioides	
Asteraceae	Baccharis trinervis	
Asteraceae	Baltimora recta	
Asteraceae	Barkleyanthus salicifolius	
Asteraceae	Bartlettina breedlovei	
Asteraceae	Bartlettina hylobia	EN
Asteraceae	Bartlettina karwinskiana	
Asteraceae	Bartlettina lanicaulis	
Asteraceae	Bartlettina luxii	VU
Asteraceae	Bartlettina macdougallii	
Asteraceae	Bartlettina oresbia	
Asteraceae	Bartlettina oresbioides	VU
Asteraceae	Bartlettina ornata	VU
Asteraceae	Bartlettina pansamalensis	
Asteraceae	Bartlettina pinabetensis	VU
Asteraceae	Bartlettina platyphylla	
Asteraceae	Bartlettina sordida	
Asteraceae	Bartlettina tuerckheimii	
Asteraceae	Bidens alba	
Asteraceae	Bidens anthemoides	
Asteraceae	Bidens anthriscoides	EN
Asteraceae	Bidens aurea	
Asteraceae	Bidens bicolor	
Asteraceae	Bidens chiapensis	
Asteraceae	Bidens chrysanthemifolia	VU

Asteraceae	Bidens hintonii	VU
Asteraceae	Bidens mexicana	
Asteraceae	Bidens odorata	
Asteraceae	Bidens ostruthioides	
Asteraceae	Bidens pilosa	
Asteraceae	Bidens reptans	
Asteraceae	Bidens riparia	
Asteraceae	Bidens serrulata	
Asteraceae	Bidens sharpii	
Asteraceae	Bidens sharpii var. tamazulapana	VU
Asteraceae	Bidens tenuisecta	
Asteraceae	Bidens triplinervia	
Asteraceae	Brenandendron donianum	EN
Asteraceae	Brickellia argyrolepis	
Asteraceae	Brickellia californica	
Asteraceae	Brickellia cavanillesii	
Asteraceae	Brickellia coahuilensis	
Asteraceae	Brickellia coulteri	
Asteraceae	Brickellia coulteri var. adenopoda	
Asteraceae	Brickellia diffusa	
Asteraceae	Brickellia eupatorioides	
Asteraceae	Brickellia eupatorioides var. chlorolepis	
Asteraceae	Brickellia filipes	
Asteraceae	Brickellia glandulosa	
Asteraceae	Brickellia kellermanii	VU
Asteraceae	Brickellia lemmonii	
Asteraceae	Brickellia lemmonii var. conduplicata	
Asteraceae	Brickellia lemmonii var. nelsonii	
Asteraceae	Brickellia nutanticeps	
Asteraceae	Brickellia oliganthes	
Asteraceae	Brickellia orizabaensis	
Asteraceae	Brickellia paniculata	
Asteraceae	Brickellia pedunculosa	
Asteraceae	Brickellia pendula	

Asteraceae	Brickellia robinsoniana	EN	
Asteraceae	Brickellia scoparia		
Asteraceae	Brickellia secundiflora		
Asteraceae	Brickellia squarrosa		
Asteraceae	Brickellia subuligera		
Asteraceae	Brickellia tomentella		
Asteraceae	Brickellia veronicifolia		
Asteraceae	Calanticaria bicolor		
Asteraceae	Calea jamaicensis		
Asteraceae	Calea longipedicellata		
Asteraceae	Calea megacephala		
Asteraceae	Calea oaxacana	VU	
Asteraceae	Calea oxylepis	CR	YES
Asteraceae	Calea ternifolia		
Asteraceae	Calea trichotoma		
Asteraceae	Calea urticifolia		
Asteraceae	Calendula officinalis		
Asteraceae	Calyptocarpus vialis		
Asteraceae	Calyptocarpus wendlandii		
Asteraceae	Campuloclinium macrocephalum	EN	
Asteraceae	Carminatia papagayana	CR	
Asteraceae	Carminatia recondita		
Asteraceae	Carminatia tenuiflora		
Asteraceae	Carphochaete grahamii		
Asteraceae	Centratherum punctatum		
Asteraceae	Chaetopappa asteroides	EN	
Asteraceae	Chaetopappa ericoides		
Asteraceae	Chaptalia albicans	VU	
Asteraceae	Chaptalia madrensis		
Asteraceae	Chaptalia nutans		
Asteraceae	Chaptalia pringlei		
Asteraceae	Chaptalia texana		
Asteraceae	Chaptalia transiliens		
Asteraceae	Chloracantha spinosa		

Asteraceae	Chromolaena breedlovei		
Asteraceae	Chromolaena collina		
Asteraceae	Chromolaena glaberrima		
Asteraceae	Chromolaena guiengolensis	EN	
Asteraceae	Chromolaena ivifolia	VU	
Asteraceae	Chromolaena laevigata		
Asteraceae	Chromolaena leucocephala		
Asteraceae	Chromolaena lundellii		
Asteraceae	Chromolaena misella	EN	
Asteraceae	Chromolaena odorata		
Asteraceae	Chromolaena opadoclinia		
Asteraceae	Chromolaena ovaliflora		
Asteraceae	Chromolaena pulchella		
Asteraceae	Chromolaena quercetorum		
Asteraceae	Chromolaena tamaulipasensis	VU	
Asteraceae	Chrysactinia mexicana		
Asteraceae	Chrysactinia pinnata		
Asteraceae	Chrysanthellum americanum	VU	
Asteraceae	Chrysanthellum indicum		
Asteraceae	Chrysanthellum indicum subsp. mexicanum		
Asteraceae	Chrysanthellum integrifolium	VU	
Asteraceae	Chrysanthemum × morifolium		
Asteraceae	Chrysanthemum indicum	EN	
Asteraceae	Cirsium anartiolepis		
Asteraceae	Cirsium conspicuum		
Asteraceae	Cirsium ehrenbergii		
Asteraceae	Cirsium engelmannii	CR	YES
Asteraceae	Cirsium excelsius	VU	
Asteraceae	Cirsium faucium		
Asteraceae	Cirsium horridulum		
Asteraceae	Cirsium lappoides		
Asteraceae	Cirsium mexicanum		
Asteraceae	Cirsium nigriceps	EN	
Asteraceae	Cirsium pinetorum		

Asteraceae	Cirsium radians	VU	
Asteraceae	Cirsium subcoriaceum		
Asteraceae	Cirsium subuliforme		
Asteraceae	Cirsium vulgare		
Asteraceae	Clibadium arboreum		
Asteraceae	Clibadium surinamense		
Asteraceae	Comaclinium montanum		
Asteraceae	Conoclinium betonicifolium		
Asteraceae	Conoclinium coelestinum		
Asteraceae	Coreopsis davilae	EN	YES
Asteraceae	Coreopsis rhyacophila		
Asteraceae	Cosmos atrosanguineus		
Asteraceae	Cosmos bipinnatus		
Asteraceae	Cosmos caudatus		
Asteraceae	Cosmos crithmifolius		
Asteraceae	Cosmos diversifolius		
Asteraceae	Cosmos linearifolius		
Asteraceae	Cosmos parviflorus		
Asteraceae	Cosmos scabiosoides		
Asteraceae	Cosmos sulphureus		
Asteraceae	Cotula australis		
Asteraceae	Critonia bartlettii	EN	
Asteraceae	Critonia billbergiana	EN	
Asteraceae	Critonia breedlovei	EN	
Asteraceae	Critonia daleoides		
Asteraceae	Critonia hebebotrya		
Asteraceae	Critonia hospitalis		
Asteraceae	Critonia iltisii	EN	
Asteraceae	Critonia lozanoana		
Asteraceae	Critonia morifolia		
Asteraceae	Critonia quadrangularis		
Asteraceae	Critonia sexangularis		
Asteraceae	Critonia siltepecana	EN	YES
Asteraceae	Critonia tuxtlae	EN	

Asteraceae	Critonia yashanalensis		EN	
Asteraceae	Critoniadelphus microdon		EN	
Asteraceae	Critoniadelphus nubigenus			
Asteraceae	Critoniopsis uniflora			
Asteraceae	Critoniopsis uniflosculosa		EN	
Asteraceae	Cyanthillium cinereum			
Asteraceae	Cyrtocymura scorpioides			
Asteraceae	Dahlia apiculata			
Asteraceae	Dahlia australis			
Asteraceae	Dahlia coccinea			
Asteraceae	Dahlia excelsa			
Asteraceae	Dahlia imperialis			
Asteraceae	Dahlia merckii			
Asteraceae	Dahlia pinnata			
Asteraceae	Dahlia pteropoda		VU	
Asteraceae	Dahlia scapigera	NT		
Asteraceae	Dahlia sorensenii			
Asteraceae	Dahlia tamaulipana		EN	
Asteraceae	Dahlia tenuicaulis	NT		
Asteraceae	Davilanthus davilae		EN	YES
Asteraceae	Davilanthus sericeus			
Asteraceae	Decachaeta haenkeana			
Asteraceae	Decachaeta incompta			
Asteraceae	Decachaeta ovandensis		VU	
Asteraceae	Decachaeta perornata			
Asteraceae	Delilia biflora			
Asteraceae	Dendroviguiera sphaerocephala			
Asteraceae	Desmanthodium fruticosum			
Asteraceae	Desmanthodium perfoliatum			
Asteraceae	Digitacalia chiapensis		VU	
Asteraceae	Digitacalia hintoniorum		EN	
Asteraceae	Digitacalia jatrophoides			
Asteraceae	Doellingeria umbellata		EN	
Asteraceae	Dugesia mexicana			

Asteraceae	Dyssodia decipiens	
Asteraceae	Dyssodia papposa	
Asteraceae	Dyssodia pinnata	
Asteraceae	Dyssodia tagetiflora	
Asteraceae	Eclipta prostrata	
Asteraceae	Egletes liebmannii	
Asteraceae	Egletes viscosa	
Asteraceae	Electranthera mutica	
Asteraceae	Electranthera mutica var. carnosifolia	
Asteraceae	Electranthera mutica var. holotricha	
Asteraceae	Electranthera mutica var. miahuatlana	VU
Asteraceae	Electranthera mutica var. microcephala	
Asteraceae	Electranthera parvifolia	
Asteraceae	Elephantopus mollis	
Asteraceae	Elephantopus scaber	VU
Asteraceae	Elephantopus tomentosus	VU
Asteraceae	Emilia fosbergii	
Asteraceae	Emilia sonchifolia	
Asteraceae	Epaltes mexicana	
Asteraceae	Erechtites hieraciifolius	
Asteraceae	Erechtites runcinatus	
Asteraceae	Erechtites valerianifolius	
Asteraceae	Eremosis corymbosa	
Asteraceae	Eremosis heydeana	
Asteraceae	Eremosis leiocarpa	
Asteraceae	Eremosis macvaughii	
Asteraceae	Eremosis obtusa	
Asteraceae	Eremosis oolepis	
Asteraceae	Eremosis pallens	
Asteraceae	Eremosis shannonii	EN
Asteraceae	Eremosis tarchonanthifolia	
Asteraceae	Eremosis tomentosa	
Asteraceae	Eremosis triflosculosa	
Asteraceae	Erigeron annuus	EN

Asteraceae	Erigeron bonariensis		
Asteraceae	Erigeron canadensis		
Asteraceae	Erigeron columnaris		
Asteraceae	Erigeron flagellaris		
Asteraceae	Erigeron floribundus		
Asteraceae	Erigeron glacialis		
Asteraceae	Erigeron heteromorphus	VU	
Asteraceae	Erigeron karvinskianus		
Asteraceae	Erigeron laevigatus		
Asteraceae	Erigeron longipes		
Asteraceae	Erigeron palmeri		
Asteraceae	Erigeron pubescens		
Asteraceae	Erigeron quiexobrensis	EN	
Asteraceae	Erigeron sumatrensis		
Asteraceae	Erigeron variifolius		
Asteraceae	Erigeron velutipes		
Asteraceae	Erigeron veracruzensis		
Asteraceae	Espejoa mexicana		
Asteraceae	Eupatoriastrum angulifolium	VU	
Asteraceae	Eupatoriastrum corvi		
Asteraceae	Eupatoriastrum triangulare		
Asteraceae	Eupatorium leucolepis	CR	YES
Asteraceae	Euphrosyne partheniifolia		
Asteraceae	Eutetras pringlei		
Asteraceae	Flaveria pringlei		
Asteraceae	Flaveria trinervia		
Asteraceae	Fleischmannia arguta		
Asteraceae	Fleischmannia blakei	EN	
Asteraceae	Fleischmannia bohlmanniana	VU	
Asteraceae	Fleischmannia holwayana	VU	
Asteraceae	Fleischmannia imitans		
Asteraceae	Fleischmannia incarnata		
Asteraceae	Fleischmannia matudae		
Asteraceae	Fleischmannia porphyranthema		

Asteraceae	Fleischmannia pratensis		
Asteraceae	Fleischmannia pycnocephala		
Asteraceae	Fleischmannia pycnocephaloides		
Asteraceae	Fleischmannia seleriana		
Asteraceae	Fleischmannia sideritidis	CR	YES
Asteraceae	Fleischmannia sinclairii		
Asteraceae	Fleischmannia sonorae		
Asteraceae	Fleischmanniopsis leucocephala		
Asteraceae	Florestina latifolia		
Asteraceae	Florestina pedata		
Asteraceae	Florestina platyphylla		
Asteraceae	Florestina simplicifolia		
Asteraceae	Florestina tripteris		
Asteraceae	Flourensia collodes		
Asteraceae	Flourensia glutinosa		
Asteraceae	Flyriella harrimanii	CR	
Asteraceae	Galeana pratensis		
Asteraceae	Galinsoga parviflora		
Asteraceae	Galinsoga quadriradiata		
Asteraceae	Gamochaeta americana		
Asteraceae	Gamochaeta purpurea		
Asteraceae	Gamochaeta sphacelata		
Asteraceae	Geissolepis suaedifolia	EN	
Asteraceae	Glebionis coronaria		
Asteraceae	Gnaphaliothamnus aecidiocephalus	EN	
Asteraceae	Gnaphaliothamnus lavandulifolius		
Asteraceae	Gnaphaliothamnus salicifolius		
Asteraceae	Goldmanella sarmentosa		
Asteraceae	Grindelia inuloides		
Asteraceae	Grindelia microcephala	EN	
Asteraceae	Grindelia oaxacana	EN	
Asteraceae	Grindelia oxylepis		
Asteraceae	Grindelia subdecurrens		
Asteraceae	Gutierrezia microcephala		

Asteraceae	Gymnocoronis latifolia	
Asteraceae	Gymnolaena chiapasana	
Asteraceae	Gymnosperma glutinosum	
Asteraceae	Gynura aurantiaca	VU
Asteraceae	Harleya oxylepis	VU
Asteraceae	Hebeclinium macrophyllum	
Asteraceae	Helenium elegans	
Asteraceae	Helenium elegans var. amphibolum	
Asteraceae	Helenium mexicanum	
Asteraceae	Helenium microcephalum	
Asteraceae	Helenium quadridentatum	
Asteraceae	Helenium scorzonerifolium	
Asteraceae	Helianthella gypsophila	
Asteraceae	Helianthus annuus	
Asteraceae	Heliopsis annua	
Asteraceae	Heliopsis buphthalmoides	
Asteraceae	Heliopsis parvifolia	
Asteraceae	Helminthotheca echioides	
Asteraceae	Heterosperma pinnatum	
Asteraceae	Heterotheca inuloides	
Asteraceae	Heterotheca subaxillaris	
Asteraceae	Heterotheca viridis	
Asteraceae	Hidalgoa ternata	
Asteraceae	Hidalgoa uspanapa	EN
Asteraceae	Hieracium abscissum	
Asteraceae	Hieracium gronovii	VU
Asteraceae	Hieracium gypsophilum	
Asteraceae	Hieracium irasuense	
Asteraceae	Hieracium macdonaldii	EN
Asteraceae	Hieracium macdonaldii var. quiexobranum	EN
Asteraceae	Hieracium mexicanum	
Asteraceae	Hieracium pringlei	
Asteraceae	Hofmeisteria dissecta	
Asteraceae	Hofmeisteria standleyi	

Asteraceae	Hofmeisteria urenifolia		
Asteraceae	Hymenostephium cordatum		
Asteraceae	Hymenostephium tenue		
Asteraceae	Hymenoxys insignis		
Asteraceae	Hymenoxys integrifolia		
Asteraceae	Iostephane heterophylla		
Asteraceae	Iostephane madrensis		
Asteraceae	Iostephane papposa		
Asteraceae	Iostephane trilobata		
Asteraceae	Isocarpha oppositifolia		
Asteraceae	Isocarpha oppositifolia var. achyranthes		
Asteraceae	Isocoma veneta		
Asteraceae	Jacobaea gnaphalioides		
Asteraceae	Jaegeria bellidiflora		
Asteraceae	Jaegeria gracilis	CR	YES
Asteraceae	Jaegeria hirta		
Asteraceae	Jaegeria macrocephala		
Asteraceae	Jefea lantanifolia		
Asteraceae	Jefea pringlei		
Asteraceae	Jungia ferruginea	VU	
Asteraceae	Koanophyllon albicaule		
Asteraceae	Koanophyllon coulteri		
Asteraceae	Koanophyllon galeottii		
Asteraceae	Koanophyllon gracilicaule	VU	
Asteraceae	Koanophyllon longifolium		
Asteraceae	Koanophyllon monanthum		
Asteraceae	Koanophyllon palmeri		
Asteraceae	Koanophyllon pittieri		
Asteraceae	Koanophyllon ravenii	VU	
Asteraceae	Koanophyllon richardsonii		
Asteraceae	Koanophyllon solidaginoides		
Asteraceae	Koanophyllon tripartitum	EN	YES
Asteraceae	Kyrsteniopsis dibollii	VU	
Asteraceae	Kyrsteniopsis perpetiolata	VU	

Asteraceae	Kyrsteniopsis spinaciifolia
Asteraceae	Lactuca graminifolia
Asteraceae	Lactuca sativa
Asteraceae	Laennecia confusa
Asteraceae	Laennecia filaginoides
Asteraceae	Laennecia gnaphalioides
Asteraceae	Laennecia schiedeana
Asteraceae	Laennecia sophiifolia
Asteraceae	Lagascea helianthifolia
Asteraceae	Lagascea mollis
Asteraceae	Lagascea palmeri
Asteraceae	Lagascea rigida
Asteraceae	Lasianthaea aurea
Asteraceae	Lasianthaea ceanothifolia
Asteraceae	Lasianthaea crocea
Asteraceae	Lasianthaea fruticosa
Asteraceae	Lasianthaea fruticosa var. alamosana
Asteraceae	Lasianthaea fruticosa var. fasciculata
Asteraceae	Lasianthaea helianthoides
Asteraceae	Lasianthaea macrocephala
Asteraceae	Lasianthaea palmeri
Asteraceae	Lasianthaea squarrosa
Asteraceae	Launaea intybacea
Asteraceae	Leibnitzia lyrata
Asteraceae	Leiboldia serrata
Asteraceae	Lepidaploa arborescens
Asteraceae	Lepidaploa argyropappa
Asteraceae	Lepidaploa canescens
Asteraceae	Lepidaploa polypleura
Asteraceae	Lepidaploa salzmannii
Asteraceae	Lepidaploa tortuosa
Asteraceae	Lepidonia jonesii
Asteraceae	Lepidonia mexicana
Asteraceae	Lepidonia salvinae

Asteraceae	Lessingianthus rubricaulis		
Asteraceae	Leucanthemum vulgare		
Asteraceae	Liabum asclepiadeum	VU	
Asteraceae	Liabum bourgeaui		
Asteraceae	Loxothysanus pedunculatus		
Asteraceae	Loxothysanus sinuatus		
Asteraceae	Lundellianthus breedlovei	EN	YES
Asteraceae	Lundellianthus guatemalensis		
Asteraceae	Lundellianthus salvinii		
Asteraceae	Macvaughiella chiapensis		
Asteraceae	Macvaughiella mexicana		
Asteraceae	Matricaria chamomilla		
Asteraceae	Matricaria discoidea		
Asteraceae	Melampodium americanum		
Asteraceae	Melampodium dicoelocarpum		
Asteraceae	Melampodium divaricatum		
Asteraceae	Melampodium gracile		
Asteraceae	Melampodium longifolium		
Asteraceae	Melampodium longipilum		
Asteraceae	Melampodium microcephalum		
Asteraceae	Melampodium mimulifolium		
Asteraceae	Melampodium montanum		
Asteraceae	Melampodium northingtonii	EN	
Asteraceae	Melampodium nutans		
Asteraceae	Melampodium paniculatum		
Asteraceae	Melampodium perfoliatum		
Asteraceae	Melampodium repens		
Asteraceae	Melampodium sericeum		
Asteraceae	Melanthera angustifolia		
Asteraceae	Melanthera nivea		
Asteraceae	Microspermum debile		
Asteraceae	Mikania cordifolia		
Asteraceae	Mikania gonzalezii		
Asteraceae	Mikania guaco	EN	

Asteraceae	Mikania houstoniana		
Asteraceae	Mikania leiostachya		
Asteraceae	Mikania micrantha		
Asteraceae	Mikania pterocaula		
Asteraceae	Mikania pyramidata		
Asteraceae	Mikania scandens		
Asteraceae	Mikania vitifolia		
Asteraceae	Milleria quinqueflora		
Asteraceae	Montanoa atriplicifolia		
Asteraceae	Montanoa bipinnatifida		
Asteraceae	Montanoa frutescens		
Asteraceae	Montanoa grandiflora		
Asteraceae	Montanoa hexagona		
Asteraceae	Montanoa hibiscifolia		
Asteraceae	Montanoa leucantha		
Asteraceae	Montanoa leucantha subsp. arborescens		
Asteraceae	Montanoa liebmannii		
Asteraceae	Montanoa mollissima		
Asteraceae	Montanoa pteropoda		
Asteraceae	Montanoa speciosa		
Asteraceae	Montanoa standleyi	VU	
Asteraceae	Montanoa tomentosa		
Asteraceae	Montanoa tomentosa subsp. tomentosa		
Asteraceae	Montanoa tomentosa subsp. xanthiifolia		
Asteraceae	Montanoa tomentosa var. microcephala		
Asteraceae	Nahuatlea hypoleuca		
Asteraceae	Nahuatlea magna		
Asteraceae	Nahuatlea obtusata		
Asteraceae	Nahuatlea purpusii		
Asteraceae	Nelsonianthus epiphyticus	VU	
Asteraceae	Nelsonianthus tapianus	VU	
Asteraceae	Neomirandea araliifolia		
Asteraceae	Neomirandea ovandensis	VU	
Asteraceae	Neomirandea pithecobia	CR	YES

Asteraceae	Neurolaena intermedia	EN	
Asteraceae	Neurolaena lobata		
Asteraceae	Neurolaena macrocephala		
Asteraceae	Neurolaena macrophylla	EN	
Asteraceae	Neurolaena oaxacana		
Asteraceae	Neurolaena venturana	VU	
Asteraceae	Onoseris onoseroides		
Asteraceae	Orthopappus angustifolius		
Asteraceae	Osbertia stolonifera		
Asteraceae	Oteiza acuminata		
Asteraceae	Oteiza scandens	VU	
Asteraceae	Otopappus brevipes		
Asteraceae	Otopappus curviflorus		
Asteraceae	Otopappus epaleaceus		
Asteraceae	Otopappus koelzii	VU	
Asteraceae	Otopappus mexicanus		
Asteraceae	Otopappus microcephalus		
Asteraceae	Otopappus scaber		
Asteraceae	Otopappus verbesinoides		
Asteraceae	Packera bellidifolia		
Asteraceae	Packera coahuilensis		
Asteraceae	Packera sanguisorbae		
Asteraceae	Packera tampicana		
Asteraceae	Packera toluccana		
Asteraceae	Paragynoxys meridana	CR	YES
Asteraceae	Parthenium bipinnatifidum		
Asteraceae	Parthenium fruticosum		
Asteraceae	Parthenium hysterophorus		
Asteraceae	Parthenium rollinsianum	VU	
Asteraceae	Parthenium tomentosum		
Asteraceae	Pectis bonplandiana		
Asteraceae	Pectis elongata		
Asteraceae	Pectis fasciculiflora		
Asteraceae	Pectis latisquama		

Asteraceae	Pectis linearis	
Asteraceae	Pectis linifolia	
Asteraceae	Pectis multiflosculosa	
Asteraceae	Pectis prostrata	
Asteraceae	Pectis repens	
Asteraceae	Pectis saturejoides	
Asteraceae	Pectis uniaristata	
Asteraceae	Pentacalia morazensis	VU
Asteraceae	Pentacalia parasitica	
Asteraceae	Pentacalia phorodendroides	EN
Asteraceae	Pentacalia venturae	VU
Asteraceae	Perityle microglossa	
Asteraceae	Perymeniopsis ovalifolia	
Asteraceae	Perymenium berlandieri	
Asteraceae	Perymenium buphthalmoides	
Asteraceae	Perymenium chloroleucum	
Asteraceae	Perymenium collinum	EN
Asteraceae	Perymenium discolor	
Asteraceae	Perymenium ghiesbreghtii	
Asteraceae	Perymenium glandulosum	VU
Asteraceae	Perymenium grande	
Asteraceae	Perymenium grande var. nelsonii	
Asteraceae	Perymenium gymnolomoides	
Asteraceae	Perymenium klattianum	
Asteraceae	Perymenium mendezii	
Asteraceae	Perymenium mendezii var. verbesinoides	
Asteraceae	Perymenium pinetorum	VU
Asteraceae	Perymenium subsquarrosum	
Asteraceae	Peteravenia malvifolia	
Asteraceae	Peteravenia phoenicolepis	
Asteraceae	Peteravenia schultzii	
Asteraceae	Philactis liebmannii	
Asteraceae	Philactis nelsonii	
Asteraceae	Philactis zinnioides	

Asteraceae	Picradeniopsis pringlei		
Asteraceae	Pinaropappus roseus		
Asteraceae	Pinaropappus spathulatus		
Asteraceae	Piptocarpha poeppigiana		
Asteraceae	Piqueria laxiflora		
Asteraceae	Piqueria pilosa		
Asteraceae	Piqueria trinervia		
Asteraceae	Pittocaulon hintonii	EN	
Asteraceae	Pittocaulon praecox		
Asteraceae	Pittocaulon velatum		
Asteraceae	Pittocaulon velatum var. tzimolensis	VU	
Asteraceae	Pityopsis graminifolia		
Asteraceae	Pityopsis latifolia		
Asteraceae	Pityopsis tenuifolia	CR	YES
Asteraceae	Pluchea baccharis		
Asteraceae	Pluchea carolinensis		
Asteraceae	Pluchea mexicana		
Asteraceae	Pluchea odorata		
Asteraceae	Pluchea salicifolia		
Asteraceae	Podachaenium chiapanum	VU	
Asteraceae	Podachaenium eminens		
Asteraceae	Podachaenium pachyphyllum	VU	
Asteraceae	Podachaenium standleyi	EN	
Asteraceae	Porophyllum calcicola		
Asteraceae	Porophyllum filiforme		
Asteraceae	Porophyllum linaria		
Asteraceae	Porophyllum lindenii		
Asteraceae	Porophyllum punctatum		
Asteraceae	Porophyllum ruderale		
Asteraceae	Porophyllum ruderale var. macrocephalum		
Asteraceae	Porophyllum tagetoides		
Asteraceae	Porophyllum viridiflorum		
Asteraceae	Psacaliopsis macdonaldii	EN	
Asteraceae	Psacaliopsis pinetorum		

Asteraceae	Psacaliopsis purpusii		
Asteraceae	Psacalium beamanii	EN	
Asteraceae	Psacalium cirsiifolium		
Asteraceae	Psacalium hintonii	EN	
Asteraceae	Psacalium hintoniorum	EN	
Asteraceae	Psacalium megaphyllum		
Asteraceae	Psacalium nelsonii	EN	
Asteraceae	Psacalium peltatum		
Asteraceae	Psacalium peltatum var. conzattii	VU	
Asteraceae	Psacalium radulifolium		
Asteraceae	Psacalium sinuatum		
Asteraceae	Psacalium tabulare		
Asteraceae	Pseudelephantopus spicatus		
Asteraceae	Pseudoconyza viscosa		
Asteraceae	Pseudognaphalium attenuatum		
Asteraceae	Pseudognaphalium biolettii		
Asteraceae	Pseudognaphalium brachypterum		
Asteraceae	Pseudognaphalium canescens		
Asteraceae	Pseudognaphalium chartaceum		
Asteraceae	Pseudognaphalium cheiranthifolium		
Asteraceae	Pseudognaphalium conoideum		
Asteraceae	Pseudognaphalium ehrenbergianum		
Asteraceae	Pseudognaphalium elegans		
Asteraceae	Pseudognaphalium greenmanii		
Asteraceae	Pseudognaphalium inornatum		
Asteraceae	Pseudognaphalium leucocephalum		
Asteraceae	Pseudognaphalium liebmannii		
Asteraceae	Pseudognaphalium liebmannii var. monticola	VU	
Asteraceae	Pseudognaphalium microcephalum		
Asteraceae	Pseudognaphalium moelleri	CR	YES
Asteraceae	Pseudognaphalium oaxacanum	VU	
Asteraceae	Pseudognaphalium oxyphyllum		
Asteraceae	Pseudognaphalium roseum		
Asteraceae	Pseudognaphalium semiamplexicaule		

Asteraceae	Pseudognaphalium semilanatum	
Asteraceae	Pseudognaphalium stramineum	
Asteraceae	Pseudognaphalium sylvicola	
Asteraceae	Pseudognaphalium viscosum	
Asteraceae	Pseudogynoxys chenopodioides	
Asteraceae	Pseudogynoxys cummingii	
Asteraceae	Pseudogynoxys haenkei	
Asteraceae	Psilactis brevilingulata	
Asteraceae	Pyrrhopappus pauciflorus	
Asteraceae	Rensonia salvadorica	
Asteraceae	Rojasianthe superba	VU
Asteraceae	Roldana acutangula	
Asteraceae	Roldana angulifolia	
Asteraceae	Roldana anisophylla	VU
Asteraceae	Roldana aschenborniana	
Asteraceae	Roldana barba-johannis	
Asteraceae	Roldana candicans	
Asteraceae	Roldana chapalensis	
Asteraceae	Roldana cristobalensis	
Asteraceae	Roldana ehrenbergiana	
Asteraceae	Roldana eriophylla	
Asteraceae	Roldana greenmanii	EN
Asteraceae	Roldana grimesii	
Asteraceae	Roldana hartwegii var. subcymosa	
Asteraceae	Roldana heterogama	
Asteraceae	Roldana heteroidea	EN
Asteraceae	Roldana hintonii	EN
Asteraceae	Roldana jurgensenii	
Asteraceae	Roldana lanicaulis	
Asteraceae	Roldana marquesii	
Asteraceae	Roldana mexicana	
Asteraceae	Roldana oaxacana	
Asteraceae	Roldana petasioides	VU
Asteraceae	Roldana petasitis	

Asteraceae	Roldana platanifolia	
Asteraceae	Roldana sartorii	
Asteraceae	Roldana schaffneri	
Asteraceae	Roldana tlacotepecana	EN
Asteraceae	Rumfordia floribunda	
Asteraceae	Rumfordia guatemalensis	
Asteraceae	Rumfordia penninervis	EN
Asteraceae	Sabazia glandulosa	
Asteraceae	Sabazia humilis	
Asteraceae	Sabazia macdonaldii	
Asteraceae	Sabazia multiradiata	
Asteraceae	Sabazia sarmentosa	
Asteraceae	Salmea orthocephala	
Asteraceae	Salmea scandens	
Asteraceae	Sanvitalia angustifolia	
Asteraceae	Sanvitalia fruticosa	
Asteraceae	Sanvitalia ocymoides	
Asteraceae	Sanvitalia procumbens	
Asteraceae	Sartwellia mexicana	
Asteraceae	Schistocarpha bicolor	
Asteraceae	Schistocarpha eupatorioides	
Asteraceae	Schistocarpha liebmannii	
Asteraceae	Schistocarpha longiligula	
Asteraceae	Schistocarpha matudae	EN
Asteraceae	Schistocarpha pedicellata	
Asteraceae	Schistocarpha platyphylla	
Asteraceae	Schkuhria pinnata	
Asteraceae	Sclerocarpus divaricatus	
Asteraceae	Sclerocarpus phyllocephalus	
Asteraceae	Sclerocarpus uniserialis	
Asteraceae	Sclerocarpus uniserialis var. frutescens	
Asteraceae	Sclerocarpus uniserialis var. rubridiscus	
Asteraceae	Selloa macdonaldii	EN
Asteraceae	Senecio bracteatus	

Asteraceae	Senecio callosus	
Asteraceae	Senecio cinerarioides	
Asteraceae	Senecio doratophyllus	
Asteraceae	Senecio flaccidus	
Asteraceae	Senecio godmanii	VU
Asteraceae	Senecio goldmanii	VU
Asteraceae	Senecio inaequidens	
Asteraceae	Senecio orizabensis	
Asteraceae	Senecio ozolotepecanus	EN
Asteraceae	Senecio picridis	
Asteraceae	Senecio polypodioides	
Asteraceae	Senecio powellii	VU
Asteraceae	Senecio prenanthoides	VU
Asteraceae	Senecio procumbens	
Asteraceae	Senecio richardsonii	
Asteraceae	Senecio roseus	
Asteraceae	Senecio stoechadiformis	
Asteraceae	Senecio subauriculatus	EN
Asteraceae	Senecio vernalis	
Asteraceae	Senecio warszewiczii	VU
Asteraceae	Sidneya pinnatilobata	
Asteraceae	Sigesbeckia agrestis	
Asteraceae	Sigesbeckia jorullensis	
Asteraceae	Sigesbeckia repens	
Asteraceae	Simsia amplexicaulis	
Asteraceae	Simsia annectens var. grayi	VU
Asteraceae	Simsia cronquistii	EN
Asteraceae	Simsia eurylepis	
Asteraceae	Simsia foetida	
Asteraceae	Simsia foetida var. megacephala	
Asteraceae	Simsia ovata	
Asteraceae	Simsia sanguinea	
Asteraceae	Simsia villasenorii	VU
Asteraceae	Sinclairia andrieuxii	

Asteraceae	Sinclairia andromachioides			
Asteraceae	Sinclairia broomeae		VU	
Asteraceae	Sinclairia caducifolia			
Asteraceae	Sinclairia deamii			
Asteraceae	Sinclairia deppeana			
Asteraceae	Sinclairia discolor			
Asteraceae	Sinclairia glabra			
Asteraceae	Sinclairia glabra var. hypoleuca			
Asteraceae	Sinclairia glabra var. minor		VU	
Asteraceae	Sinclairia klattii			
Asteraceae	Sinclairia liebmannii			
Asteraceae	Sinclairia polyantha			
Asteraceae	Sinclairia sericolepis			
Asteraceae	Sinclairia vagans		VU	
Asteraceae	Smallanthus maculatus			
Asteraceae	Smallanthus oaxacanus			
Asteraceae	Smallanthus uvedalia			
Asteraceae	Solidago stricta			
Asteraceae	Sonchus asper			
Asteraceae	Sonchus oleraceus			
Asteraceae	Sphagneticola trilobata			
Asteraceae	Squamopappus skutchii			
Asteraceae	Steiractinia klattii		EN	
Asteraceae	Stenocephalum jucundum			
Asteraceae	Stevia alatipes			
Asteraceae	Stevia berlandieri			
Asteraceae	Stevia caracasana			
Asteraceae	Stevia chiapensis		EN	
Asteraceae	Stevia connata			
Asteraceae	Stevia crassifolia		CR	YES
Asteraceae	Stevia cruzii	NT	VU	
Asteraceae	Stevia ecatepecana		EN	
Asteraceae	Stevia elatior			
Asteraceae	Stevia eupatoria			

Asteraceae	Stevia incognita		
Asteraceae	Stevia isomeca		
Asteraceae	Stevia jorullensis		
Asteraceae	Stevia latifolia		
Asteraceae	Stevia liebmannii		
Asteraceae	Stevia lita		
Asteraceae	Stevia lucida		
Asteraceae	Stevia micrantha		
Asteraceae	Stevia microchaeta		
Asteraceae	Stevia monardifolia		
Asteraceae	Stevia origanoides		
Asteraceae	Stevia orizabensis	VU	
Asteraceae	Stevia ovata		
Asteraceae	Stevia perfoliata	VU	
Asteraceae	Stevia pilosa		
Asteraceae	Stevia polycephala		
Asteraceae	Stevia porphyrea		
Asteraceae	Stevia pratheri	EN	
Asteraceae	Stevia quiexobra	EN	
Asteraceae	Stevia revoluta		
Asteraceae	Stevia salicifolia		
Asteraceae	Stevia salicifolia var. collodes		
Asteraceae	Stevia seemannii		
Asteraceae	Stevia seleriana		
Asteraceae	Stevia serrata		
Asteraceae	Stevia suaveolens		
Asteraceae	Stevia subpubescens		
Asteraceae	Stevia tephra		
Asteraceae	Stevia tephrophylla		
Asteraceae	Stevia tomentosa		
Asteraceae	Stevia trifida		
Asteraceae	Stevia triflora		
Asteraceae	Stevia viscida		
Asteraceae	Steyermarkina pyrifolia	CR	YES

Asteraceae	Stramentopappus congestiflorus	CR	YES
Asteraceae	Stramentopappus pooleae		
Asteraceae	Struchium sparganophorum	VU	
Asteraceae	Symphyotrichum bullatum		
Asteraceae	Symphyotrichum ericoides		
Asteraceae	Symphyotrichum moranense		
Asteraceae	Symphyotrichum novi-belgii		
Asteraceae	Symphyotrichum potosinum		
Asteraceae	Symphyotrichum schaffneri		
Asteraceae	Symphyotrichum subulatum		
Asteraceae	Symphyotrichum subulatum var. ligulatum		
Asteraceae	Symphyotrichum subulatum var. parviflorum		
Asteraceae	Symphyotrichum trilineatum		
Asteraceae	Synedrella nodiflora		
Asteraceae	Tagetes coronopifolia		
Asteraceae	Tagetes erecta		
Asteraceae	Tagetes filifolia		
Asteraceae	Tagetes filiformis	CR	YES
Asteraceae	Tagetes foetidissima		
Asteraceae	Tagetes linifolia		
Asteraceae	Tagetes lucida		
Asteraceae	Tagetes lunulata		
Asteraceae	Tagetes micrantha		
Asteraceae	Tagetes moorei	VU	
Asteraceae	Tagetes nelsonii		
Asteraceae	Tagetes parryi		
Asteraceae	Tagetes subulata		
Asteraceae	Tagetes tenuifolia		
Asteraceae	Tanacetum parthenium		
Asteraceae	Taraxacum campylodes	EN	
Asteraceae	Taraxacum officinale		
Asteraceae	Taraxacum sect. Taraxacum		
Asteraceae	Tehuana calzadae	VU	
Asteraceae	Telanthophora andrieuxii		

Asteraceae	Telanthophora cobanensis		
Asteraceae	Telanthophora grandifolia		
Asteraceae	Telanthophora liebmannii		
Asteraceae	Telanthophora uspantanensis		
Asteraceae	Tetrachyron brandegeei		
Asteraceae	Tetrachyron manicatum		
Asteraceae	Tetrachyron orizabaensis		
Asteraceae	Tetrachyron websteri		
Asteraceae	Thymophylla aurantiaca		
Asteraceae	Thymophylla setifolia		
Asteraceae	Thymophylla tenuifolia		
Asteraceae	Tithonia brachypappa		
Asteraceae	Tithonia diversifolia		
Asteraceae	Tithonia hondurensis	CR	YES
Asteraceae	Tithonia longiradiata		
Asteraceae	Tithonia rotundifolia		
Asteraceae	Tithonia tubaeformis		
Asteraceae	Tridax brachylepis		
Asteraceae	Tridax coronopifolia		
Asteraceae	Tridax mexicana		
Asteraceae	Tridax palmeri		
Asteraceae	Tridax platyphylla		
Asteraceae	Tridax procumbens		
Asteraceae	Tridax purpusii		
Asteraceae	Tridax tenuifolia		
Asteraceae	Trigonospermum adenostemmoides	VU	
Asteraceae	Trigonospermum annuum		
Asteraceae	Trigonospermum melampodioides		
Asteraceae	Trixis anomala	VU	
Asteraceae	Trixis chiapensis		
Asteraceae	Trixis inula		
Asteraceae	Trixis mexicana		
Asteraceae	Trixis mexicana var. macradenia	VU	
Asteraceae	Trixis nelsonii		

Asteraceae	Trixis pringlei		
Asteraceae	Trixis pringlei var. oligantha		
Asteraceae	Trixis silvatica		
Asteraceae	Tuberculocarpus ruber	CR	YES
Asteraceae	Urolepis hecatantha	CR	
Asteraceae	Verbesina abietifolia	EN	
Asteraceae	Verbesina abscondita		
Asteraceae	Verbesina acuminata	VU	
Asteraceae	Verbesina agricolarum	VU	
Asteraceae	Verbesina angustifolia		
Asteraceae	Verbesina apleura		
Asteraceae	Verbesina auriculata		
Asteraceae	Verbesina breedlovei		
Asteraceae	Verbesina chiapensis		
Asteraceae	Verbesina coulteri	VU	
Asteraceae	Verbesina crocata		
Asteraceae	Verbesina cronquistii	VU	
Asteraceae	Verbesina diversifolia	EN	
Asteraceae	Verbesina fastigiata		
Asteraceae	Verbesina furfuracea	VU	
Asteraceae	Verbesina gigantea		
Asteraceae	Verbesina gracilipes		
Asteraceae	Verbesina hypargyrea		
Asteraceae	Verbesina hypoglauca		
Asteraceae	Verbesina hypomalaca		
Asteraceae	Verbesina lanata		
Asteraceae	Verbesina liebmannii		
Asteraceae	Verbesina longipes		
Asteraceae	Verbesina macdonaldii	VU	
Asteraceae	Verbesina macvaughii		
Asteraceae	Verbesina miahuatlana	VU	
Asteraceae	Verbesina microptera		
Asteraceae	Verbesina myriocephala		
Asteraceae	Verbesina neriifolia		

Asteraceae	Verbesina oaxacana			
Asteraceae	Verbesina oligantha			
Asteraceae	Verbesina oncophora			
Asteraceae	Verbesina ovata			
Asteraceae	Verbesina ovatifolia			
Asteraceae	Verbesina pallens			
Asteraceae	Verbesina persicifolia			
Asteraceae	Verbesina perymenioides			
Asteraceae	Verbesina petzalensis		EN	
Asteraceae	Verbesina pleistocephala			
Asteraceae	Verbesina richardsonii		CR	YES
Asteraceae	Verbesina robinsonii			
Asteraceae	Verbesina scabrida			
Asteraceae	Verbesina sericea			
Asteraceae	Verbesina sororia		VU	
Asteraceae	Verbesina steinmannii		CR	
Asteraceae	Verbesina tetraptera			
Asteraceae	Verbesina trilobata			
Asteraceae	Verbesina turbacensis			
Asteraceae	Verbesina virgata			
Asteraceae	Vernonanthura cordata			
Asteraceae	Vernonanthura liatroides			
Asteraceae	Vernonanthura oaxacana			
Asteraceae	Vernonanthura patens			
Asteraceae	Vernonia alamanii			
Asteraceae	Vernonia greggii			
Asteraceae	Vernonia greggii subsp. ervendbergii			
Asteraceae	Vernonia karvinskiana			
Asteraceae	Vernonia karvinskiana subsp. inuloides			
Asteraceae	Viguiera dentata			
Asteraceae	Villanova achilleoides			
Asteraceae	Villasenoria orcuttii	EN		
Asteraceae	Wamalchitamia dionysi		EN	
Asteraceae	Wedelia acapulcensis			

Asteraceae	Wedelia acapulcensis var. hispida	
Asteraceae	Wedelia iners	EN
Asteraceae	Wedelia purpurea	
Asteraceae	Wedelia strigosa	
Asteraceae	Werneria nubigena	VU
Asteraceae	Xanthisma gymnocephalum	
Asteraceae	Xanthium strumarium	
Asteraceae	Youngia japonica	
Asteraceae	Zaluzania augusta	
Asteraceae	Zaluzania parthenioides	
Asteraceae	Zaluzania subcordata	
Asteraceae	Zaluzania triloba	
Asteraceae	Zemisia thomasii	
Asteraceae	Zexmenia serrata	
Asteraceae	Zinnia acerosa	
Asteraceae	Zinnia americana	
Asteraceae	Zinnia elegans	
Asteraceae	Zinnia flavicoma	
Asteraceae	Zinnia haageana	
Asteraceae	Zinnia juniperifolia	
Asteraceae	Zinnia maritima	
Asteraceae	Zinnia peruviana	
Asteraceae	Zinnia purpusii	
Asteraceae	Zinnia zinnioides	
Balanophoraceae	Helosis cayennensis	
Balanophoraceae	Helosis cayennensis var. mexicana	
Balsaminaceae	Impatiens balfourii	
Balsaminaceae	Impatiens balsamina	
Balsaminaceae	Impatiens hawkeri	VU
Balsaminaceae	Impatiens mexicana	VU
Balsaminaceae	Impatiens sodenii	
Balsaminaceae	Impatiens walleriana	
Basellaceae	Anredera cordifolia	
Basellaceae	Anredera ramosa	

Basellaceae	Anredera vesicaria		
Bataceae	Batis maritima		
Begoniaceae	Begonia acutiloba	EN	
Begoniaceae	Begonia alice-clarkae	CR	YES
Begoniaceae	Begonia almedana	EN	
Begoniaceae	Begonia argentea	EN	
Begoniaceae	Begonia balmisiana		
Begoniaceae	Begonia barkeri		
Begoniaceae	Begonia bettinae	EN	
Begoniaceae	Begonia biserrata		
Begoniaceae	Begonia boissieri		
Begoniaceae	Begonia breedlovei	EN	
Begoniaceae	Begonia calderonii		
Begoniaceae	Begonia calzadae	CR	YES
Begoniaceae	Begonia carrieae	CR	YES
Begoniaceae	Begonia chivatoa		
Begoniaceae	Begonia convallariodora		
Begoniaceae	Begonia corzoensis	EN	
Begoniaceae	Begonia crassicaulis	VU	
Begoniaceae	Begonia cucullata		
Begoniaceae	Begonia erecta	CR	YES
Begoniaceae	Begonia extranea		
Begoniaceae	Begonia faustinoi	EN	
Begoniaceae	Begonia fischeri		
Begoniaceae	Begonia fusca		
Begoniaceae	Begonia glabra		
Begoniaceae	Begonia glandulosa		
Begoniaceae	Begonia gracilis		
Begoniaceae	Begonia heracleifolia		
Begoniaceae	Begonia hispidivillosa	EN	
Begoniaceae	Begonia hydrocotylifolia	VU	
Begoniaceae	Begonia imperialis		
Begoniaceae	Begonia incarnata		
Begoniaceae	Begonia kenworthyae	EN	

Begoniaceae	Begonia ludicra		
Begoniaceae	Begonia lyniceorum	EN	
Begoniaceae	Begonia maculata		
Begoniaceae	Begonia manicata		
Begoniaceae	Begonia mazae	VU	
Begoniaceae	Begonia monophylla		
Begoniaceae	Begonia motozintlensis	VU	
Begoniaceae	Begonia multistaminea		
Begoniaceae	Begonia nelumbiifolia		
Begoniaceae	Begonia oaxacana		
Begoniaceae	Begonia peltata		
Begoniaceae	Begonia philodendroides		
Begoniaceae	Begonia pinetorum		
Begoniaceae	Begonia plantaginea	VU	
Begoniaceae	Begonia plebeja		
Begoniaceae	Begonia pustulata		
Begoniaceae	Begonia rhodochlamys	VU	
Begoniaceae	Begonia sandtii	VU	
Begoniaceae	Begonia sartorii		
Begoniaceae	Begonia sericoneura		
Begoniaceae	Begonia strigillosa		
Begoniaceae	Begonia thiemei		
Begoniaceae	Begonia trichosepala	CR	YES
Begoniaceae	Begonia violifolia	VU	
Begoniaceae	Begonia wallichiana		
Begoniaceae	Begonia xilitlensis	VU	
Berberidaceae	Berberis alpina		
Berberidaceae	Berberis berriozabalensis	EN	
Berberidaceae	Berberis chochoco		
Berberidaceae	Berberis ehrenbergii	VU	
Berberidaceae	Berberis gracilis		
Berberidaceae	Berberis hartwegii		
Berberidaceae	Berberis hemsleyi		
Berberidaceae	Berberis ilicina		

Berberidaceae	Berberis johnstonii			VU	
Berberidaceae	Berberis lanceolata				
Berberidaceae	Berberis longipes				
Berberidaceae	Berberis moranensis				
Berberidaceae	Berberis paniculata			CR	YES
Berberidaceae	Berberis quinquefolia				
Berberidaceae	Berberis tenuifolia				
Berberidaceae	Berberis trifoliolata				
Betulaceae	Alnus acuminata				
Betulaceae	Alnus acuminata subsp. acuminata				
Betulaceae	Alnus acuminata subsp. arguta				
Betulaceae	Alnus acuminata subsp. glabrata				
Betulaceae	Alnus jorullensis				
Betulaceae	Alnus jorullensis subsp. jorullensis				
Betulaceae	Alnus jorullensis subsp. lutea				
Betulaceae	Carpinus caroliniana	VU			
Betulaceae	Carpinus caroliniana subsp. caroliniana			CR	YES
Betulaceae	Carpinus tropicalis				
Betulaceae	Carpinus tropicalis subsp. mexicana				
Betulaceae	Ostrya virginiana	NT			
Betulaceae	Ostrya virginiana subsp. guatemalensis				
Betulaceae	Ostrya virginiana subsp. virginiana				
Bignoniaceae	Adenocalymma apurense				
Bignoniaceae	Adenocalymma calderonii				
Bignoniaceae	Adenocalymma inundatum				
Bignoniaceae	Amphilophium crucigerum				
Bignoniaceae	Amphilophium laxiflorum				
Bignoniaceae	Amphilophium paniculatum				
Bignoniaceae	Amphitecna apiculata				
Bignoniaceae	Amphitecna breedlovei				
Bignoniaceae	Amphitecna latifolia				
Bignoniaceae	Amphitecna loreae		CR	EN	
Bignoniaceae	Amphitecna macrophylla				
Bignoniaceae	Amphitecna montana				

Bignoniaceae	Amphitecna regalis	EN	
Bignoniaceae	Amphitecna silvicola		VU
Bignoniaceae	Amphitecna steyermarkii		VU
Bignoniaceae	Amphitecna tuxtlensis	EN	
Bignoniaceae	Anemopaegma chrysanthum		
Bignoniaceae	Anemopaegma puberulum		
Bignoniaceae	Astianthus viminalis		
Bignoniaceae	Bignonia aequinoctialis		
Bignoniaceae	Bignonia binata		
Bignoniaceae	Bignonia diversifolia		
Bignoniaceae	Bignonia hyacinthina		
Bignoniaceae	Bignonia neoheterophylla		
Bignoniaceae	Bignonia noterophila		
Bignoniaceae	Bignonia potosina		
Bignoniaceae	Callichlamys latifolia		
Bignoniaceae	Campsis radicans		VU
Bignoniaceae	Crescentia alata		
Bignoniaceae	Crescentia cujete		
Bignoniaceae	Cuspidaria inaequalis		VU
Bignoniaceae	Dolichandra quadrivalvis		
Bignoniaceae	Dolichandra uncata		
Bignoniaceae	Dolichandra unguis-cati		
Bignoniaceae	Fridericia candicans		VU
Bignoniaceae	Fridericia chica		
Bignoniaceae	Fridericia costaricensis		
Bignoniaceae	Fridericia dichotoma		
Bignoniaceae	Fridericia floribunda		
Bignoniaceae	Fridericia florida		
Bignoniaceae	Fridericia mollissima		
Bignoniaceae	Fridericia patellifera		
Bignoniaceae	Fridericia podopogon		
Bignoniaceae	Fridericia pubescens		
Bignoniaceae	Fridericia schumanniana		
Bignoniaceae	Godmania aesculifolia		

Bignoniaceae	Handroanthus chrysanthus		VU	
Bignoniaceae	Handroanthus guayacan			
Bignoniaceae	Handroanthus impetiginosus	VU		
Bignoniaceae	Jacaranda acutifolia			
Bignoniaceae	Jacaranda mimosifolia		VU	
Bignoniaceae	Lundia puberula			EN
Bignoniaceae	Mansoa alliacea			VU
Bignoniaceae	Mansoa hymenaea			
Bignoniaceae	Mansoa verrucifera			
Bignoniaceae	Martinella obovata			EN
Bignoniaceae	Parmentiera aculeata			
Bignoniaceae	Podranea ricasoliana			
Bignoniaceae	Pyrostegia venusta			
Bignoniaceae	Roseodendron donnell-smithii			
Bignoniaceae	Spathodea campanulata			
Bignoniaceae	Stizophyllum riparium			
Bignoniaceae	Tabebuia heterophylla			
Bignoniaceae	Tabebuia rosea			
Bignoniaceae	Tanaecium caudiculatum			
Bignoniaceae	Tanaecium pyramidatum			
Bignoniaceae	Tanaecium tetragonolobum			
Bignoniaceae	Tecoma stans			
Bignoniaceae	Tecoma stans var. stans			
Bignoniaceae	Tecoma stans var. velutina			
Bignoniaceae	Tecomaria capensis			
Bignoniaceae	Tourrettia lappacea			VU
Bignoniaceae	Tynanthus guatemalensis			
Bignoniaceae	Xylophragma seemannianum			
Bixaceae	Bixa orellana			
Bixaceae	Cochlospermum palmatifidum			
Bixaceae	Cochlospermum vitifolium			
Bixaceae	Cochlospermum wrightii			
Boraginaceae	Antiphytum caespitosum			
Boraginaceae	Antiphytum heliotropioides			

Boraginaceae	Antiphytum paniculatum			
Boraginaceae	Antiphytum parryi			
Boraginaceae	Borago officinalis			
Boraginaceae	Bourreria andrieuxii			
Boraginaceae	Bourreria hintonii			
Boraginaceae	Bourreria huanita	VU		
Boraginaceae	Bourreria mollis			
Boraginaceae	Bourreria motaguensis		CR	
Boraginaceae	Bourreria pulchra			
Boraginaceae	Bourreria spathulata			
Boraginaceae	Bourreria superba	EN		
Boraginaceae	Cordia alliodora			
Boraginaceae	Cordia bicolor			
Boraginaceae	Cordia boissieri			
Boraginaceae	Cordia colimensis	CR	EN	
Boraginaceae	Cordia collococca			
Boraginaceae	Cordia cordiformis	VU	EN	
Boraginaceae	Cordia dentata			
Boraginaceae	Cordia diversifolia			
Boraginaceae	Cordia dodecandra			
Boraginaceae	Cordia elaeagnoides			
Boraginaceae	Cordia eriostigma			
Boraginaceae	Cordia gerascanthus			
Boraginaceae	Cordia guerkeana	VU		
Boraginaceae	Cordia megalantha	VU		
Boraginaceae	Cordia naidophila		CR	YES
Boraginaceae	Cordia prunifolia			
Boraginaceae	Cordia salvadorensis			
Boraginaceae	Cordia seleriana			
Boraginaceae	Cordia stellifera			
Boraginaceae	Cordia stenoclada			
Boraginaceae	Cryptantha maritima			
Boraginaceae	Cynoglossum amabile			
Boraginaceae	Ehretia anacua			

Boraginaceae	Ehretia latifolia	
Boraginaceae	Ehretia tinifolia	
Boraginaceae	Euploca confertifolia	
Boraginaceae	Euploca fallax	
Boraginaceae	Euploca filiformis	
Boraginaceae	Euploca foliosissima	
Boraginaceae	Euploca fruticosa	
Boraginaceae	Euploca humilis	
Boraginaceae	Euploca limbata	
Boraginaceae	Euploca pringlei	
Boraginaceae	Euploca procumbens	
Boraginaceae	Euploca queretaroana	
Boraginaceae	Euploca strigosa	
Boraginaceae	Hackelia hintoniorum	VU
Boraginaceae	Hackelia mexicana	
Boraginaceae	Heliotropium angiospermum	
Boraginaceae	Heliotropium curassavicum	
Boraginaceae	Heliotropium indicum	
Boraginaceae	Heliotropium macrostachyum	
Boraginaceae	Heliotropium petiolare	
Boraginaceae	Heliotropium pileiforme	EN
Boraginaceae	Heliotropium rufipilum	
Boraginaceae	Heliotropium sarmentosum	
Boraginaceae	Heliotropium transalpinum	VU
Boraginaceae	Heliotropium verdcourtii	
Boraginaceae	Johnstonella albida	
Boraginaceae	Johnstonella angustifolia	
Boraginaceae	Lennoa madreporoides	
Boraginaceae	Lepidocordia williamsii	
Boraginaceae	Lithospermum album	EN
Boraginaceae	Lithospermum calcicola	
Boraginaceae	Lithospermum discolor	
Boraginaceae	Lithospermum distichum	
Boraginaceae	Lithospermum flavum	

Boraginaceae	Lithospermum guatemalense	VU	
Boraginaceae	Lithospermum revolutum		
Boraginaceae	Lithospermum strictum		
Boraginaceae	Lithospermum strigosum	EN	
Boraginaceae	Lithospermum trinervium		
Boraginaceae	Lithospermum unicum		
Boraginaceae	Mimophytum cardiophyllum		
Boraginaceae	Mimophytum omphalodoides	VU	
Boraginaceae	Mimophytum richardsonii	EN	
Boraginaceae	Myriopus maculatus		
Boraginaceae	Myriopus volubilis		
Boraginaceae	Nama biflora		
Boraginaceae	Nama dichotoma		
Boraginaceae	Nama jamaicensis		
Boraginaceae	Nama marshii	EN	
Boraginaceae	Nama palmeri		
Boraginaceae	Nama sericea		
Boraginaceae	Nama stenocarpa		
Boraginaceae	Nama undulata		
Boraginaceae	Phacelia coulteri	VU	
Boraginaceae	Phacelia platycarpa		
Boraginaceae	Phacelia zaragozana		
Boraginaceae	Rochefortia lundellii		
Boraginaceae	Rochefortia spinosa		
Boraginaceae	Tiquilia canescens		
Boraginaceae	Tournefortia albifolia	CR	YES
Boraginaceae	Tournefortia bicolor		
Boraginaceae	Tournefortia calycina		
Boraginaceae	Tournefortia capitata		
Boraginaceae	Tournefortia densiflora		
Boraginaceae	Tournefortia elongata		
Boraginaceae	Tournefortia fruticosa	CR	YES
Boraginaceae	Tournefortia glabra		
Boraginaceae	Tournefortia hartwegiana		

Boraginaceae	Tournefortia longiloba	CR	YES
Boraginaceae	Tournefortia mutabilis		
Boraginaceae	Tournefortia trichocalycina		
Boraginaceae	Tournefortia umbellata		
Boraginaceae	Varronia ambigua		
Boraginaceae	Varronia curassavica		
Boraginaceae	Varronia cylindrostachya		
Boraginaceae	Varronia foliosa		
Boraginaceae	Varronia globosa		
Boraginaceae	Varronia guanacastensis		
Boraginaceae	Varronia inermis		
Boraginaceae	Varronia macrocephala		
Boraginaceae	Varronia oaxacana		
Boraginaceae	Varronia podocephala		
Boraginaceae	Varronia polycephala	EN	
Boraginaceae	Varronia spinescens		
Boraginaceae	Wigandia urens		
Brassicaceae	Brassica cretica	EN	
Brassicaceae	Brassica juncea		
Brassicaceae	Brassica napus		
Brassicaceae	Brassica nigra		
Brassicaceae	Brassica oleracea		
Brassicaceae	Brassica rapa		
Brassicaceae	Capsella bursa-pastoris		
Brassicaceae	Cardamine auriculata		
Brassicaceae	Cardamine bonariensis		
Brassicaceae	Cardamine flaccida		
Brassicaceae	Cardamine flexuosa	VU	
Brassicaceae	Cardamine fulcrata		
Brassicaceae	Cardamine hirsuta		
Brassicaceae	Cardamine mexicana		
Brassicaceae	Cardamine obliqua		
Brassicaceae	Descurainia hartwegiana	EN	
Brassicaceae	Descurainia impatiens		

Brassicaceae	Descurainia streptocarpa			
Brassicaceae	Draba jorullensis			
Brassicaceae	Draba nivicola	VU		
Brassicaceae	Eruca vesicaria			
Brassicaceae	Erysimum capitatum			
Brassicaceae	Exhalimolobos berlandieri			
Brassicaceae	Hirschfeldia incana			
Brassicaceae	Lepidium armoracium		EN	
Brassicaceae	Lepidium costaricense			
Brassicaceae	Lepidium oblongum			
Brassicaceae	Lepidium schaffneri			
Brassicaceae	Lepidium virginicum			
Brassicaceae	Lepidium virginicum subsp. menziesii			
Brassicaceae	Lobularia maritima			
Brassicaceae	Matthiola incana			
Brassicaceae	Nasturtium gambelii		VU	
Brassicaceae	Nasturtium officinale			
Brassicaceae	Nerisyrenia gracilis			
Brassicaceae	Ornithocarpa torulosa		VU	
Brassicaceae	Pennellia patens			
Brassicaceae	Physaria pueblensis		VU	
Brassicaceae	Physaria purpurea			
Brassicaceae	Physaria rosei			
Brassicaceae	Physaria rosei subsp. perotensis		CR	YES
Brassicaceae	Raphanus raphanistrum			
Brassicaceae	Raphanus raphanistrum subsp. sativus			
Brassicaceae	Rapistrum rugosum			
Brassicaceae	Romanschulzia arabiformis			
Brassicaceae	Romanschulzia mexicana		EN	
Brassicaceae	Rorippa mexicana			
Brassicaceae	Rorippa teres			
Brassicaceae	Sisymbrium irio			
Bromeliaceae	Aechmea bracteata			
Bromeliaceae	Aechmea lueddemanniana			

Bromeliaceae	Aechmea magdalenae			
Bromeliaceae	Aechmea mexicana			
Bromeliaceae	Aechmea nudicaulis			
Bromeliaceae	Aechmea nudicaulis var. nudicaulis		EN	
Bromeliaceae	Aechmea tillandsioides			
Bromeliaceae	Ananas comosus			
Bromeliaceae	Ananas comosus var. comosus		EN	
Bromeliaceae	Androlepis skinneri			
Bromeliaceae	Billbergia pallidiflora			
Bromeliaceae	Billbergia viridiflora		VU	
Bromeliaceae	Bromelia karatas			
Bromeliaceae	Bromelia palmeri			
Bromeliaceae	Bromelia pinguin			
Bromeliaceae	Catopsis berteroniana	NT		
Bromeliaceae	Catopsis compacta			
Bromeliaceae	Catopsis floribunda			
Bromeliaceae	Catopsis juncifolia		VU	
Bromeliaceae	Catopsis montana		EN	
Bromeliaceae	Catopsis morreniana			
Bromeliaceae	Catopsis nitida			
Bromeliaceae	Catopsis nutans			
Bromeliaceae	Catopsis occulta		VU	
Bromeliaceae	Catopsis oerstediana		VU	
Bromeliaceae	Catopsis paniculata			
Bromeliaceae	Catopsis sessiliflora			
Bromeliaceae	Catopsis subulata			
Bromeliaceae	Catopsis wangerinii			
Bromeliaceae	Fosterella micrantha			
Bromeliaceae	Greigia oaxacana			
Bromeliaceae	Greigia steyermarkii		EN	
Bromeliaceae	Guzmania lingulata		VU	
Bromeliaceae	Guzmania lingulata var. minor		EN	
Bromeliaceae	Guzmania nicaraguensis			
Bromeliaceae	Guzmania strobilantha		CR	YES

Bromeliaceae	Hechtia bracteata	VU	
Bromeliaceae	Hechtia caudata	VU	
Bromeliaceae	Hechtia confusa		
Bromeliaceae	Hechtia fragilis	VU	
Bromeliaceae	Hechtia glomerata		
Bromeliaceae	Hechtia liebmannii		
Bromeliaceae	Hechtia lundelliorum		
Bromeliaceae	Hechtia mexicana	EN	
Bromeliaceae	Hechtia perotensis		
Bromeliaceae	Hechtia podantha		
Bromeliaceae	Hechtia pringlei		
Bromeliaceae	Hechtia rosea		
Bromeliaceae	Hechtia schottii		
Bromeliaceae	Hechtia tillandsioides	VU	
Bromeliaceae	Neoregelia concentrica	CR	YES
Bromeliaceae	Neoregelia sanguinea	CR	YES
Bromeliaceae	Pitcairnia amblyosperma		
Bromeliaceae	Pitcairnia atrorubens	EN	
Bromeliaceae	Pitcairnia breedlovei		
Bromeliaceae	Pitcairnia calderonii	VU	
Bromeliaceae	Pitcairnia chiapensis		
Bromeliaceae	Pitcairnia foliacea	EN	
Bromeliaceae	Pitcairnia heterophylla		
Bromeliaceae	Pitcairnia imbricata		
Bromeliaceae	Pitcairnia mirandae	EN	YES
Bromeliaceae	Pitcairnia modesta	EN	
Bromeliaceae	Pitcairnia puberula	EN	
Bromeliaceae	Pitcairnia punicea		
Bromeliaceae	Pitcairnia recurvata		
Bromeliaceae	Pitcairnia ringens		
Bromeliaceae	Pitcairnia secundiflora	VU	
Bromeliaceae	Pitcairnia tuerckheimii	EN	
Bromeliaceae	Pitcairnia undulata		
Bromeliaceae	Pitcairnia vallisoletana	VU	

Bromeliaceae	Pitcairnia wendlandii		VU
Bromeliaceae	Pitcairnia xanthocalyx		VU
Bromeliaceae	Pseudalcantarea grandis		
Bromeliaceae	Pseudalcantarea macropetala		EN
Bromeliaceae	Pseudalcantarea viridiflora		
Bromeliaceae	Racinaea ghiesbreghtii		
Bromeliaceae	Racinaea rothschuhiana		
Bromeliaceae	Tillandsia achyrostachys		
Bromeliaceae	Tillandsia argentea		
Bromeliaceae	Tillandsia atroviridipetala		
Bromeliaceae	Tillandsia baileyi		
Bromeliaceae	Tillandsia balbisiana		
Bromeliaceae	Tillandsia bartramii		
Bromeliaceae	Tillandsia belloensis		
Bromeliaceae	Tillandsia bourgaei		
Bromeliaceae	Tillandsia brachycaulos		
Bromeliaceae	Tillandsia bulbosa		
Bromeliaceae	Tillandsia butzii		
Bromeliaceae	Tillandsia calothyrsus		
Bromeliaceae	Tillandsia capitata		
Bromeliaceae	Tillandsia caput-medusae		
Bromeliaceae	Tillandsia carlsoniae		
Bromeliaceae	Tillandsia chaetophylla		
Bromeliaceae	Tillandsia chiapensis	VU	VU
Bromeliaceae	Tillandsia chlorophylla		
Bromeliaceae	Tillandsia comitanensis		VU
Bromeliaceae	Tillandsia compressa		
Bromeliaceae	Tillandsia concolor	VU	
Bromeliaceae	Tillandsia cucaensis		
Bromeliaceae	Tillandsia dasyliriifolia		
Bromeliaceae	Tillandsia deppeana		
Bromeliaceae	Tillandsia dugesii		
Bromeliaceae	Tillandsia eistetteri		
Bromeliaceae	Tillandsia eizii		

Bromeliaceae	Tillandsia elusiva		VU	
Bromeliaceae	Tillandsia erubescens			
Bromeliaceae	Tillandsia erubescens var. arroyoensis			
Bromeliaceae	Tillandsia erubescens var. erubescens			
Bromeliaceae	Tillandsia excelsa			
Bromeliaceae	Tillandsia fasciculata			
Bromeliaceae	Tillandsia fasciculata var. fasciculata			
Bromeliaceae	Tillandsia festucoides	NT		
Bromeliaceae	Tillandsia filifolia			
Bromeliaceae	Tillandsia flabellata			
Bromeliaceae	Tillandsia flavobracteata			
Bromeliaceae	Tillandsia guatemalensis			
Bromeliaceae	Tillandsia gymnobotrya			
Bromeliaceae	Tillandsia heliconioides			
Bromeliaceae	Tillandsia heterophylla			
Bromeliaceae	Tillandsia ignesiae			
Bromeliaceae	Tillandsia imperialis	EN		
Bromeliaceae	Tillandsia inopinata			
Bromeliaceae	Tillandsia intermedia			
Bromeliaceae	Tillandsia ionantha			
Bromeliaceae	Tillandsia ionantha var. ionantha		VU	
Bromeliaceae	Tillandsia juerg-rutschmannii		CR	YES
Bromeliaceae	Tillandsia juncea			
Bromeliaceae	Tillandsia karwinskyana			
Bromeliaceae	Tillandsia kirchhoffiana			
Bromeliaceae	Tillandsia lampropoda	VU		
Bromeliaceae	Tillandsia lautneri			
Bromeliaceae	Tillandsia leiboldiana			
Bromeliaceae	Tillandsia lepidosepala			
Bromeliaceae	Tillandsia limbata			
Bromeliaceae	Tillandsia lucida			
Bromeliaceae	Tillandsia macdougallii			
Bromeliaceae	Tillandsia macrochlamys		VU	
Bromeliaceae	Tillandsia magnusiana			

Bromeliaceae	Tillandsia makoyana			
Bromeliaceae	Tillandsia malzinei			
Bromeliaceae	Tillandsia marabascoensis		EN	
Bromeliaceae	Tillandsia mirabilis		EN	
Bromeliaceae	Tillandsia multicaulis			
Bromeliaceae	Tillandsia oaxacana			
Bromeliaceae	Tillandsia orogenes		VU	
Bromeliaceae	Tillandsia plumosa			
Bromeliaceae	Tillandsia polita	VU	VU	
Bromeliaceae	Tillandsia polystachia			
Bromeliaceae	Tillandsia ponderosa	VU		
Bromeliaceae	Tillandsia pringlei			
Bromeliaceae	Tillandsia prodigiosa			
Bromeliaceae	Tillandsia pruinosa			
Bromeliaceae	Tillandsia pseudobaileyi			
Bromeliaceae	Tillandsia pseudosetacea			
Bromeliaceae	Tillandsia punctulata			
Bromeliaceae	Tillandsia quaquaflorifera		VU	
Bromeliaceae	Tillandsia recurvata			
Bromeliaceae	Tillandsia rhomboidea		EN	YES
Bromeliaceae	Tillandsia rodrigueziana			
Bromeliaceae	Tillandsia roland-gosselinii	VU		
Bromeliaceae	Tillandsia rotundata			
Bromeliaceae	Tillandsia schiedeana			
Bromeliaceae	Tillandsia seleriana	VU		
Bromeliaceae	Tillandsia setacea			
Bromeliaceae	Tillandsia socialis	VU	EN	
Bromeliaceae	Tillandsia standleyi		EN	
Bromeliaceae	Tillandsia streptophylla			
Bromeliaceae	Tillandsia takizawae		EN	
Bromeliaceae	Tillandsia tehuacana			
Bromeliaceae	Tillandsia tenuifolia		EN	
Bromeliaceae	Tillandsia tricolor	VU		
Bromeliaceae	Tillandsia usneoides			

Bromeliaceae	Tillandsia utriculata				
Bromeliaceae	Tillandsia variabilis				
Bromeliaceae	Tillandsia vicentina				
Bromeliaceae	Tillandsia violacea				
Bromeliaceae	Tillandsia zoquensis			VU	
Bromeliaceae	Werauhia gladioliflora				
Bromeliaceae	Werauhia hygrometrica			EN	
Bromeliaceae	Werauhia pectinata			VU	
Bromeliaceae	Werauhia pycnantha	VU			
Bromeliaceae	Werauhia vanhyningii				
Bromeliaceae	Werauhia werckleana	VU			
Brunelliaceae	Brunellia mexicana				
Burmanniaceae	Apteria aphylla				
Burmanniaceae	Burmannia flava			CR	YES
Burmanniaceae	Dictyostega orobanchoides				
Burmanniaceae	Gymnosiphon divaricatus			EN	
Burmanniaceae	Gymnosiphon panamensis			VU	
Burmanniaceae	Gymnosiphon suaveolens				
Burseraceae	Beiselia mexicana	EN		VU	
Burseraceae	Bursera aptera				
Burseraceae	Bursera ariensis				
Burseraceae	Bursera attenuata				
Burseraceae	Bursera bicolor				
Burseraceae	Bursera biflora				
Burseraceae	Bursera bipinnata				
Burseraceae	Bursera cinerea				
Burseraceae	Bursera citronella		VU		
Burseraceae	Bursera copallifera				
Burseraceae	Bursera denticulata		VU		
Burseraceae	Bursera discolor				
Burseraceae	Bursera diversifolia				
Burseraceae	Bursera esparzae		VU		
Burseraceae	Bursera excelsa				
Burseraceae	Bursera fagaroides				

Burseraceae	Bursera galeottiana			
Burseraceae	Bursera glabrifolia			
Burseraceae	Bursera grandifolia			
Burseraceae	Bursera graveolens			
Burseraceae	Bursera heliae		EN	
Burseraceae	Bursera heteresthes		EN	
Burseraceae	Bursera hintonii		VU	
Burseraceae	Bursera instabilis		VU	
Burseraceae	Bursera kerberi			
Burseraceae	Bursera krusei		VU	
Burseraceae	Bursera lancifolia			
Burseraceae	Bursera laurihuertae		VU	
Burseraceae	Bursera linanoe		VU	
Burseraceae	Bursera longipes			
Burseraceae	Bursera mirandae		VU	
Burseraceae	Bursera ovalifolia			
Burseraceae	Bursera roseana			
Burseraceae	Bursera sarcopoda		EN	
Burseraceae	Bursera schlechtendalii			
Burseraceae	Bursera simaruba			
Burseraceae	Bursera submoniliformis			
Burseraceae	Bursera tecomaca		EN	
Burseraceae	Bursera tomentosa			
Burseraceae	Bursera vejar-vazquezii		VU	
Burseraceae	Protium confusum			
Burseraceae	Protium copal			
Burseraceae	Protium glabrum			
Burseraceae	Protium heptaphyllum			EN
Burseraceae	Protium multiramiflorum		VU	
Buxaceae	Buxus bartlettii			
Buxaceae	Buxus lancifolia			EN
Buxaceae	Buxus moctezumae		EN	VU
Cabombaceae	Brasenia schreberi	VU		VU
Cabombaceae	Cabomba palaeformis			

Cactaceae	Acanthocereus castellae			VU
Cactaceae	Acanthocereus chiapensis			
Cactaceae	Acanthocereus fosterianus	NT		
Cactaceae	Acanthocereus oaxacensis			
Cactaceae	Acanthocereus tetragonus			
Cactaceae	Aporocactus flagelliformis	EN		
Cactaceae	Aporocactus martianus			
Cactaceae	Astrophytum ornatum	VU	VU	
Cactaceae	Brasiliopuntia brasiliensis			VU
Cactaceae	Cephalocereus apicicephalium			
Cactaceae	Cephalocereus euphorbioides			
Cactaceae	Cephalocereus fulviceps			
Cactaceae	Cephalocereus mezcalaensis			
Cactaceae	Cephalocereus nudus			
Cactaceae	Cephalocereus polylophus			
Cactaceae	Cephalocereus scoparius			
Cactaceae	Cephalocereus tetetzo			
Cactaceae	Coryphantha compacta			
Cactaceae	Coryphantha elephantidens			
Cactaceae	Coryphantha elephantidens subsp. bumamma			
Cactaceae	Coryphantha erecta			
Cactaceae	Coryphantha octacantha			
Cactaceae	Coryphantha ottonis			
Cactaceae	Coryphantha pallida			
Cactaceae	Coryphantha pycnacantha		EN	
Cactaceae	Coryphantha retusa	NT		
Cactaceae	Cumarinia odorata	NT		
Cactaceae	Cylindropuntia imbricata			
Cactaceae	Cylindropuntia leptocaulis			
Cactaceae	Cylindropuntia tunicata			
Cactaceae	Deamia testudo			
Cactaceae	Disocactus ackermannii			
Cactaceae	Disocactus ackermannii subsp. conzattianus			EN
Cactaceae	Disocactus crenatus			

Cactaceae	Disocactus macranthus			
Cactaceae	Disocactus nelsonii			
Cactaceae	Disocactus phyllanthoides	VU	VU	
Cactaceae	Disocactus quezaltecus			EN
Cactaceae	Disocactus speciosus			
Cactaceae	Disocactus speciosus subsp. cinnabarinus			
Cactaceae	Echinocactus platyacanthus	EN		
Cactaceae	Echinocactus texensis			
Cactaceae	Echinocereus pentalophus			
Cactaceae	Epiphyllum hookeri			
Cactaceae	Epiphyllum oxypetalum			
Cactaceae	Epiphyllum phyllanthus			
Cactaceae	Epiphyllum pumilum			
Cactaceae	Epiphyllum thomasianum			
Cactaceae	Ferocactus echidne			
Cactaceae	Ferocactus flavovirens		EN	
Cactaceae	Ferocactus haematacanthus	EN	EN	
Cactaceae	Ferocactus latispinus			
Cactaceae	Ferocactus robustus		VU	
Cactaceae	Kadenicarpus horripilus			
Cactaceae	Leuenbergeria lychnidiflora			
Cactaceae	Lophocereus marginatus			
Cactaceae	Mammillaria albilanata			
Cactaceae	Mammillaria beneckei			
Cactaceae	Mammillaria columbiana subsp. yucatanensis	VU		
Cactaceae	Mammillaria compressa			
Cactaceae	Mammillaria crinita			
Cactaceae	Mammillaria discolor			
Cactaceae	Mammillaria eriacantha		VU	
Cactaceae	Mammillaria geminispina			
Cactaceae	Mammillaria haageana			
Cactaceae	Mammillaria karwinskiana			
Cactaceae	Mammillaria karwinskiana subsp. collinsii			
Cactaceae	Mammillaria longimamma	VU	VU	

Cactaceae	Mammillaria magnimamma			
Cactaceae	Mammillaria mystax			
Cactaceae	Mammillaria parkinsonii	NT	EN	
Cactaceae	Mammillaria pectinifera	VU	EN	
Cactaceae	Mammillaria polythele			
Cactaceae	Mammillaria poselgeri			
Cactaceae	Mammillaria prolifera			
Cactaceae	Mammillaria prolifera subsp. arachnoidea			EN
Cactaceae	Mammillaria sartorii			VU
Cactaceae	Mammillaria sphacelata			
Cactaceae	Mammillaria uncinata			
Cactaceae	Mammillaria voburnensis			
Cactaceae	Mammillaria zublerae		EN	EN
Cactaceae	Melocactus curvispinus			
Cactaceae	Mitrocereus militaris			
Cactaceae	Myrtillocactus geometrizans			
Cactaceae	Nyctocereus serpentinus			
Cactaceae	Opuntia aciculata			
Cactaceae	Opuntia auberi			
Cactaceae	Opuntia cochenillifera			
Cactaceae	Opuntia deamii			VU
Cactaceae	Opuntia decumbens			
Cactaceae	Opuntia dejecta			
Cactaceae	Opuntia depressa			
Cactaceae	Opuntia dillenii			
Cactaceae	Opuntia engelmannii			
Cactaceae	Opuntia engelmannii var. cuija			
Cactaceae	Opuntia engelmannii var. lindheimeri			
Cactaceae	Opuntia escuintlensis			VU
Cactaceae	Opuntia excelsa	NT		
Cactaceae	Opuntia ficus-indica			
Cactaceae	Opuntia huajuapensis			
Cactaceae	Opuntia humifusa			
Cactaceae	Opuntia hyptiacantha			

Cactaceae	Opuntia inaperta		
Cactaceae	Opuntia karwinskiana		
Cactaceae	Opuntia lasiacantha		
Cactaceae	Opuntia leucotricha		
Cactaceae	Opuntia lutea		VU
Cactaceae	Opuntia macrocentra		
Cactaceae	Opuntia microdasys		
Cactaceae	Opuntia phaeacantha		
Cactaceae	Opuntia pilifera		
Cactaceae	Opuntia puberula		
Cactaceae	Opuntia pubescens		
Cactaceae	Opuntia rastrera		
Cactaceae	Opuntia streptacantha		
Cactaceae	Opuntia stricta		
Cactaceae	Opuntia tomentosa		
Cactaceae	Opuntia velutina		
Cactaceae	Opuntia wilcoxii		
Cactaceae	Pachycereus eichlamii		
Cactaceae	Pachycereus grandis	VU	
Cactaceae	Pachycereus pecten-aboriginum		
Cactaceae	Peniocereus striatus		
Cactaceae	Peniocereus viperinus		
Cactaceae	Pereskia aculeata		
Cactaceae	Pereskiopsis diguetii		
Cactaceae	Pereskiopsis rotundifolia		
Cactaceae	Pilosocereus chrysacanthus		
Cactaceae	Pilosocereus collinsii		
Cactaceae	Pilosocereus leucocephalus		
Cactaceae	Pilosocereus purpusii		
Cactaceae	Pilosocereus quadricentralis	EN	
Cactaceae	Polaskia chichipe		
Cactaceae	Pseudorhipsalis ramulosa		
Cactaceae	Pterocereus gaumeri		
Cactaceae	Pterocereus gaumeri subsp. foetidus		EN

Cactaceae	Rhipsalis baccifera				
Cactaceae	Rhipsalis burchellii			CR	YES
Cactaceae	Schlumbergera russelliana		EN	EN	
Cactaceae	Selenicereus anthonyanus	VU			
Cactaceae	Selenicereus chontalensis			VU	
Cactaceae	Selenicereus grandiflorus				
Cactaceae	Selenicereus grandiflorus subsp. hondurensis			VU	
Cactaceae	Selenicereus hamatus				
Cactaceae	Selenicereus inermis			EN	YES
Cactaceae	Selenicereus ocamponis				
Cactaceae	Selenicereus pteranthus				
Cactaceae	Selenicereus purpusii				
Cactaceae	Selenicereus spinulosus				
Cactaceae	Selenicereus tricae			EN	
Cactaceae	Selenicereus undatus				
Cactaceae	Selenicereus vagans				
Cactaceae	Stenocereus chrysocarpus		EN		
Cactaceae	Stenocereus dumortieri				
Cactaceae	Stenocereus griseus				
Cactaceae	Stenocereus pruinosus				
Cactaceae	Stenocereus stellatus				
Cactaceae	Stenocereus treleasei				
Cactaceae	Thelocactus conothelos				
Cactaceae	Turbinicarpus schmiedickeanus	VU			
Cactaceae	Turbinicarpus schmiedickeanus subsp. jauernigii				
Cactaceae	Weberocereus glaber			VU	
Cactaceae	Weberocereus glaber subsp. mirandae			EN	
Calceolariaceae	Calceolaria chelidonioides			EN	
Calceolariaceae	Calceolaria mexicana				
Calceolariaceae	Calceolaria trilobata			VU	
Calceolariaceae	Calceolaria tripartita				
Calophyllaceae	Calophyllum brasiliense	VU			
Calophyllaceae	Calophyllum brasiliense var. antillanum			EN	
Calophyllaceae	Calophyllum brasiliense var. rekoi				

Calophyllaceae	Mammea americana		
Calophyllaceae	Marila laxiflora	EN	
Campanulaceae	Burmeistera virescens	VU	
Campanulaceae	Campanula medium	EN	
Campanulaceae	Centropogon cordifolius		
Campanulaceae	Centropogon grandidentatus		
Campanulaceae	Diastatea micrantha		
Campanulaceae	Diastatea tenera		
Campanulaceae	Heterotoma lobelioides		
Campanulaceae	Hippobroma longiflora		
Campanulaceae	Lobelia aguana		
Campanulaceae	Lobelia alsinoides var. trigona	CR	YES
Campanulaceae	Lobelia berlandieri		
Campanulaceae	Lobelia berlandieri subsp. berlandieri	VU	
Campanulaceae	Lobelia boykinii	EN	
Campanulaceae	Lobelia caeciliae	VU	
Campanulaceae	Lobelia cardinalis		
Campanulaceae	Lobelia cliffortiana	EN	
Campanulaceae	Lobelia cordifolia		
Campanulaceae	Lobelia diastateoides		
Campanulaceae	Lobelia divaricata		
Campanulaceae	Lobelia ehrenbergii		
Campanulaceae	Lobelia erinus	EN	
Campanulaceae	Lobelia fenestralis		
Campanulaceae	Lobelia gruina		
Campanulaceae	Lobelia gruina subsp. gruina		
Campanulaceae	Lobelia hartwegii		
Campanulaceae	Lobelia hintoniorum	EN	
Campanulaceae	Lobelia laxiflora		
Campanulaceae	Lobelia laxiflora subsp. angustifolia		
Campanulaceae	Lobelia laxiflora subsp. laxiflora		
Campanulaceae	Lobelia lithophila	CR	YES
Campanulaceae	Lobelia longicaulis		
Campanulaceae	Lobelia macdonaldii	EN	

Campanulaceae	Lobelia mexicana		
Campanulaceae	Lobelia occidentalis		
Campanulaceae	Lobelia parviflora	EN	
Campanulaceae	Lobelia purpusii		
Campanulaceae	Lobelia quiexobrae	CR	YES
Campanulaceae	Lobelia sartorii		
Campanulaceae	Lobelia stolonifera	VU	
Campanulaceae	Lobelia tarsophora		
Campanulaceae	Lobelia volcanica		
Campanulaceae	Lobelia xalapensis		
Campanulaceae	Lobelia yucatana	VU	
Campanulaceae	Pseudonemacladus oppositifolius		
Campanulaceae	Triodanis perfoliata		
Campanulaceae	Triodanis perfoliata subsp. perfoliata		
Cannabaceae	Aphananthe monoica		
Cannabaceae	Cannabis sativa		
Cannabaceae	Celtis caudata		
Cannabaceae	Celtis iguanaea		
Cannabaceae	Celtis laevigata		
Cannabaceae	Celtis pallida		
Cannabaceae	Celtis schippii	EN	
Cannabaceae	Celtis spinosa		
Cannabaceae	Lozanella enantiophylla		
Cannabaceae	Trema domingense	CR	YES
Cannaceae	Canna × hybrida		
Cannaceae	Canna glauca		
Cannaceae	Canna indica		
Cannaceae	Canna tuerckheimii		
Capparaceae	Capparis rheedei		
Capparaceae	Crateva palmeri		
Capparaceae	Crateva tapia		
Capparaceae	Morisonia americana		
Capparaceae	Morisonia amplissima	EN	
Capparaceae	Morisonia angustifolia		

Capparaceae	Morisonia asperifolia	
Capparaceae	Morisonia discolor	
Capparaceae	Morisonia flexuosa	
Capparaceae	Morisonia frondosa	
Capparaceae	Morisonia incana	
Capparaceae	Morisonia indica	
Capparaceae	Morisonia lundellii	
Capparaceae	Morisonia mollicella	
Capparaceae	Morisonia odoratissmia	
Capparaceae	Morisonia pachaca	
Capparaceae	Morisonia pittieri	EN
Capparaceae	Morisonia pringlei	
Capparaceae	Morisonia quiriguensis	
Capparaceae	Morisonia verrucosa	
Caprifoliaceae	Linnaea coriacea	
Caprifoliaceae	Linnaea floribunda	
Caprifoliaceae	Lonicera japonica	
Caprifoliaceae	Lonicera mexicana	
Caprifoliaceae	Lonicera pilosa	
Caprifoliaceae	Symphoricarpos microphyllus	
Caprifoliaceae	Valeriana barbareifolia	
Caprifoliaceae	Valeriana chiapensis	VU
Caprifoliaceae	Valeriana clematitis	
Caprifoliaceae	Valeriana cucurbitifolia	
Caprifoliaceae	Valeriana deltoidea	
Caprifoliaceae	Valeriana densiflora	
Caprifoliaceae	Valeriana edulis subsp. procera	
Caprifoliaceae	Valeriana naidae	
Caprifoliaceae	Valeriana palmeri	
Caprifoliaceae	Valeriana philippiana	EN
Caprifoliaceae	Valeriana prionophylla	VU
Caprifoliaceae	Valeriana pulchella	
Caprifoliaceae	Valeriana robertianifolia	
Caprifoliaceae	Valeriana scandens	

Caprifoliaceae	Valeriana scandens var. candolleana			
Caprifoliaceae	Valeriana sorbifolia			
Caprifoliaceae	Valeriana sorbifolia var. mexicana			
Caprifoliaceae	Valeriana urticifolia			
Caprifoliaceae	Vesalea coriacea			
Caprifoliaceae	Vesalea floribunda			
Caricaceae	Carica papaya			
Caricaceae	Horovitzia cnidoscoloides		VU	
Caricaceae	Jacaratia dolichaula			
Caricaceae	Jacaratia mexicana			
Caricaceae	Jarilla chocola			
Caricaceae	Jarilla heterophylla			
Caricaceae	Vasconcellea cauliflora			
Caryophyllaceae	Achyronychia cooperi			
Caryophyllaceae	Arenaria bryoides	NT		
Caryophyllaceae	Arenaria lanuginosa			
Caryophyllaceae	Arenaria lanuginosa var. saxosa			
Caryophyllaceae	Arenaria lycopodioides			
Caryophyllaceae	Arenaria moehringioides			
Caryophyllaceae	Arenaria reptans			
Caryophyllaceae	Cerastium glomeratum			
Caryophyllaceae	Cerastium guatemalense		VU	
Caryophyllaceae	Cerastium nutans			
Caryophyllaceae	Cerastium orithales			
Caryophyllaceae	Cerastium ramigerum			
Caryophyllaceae	Dianthus barbatus		VU	
Caryophyllaceae	Dianthus carthusianorum		EN	
Caryophyllaceae	Dianthus caryophyllus			
Caryophyllaceae	Dianthus deltoides		CR	YES
Caryophyllaceae	Drymaria cordata			
Caryophyllaceae	Drymaria effusa			
Caryophyllaceae	Drymaria glandulosa			
Caryophyllaceae	Drymaria gracilis			
Caryophyllaceae	Drymaria laxiflora			

Caryophyllaceae	Drymaria molluginea			
Caryophyllaceae	Drymaria multiflora			
Caryophyllaceae	Drymaria villosa			
Caryophyllaceae	Drymaria villosa subsp. palustris			
Caryophyllaceae	Drymaria xerophylla			
Caryophyllaceae	Paronychia mexicana			
Caryophyllaceae	Sagina apetala		EN	
Caryophyllaceae	Saponaria officinalis			
Caryophyllaceae	Scopulophila parryi			
Caryophyllaceae	Silene laciniata			
Caryophyllaceae	Stellaria alsine		VU	
Caryophyllaceae	Stellaria cuspidata			
Caryophyllaceae	Stellaria cuspidata subsp. prostrata			
Caryophyllaceae	Stellaria graminea		VU	
Caryophyllaceae	Stellaria irazuensis		VU	
Caryophyllaceae	Stellaria media			
Caryophyllaceae	Stellaria miahuatlana		EN	
Caryophyllaceae	Stellaria nemorum			
Caryophyllaceae	Stellaria ovata			
Caryophyllaceae	Triplateia moehringiodes			
Celastraceae	Acanthothamnus aphyllus			
Celastraceae	Celastrus lenticellatus		EN	
Celastraceae	Celastrus pringlei			
Celastraceae	Celastrus vulcanicola			
Celastraceae	Cheiloclinium belizense		VU	
Celastraceae	Crossopetalum densiflorum	EN	VU	
Celastraceae	Crossopetalum filipes			
Celastraceae	Crossopetalum glabrum	CR	EN	
Celastraceae	Crossopetalum lanceifolium		CR	YES
Celastraceae	Crossopetalum macrocarpum		EN	
Celastraceae	Crossopetalum minimiflorum	VU		
Celastraceae	Crossopetalum parviflorum			
Celastraceae	Crossopetalum riparium		EN	
Celastraceae	Crossopetalum scoparium			

Celastraceae	Crossopetalum standleyi		
Celastraceae	Crossopetalum uragoga		
Celastraceae	Cuervea kappleriana		
Celastraceae	Elaeodendron xylocarpum		
Celastraceae	Euonymus benthamii	VU	
Celastraceae	Euonymus chiapensis	CR	VU
Celastraceae	Euonymus mexicanus	EN	
Celastraceae	Gyminda tonduzii		
Celastraceae	Hippocratea volubilis		
Celastraceae	Maytenus chiapensis		
Celastraceae	Maytenus matudae		
Celastraceae	Maytenus phyllanthoides		
Celastraceae	Maytenus repanda		
Celastraceae	Maytenus schippii		
Celastraceae	Maytenus stipitata		
Celastraceae	Mortonia diffusa		
Celastraceae	Orthosphenia mexicana		
Celastraceae	Pristimera celastroides		
Celastraceae	Quetzalia contracta		
Celastraceae	Quetzalia schiedeana		
Celastraceae	Salacia cordata		
Celastraceae	Salacia cordata subsp. petenensis		VU
Celastraceae	Salacia impressifolia		
Celastraceae	Schaefferia frutescens		
Celastraceae	Schaefferia stenophylla		
Celastraceae	Semialarium mexicanum		
Celastraceae	Wimmeria acuminata	EN	
Celastraceae	Wimmeria bartlettii		
Celastraceae	Wimmeria chiapensis	EN	EN
Celastraceae	Wimmeria concolor	VU	
Celastraceae	Wimmeria cyclocarpa	EN	VU
Celastraceae	Wimmeria lanceolata		
Celastraceae	Wimmeria microphylla		
Celastraceae	Wimmeria montana	VU	

Celastraceae	Wimmeria persicifolia				
Celastraceae	Wimmeria pubescens				
Celastraceae	Wimmeria serrulata				
Celastraceae	Wimmeria sternii		EN		
Celastraceae	Zinowiewia concinna	EN	EN		
Celastraceae	Zinowiewia integerrima				
Celastraceae	Zinowiewia matudae				
Celastraceae	Zinowiewia pauciflora			EN	YES
Celastraceae	Zinowiewia rubra		VU		
Celastraceae	Zinowiewia tacanensis			VU	
Ceratophyllaceae	Ceratophyllum demersum				
Chloranthaceae	Hedyosmum mexicanum		VU		
Chrysobalanaceae	Chrysobalanus icaco				
Chrysobalanaceae	Couepia polyandra				
Chrysobalanaceae	Geobalanus retifolius		EN		
Chrysobalanaceae	Hirtella americana				
Chrysobalanaceae	Hirtella racemosa				
Chrysobalanaceae	Hirtella racemosa var. hexandra				
Chrysobalanaceae	Hirtella triandra				
Chrysobalanaceae	Hirtella triandra subsp. media				
Chrysobalanaceae	Hirtella triandra subsp. triandra			VU	
Chrysobalanaceae	Leptobalanus sparsipilis		VU		
Chrysobalanaceae	Licania hypoleuca				
Chrysobalanaceae	Licania hypoleuca var. hypoleuca			VU	
Chrysobalanaceae	Microdesmia arborea				
Chrysobalanaceae	Moquilea platypus				
Cistaceae	Crocanthemum glomeratum				
Cistaceae	Helianthemum coulteri				
Cistaceae	Helianthemum patens				
Cistaceae	Lechea tripetala				
Cleomaceae	Cleome aculeata				
Cleomaceae	Cleome dodecandra				
Cleomaceae	Cleome gigantea				
Cleomaceae	Cleome guianensis				

Cleomaceae	Cleome gynandra		
Cleomaceae	Cleome hemsleyana		
Cleomaceae	Cleome houtteana		
Cleomaceae	Cleome magnifica		
Cleomaceae	Cleome parviflora		VU
Cleomaceae	Cleome pilosa		
Cleomaceae	Cleome serrata		
Cleomaceae	Cleome speciosa		
Cleomaceae	Cleome spinosa		
Cleomaceae	Cleome uniglandulosa		
Cleomaceae	Cleome usambarica		EN
Cleomaceae	Cleome viscosa		
Clethraceae	Clethra alcoceri	VU	
Clethraceae	Clethra chiapensis	EN	EN
Clethraceae	Clethra conzattiana	EN	VU
Clethraceae	Clethra galeottiana		
Clethraceae	Clethra hartwegii		
Clethraceae	Clethra hondurensis		
Clethraceae	Clethra macrophylla		
Clethraceae	Clethra mexicana		
Clethraceae	Clethra occidentalis		
Clethraceae	Clethra oleoides	VU	
Clethraceae	Clethra pachecoana	VU	
Clethraceae	Clethra pringlei		
Clethraceae	Clethra purpusii	EN	EN
Clethraceae	Clethra suaveolens		
Clethraceae	Clethra tuxtlensis	VU	EN
Clethraceae	Clethra vicentina		
Clusiaceae	Chrysochlamys guatemaltecana		
Clusiaceae	Chrysochlamys nicaraguensis		VU
Clusiaceae	Chrysochlamys psychotriifolia		VU
Clusiaceae	Clusia belizensis		VU
Clusiaceae	Clusia flava		
Clusiaceae	Clusia flavida		CR

Clusiaceae	Clusia guatemalensis			
Clusiaceae	Clusia guatemalensis var. orizabae		VU	
Clusiaceae	Clusia lundellii			
Clusiaceae	Clusia lusoria			
Clusiaceae	Clusia massoniana			
Clusiaceae	Clusia minor			
Clusiaceae	Clusia quadrangula			
Clusiaceae	Clusia rosea			
Clusiaceae	Clusia salvinii			
Clusiaceae	Clusia tetra-trianthera		VU	
Clusiaceae	Clusia uvitana		EN	
Clusiaceae	Garcinia buchneri			
Clusiaceae	Garcinia intermedia			
Clusiaceae	Garcinia macrantha		EN	
Clusiaceae	Garcinia macrophylla			
Clusiaceae	Garcinia parviflora		CR	YES
Clusiaceae	Garcinia parvifolia		CR	YES
Clusiaceae	Symphonia globulifera		VU	
Combretaceae	Combretum argenteum			
Combretaceae	Combretum decandrum			
Combretaceae	Combretum farinosum			
Combretaceae	Combretum fruticosum			
Combretaceae	Combretum grandiflorum		EN	
Combretaceae	Combretum laxum			
Combretaceae	Combretum rovirosae			
Combretaceae	Conocarpus erectus	NT		
Combretaceae	Laguncularia racemosa	VU		
Combretaceae	Terminalia amazonia			
Combretaceae	Terminalia buceras			
Combretaceae	Terminalia catappa			
Combretaceae	Terminalia macrostachya			
Combretaceae	Terminalia oblonga			
Commelinaceae	Callisia amplexicaulis			
Commelinaceae	Callisia angustifolia			

Commelinaceae	Callisia cordifolia		
Commelinaceae	Callisia disgrega		
Commelinaceae	Callisia fragrans		
Commelinaceae	Callisia gentlei		
Commelinaceae	Callisia gentlei var. macdougallii	VU	
Commelinaceae	Callisia grandiflora		
Commelinaceae	Callisia insignis		
Commelinaceae	Callisia monandra		
Commelinaceae	Callisia montana		
Commelinaceae	Callisia multiflora		
Commelinaceae	Callisia navicularis		
Commelinaceae	Callisia palmeri		
Commelinaceae	Callisia purpurascens		
Commelinaceae	Callisia repens		
Commelinaceae	Callisia serrulata		
Commelinaceae	Callisia tehuantepecana	VU	
Commelinaceae	Commelina bravoa	CR	YES
Commelinaceae	Commelina communis	EN	
Commelinaceae	Commelina dianthifolia		
Commelinaceae	Commelina diffusa		
Commelinaceae	Commelina diffusa var. gigas	VU	
Commelinaceae	Commelina elliptica		
Commelinaceae	Commelina erecta		
Commelinaceae	Commelina erecta subsp. erecta		
Commelinaceae	Commelina geniculata	EN	
Commelinaceae	Commelina leiocarpa		
Commelinaceae	Commelina obliqua		
Commelinaceae	Commelina pallida		
Commelinaceae	Commelina rufipes		
Commelinaceae	Commelina standleyi		
Commelinaceae	Commelina texcocana		
Commelinaceae	Commelina tuberosa		
Commelinaceae	Cyanotis loureiroana	VU	
Commelinaceae	Dichorisandra amabilis	EN	

Commelinaceae	Dichorisandra hexandra	
Commelinaceae	Dichorisandra thyrsiflora	VU
Commelinaceae	Elasis guatemalensis	
Commelinaceae	Gibasis consobrina	
Commelinaceae	Gibasis geniculata	
Commelinaceae	Gibasis karwinskyana	
Commelinaceae	Gibasis linearis	
Commelinaceae	Gibasis oaxacana	
Commelinaceae	Gibasis pellucida	
Commelinaceae	Gibasis pulchella	
Commelinaceae	Gibasoides laxiflora	
Commelinaceae	Matudanthus nanus	
Commelinaceae	Murdannia nudiflora	
Commelinaceae	Thyrsanthemum floribundum	
Commelinaceae	Thyrsanthemum macrophyllum	
Commelinaceae	Tinantia erecta	
Commelinaceae	Tinantia glabra	VU
Commelinaceae	Tinantia leiocalyx	
Commelinaceae	Tinantia longipedunculata	
Commelinaceae	Tinantia pringlei	
Commelinaceae	Tinantia standleyi	
Commelinaceae	Tinantia violacea	
Commelinaceae	Tradescantia andrieuxii	
Commelinaceae	Tradescantia brevifolia	
Commelinaceae	Tradescantia buckleyi	
Commelinaceae	Tradescantia commelinoides	
Commelinaceae	Tradescantia crassifolia	
Commelinaceae	Tradescantia crassifolia var. acaulis	
Commelinaceae	Tradescantia cymbispatha	CR
Commelinaceae	Tradescantia deficiens	
Commelinaceae	Tradescantia exaltata	EN
Commelinaceae	Tradescantia fluminensis	
Commelinaceae	Tradescantia guiengolensis	EN
Commelinaceae	Tradescantia huehueteca	EN

Commelinaceae	Tradescantia pallida	
Commelinaceae	Tradescantia plusiantha	
Commelinaceae	Tradescantia poelliae	
Commelinaceae	Tradescantia schippii	
Commelinaceae	Tradescantia soconuscana	
Commelinaceae	Tradescantia spathacea	
Commelinaceae	Tradescantia standleyi	EN
Commelinaceae	Tradescantia zanonia	
Commelinaceae	Tradescantia zebrina	
Commelinaceae	Tradescantia zebrina var. flocculosa	VU
Commelinaceae	Tradescantia zebrina var. mollipila	VU
Commelinaceae	Tradescantia zebrina var. zebrina	
Commelinaceae	Weldenia candida	
Connaraceae	Cnestidium rufescens	
Connaraceae	Connarus lambertii	VU
Connaraceae	Connarus lentiginosus	
Connaraceae	Connarus schultesii	
Connaraceae	Connarus stenophyllus	EN
Connaraceae	Rourea glabra	
Connaraceae	Rourea schippii	
Convolvulaceae	Aniseia martinicensis	
Convolvulaceae	Bonamia sulphurea	
Convolvulaceae	Camonea umbellata	
Convolvulaceae	Convolvulus arvensis	
Convolvulaceae	Convolvulus equitans	
Convolvulaceae	Cuscuta corymbosa	
Convolvulaceae	Cuscuta cozumeliensis	EN
Convolvulaceae	Cuscuta desmouliniana	
Convolvulaceae	Cuscuta jalapensis	
Convolvulaceae	Cuscuta ortegana	VU
Convolvulaceae	Cuscuta rugosiceps	
Convolvulaceae	Cuscuta tinctoria	
Convolvulaceae	Cuscuta woodsonii	VU
Convolvulaceae	Dichondra argentea	

Convolvulaceae	Dichondra repens	
Convolvulaceae	Dichondra sericea	
Convolvulaceae	Distimake aegyptius	
Convolvulaceae	Distimake austinii	EN
Convolvulaceae	Distimake cielensis	EN
Convolvulaceae	Distimake cissoides	
Convolvulaceae	Distimake dissectus	
Convolvulaceae	Distimake macrocalyx	CR
Convolvulaceae	Distimake quinquefolius	
Convolvulaceae	Distimake tuberosus	
Convolvulaceae	Evolvulus alsinoides	
Convolvulaceae	Evolvulus nummularius	
Convolvulaceae	Evolvulus ovatus	
Convolvulaceae	Evolvulus prostratus	
Convolvulaceae	Evolvulus sericeus	
Convolvulaceae	Ipomoea aculeata	EN
Convolvulaceae	Ipomoea alba	
Convolvulaceae	Ipomoea anisomeres	
Convolvulaceae	Ipomoea arborescens	
Convolvulaceae	Ipomoea aristolochiifolia	
Convolvulaceae	Ipomoea aurantiaca	VU
Convolvulaceae	Ipomoea batatas	
Convolvulaceae	Ipomoea batatas var. apiculata	VU
Convolvulaceae	Ipomoea batatas var. batatas	
Convolvulaceae	Ipomoea batatoides	
Convolvulaceae	Ipomoea bernoulliana	EN
Convolvulaceae	Ipomoea bombycina	VU
Convolvulaceae	Ipomoea bracteata	
Convolvulaceae	Ipomoea bracteata var. bracteata	VU
Convolvulaceae	Ipomoea cairica	
Convolvulaceae	Ipomoea capillacea	
Convolvulaceae	Ipomoea carnea	
Convolvulaceae	Ipomoea carnea subsp. carnea	
Convolvulaceae	Ipomoea carnea subsp. fistulosa	

Convolvulaceae	Ipomoea chenopodiifolia		
Convolvulaceae	Ipomoea cholulensis		
Convolvulaceae	Ipomoea clavata		
Convolvulaceae	Ipomoea coccinea		
Convolvulaceae	Ipomoea concolora	VU	
Convolvulaceae	Ipomoea conzattii		
Convolvulaceae	Ipomoea cordatotriloba		
Convolvulaceae	Ipomoea corymbosa		
Convolvulaceae	Ipomoea costellata		
Convolvulaceae	Ipomoea crinicalyx		
Convolvulaceae	Ipomoea cristulata		
Convolvulaceae	Ipomoea dimorphophylla		
Convolvulaceae	Ipomoea dumetorum		
Convolvulaceae	Ipomoea dumosa		
Convolvulaceae	Ipomoea electrina	CR	YES
Convolvulaceae	Ipomoea elongata		
Convolvulaceae	Ipomoea funis		
Convolvulaceae	Ipomoea funis var. funis		
Convolvulaceae	Ipomoea funis var. langlassei		
Convolvulaceae	Ipomoea hartwegii		
Convolvulaceae	Ipomoea hastigera		
Convolvulaceae	Ipomoea hederacea		
Convolvulaceae	Ipomoea hederifolia		
Convolvulaceae	Ipomoea heterodoxa		
Convolvulaceae	Ipomoea hochstetteri	VU	
Convolvulaceae	Ipomoea imperati		
Convolvulaceae	Ipomoea incarnata	CR	YES
Convolvulaceae	Ipomoea indica		
Convolvulaceae	Ipomoea intrapilosa		
Convolvulaceae	Ipomoea jalapa		
Convolvulaceae	Ipomoea leucotricha	VU	
Convolvulaceae	Ipomoea lindenii		
Convolvulaceae	Ipomoea lobata		
Convolvulaceae	Ipomoea lottiae	VU	

Convolvulaceae	Ipomoea lutea	
Convolvulaceae	Ipomoea mairetii	
Convolvulaceae	Ipomoea meyeri	
Convolvulaceae	Ipomoea microsepala	
Convolvulaceae	Ipomoea mitchellae	
Convolvulaceae	Ipomoea murucoides	
Convolvulaceae	Ipomoea neei	
Convolvulaceae	Ipomoea neurocephala	
Convolvulaceae	Ipomoea nil	
Convolvulaceae	Ipomoea orizabensis	
Convolvulaceae	Ipomoea orizabensis var. orizabensis	
Convolvulaceae	Ipomoea pauciflora	
Convolvulaceae	Ipomoea pedicellaris	
Convolvulaceae	Ipomoea pes-caprae	
Convolvulaceae	Ipomoea peteri	
Convolvulaceae	Ipomoea philomega	
Convolvulaceae	Ipomoea plummerae var. plummerae	VU
Convolvulaceae	Ipomoea populina	
Convolvulaceae	Ipomoea praecana	
Convolvulaceae	Ipomoea pubescens	
Convolvulaceae	Ipomoea puncticulata	
Convolvulaceae	Ipomoea purga	
Convolvulaceae	Ipomoea purpurea	
Convolvulaceae	Ipomoea quamoclit	
Convolvulaceae	Ipomoea ramosissima	VU
Convolvulaceae	Ipomoea reticulata	
Convolvulaceae	Ipomoea robinsonii	
Convolvulaceae	Ipomoea sagittata	
Convolvulaceae	Ipomoea santillanii	
Convolvulaceae	Ipomoea seducta	
Convolvulaceae	Ipomoea sepacuitensis	VU
Convolvulaceae	Ipomoea setifera	EN
Convolvulaceae	Ipomoea setosa	
Convolvulaceae	Ipomoea silvicola	

Convolvulaceae	Ipomoea simulans	
Convolvulaceae	Ipomoea splendor-sylvae	
Convolvulaceae	Ipomoea squamosa	
Convolvulaceae	Ipomoea stans	
Convolvulaceae	Ipomoea suaveolens	
Convolvulaceae	Ipomoea suffulta	
Convolvulaceae	Ipomoea tehuantepecensis	EN
Convolvulaceae	Ipomoea ternifolia	
Convolvulaceae	Ipomoea tiliacea	
Convolvulaceae	Ipomoea tricolor	
Convolvulaceae	Ipomoea trifida	
Convolvulaceae	Ipomoea triloba	
Convolvulaceae	Ipomoea villifera	
Convolvulaceae	Ipomoea wolcottiana	
Convolvulaceae	Itzaea sericea	
Convolvulaceae	Jacquemontia abutiloides	
Convolvulaceae	Jacquemontia confusa	
Convolvulaceae	Jacquemontia evolvuloides	
Convolvulaceae	Jacquemontia nodiflora	
Convolvulaceae	Jacquemontia oaxacana	
Convolvulaceae	Jacquemontia paniculata	EN
Convolvulaceae	Jacquemontia pentanthos	
Convolvulaceae	Jacquemontia polyantha	
Convolvulaceae	Jacquemontia pycnocephala	VU
Convolvulaceae	Jacquemontia smithii	
Convolvulaceae	Jacquemontia sphaerostigma	
Convolvulaceae	Jacquemontia tamnifolia	
Convolvulaceae	Jacquemontia verticillata	
Convolvulaceae	Merremia discoidesperma	
Convolvulaceae	Merremia platyphylla	
Convolvulaceae	Merremia poranoides	
Convolvulaceae	Odonellia hirtiflora	
Convolvulaceae	Operculina pinnatifida	
Convolvulaceae	Operculina pteripes	

Convolvulaceae	Operculina ventricosa	EN
Convolvulaceae	Porana nutans	
Convolvulaceae	Xenostegia pinnata	VU
Coriariaceae	Coriaria ruscifolia	
Coriariaceae	Coriaria ruscifolia subsp. microphylla	
Cornaceae	Cornus disciflora	
Cornaceae	Cornus excelsa	
Cornaceae	Cornus florida	
Costaceae	Costus comosus var. bakeri	VU
Costaceae	Costus dirzoi	
Costaceae	Costus guanaiensis	VU
Costaceae	Costus guanaiensis var. macrostrobilus	VU
Costaceae	Costus laevis	VU
Costaceae	Costus pictus	
Costaceae	Costus pulverulentus	
Costaceae	Costus scaber	
Costaceae	Costus spicatus	
Costaceae	Costus villosissimus	
Costaceae	Hellenia speciosa	
Crassulaceae	Echeveria acutifolia	
Crassulaceae	Echeveria alata	EN
Crassulaceae	Echeveria amoena	
Crassulaceae	Echeveria bifurcata	
Crassulaceae	Echeveria carminea	VU
Crassulaceae	Echeveria chiapensis	
Crassulaceae	Echeveria coccinea	
Crassulaceae	Echeveria fulgens	
Crassulaceae	Echeveria gibbiflora	
Crassulaceae	Echeveria gigantea	
Crassulaceae	Echeveria goldmanii	EN
Crassulaceae	Echeveria guatemalensis	EN
Crassulaceae	Echeveria heterosepala	
Crassulaceae	Echeveria lilacina	
Crassulaceae	Echeveria megacalyx	

Crassulaceae	Echeveria microcalyx			VU
Crassulaceae	Echeveria mucronata			
Crassulaceae	Echeveria nodulosa			
Crassulaceae	Echeveria nuda			VU
Crassulaceae	Echeveria paniculata var. maculata			
Crassulaceae	Echeveria prunina			EN
Crassulaceae	Echeveria pubescens			EN
Crassulaceae	Echeveria racemosa			
Crassulaceae	Echeveria rosea			
Crassulaceae	Echeveria rubromarginata			
Crassulaceae	Echeveria runyonii			
Crassulaceae	Echeveria secunda			
Crassulaceae	Echeveria semivestita			
Crassulaceae	Echeveria semivestita var. floresiana			EN
Crassulaceae	Echeveria subalpina			
Crassulaceae	Echeveria tamaulipana			VU
Crassulaceae	Echeveria viridissima			EN
Crassulaceae	Echeveria walpoleana			
Crassulaceae	Graptopetalum macdougallii	EN		
Crassulaceae	Kalanchoe daigremontiana		EN	
Crassulaceae	Kalanchoe delagoensis			
Crassulaceae	Kalanchoe fedtschenkoi			
Crassulaceae	Kalanchoe glaucescens			EN
Crassulaceae	Kalanchoe marmorata			
Crassulaceae	Kalanchoe mortagei			EN
Crassulaceae	Kalanchoe pinnata			
Crassulaceae	Kalanchoe rosei			EN
Crassulaceae	Pachyphytum longifolium			VU
Crassulaceae	Sedum adolphi			
Crassulaceae	Sedum batesii			
Crassulaceae	Sedum botterii			
Crassulaceae	Sedum calcicola			
Crassulaceae	Sedum chloropetalum			EN
Crassulaceae	Sedum commixtum			EN

Crassulaceae	Sedum corynephyllum		
Crassulaceae	Sedum dendroideum		
Crassulaceae	Sedum goldmanii		
Crassulaceae	Sedum guatemalense		
Crassulaceae	Sedum hultenii		
Crassulaceae	Sedum jerzedowskii	EN	
Crassulaceae	Sedum jurgensenii		
Crassulaceae	Sedum jurgensenii subsp. attenuatum	EN	
Crassulaceae	Sedum liebmannianum		
Crassulaceae	Sedum lucidum	EN	
Crassulaceae	Sedum luteoviride	CR	YES
Crassulaceae	Sedum mesoamericanum	VU	
Crassulaceae	Sedum moranense		
Crassulaceae	Sedum palmeri		
Crassulaceae	Sedum praealtum		
Crassulaceae	Sedum quevae		
Crassulaceae	Sedum retusum		
Crassulaceae	Sedum stahlii		
Crassulaceae	Thompsonella minutiflora		
Crassulaceae	Villadia guatemalensis		
Crassulaceae	Villadia misera		
Crossosomataceae	Velascoa recondita	EN	
Cucurbitaceae	Cayaponia attenuata		
Cucurbitaceae	Cayaponia longiloba		
Cucurbitaceae	Cayaponia racemosa		
Cucurbitaceae	Cionosicys excisus		
Cucurbitaceae	Cionosicys macranthus		
Cucurbitaceae	Citrullus lanatus		
Cucurbitaceae	Cucumis anguria		
Cucurbitaceae	Cucumis melo		
Cucurbitaceae	Cucurbita argyrosperma		
Cucurbitaceae	Cucurbita digitata		
Cucurbitaceae	Cucurbita ficifolia		
Cucurbitaceae	Cucurbita maxima		

Cucurbitaceae	Cucurbita moschata		
Cucurbitaceae	Cucurbita okeechobeensis subsp. martinezii		
Cucurbitaceae	Cucurbita pedatifolia		
Cucurbitaceae	Cucurbita pepo		
Cucurbitaceae	Cucurbita radicans	EN	
Cucurbitaceae	Cyclanthera carthagenensis		
Cucurbitaceae	Cyclanthera dissecta		
Cucurbitaceae	Cyclanthera gracillima		VU
Cucurbitaceae	Cyclanthera integrifoliola		
Cucurbitaceae	Cyclanthera langaei		
Cucurbitaceae	Cyclanthera multifoliola		
Cucurbitaceae	Cyclanthera naudiniana		
Cucurbitaceae	Cyclanthera ribiflora		
Cucurbitaceae	Cyclanthera steyermarkii		VU
Cucurbitaceae	Cyclanthera tamnoides		
Cucurbitaceae	Doyerea emetocathartica		
Cucurbitaceae	Echinopepon coulteri		
Cucurbitaceae	Echinopepon milleflorus		
Cucurbitaceae	Echinopepon paniculatus		
Cucurbitaceae	Echinopepon pubescens		
Cucurbitaceae	Echinopepon racemosus		
Cucurbitaceae	Echinopepon torquatus		
Cucurbitaceae	Echinopepon wrightii		
Cucurbitaceae	Gurania makoyana		
Cucurbitaceae	Hanburia mexicana		
Cucurbitaceae	Hanburia parviflora		
Cucurbitaceae	Ibervillea hypoleuca		
Cucurbitaceae	Ibervillea millspaughii		
Cucurbitaceae	Lagenaria siceraria		
Cucurbitaceae	Luffa aegyptiaca		
Cucurbitaceae	Melothria pendula		
Cucurbitaceae	Melothria scabra		
Cucurbitaceae	Melothria trilobata		
Cucurbitaceae	Microsechium compositum		

Cucurbitaceae	Microsechium palmatum		
Cucurbitaceae	Momordica balsamina	VU	
Cucurbitaceae	Momordica charantia		
Cucurbitaceae	Parasicyos dieterleae	VU	
Cucurbitaceae	Parasicyos maculatus	EN	
Cucurbitaceae	Peponopsis adhaerens		
Cucurbitaceae	Polyclathra cucumerina		
Cucurbitaceae	Psiguria triphylla		
Cucurbitaceae	Psiguria warscewiczii		
Cucurbitaceae	Schizocarpum attenuatum	VU	
Cucurbitaceae	Schizocarpum filiforme		
Cucurbitaceae	Schizocarpum liebmannii		
Cucurbitaceae	Schizocarpum longisepalum		
Cucurbitaceae	Schizocarpum palmeri		
Cucurbitaceae	Sechiopsis diptera	EN	
Cucurbitaceae	Sechium chinantlense	VU	
Cucurbitaceae	Sechium mexicanum		
Cucurbitaceae	Sicana odorifera		
Cucurbitaceae	Sicydium davilae	EN	YES
Cucurbitaceae	Sicydium schiedeanum		
Cucurbitaceae	Sicydium synantherum		
Cucurbitaceae	Sicydium tamnifolium		
Cucurbitaceae	Sicydium tuerckheimii		
Cucurbitaceae	Sicyos chiriquensis	VU	
Cucurbitaceae	Sicyos davilae	VU	
Cucurbitaceae	Sicyos edulis		
Cucurbitaceae	Sicyos galeottii		
Cucurbitaceae	Sicyos guatemalensis		
Cucurbitaceae	Sicyos kunthii	EN	
Cucurbitaceae	Sicyos laciniatus		
Cucurbitaceae	Sicyos longisepalus		
Cucurbitaceae	Sicyos microphyllus		
Cucurbitaceae	Sicyos peninsularis		
Cucurbitaceae	Sicyos sertulifer		

Cucurbitaceae	Tecunumania quetzalteca			EN
Cunoniaceae	Weinmannia intermedia		EN	
Cunoniaceae	Weinmannia pinnata			
Cupressaceae	Hesperocyparis benthamii			
Cupressaceae	Hesperocyparis lusitanica			
Cupressaceae	Juniperus comitana		EN	
Cupressaceae	Juniperus deppeana			
Cupressaceae	Juniperus deppeana var. deppeana			
Cupressaceae	Juniperus deppeana var. robusta			
Cupressaceae	Juniperus flaccida			
Cupressaceae	Juniperus gamboana		EN	
Cupressaceae	Juniperus monosperma			
Cupressaceae	Juniperus poblana			VU
Cupressaceae	Platycladus orientalis			
Cupressaceae	Taxodium distichum var. mexicanum			
Cupressaceae	Thuja occidentalis			VU
Cyatheaceae	Alsophila firma	EN		
Cyatheaceae	Alsophila salvinii	NT		
Cyatheaceae	Cibotium schiedei	EN		
Cyatheaceae	Culcita coniifolia	NT		
Cyatheaceae	Cyathea bicrenata	NT		
Cyatheaceae	Cyathea costaricensis	EN		
Cyatheaceae	Cyathea divergens			
Cyatheaceae	Cyathea fulva	NT		
Cyatheaceae	Cyathea godmanii	NT		
Cyatheaceae	Cyathea liebmannii	NT		
Cyatheaceae	Cyathea microdonta			VU
Cyatheaceae	Cyathea schiedeana	NT		
Cyatheaceae	Cyathea tuerckheimii			VU
Cyatheaceae	Dicksonia karsteniana			
Cyatheaceae	Dicksonia sellowiana	NT		
Cyatheaceae	Lophosoria quadripinnata			
Cyatheaceae	Plagiogyria pectinata			
Cyatheaceae	Sphaeropteris horrida	NT		

Cyatheaceae	Sphaeropteris myosuroides		
Cyclanthaceae	Asplundia labela		
Cyclanthaceae	Asplundia liebmannii		
Cyclanthaceae	Asplundia utilis	EN	
Cyclanthaceae	Carludovica drudei	VU	
Cyclanthaceae	Carludovica palmata		
Cyclanthaceae	Cyclanthus bipartitus	EN	
Cyclanthaceae	Dicranopygium gracile		
Cyperaceae	Bulbostylis capillaris		
Cyperaceae	Bulbostylis funckii		
Cyperaceae	Bulbostylis juncoides		
Cyperaceae	Bulbostylis trilobata	EN	
Cyperaceae	Bulbostylis vestita		
Cyperaceae	Calyptrocarya glomerulata		
Cyperaceae	Carex anisostachys		
Cyperaceae	Carex asynchrona	VU	
Cyperaceae	Carex atractodes	VU	
Cyperaceae	Carex aztecica		
Cyperaceae	Carex brevior	EN	
Cyperaceae	Carex caeligena	EN	
Cyperaceae	Carex chordalis		
Cyperaceae	Carex complanata	VU	
Cyperaceae	Carex complanata subsp. tropicalis	EN	
Cyperaceae	Carex cortesii		
Cyperaceae	Carex donnell-smithii		
Cyperaceae	Carex eburnea	CR	
Cyperaceae	Carex evadens	CR	YES
Cyperaceae	Carex flexirostris	EN	
Cyperaceae	Carex fructus	EN	
Cyperaceae	Carex hamata		
Cyperaceae	Carex hultenii	VU	
Cyperaceae	Carex humboldtiana		
Cyperaceae	Carex jamesonii		
Cyperaceae	Carex longii		

Cyperaceae	Carex melanosperma	VU	
Cyperaceae	Carex morrowii	CR	YES
Cyperaceae	Carex muehlenbergii var. xalapensis		
Cyperaceae	Carex peucophila		
Cyperaceae	Carex planostachys		
Cyperaceae	Carex polystachya		
Cyperaceae	Carex polystachya var. bartlettii	EN	
Cyperaceae	Carex polystachya var. polystachya		
Cyperaceae	Carex pringlei		
Cyperaceae	Carex pubigluma	CR	YES
Cyperaceae	Carex spissa		
Cyperaceae	Carex standleyana		
Cyperaceae	Carex thurberi		
Cyperaceae	Carex tuberculata		
Cyperaceae	Carex turbinata		
Cyperaceae	Cladium mariscus	VU	
Cyperaceae	Cladium mariscus subsp. jamaicense		
Cyperaceae	Cyperus aggregatus		
Cyperaceae	Cyperus aggregatus var. aggregatus		
Cyperaceae	Cyperus alternifolius subsp. flabelliformis		
Cyperaceae	Cyperus amabilis		
Cyperaceae	Cyperus articulatus		
Cyperaceae	Cyperus bipartitus		
Cyperaceae	Cyperus blepharoleptos		
Cyperaceae	Cyperus brevifolius		
Cyperaceae	Cyperus brevifolius var. brevifolius		
Cyperaceae	Cyperus camphoratus	VU	
Cyperaceae	Cyperus canus		
Cyperaceae	Cyperus ciliatus		
Cyperaceae	Cyperus compressus		
Cyperaceae	Cyperus conglobatus		
Cyperaceae	Cyperus cuspidatus		
Cyperaceae	Cyperus dentoniae		
Cyperaceae	Cyperus diffusus		

Cyperaceae	Cyperus digitatus	
Cyperaceae	Cyperus elegans	
Cyperaceae	Cyperus entrerianus	
Cyperaceae	Cyperus esculentus	
Cyperaceae	Cyperus filiculmis	EN
Cyperaceae	Cyperus flavescens	
Cyperaceae	Cyperus friburgensis	EN
Cyperaceae	Cyperus giganteus	
Cyperaceae	Cyperus haspan	
Cyperaceae	Cyperus hermaphroditus	
Cyperaceae	Cyperus hortensis	VU
Cyperaceae	Cyperus humilis	
Cyperaceae	Cyperus imbricatus	
Cyperaceae	Cyperus iria	
Cyperaceae	Cyperus ischnos	
Cyperaceae	Cyperus lanceolatus	
Cyperaceae	Cyperus laxus	
Cyperaceae	Cyperus ligularis	
Cyperaceae	Cyperus luzulae	
Cyperaceae	Cyperus manimae	
Cyperaceae	Cyperus manimae var. apiculatus	
Cyperaceae	Cyperus manimae var. manimae	
Cyperaceae	Cyperus matudae	VU
Cyperaceae	Cyperus megalanthus	
Cyperaceae	Cyperus mutisii	
Cyperaceae	Cyperus niger	
Cyperaceae	Cyperus ochraceus	
Cyperaceae	Cyperus odoratus	
Cyperaceae	Cyperus odoratus subsp. odoratus	
Cyperaceae	Cyperus oxylepis	
Cyperaceae	Cyperus polystachyos	
Cyperaceae	Cyperus prolixus	
Cyperaceae	Cyperus pseudovegetus	
Cyperaceae	Cyperus reflexus	

Cyperaceae	Cyperus richardii		
Cyperaceae	Cyperus rotundus		
Cyperaceae	Cyperus seslerioides		
Cyperaceae	Cyperus sesquiflorus		
Cyperaceae	Cyperus spectabilis		
Cyperaceae	Cyperus spectabilis var. spectabilis	CR	YES
Cyperaceae	Cyperus sphaerolepis		
Cyperaceae	Cyperus squarrosus		
Cyperaceae	Cyperus subsquarrosus		
Cyperaceae	Cyperus surinamensis		
Cyperaceae	Cyperus svensonii	VU	
Cyperaceae	Cyperus tenerrimus		
Cyperaceae	Cyperus tenuifolius	VU	
Cyperaceae	Cyperus tenuis		
Cyperaceae	Cyperus tetragonus	VU	
Cyperaceae	Cyperus thyrsiflorus		
Cyperaceae	Cyperus unioloides		
Cyperaceae	Cyperus virens		
Cyperaceae	Cyperus virens var. virens		
Cyperaceae	Cypringlea analecta		
Cyperaceae	Eleocharis acicularis		
Cyperaceae	Eleocharis acicularis var. acicularis	VU	
Cyperaceae	Eleocharis acutangula		
Cyperaceae	Eleocharis acutangula subsp. acutangula		
Cyperaceae	Eleocharis acutangula subsp. breviseta	EN	
Cyperaceae	Eleocharis baldwinii	CR	YES
Cyperaceae	Eleocharis densa		
Cyperaceae	Eleocharis dombeyana		
Cyperaceae	Eleocharis elegans		
Cyperaceae	Eleocharis filiculmis		
Cyperaceae	Eleocharis flavescens		
Cyperaceae	Eleocharis geniculata		
Cyperaceae	Eleocharis interstincta		
Cyperaceae	Eleocharis macrostachya		

Cyperaceae	Eleocharis minima	
Cyperaceae	Eleocharis montana	
Cyperaceae	Eleocharis montevidensis	
Cyperaceae	Eleocharis plicarhachis	VU
Cyperaceae	Eleocharis retroflexa	VU
Cyperaceae	Eleocharis rostellata	
Cyperaceae	Eleocharis schaffneri	
Cyperaceae	Fimbristylis autumnalis	
Cyperaceae	Fimbristylis complanata	
Cyperaceae	Fimbristylis cymosa	
Cyperaceae	Fimbristylis dichotoma	
Cyperaceae	Fimbristylis dichotoma subsp. dichotoma	
Cyperaceae	Fimbristylis littoralis	
Cyperaceae	Fimbristylis mexicana	
Cyperaceae	Fimbristylis simplex	
Cyperaceae	Fimbristylis spadicea	
Cyperaceae	Fuirena camptotricha	
Cyperaceae	Fuirena incompleta	
Cyperaceae	Fuirena robusta	VU
Cyperaceae	Fuirena simplex	
Cyperaceae	Fuirena umbellata	
Cyperaceae	Hypolytrum longifolium subsp. nicaraguense	VU
Cyperaceae	Rhynchospora angosturensis	VU
Cyperaceae	Rhynchospora aristata	
Cyperaceae	Rhynchospora barbata	
Cyperaceae	Rhynchospora blepharophora	
Cyperaceae	Rhynchospora cephalotes	
Cyperaceae	Rhynchospora colorata	
Cyperaceae	Rhynchospora consanguinea	
Cyperaceae	Rhynchospora contracta	
Cyperaceae	Rhynchospora corymbosa	
Cyperaceae	Rhynchospora divergens	EN
Cyperaceae	Rhynchospora dives	
Cyperaceae	Rhynchospora eximia	

Cyperaceae	Rhynchospora floridensis		
Cyperaceae	Rhynchospora gigantea	VU	
Cyperaceae	Rhynchospora globosa		
Cyperaceae	Rhynchospora holoschoenoides		
Cyperaceae	Rhynchospora intermixta	CR	YES
Cyperaceae	Rhynchospora kunthii		
Cyperaceae	Rhynchospora marisculus		
Cyperaceae	Rhynchospora nervosa		
Cyperaceae	Rhynchospora polyphylla		
Cyperaceae	Rhynchospora polystachys	CR	YES
Cyperaceae	Rhynchospora pubera	VU	
Cyperaceae	Rhynchospora pura		
Cyperaceae	Rhynchospora radicans		
Cyperaceae	Rhynchospora radicans subsp. microcephala		
Cyperaceae	Rhynchospora radicans subsp. radicans		
Cyperaceae	Rhynchospora rariflora		
Cyperaceae	Rhynchospora robusta	VU	
Cyperaceae	Rhynchospora rosae	EN	
Cyperaceae	Rhynchospora rugosa		
Cyperaceae	Rhynchospora schiedeana		
Cyperaceae	Rhynchospora scutellata		
Cyperaceae	Rhynchospora tenerrima		
Cyperaceae	Rhynchospora tenerrima subsp. tenerrima		
Cyperaceae	Rhynchospora tenuis		
Cyperaceae	Rhynchospora tenuis subsp. tenuis	VU	
Cyperaceae	Rhynchospora torresiana	CR	YES
Cyperaceae	Rhynchospora tuerckheimii		
Cyperaceae	Rhynchospora vulcani	EN	
Cyperaceae	Rhynchospora watsonii	VU	
Cyperaceae	Schoenus nigricans		
Cyperaceae	Scleria bourgeaui		
Cyperaceae	Scleria bracteata		
Cyperaceae	Scleria colorata	CR	YES
Cyperaceae	Scleria eggersiana		

Cyperaceae	Scleria gaertneri		
Cyperaceae	Scleria hirtella		
Cyperaceae	Scleria induta	EN	
Cyperaceae	Scleria interrupta	VU	
Cyperaceae	Scleria latifolia		
Cyperaceae	Scleria lithosperma		
Cyperaceae	Scleria macrophylla		
Cyperaceae	Scleria microcarpa		
Cyperaceae	Scleria oligantha		
Cyperaceae	Scleria reticularis		
Cyperaceae	Scleria scabriuscula	VU	
Cyperaceae	Scleria secans		
Cyperaceae	Scleria setulosociliata		
Cyperaceae	Scleria sororia		
Cytinaceae	Bdallophytum americanum		
Cytinaceae	Bdallophytum oxylepis		
Dennstaedtiaceae	Dennstaedtia arborescens	EN	
Dennstaedtiaceae	Dennstaedtia bipinnata		
Dennstaedtiaceae	Dennstaedtia cicutaria		
Dennstaedtiaceae	Dennstaedtia cornuta	EN	
Dennstaedtiaceae	Dennstaedtia dissecta		
Dennstaedtiaceae	Dennstaedtia distenta		
Dennstaedtiaceae	Dennstaedtia globulifera		
Dennstaedtiaceae	Dennstaedtia obtusifolia	EN	
Dennstaedtiaceae	Dennstaedtia spinosa	CR	YES
Dennstaedtiaceae	Hypolepis blepharochlaena	VU	
Dennstaedtiaceae	Hypolepis bogotensis	CR	YES
Dennstaedtiaceae	Hypolepis nigrescens		
Dennstaedtiaceae	Hypolepis repens		
Dennstaedtiaceae	Hypolepis trichochlaena	CR	YES
Dennstaedtiaceae	Pteridium aquilinum		
Dennstaedtiaceae	Pteridium arachnoideum		
Dennstaedtiaceae	Pteridium caudatum		
Dennstaedtiaceae	Pteridium feei		

Dennstaedtiaceae	Pteridium pubescens	EN
Dichapetalaceae	Dichapetalum donnell-smithii	
Dichapetalaceae	Dichapetalum donnell-smithii var. chiapasense	
Dilleniaceae	Curatella americana	
Dilleniaceae	Davilla kunthii	
Dilleniaceae	Davilla nitida	
Dilleniaceae	Doliocarpus dentatus	
Dilleniaceae	Tetracera portobellensis	
Dilleniaceae	Tetracera volubilis	
Dilleniaceae	Tetracera volubilis subsp. mollis	
Dioscoreaceae	Dioscorea alata	
Dioscoreaceae	Dioscorea bartlettii	
Dioscoreaceae	Dioscorea bulbifera	
Dioscoreaceae	Dioscorea carionis	
Dioscoreaceae	Dioscorea carpomaculata	
Dioscoreaceae	Dioscorea carpomaculata var. carpomaculata	
Dioscoreaceae	Dioscorea carpomaculata var. cinerea	
Dioscoreaceae	Dioscorea composita	
Dioscoreaceae	Dioscorea convolvulacea	
Dioscoreaceae	Dioscorea convolvulacea subsp. convolvulacea	
Dioscoreaceae	Dioscorea cyanisticta	
Dioscoreaceae	Dioscorea cymosula	
Dioscoreaceae	Dioscorea densiflora	
Dioscoreaceae	Dioscorea dugesii	
Dioscoreaceae	Dioscorea floribunda	
Dioscoreaceae	Dioscorea galeottiana	
Dioscoreaceae	Dioscorea gaumeri	
Dioscoreaceae	Dioscorea gomez-pompae	
Dioscoreaceae	Dioscorea hondurensis	EN
Dioscoreaceae	Dioscorea howardiana	EN
Dioscoreaceae	Dioscorea juxtlahuacensis	VU
Dioscoreaceae	Dioscorea lepida	EN
Dioscoreaceae	Dioscorea liebmannii	
Dioscoreaceae	Dioscorea matagalpensis	

Dioscoreaceae	Dioscorea matudae			
Dioscoreaceae	Dioscorea mexicana			
Dioscoreaceae	Dioscorea multinervis			
Dioscoreaceae	Dioscorea nelsonii			
Dioscoreaceae	Dioscorea oaxacensis			
Dioscoreaceae	Dioscorea orizabensis		VU	
Dioscoreaceae	Dioscorea pallens			
Dioscoreaceae	Dioscorea pilosiuscula			
Dioscoreaceae	Dioscorea plumifera			
Dioscoreaceae	Dioscorea polygonoides			
Dioscoreaceae	Dioscorea racemosa		VU	
Dioscoreaceae	Dioscorea remotiflora			
Dioscoreaceae	Dioscorea spiculiflora			
Dioscoreaceae	Dioscorea subtomentosa			
Dioscoreaceae	Dioscorea sumiderensis			
Dioscoreaceae	Dioscorea triandria			
Dioscoreaceae	Dioscorea urceolata			
Dioscoreaceae	Dioscorea urophylla			
Dioscoreaceae	Dioscorea villosa		EN	
Dipentodontaceae	Perrottetia longistylis			
Dipentodontaceae	Perrottetia ovata			
Droseraceae	Drosera brevifolia		EN	
Ebenaceae	Diospyros acapulcensis		VU	
Ebenaceae	Diospyros acapulcensis subsp. nicaraguensis			
Ebenaceae	Diospyros acapulcensis subsp. verae-crucis			
Ebenaceae	Diospyros aequoris subsp. chutlensis		EN	
Ebenaceae	Diospyros aequoris subsp. tehuantepecensis		EN	
Ebenaceae	Diospyros alisu		CR	YES
Ebenaceae	Diospyros anisandra			
Ebenaceae	Diospyros conzattii	EN		
Ebenaceae	Diospyros juruensis subsp. campechiana			
Ebenaceae	Diospyros kirkii			
Ebenaceae	Diospyros nigra			
Ebenaceae	Diospyros oaxacana			

Ebenaceae	Diospyros palmeri			
Ebenaceae	Diospyros salicifolia			
Ebenaceae	Diospyros virginiana			
Ebenaceae	Diospyros yatesiana			
Ebenaceae	Diospyros yucatanensis			
Ebenaceae	Diospyros yucatanensis subsp. yucatanensis			VU
Elaeocarpaceae	Sloanea ampla			EN
Elaeocarpaceae	Sloanea cruenta		EN	VU
Elaeocarpaceae	Sloanea medusula			
Elaeocarpaceae	Sloanea meianthera			VU
Elaeocarpaceae	Sloanea petenensis			VU
Elaeocarpaceae	Sloanea terniflora	NT		
Elaeocarpaceae	Sloanea tuerckheimii			
Ephedraceae	Ephedra compacta			
Equisetaceae	Equisetum × ferrissii			VU
Equisetaceae	Equisetum × haukeanum			
Equisetaceae	Equisetum giganteum			
Equisetaceae	Equisetum hyemale			
Equisetaceae	Equisetum myriochaetum			
Ericaceae	Agarista mexicana			
Ericaceae	Agarista mexicana var. pinetorum			
Ericaceae	Arbutus bicolor			
Ericaceae	Arbutus tessellata			
Ericaceae	Arbutus xalapensis			
Ericaceae	Arctostaphylos pungens			
Ericaceae	Bejaria aestuans			
Ericaceae	Cavendishia bracteata			
Ericaceae	Cavendishia callista			EN
Ericaceae	Cavendishia laurifolia			
Ericaceae	Chimaphila maculata			
Ericaceae	Chimaphila umbellata			
Ericaceae	Comarostaphylis arbutoides			
Ericaceae	Comarostaphylis discolor	NT		
Ericaceae	Comarostaphylis discolor subsp. rupestris			

Ericaceae	Comarostaphylis glaucescens		
Ericaceae	Comarostaphylis longifolia		
Ericaceae	Comarostaphylis polifolia		
Ericaceae	Comarostaphylis polifolia subsp. minor		
Ericaceae	Comarostaphylis sharpii		EN
Ericaceae	Comarostaphylis spinulosa		
Ericaceae	Gaultheria acuminata		
Ericaceae	Gaultheria erecta		
Ericaceae	Gaultheria myrsinoides		
Ericaceae	Lyonia ferruginea		
Ericaceae	Lyonia squamulosa		
Ericaceae	Macleania insignis		
Ericaceae	Monotropa coccinea		
Ericaceae	Monotropa hypopitys	NT	
Ericaceae	Monotropa uniflora		
Ericaceae	Ornithostaphylos oppositifolia		
Ericaceae	Orthilia secunda		
Ericaceae	Pterospora andromedea		
Ericaceae	Pyrola angustifolia		
Ericaceae	Rhododendron indicum		
Ericaceae	Satyria meiantha		VU
Ericaceae	Satyria panurensis		
Ericaceae	Satyria warszewiczii		
Ericaceae	Sphyrospermum buxifolium		
Ericaceae	Vaccinium breedlovei		EN
Ericaceae	Vaccinium confertum		
Ericaceae	Vaccinium consanguineum		
Ericaceae	Vaccinium cordifolium		
Ericaceae	Vaccinium haematinum		
Ericaceae	Vaccinium kunthianum		
Ericaceae	Vaccinium leucanthum		
Ericaceae	Vaccinium lundellianum		VU
Ericaceae	Vaccinium selerianum		VU
Ericaceae	Vaccinium stenophyllum		

Eriocaulaceae	Eriocaulon benthamii	
Eriocaulaceae	Eriocaulon schiedeanum	VU
Eriocaulaceae	Paepalanthus chiapensis	EN
Erythroxylaceae	Erythroxylum areolatum	
Erythroxylaceae	Erythroxylum guatemalense	
Erythroxylaceae	Erythroxylum havanense	
Erythroxylaceae	Erythroxylum macrophyllum	
Erythroxylaceae	Erythroxylum mexicanum	
Erythroxylaceae	Erythroxylum panamense	
Erythroxylaceae	Erythroxylum rotundifolium	
Euphorbiaceae	Acalypha adenostachya	
Euphorbiaceae	Acalypha alopecuroidea	
Euphorbiaceae	Acalypha arvensis	
Euphorbiaceae	Acalypha botteriana	
Euphorbiaceae	Acalypha chiapensis	
Euphorbiaceae	Acalypha cincta	
Euphorbiaceae	Acalypha costaricensis	
Euphorbiaceae	Acalypha dioica	
Euphorbiaceae	Acalypha diversifolia	
Euphorbiaceae	Acalypha ferdinandi	VU
Euphorbiaceae	Acalypha filipes	
Euphorbiaceae	Acalypha firmula	
Euphorbiaceae	Acalypha flavescens	
Euphorbiaceae	Acalypha fournieri	
Euphorbiaceae	Acalypha fredericii	EN
Euphorbiaceae	Acalypha gracilis	EN
Euphorbiaceae	Acalypha guatemalensis	VU
Euphorbiaceae	Acalypha gummifera	
Euphorbiaceae	Acalypha havanensis	
Euphorbiaceae	Acalypha hispida	
Euphorbiaceae	Acalypha indica	
Euphorbiaceae	Acalypha infesta	VU
Euphorbiaceae	Acalypha langiana	
Euphorbiaceae	Acalypha laxiflora	

Euphorbiaceae	Acalypha leptopoda		
Euphorbiaceae	Acalypha longipes		
Euphorbiaceae	Acalypha longispicata	VU	
Euphorbiaceae	Acalypha macrostachya		
Euphorbiaceae	Acalypha macrostachyoides		
Euphorbiaceae	Acalypha membranacea		
Euphorbiaceae	Acalypha mexicana		
Euphorbiaceae	Acalypha microphylla		
Euphorbiaceae	Acalypha microphylla var. interior		
Euphorbiaceae	Acalypha mollis		
Euphorbiaceae	Acalypha monostachya		
Euphorbiaceae	Acalypha mortoniana	VU	
Euphorbiaceae	Acalypha obscura	VU	
Euphorbiaceae	Acalypha ocymoides		
Euphorbiaceae	Acalypha oligantha		
Euphorbiaceae	Acalypha oligodonta		
Euphorbiaceae	Acalypha oreopola	VU	
Euphorbiaceae	Acalypha persimilis		
Euphorbiaceae	Acalypha phleoides		
Euphorbiaceae	Acalypha polystachya		
Euphorbiaceae	Acalypha pseudalopecuroides		
Euphorbiaceae	Acalypha purpurascens		
Euphorbiaceae	Acalypha retifera	CR	YES
Euphorbiaceae	Acalypha rhombifolia		
Euphorbiaceae	Acalypha schiedeana		
Euphorbiaceae	Acalypha schlechtendaliana		
Euphorbiaceae	Acalypha seleriana		
Euphorbiaceae	Acalypha setosa		
Euphorbiaceae	Acalypha skutchii		
Euphorbiaceae	Acalypha subviscida		
Euphorbiaceae	Acalypha synoica		
Euphorbiaceae	Acalypha tenuicauda	CR	YES
Euphorbiaceae	Acalypha trachyloba		
Euphorbiaceae	Acalypha vagans		

Euphorbiaceae	Acalypha villosa		
Euphorbiaceae	Acalypha wilkesiana		
Euphorbiaceae	Acidocroton madrigalensis	CR	EN
Euphorbiaceae	Adelia barbinervis		
Euphorbiaceae	Adelia oaxacana		
Euphorbiaceae	Adelia vaseyi		
Euphorbiaceae	Alchornea chiapasana	EN	EN
Euphorbiaceae	Alchornea latifolia		
Euphorbiaceae	Argythamnia lottiae		
Euphorbiaceae	Argythamnia moorei		EN
Euphorbiaceae	Astraea lobata		
Euphorbiaceae	Bernardia dodecandra		
Euphorbiaceae	Bernardia heteropilosa	EN	EN
Euphorbiaceae	Bernardia macrocarpa	CR	EN
Euphorbiaceae	Bernardia mexicana		
Euphorbiaceae	Bernardia oblanceolata		VU
Euphorbiaceae	Bernardia yucatanensis		
Euphorbiaceae	Caperonia castaneifolia		
Euphorbiaceae	Caperonia palustris		
Euphorbiaceae	Chiropetalum astroplethos		
Euphorbiaceae	Chiropetalum schiedeanum		
Euphorbiaceae	Cleidion castaneifolium		
Euphorbiaceae	Cnidoscolus aconitifolius		
Euphorbiaceae	Cnidoscolus aconitifolius subsp. aconitifolius		
Euphorbiaceae	Cnidoscolus aconitifolius subsp. polyanthus		EN
Euphorbiaceae	Cnidoscolus albibracteatus		EN
Euphorbiaceae	Cnidoscolus albidus		
Euphorbiaceae	Cnidoscolus megacanthus		
Euphorbiaceae	Cnidoscolus multilobus		
Euphorbiaceae	Cnidoscolus multilobus subsp. hirtiflorus		
Euphorbiaceae	Cnidoscolus multilobus subsp. multilobus		
Euphorbiaceae	Cnidoscolus rostratus subsp. glabratus		
Euphorbiaceae	Cnidoscolus rotundifolius		
Euphorbiaceae	Cnidoscolus souzae		

Euphorbiaceae	Cnidoscolus spinosus		VU		
Euphorbiaceae	Cnidoscolus tubulosus				
Euphorbiaceae	Cnidoscolus urens				
Euphorbiaceae	Codiaeum variegatum				
Euphorbiaceae	Croton adspersus				
Euphorbiaceae	Croton amphileucus			EN	
Euphorbiaceae	Croton arboreus				
Euphorbiaceae	Croton argenteus				
Euphorbiaceae	Croton billbergianus				
Euphorbiaceae	Croton bracteatus		EN	CR	YES
Euphorbiaceae	Croton breedlovei			EN	
Euphorbiaceae	Croton brevipes			CR	
Euphorbiaceae	Croton carpostellatus			CR	YES
Euphorbiaceae	Croton chamelensis			VU	
Euphorbiaceae	Croton ciliatoglandulifer				
Euphorbiaceae	Croton conspurcatus			EN	
Euphorbiaceae	Croton cortesianus				
Euphorbiaceae	Croton decalobus			VU	
Euphorbiaceae	Croton dioicus				
Euphorbiaceae	Croton draco				
Euphorbiaceae	Croton ehrenbergii				
Euphorbiaceae	Croton flavens				
Euphorbiaceae	Croton fragilis				
Euphorbiaceae	Croton francoanus				
Euphorbiaceae	Croton fruticulosus				
Euphorbiaceae	Croton glabellus				
Euphorbiaceae	Croton glandulosepalus				
Euphorbiaceae	Croton glandulosus				
Euphorbiaceae	Croton glandulosus var. glandulosus			VU	
Euphorbiaceae	Croton gomezii				
Euphorbiaceae	Croton grewiifolius				
Euphorbiaceae	Croton guatemalensis	NT			
Euphorbiaceae	Croton hirtus				
Euphorbiaceae	Croton humilis				

Euphorbiaceae	Croton hypoleucus			
Euphorbiaceae	Croton incanus			
Euphorbiaceae	Croton leucophyllus			
Euphorbiaceae	Croton liebmannii			
Euphorbiaceae	Croton macrodontus			
Euphorbiaceae	Croton meissneri		EN	
Euphorbiaceae	Croton mexicanus	VU		
Euphorbiaceae	Croton miradorensis			
Euphorbiaceae	Croton monanthogynus			
Euphorbiaceae	Croton morifolius			
Euphorbiaceae	Croton nitens			
Euphorbiaceae	Croton niveus			
Euphorbiaceae	Croton oerstedianus			
Euphorbiaceae	Croton origanifolius		CR	YES
Euphorbiaceae	Croton pagiveteris		EN	
Euphorbiaceae	Croton pedicellatus			
Euphorbiaceae	Croton pendens		EN	
Euphorbiaceae	Croton pseudoniveus			
Euphorbiaceae	Croton ramillatus			
Euphorbiaceae	Croton reflexifolius			
Euphorbiaceae	Croton repens			
Euphorbiaceae	Croton rosarianus		CR	YES
Euphorbiaceae	Croton schiedeanus			
Euphorbiaceae	Croton siltepecensis		EN	
Euphorbiaceae	Croton soliman			
Euphorbiaceae	Croton sonorae			
Euphorbiaceae	Croton sousae	VU	EN	
Euphorbiaceae	Croton stipulaceus			
Euphorbiaceae	Croton suberosus			
Euphorbiaceae	Croton subfragilis		EN	
Euphorbiaceae	Croton sulcifructus	VU	EN	
Euphorbiaceae	Croton tabascensis		VU	
Euphorbiaceae	Croton trinitatis			
Euphorbiaceae	Croton watsonii			

Euphorbiaceae	Croton xalapensis		
Euphorbiaceae	Croton yucatanensis		
Euphorbiaceae	Dalechampia cissifolia		
Euphorbiaceae	Dalechampia dioscoreifolia		VU
Euphorbiaceae	Dalechampia magnistipulata		
Euphorbiaceae	Dalechampia scandens		
Euphorbiaceae	Dalechampia spathulata		
Euphorbiaceae	Dalechampia tiliifolia		
Euphorbiaceae	Dalembertia hahniana		EN
Euphorbiaceae	Dalembertia populifolia		
Euphorbiaceae	Dalembertia triangularis		
Euphorbiaceae	Ditaxis guatemalensis		
Euphorbiaceae	Ditaxis manzanilloana		
Euphorbiaceae	Ditaxis pringlei		
Euphorbiaceae	Euphorbia adenoptera		
Euphorbiaceae	Euphorbia antisyphilitica		
Euphorbiaceae	Euphorbia anychioides		
Euphorbiaceae	Euphorbia ariensis		
Euphorbiaceae	Euphorbia arizonica		
Euphorbiaceae	Euphorbia berteroana		
Euphorbiaceae	Euphorbia calcarata		
Euphorbiaceae	Euphorbia capitellata		
Euphorbiaceae	Euphorbia chiapensis		VU
Euphorbiaceae	Euphorbia colletioides		
Euphorbiaceae	Euphorbia cotinifolia		
Euphorbiaceae	Euphorbia cumbrae		
Euphorbiaceae	Euphorbia cuphosperma		
Euphorbiaceae	Euphorbia cyri	EN	VU
Euphorbiaceae	Euphorbia delicatula		
Euphorbiaceae	Euphorbia densiflora		
Euphorbiaceae	Euphorbia dentata		
Euphorbiaceae	Euphorbia dioeca		
Euphorbiaceae	Euphorbia dioscoreoides		
Euphorbiaceae	Euphorbia esuliformis		

Euphorbiaceae	Euphorbia fimbrilligera		
Euphorbiaceae	Euphorbia finkii	VU	VU
Euphorbiaceae	Euphorbia francoana		
Euphorbiaceae	Euphorbia furcillata		
Euphorbiaceae	Euphorbia graminea		
Euphorbiaceae	Euphorbia graminea var. graminea		
Euphorbiaceae	Euphorbia greggii		
Euphorbiaceae	Euphorbia guatemalensis		VU
Euphorbiaceae	Euphorbia guiengola		
Euphorbiaceae	Euphorbia heterophylla		
Euphorbiaceae	Euphorbia heterophylla var. cyathophora		
Euphorbiaceae	Euphorbia hirta		
Euphorbiaceae	Euphorbia hormorrhiza		VU
Euphorbiaceae	Euphorbia humayensis		
Euphorbiaceae	Euphorbia hypericifolia		
Euphorbiaceae	Euphorbia hyssopifolia		
Euphorbiaceae	Euphorbia indivisa		
Euphorbiaceae	Euphorbia ixtlana		
Euphorbiaceae	Euphorbia jaliscensis		
Euphorbiaceae	Euphorbia lacera		
Euphorbiaceae	Euphorbia lancifolia		
Euphorbiaceae	Euphorbia lasiocarpa		
Euphorbiaceae	Euphorbia leucocephala		
Euphorbiaceae	Euphorbia lucii-smithii		
Euphorbiaceae	Euphorbia lundelliana		VU
Euphorbiaceae	Euphorbia macropus		
Euphorbiaceae	Euphorbia maculata		
Euphorbiaceae	Euphorbia marginata		
Euphorbiaceae	Euphorbia mendezii		
Euphorbiaceae	Euphorbia micromera		
Euphorbiaceae	Euphorbia milii		
Euphorbiaceae	Euphorbia milii var. splendens		VU
Euphorbiaceae	Euphorbia nutans		
Euphorbiaceae	Euphorbia oaxacana		

Euphorbiaceae	Euphorbia ocymoidea	
Euphorbiaceae	Euphorbia ophthalmica	
Euphorbiaceae	Euphorbia orizabae	
Euphorbiaceae	Euphorbia parviflora	VU
Euphorbiaceae	Euphorbia peganoides	
Euphorbiaceae	Euphorbia peplus	
Euphorbiaceae	Euphorbia picachensis	EN
Euphorbiaceae	Euphorbia potosina	
Euphorbiaceae	Euphorbia prostrata	
Euphorbiaceae	Euphorbia pseudofulva	VU
Euphorbiaceae	Euphorbia pteroneura	
Euphorbiaceae	Euphorbia pueblensis	EN
Euphorbiaceae	Euphorbia pulcherrima	
Euphorbiaceae	Euphorbia radians	
Euphorbiaceae	Euphorbia rossiana	
Euphorbiaceae	Euphorbia scandens	
Euphorbiaceae	Euphorbia schiedeana	
Euphorbiaceae	Euphorbia schlechtendalii	
Euphorbiaceae	Euphorbia schlechtendalii var. schlechtendalii	
Euphorbiaceae	Euphorbia schlechtendalii var. websteri	
Euphorbiaceae	Euphorbia sciadophila	CR
Euphorbiaceae	Euphorbia segoviensis	
Euphorbiaceae	Euphorbia serpens	
Euphorbiaceae	Euphorbia serpillifolia	
Euphorbiaceae	Euphorbia sinclairiana	VU
Euphorbiaceae	Euphorbia sphaerorhiza	
Euphorbiaceae	Euphorbia splendens	
Euphorbiaceae	Euphorbia stictospora	
Euphorbiaceae	Euphorbia subpeltata	
Euphorbiaceae	Euphorbia tanquahuete	
Euphorbiaceae	Euphorbia thymifolia	
Euphorbiaceae	Euphorbia tirucalli	
Euphorbiaceae	Euphorbia tithymaloides	
Euphorbiaceae	Euphorbia tithymaloides subsp. tithymaloides	

Euphorbiaceae	Euphorbia tricolor		
Euphorbiaceae	Euphorbia triphylla		
Euphorbiaceae	Euphorbia velleriflora		
Euphorbiaceae	Euphorbia vestita		VU
Euphorbiaceae	Euphorbia villifera		
Euphorbiaceae	Euphorbia whitei		
Euphorbiaceae	Euphorbia xalapensis		
Euphorbiaceae	Garcia nutans	EN	
Euphorbiaceae	Garcia parviflora		
Euphorbiaceae	Gymnanthes actinostemoides		
Euphorbiaceae	Gymnanthes longipes	EN	
Euphorbiaceae	Gymnanthes riparia		
Euphorbiaceae	Hevea brasiliensis		
Euphorbiaceae	Hippomane mancinella		
Euphorbiaceae	Hura polyandra		
Euphorbiaceae	Jatropha alamanii	VU	
Euphorbiaceae	Jatropha ciliata		
Euphorbiaceae	Jatropha curcas		
Euphorbiaceae	Jatropha dioica		
Euphorbiaceae	Jatropha gaumeri		
Euphorbiaceae	Jatropha gossypiifolia		
Euphorbiaceae	Jatropha integerrima		
Euphorbiaceae	Jatropha malacophylla		
Euphorbiaceae	Jatropha oaxacana		
Euphorbiaceae	Jatropha ortegae	EN	VU
Euphorbiaceae	Jatropha peltata		
Euphorbiaceae	Jatropha podagrica		
Euphorbiaceae	Jatropha pseudocurcas		
Euphorbiaceae	Jatropha sotoi-nunyezii	EN	EN
Euphorbiaceae	Jatropha standleyi	VU	
Euphorbiaceae	Jatropha tehuantepecana	EN	EN
Euphorbiaceae	Mabea excelsa		VU
Euphorbiaceae	Mabea occidentalis		
Euphorbiaceae	Manihot aesculifolia		

Euphorbiaceae	Manihot angustiloba				
Euphorbiaceae	Manihot chlorosticta				
Euphorbiaceae	Manihot esculenta				
Euphorbiaceae	Manihot foetida				
Euphorbiaceae	Manihot oaxacana				
Euphorbiaceae	Manihot pringlei				
Euphorbiaceae	Manihot rhomboidea				
Euphorbiaceae	Manihot rhomboidea subsp. microcarpa				
Euphorbiaceae	Manihot rubricaulis				
Euphorbiaceae	Manihot triloba			VU	
Euphorbiaceae	Omphalea oleifera				
Euphorbiaceae	Ophellantha spinosa				
Euphorbiaceae	Ophellantha steyermarkii			EN	
Euphorbiaceae	Pleradenophora tikalana		VU		
Euphorbiaceae	Pleradenophora tuerckheimiana				
Euphorbiaceae	Plukenetia carabiasiae			EN	YES
Euphorbiaceae	Plukenetia penninervia				
Euphorbiaceae	Plukenetia stipellata			EN	
Euphorbiaceae	Plukenetia volubilis			VU	
Euphorbiaceae	Ricinus communis				
Euphorbiaceae	Sapium appendiculatum				
Euphorbiaceae	Sapium glandulosum				
Euphorbiaceae	Sapium lateriflorum				
Euphorbiaceae	Sapium macrocarpum	VU			
Euphorbiaceae	Sebastiania cruenta				
Euphorbiaceae	Sebastiania glandulosa				
Euphorbiaceae	Sebastiania hintonii		VU		
Euphorbiaceae	Sebastiania pavoniana				
Euphorbiaceae	Stillingia acutifolia				
Euphorbiaceae	Stillingia diphtherina			VU	
Euphorbiaceae	Stillingia sanguinolenta				
Euphorbiaceae	Stillingia zelayensis				
Euphorbiaceae	Tetrorchidium brevifolium				
Euphorbiaceae	Tetrorchidium rotundatum	VU			

Euphorbiaceae	Tragia cordata	EN
Euphorbiaceae	Tragia glanduligera	
Euphorbiaceae	Tragia mexicana	
Euphorbiaceae	Tragia nepetifolia	
Euphorbiaceae	Tragia potosina	EN
Euphorbiaceae	Tragia ramosa	
Euphorbiaceae	Tragia volubilis	
Euphorbiaceae	Tragia yucatanensis	
Euphorbiaceae	Triadica sebifera	
Euphorbiaceae	Zuckertia cordata	
Fabaceae	Abarema idiopoda	
Fabaceae	Abarema zolleriana	
Fabaceae	Acacia floribunda	
Fabaceae	Acacia neriifolia	EN
Fabaceae	Acaciella angustissima	
Fabaceae	Acaciella angustissima var. angustissima	
Fabaceae	Acaciella angustissima var. filicioides	
Fabaceae	Acaciella angustissima var. texensis	
Fabaceae	Acaciella bicolor	
Fabaceae	Acaciella chamelensis	EN
Fabaceae	Acaciella goldmanii	
Fabaceae	Acaciella hartwegii	
Fabaceae	Acaciella tequilana	
Fabaceae	Acaciella tequilana var. tequilana	
Fabaceae	Acaciella villosa	
Fabaceae	Adenopodia oaxacana	
Fabaceae	Adenopodia patens	
Fabaceae	Aeschynomene americana	
Fabaceae	Aeschynomene americana var. flabellata	
Fabaceae	Aeschynomene brasiliana	
Fabaceae	Aeschynomene compacta	
Fabaceae	Aeschynomene elegans	
Fabaceae	Aeschynomene fascicularis	
Fabaceae	Aeschynomene nicaraguensis	

Fabaceae	Aeschynomene palmeri		VU
Fabaceae	Aeschynomene paniculata		
Fabaceae	Aeschynomene paucifoliolata		
Fabaceae	Aeschynomene petraea		
Fabaceae	Aeschynomene pinetorum		
Fabaceae	Aeschynomene rudis		
Fabaceae	Aeschynomene scabra		
Fabaceae	Aeschynomene sensitiva		
Fabaceae	Aeschynomene villosa		
Fabaceae	Aeschynomene villosa var. mexicana		
Fabaceae	Aeschynomene villosa var. villosa		
Fabaceae	Albizia adinocephala		
Fabaceae	Albizia canescens		
Fabaceae	Albizia carbonaria		EN
Fabaceae	Albizia lebbeck		
Fabaceae	Albizia niopoides var. niopoides		
Fabaceae	Albizia occidentalis		
Fabaceae	Albizia purpusii		
Fabaceae	Albizia tomentosa		
Fabaceae	Albizia xerophytica	EN	EN
Fabaceae	Alysicarpus vaginalis		
Fabaceae	Amicia zygomeris		
Fabaceae	Amphicarpaea bracteata		VU
Fabaceae	Ancistrotropis peduncularis		
Fabaceae	Andira galeottiana	VU	
Fabaceae	Andira inermis		
Fabaceae	Andira inermis subsp. inermis		
Fabaceae	Apoplanesia paniculata		
Fabaceae	Arachis hypogaea		
Fabaceae	Arachis pintoi		VU
Fabaceae	Astragalus guatemalensis		
Fabaceae	Astragalus helleri		
Fabaceae	Astragalus hypoleucus		
Fabaceae	Astragalus micranthus		

Fabaceae	Astragalus mollissimus		
Fabaceae	Astragalus oxyrhynchus		
Fabaceae	Astragalus strigulosus		
Fabaceae	Astragalus wootonii		
Fabaceae	Ateleia albolutescens	EN	
Fabaceae	Ateleia chicoasensis	EN	EN
Fabaceae	Ateleia glabrata	CR	EN
Fabaceae	Ateleia hexandra	EN	EN
Fabaceae	Ateleia pterocarpa		
Fabaceae	Ateleia tenorioi	EN	VU
Fabaceae	Balizia leucocalyx		
Fabaceae	Barbieria pinnata		VU
Fabaceae	Bauhinia andrieuxii		
Fabaceae	Bauhinia bartlettii	EN	VU
Fabaceae	Bauhinia chapulhuacania		
Fabaceae	Bauhinia cookii		
Fabaceae	Bauhinia coulteri		
Fabaceae	Bauhinia deserti		
Fabaceae	Bauhinia dipetala		
Fabaceae	Bauhinia divaricata		
Fabaceae	Bauhinia forficata		
Fabaceae	Bauhinia jenningsii		
Fabaceae	Bauhinia lunarioides		
Fabaceae	Bauhinia macranthera		
Fabaceae	Bauhinia miriamae		EN
Fabaceae	Bauhinia monandra		
Fabaceae	Bauhinia pansamalana		VU
Fabaceae	Bauhinia pauletia		
Fabaceae	Bauhinia purpurea		
Fabaceae	Bauhinia ramosissima		
Fabaceae	Bauhinia retifolia	EN	VU
Fabaceae	Bauhinia rubeleruziana		
Fabaceae	Bauhinia seleriana		
Fabaceae	Bauhinia subrotundifolia		

Fabaceae	Bauhinia ungulata			
Fabaceae	Bauhinia variegata			
Fabaceae	Bauhinia wunderlinii		EN	
Fabaceae	Brongniartia bracteolata			
Fabaceae	Brongniartia foliolosa			
Fabaceae	Brongniartia glabrata			
Fabaceae	Brongniartia intermedia			
Fabaceae	Brongniartia lupinoides			
Fabaceae	Brongniartia magnibracteata			
Fabaceae	Brongniartia mollis			
Fabaceae	Brongniartia sericea		EN	
Fabaceae	Brongniartia sousae			
Fabaceae	Brongniartia stipitata		CR	YES
Fabaceae	Caesalpinia pringlei			
Fabaceae	Caesalpinia pulcherrima			
Fabaceae	Cajanus acutifolius			
Fabaceae	Cajanus cajan			
Fabaceae	Calliandra caeciliae			
Fabaceae	Calliandra conferta			
Fabaceae	Calliandra eriophylla			
Fabaceae	Calliandra grandifolia	EN		
Fabaceae	Calliandra hirsuta			
Fabaceae	Calliandra houstoniana			
Fabaceae	Calliandra houstoniana var. acapulcensis			
Fabaceae	Calliandra houstoniana var. anomala			
Fabaceae	Calliandra houstoniana var. calothyrsus			
Fabaceae	Calliandra humilis			
Fabaceae	Calliandra humilis var. reticulata			
Fabaceae	Calliandra juzepczukii			
Fabaceae	Calliandra magdalenae			
Fabaceae	Calliandra magdalenae var. colombiana			
Fabaceae	Calliandra ricoana	CR	EN	YES
Fabaceae	Calliandra rubescens			
Fabaceae	Calliandra tergemina			

Fabaceae	Calliandra tergemina var. emarginata		
Fabaceae	Calliandra trinervia		VU
Fabaceae	Calliandra trinervia var. arborea	VU	
Fabaceae	Calopogonium caeruleum		
Fabaceae	Calopogonium galactioides		
Fabaceae	Calopogonium lanceolatum		
Fabaceae	Calopogonium mucunoides		
Fabaceae	Canavalia acuminata		
Fabaceae	Canavalia bonariensis		EN
Fabaceae	Canavalia brasiliensis		
Fabaceae	Canavalia dura		
Fabaceae	Canavalia ensiformis		
Fabaceae	Canavalia glabra		
Fabaceae	Canavalia hirsutissima		
Fabaceae	Canavalia matudae		EN
Fabaceae	Canavalia oxyphylla		
Fabaceae	Canavalia palmeri		
Fabaceae	Canavalia rosea		
Fabaceae	Canavalia septentrionalis		
Fabaceae	Canavalia villosa		
Fabaceae	Cassia fistula		
Fabaceae	Cassia grandis		
Fabaceae	Cassia hintonii		
Fabaceae	Cassia moschata		
Fabaceae	Cenostigma eriostachys		
Fabaceae	Centrosema macrocarpum		
Fabaceae	Centrosema molle		
Fabaceae	Centrosema plumieri		
Fabaceae	Centrosema pubescens		
Fabaceae	Centrosema sagittatum		
Fabaceae	Centrosema schottii		
Fabaceae	Centrosema unifoliatum		VU
Fabaceae	Centrosema virginianum		
Fabaceae	Cercis canadensis		

Fabaceae	Cercis canadensis subsp. mexicana		
Fabaceae	Cercis canadensis subsp. texensis		
Fabaceae	Chamaecrista absus var. meonandra		
Fabaceae	Chamaecrista aurivilla	CR	YES
Fabaceae	Chamaecrista chamaecristoides		
Fabaceae	Chamaecrista diphylla		
Fabaceae	Chamaecrista flexuosa		
Fabaceae	Chamaecrista glandulosa		
Fabaceae	Chamaecrista glandulosa var. flavicoma		
Fabaceae	Chamaecrista glandulosa var. tristicula	VU	
Fabaceae	Chamaecrista greggii		
Fabaceae	Chamaecrista hispidula		
Fabaceae	Chamaecrista kunthiana		
Fabaceae	Chamaecrista nictitans		
Fabaceae	Chamaecrista nictitans subsp. disadena		
Fabaceae	Chamaecrista nictitans subsp. patellaria		
Fabaceae	Chamaecrista nictitans var. glabrata		
Fabaceae	Chamaecrista nictitans var. jaliscensis		
Fabaceae	Chamaecrista nictitans var. paraguariensis		
Fabaceae	Chamaecrista nictitans var. pilosa		
Fabaceae	Chamaecrista punctata	EN	
Fabaceae	Chamaecrista rotundifolia		
Fabaceae	Chamaecrista rufa		
Fabaceae	Chamaecrista serpens		
Fabaceae	Chamaecrista serpens var. grandiflora	CR	
Fabaceae	Chamaecrista serpens var. wrightii		
Fabaceae	Chapmannia prismatica		
Fabaceae	Chloroleucon mangense		
Fabaceae	Chloroleucon mangense var. leucospermum		
Fabaceae	Clitoria falcata		
Fabaceae	Clitoria guianensis		
Fabaceae	Clitoria mexicana		
Fabaceae	Clitoria polystachya		
Fabaceae	Clitoria ternatea		

Fabaceae	Cochliasanthus caracalla		
Fabaceae	Cojoba arborea		
Fabaceae	Cojoba costaricensis		CR
Fabaceae	Cojoba escuintlensis		
Fabaceae	Cojoba graciliflora		
Fabaceae	Cojoba sophorocarpa		
Fabaceae	Cologania angustifolia		
Fabaceae	Cologania biloba		
Fabaceae	Cologania broussonetii		
Fabaceae	Cologania grandiflora		
Fabaceae	Cologania hirta		
Fabaceae	Cologania obovata		
Fabaceae	Cologania procumbens		
Fabaceae	Condylostylis latidenticulata		EN
Fabaceae	Conzattia chiapensis		EN
Fabaceae	Conzattia multiflora		
Fabaceae	Coulteria glabra	VU	EN
Fabaceae	Coulteria platyloba		
Fabaceae	Coulteria velutina		
Fabaceae	Coursetia caribaea		
Fabaceae	Coursetia caribaea var. chiapensis		VU
Fabaceae	Coursetia chiapensis		EN
Fabaceae	Coursetia glandulosa		
Fabaceae	Coursetia greenmanii		
Fabaceae	Coursetia mollis		
Fabaceae	Coursetia polyphylla		
Fabaceae	Coursetia pumila		
Fabaceae	Crotalaria angulata		
Fabaceae	Crotalaria bupleurifolia		
Fabaceae	Crotalaria bupleurifolia var. robusta		
Fabaceae	Crotalaria cajanifolia		
Fabaceae	Crotalaria filifolia		
Fabaceae	Crotalaria incana		
Fabaceae	Crotalaria longirostrata		

Fabaceae	Crotalaria maypurensis				
Fabaceae	Crotalaria micans				
Fabaceae	Crotalaria mollicula				
Fabaceae	Crotalaria nayaritensis				
Fabaceae	Crotalaria nitens				
Fabaceae	Crotalaria polyphylla				
Fabaceae	Crotalaria pumila				
Fabaceae	Crotalaria purshii			VU	
Fabaceae	Crotalaria quercetorum				
Fabaceae	Crotalaria rotundifolia				
Fabaceae	Crotalaria sagittalis				
Fabaceae	Crotalaria schiedeana				
Fabaceae	Crotalaria spectabilis				
Fabaceae	Crotalaria stipularia			CR	YES
Fabaceae	Crotalaria vitellina				
Fabaceae	Ctenodon elegans var. elegans			EN	
Fabaceae	Cynometra bauhiniifolia			CR	
Fabaceae	Cynometra hemitomophylla			EN	
Fabaceae	Cynometra oaxacana				
Fabaceae	Cynometra retusa				
Fabaceae	Dalbergia calderonii	EN	CR	VU	
Fabaceae	Dalbergia calycina	VU	VU		
Fabaceae	Dalbergia congestiflora	EN	EN		
Fabaceae	Dalbergia glabra				
Fabaceae	Dalbergia glabra var. paucifoliolata			EN	
Fabaceae	Dalbergia glomerata	NT	CR		
Fabaceae	Dalbergia granadillo	EN	CR		
Fabaceae	Dalbergia melanocardium	EN	EN		
Fabaceae	Dalbergia palo-escrito	VU	EN		
Fabaceae	Dalbergia stevensonii	EN	CR	VU	
Fabaceae	Dalbergia tucurensis	EN	EN		
Fabaceae	Dalea ayavacensis			CR	YES
Fabaceae	Dalea bicolor				
Fabaceae	Dalea bicolor var. naviculifolia			EN	

Fabaceae	Dalea botterii	
Fabaceae	Dalea caeciliae	
Fabaceae	Dalea capitata	
Fabaceae	Dalea carthagenensis	
Fabaceae	Dalea carthagenensis var. barbata	VU
Fabaceae	Dalea carthagenensis var. capitulata	
Fabaceae	Dalea cliffortiana	
Fabaceae	Dalea confusa	VU
Fabaceae	Dalea dorycnioides	
Fabaceae	Dalea elata	
Fabaceae	Dalea exserta	
Fabaceae	Dalea filiciformis	
Fabaceae	Dalea foliolosa	
Fabaceae	Dalea foliolosa var. citrina	
Fabaceae	Dalea greggii	
Fabaceae	Dalea humilis	
Fabaceae	Dalea insignis	
Fabaceae	Dalea leporina	
Fabaceae	Dalea leucosericea	
Fabaceae	Dalea lutea	
Fabaceae	Dalea lutea var. gigantea	
Fabaceae	Dalea melantha	
Fabaceae	Dalea melantha var. berlandieri	
Fabaceae	Dalea minutifolia	
Fabaceae	Dalea pectinata	
Fabaceae	Dalea purpurea	VU
Fabaceae	Dalea quercetorum	VU
Fabaceae	Dalea scandens	
Fabaceae	Dalea scandens var. paucifolia	
Fabaceae	Dalea scandens var. vulneraria	EN
Fabaceae	Dalea sericea	
Fabaceae	Dalea tomentosa	
Fabaceae	Dalea versicolor	
Fabaceae	Dalea versicolor var. involuta	

Fabaceae	Dalea virgata	
Fabaceae	Dalea zimapanica	
Fabaceae	Delonix regia	
Fabaceae	Dermatophyllum secundiflorum	
Fabaceae	Desmanthus bicornutus	
Fabaceae	Desmanthus leptophyllus	
Fabaceae	Desmanthus paspalaceus	EN
Fabaceae	Desmanthus pubescens	
Fabaceae	Desmanthus virgatus	
Fabaceae	Desmodium affine	
Fabaceae	Desmodium alamanii	
Fabaceae	Desmodium amplifolium	
Fabaceae	Desmodium angustifolium	
Fabaceae	Desmodium axillare	
Fabaceae	Desmodium cajanifolium	
Fabaceae	Desmodium callilepis	
Fabaceae	Desmodium caripense	
Fabaceae	Desmodium chiapense	VU
Fabaceae	Desmodium cinereum	
Fabaceae	Desmodium conzattii	
Fabaceae	Desmodium cordistipulum	
Fabaceae	Desmodium distortum	
Fabaceae	Desmodium foliosum	EN
Fabaceae	Desmodium glabrum	
Fabaceae	Desmodium grahamii	
Fabaceae	Desmodium guadalajaranum	
Fabaceae	Desmodium hartwegianum	
Fabaceae	Desmodium helleri	
Fabaceae	Desmodium incanum	
Fabaceae	Desmodium infractum	
Fabaceae	Desmodium intortum	
Fabaceae	Desmodium jaliscanum	
Fabaceae	Desmodium lindheimeri	
Fabaceae	Desmodium lupulinum	EN

Fabaceae	Desmodium macrodesmum		
Fabaceae	Desmodium macrostachyum		
Fabaceae	Desmodium maxonii		
Fabaceae	Desmodium metallicum		
Fabaceae	Desmodium molliculum		
Fabaceae	Desmodium monticola		EN
Fabaceae	Desmodium nicaraguense		
Fabaceae	Desmodium nitidum		
Fabaceae	Desmodium orbiculare		
Fabaceae	Desmodium orizabanum		EN
Fabaceae	Desmodium palmeri		
Fabaceae	Desmodium paniculatum		
Fabaceae	Desmodium plicatum		
Fabaceae	Desmodium polystachyum		
Fabaceae	Desmodium prehensile		
Fabaceae	Desmodium pringlei		
Fabaceae	Desmodium procumbens		
Fabaceae	Desmodium procumbens var. neomexicanum		
Fabaceae	Desmodium procumbens var. transversum		
Fabaceae	Desmodium pseudoamplifolium	CR	EN
Fabaceae	Desmodium psilophyllum		
Fabaceae	Desmodium purpusii		
Fabaceae	Desmodium raymundoramirezii		EN
Fabaceae	Desmodium retinens		
Fabaceae	Desmodium scorpiurus		
Fabaceae	Desmodium sericophyllum		
Fabaceae	Desmodium sericophyllum var. strobilaceum		
Fabaceae	Desmodium skinneri		
Fabaceae	Desmodium subsessile		
Fabaceae	Desmodium sumichrastii		
Fabaceae	Desmodium tortuosum		
Fabaceae	Desmodium uncinatum		
Fabaceae	Desmodium urarioides		
Fabaceae	Desmodium venustum		VU

Fabaceae	Desmodium wydlerianum		EN
Fabaceae	Dialium guianense		
Fabaceae	Dioclea wilsonii		
Fabaceae	Diphysa americana		
Fabaceae	Diphysa carthagenensis		
Fabaceae	Diphysa floribunda		
Fabaceae	Diphysa humilis		
Fabaceae	Diphysa minutifolia		
Fabaceae	Diphysa occidentalis		
Fabaceae	Diphysa ormocarpoides		
Fabaceae	Diphysa puberulenta		
Fabaceae	Diphysa punctata		
Fabaceae	Diphysa racemosa		
Fabaceae	Diphysa sennoides		
Fabaceae	Diphysa spinosa		
Fabaceae	Diphysa suberosa		
Fabaceae	Diphysa yucatanensis		
Fabaceae	Dussia cuscatlanica		VU
Fabaceae	Dussia mexicana		
Fabaceae	Ebenopsis ebano		
Fabaceae	Entada phaseoloides		EN
Fabaceae	Entada polystachya		
Fabaceae	Enterolobium cyclocarpum		
Fabaceae	Enterolobium schomburgkii	VU	
Fabaceae	Eriosema crinitum		
Fabaceae	Eriosema diffusum		
Fabaceae	Eriosema grandiflorum		
Fabaceae	Eriosema multiflorum		
Fabaceae	Eriosema pulchellum		
Fabaceae	Erythrina americana		
Fabaceae	Erythrina berenices		
Fabaceae	Erythrina berteroana		
Fabaceae	Erythrina caribaea		
Fabaceae	Erythrina chiapasana		

Fabaceae	Erythrina cobanensis	EN	CR	YES
Fabaceae	Erythrina florenciae		EN	
Fabaceae	Erythrina folkersii			
Fabaceae	Erythrina goldmanii			
Fabaceae	Erythrina herbacea			
Fabaceae	Erythrina horrida			
Fabaceae	Erythrina lanata			
Fabaceae	Erythrina lanata subsp. calvescens		VU	
Fabaceae	Erythrina leptorhiza			
Fabaceae	Erythrina macrophylla		EN	
Fabaceae	Erythrina mexicana			
Fabaceae	Erythrina nigrorosea			
Fabaceae	Erythrina pudica		VU	
Fabaceae	Erythrina rubrinervia		CR	YES
Fabaceae	Erythrina salviiflora	EN	EN	
Fabaceae	Erythrina sousae		VU	
Fabaceae	Erythrina standleyana			
Fabaceae	Erythrina tuxtlana	VU	VU	
Fabaceae	Erythrostemon acapulcensis			
Fabaceae	Erythrostemon caladenia			
Fabaceae	Erythrostemon exostemma			
Fabaceae	Erythrostemon mexicanus			
Fabaceae	Erythrostemon yucatanensis			
Fabaceae	Eysenhardtia adenostylis			
Fabaceae	Eysenhardtia platycarpa			
Fabaceae	Eysenhardtia polystachya			
Fabaceae	Eysenhardtia texana			
Fabaceae	Flemingia macrophylla		CR	YES
Fabaceae	Galactia acapulcensis			
Fabaceae	Galactia argentea			
Fabaceae	Galactia brachystachys			
Fabaceae	Galactia incana			
Fabaceae	Galactia multiflora			
Fabaceae	Galactia spiciformis			

Fabaceae	Galactia striata			
Fabaceae	Galactia striata var. villosa		EN	
Fabaceae	Galactia texana		VU	
Fabaceae	Galactia viridiflora			
Fabaceae	Galactia wrightii			
Fabaceae	Gliricidia ehrenbergii			
Fabaceae	Gliricidia maculata			
Fabaceae	Gliricidia robusta		VU	
Fabaceae	Gliricidia sepium			
Fabaceae	Grona adscendens			
Fabaceae	Grona barbata			
Fabaceae	Grona heterocarpos var. strigosa		EN	
Fabaceae	Grona triflora			
Fabaceae	Guilandina bonduc			
Fabaceae	Guilandina major		VU	
Fabaceae	Haematoxylum brasiletto			
Fabaceae	Haematoxylum campechianum			
Fabaceae	Harpalyce arborescens			
Fabaceae	Harpalyce formosa			
Fabaceae	Harpalyce formosa var. formosa			
Fabaceae	Harpalyce formosa var. goldmanii			
Fabaceae	Havardia acatlensis			
Fabaceae	Havardia albicans			
Fabaceae	Havardia campylacantha			
Fabaceae	Havardia pallens			
Fabaceae	Havardia platyloba			
Fabaceae	Helicotropis linearis			
Fabaceae	Helicotropis spectabilis		VU	
Fabaceae	Heteroflorum sclerocarpum	EN		
Fabaceae	Hosackia pinnata		CR	YES
Fabaceae	Hosackia repens			
Fabaceae	Hylodesmum glutinosum			
Fabaceae	Hylodesmum nudiflorum		EN	
Fabaceae	Hymenaea courbaril			

Fabaceae	Indigofera conzattii			
Fabaceae	Indigofera cuernavacana			
Fabaceae	Indigofera densiflora			
Fabaceae	Indigofera hirsuta			
Fabaceae	Indigofera jaliscensis			
Fabaceae	Indigofera lespedezioides			
Fabaceae	Indigofera micheliana		EN	
Fabaceae	Indigofera miniata			
Fabaceae	Indigofera mucronata			
Fabaceae	Indigofera palmeri			
Fabaceae	Indigofera purpusii			
Fabaceae	Indigofera subulata var. scabra			
Fabaceae	Indigofera suffruticosa			
Fabaceae	Indigofera thibaudiana			
Fabaceae	Indigofera trita			
Fabaceae	Inga acrocephala			
Fabaceae	Inga acuminata		CR	
Fabaceae	Inga affinis			
Fabaceae	Inga alba		EN	
Fabaceae	Inga barbourii		VU	
Fabaceae	Inga belizensis		VU	
Fabaceae	Inga cabrerae		CR	YES
Fabaceae	Inga calcicola	VU	EN	
Fabaceae	Inga calderonii			
Fabaceae	Inga chiapensis	VU	VU	
Fabaceae	Inga dasycarpa		EN	
Fabaceae	Inga densiflora		VU	
Fabaceae	Inga edulis			
Fabaceae	Inga flexuosa			
Fabaceae	Inga huastecana		VU	
Fabaceae	Inga inicuil			
Fabaceae	Inga ismaelis	VU	VU	
Fabaceae	Inga laurina			
Fabaceae	Inga marginata		VU	

Fabaceae	Inga mexicana		VU	
Fabaceae	Inga multijuga subsp. aestuariorum			
Fabaceae	Inga nobilis		EN	
Fabaceae	Inga nobilis subsp. quaternata			
Fabaceae	Inga oerstediana			
Fabaceae	Inga paterno			
Fabaceae	Inga pinetorum			
Fabaceae	Inga punctata			
Fabaceae	Inga ruiziana			
Fabaceae	Inga sapindoides			
Fabaceae	Inga sierrae		EN	
Fabaceae	Inga sinacae	EN	VU	
Fabaceae	Inga spectabilis		EN	
Fabaceae	Inga thibaudiana		VU	
Fabaceae	Inga thibaudiana subsp. thibaudiana		CR	YES
Fabaceae	Inga vera			
Fabaceae	Inga vera subsp. eriocarpa			
Fabaceae	Inga vera subsp. vera			
Fabaceae	Inga xalapensis			
Fabaceae	Lachesiodendron viridiflorum			
Fabaceae	Lathyrus latifolius		VU	
Fabaceae	Lathyrus odoratus	CR	VU	
Fabaceae	Lathyrus oleraceus			
Fabaceae	Lathyrus speciosus			
Fabaceae	Lecointea amazonica		VU	
Fabaceae	Lennea melanocarpa			
Fabaceae	Lennea modesta			
Fabaceae	Lennea viridiflora			
Fabaceae	Leptolobium panamense			
Fabaceae	Leptospron adenanthum			
Fabaceae	Leptospron gentryi			
Fabaceae	Lessertia frutescens			
Fabaceae	Leucaena collinsii			
Fabaceae	Leucaena confertiflora			

Fabaceae	Leucaena confertiflora subsp. adenotheloidea		EN	
Fabaceae	Leucaena cuspidata	VU		
Fabaceae	Leucaena diversifolia			
Fabaceae	Leucaena esculenta			
Fabaceae	Leucaena lanceolata			
Fabaceae	Leucaena lanceolata var. lanceolata			
Fabaceae	Leucaena lanceolata var. sousae			
Fabaceae	Leucaena leucocephala			
Fabaceae	Leucaena leucocephala subsp. glabrata			
Fabaceae	Leucaena leucocephala subsp. leucocephala			
Fabaceae	Leucaena macrophylla			
Fabaceae	Leucaena macrophylla subsp. macrophylla			
Fabaceae	Leucaena pallida			
Fabaceae	Leucaena pulverulenta			
Fabaceae	Leucaena shannonii			
Fabaceae	Leucaena trichandra			
Fabaceae	Leucaena trichodes		EN	
Fabaceae	Libidibia coriaria			
Fabaceae	Libidibia glabrata		EN	
Fabaceae	Libidibia sclerocarpa			
Fabaceae	Lonchocarpus acuminatus			
Fabaceae	Lonchocarpus angusticarpus	EN	EN	
Fabaceae	Lonchocarpus atropurpureus			
Fabaceae	Lonchocarpus berriozabalensis	EN	EN	YES
Fabaceae	Lonchocarpus castilloi			
Fabaceae	Lonchocarpus caudatus			
Fabaceae	Lonchocarpus cochleatus	EN		
Fabaceae	Lonchocarpus comitensis	EN	EN	
Fabaceae	Lonchocarpus constrictus			
Fabaceae	Lonchocarpus cruentus			
Fabaceae	Lonchocarpus emarginatus	VU		
Fabaceae	Lonchocarpus eriocarinalis			
Fabaceae	Lonchocarpus eriophyllus			
Fabaceae	Lonchocarpus fuscopurpureus	EN		

Fabaceae	Lonchocarpus guatemalensis		
Fabaceae	Lonchocarpus heptaphyllus		
Fabaceae	Lonchocarpus hidalgensis		
Fabaceae	Lonchocarpus hintonii		
Fabaceae	Lonchocarpus hondurensis		
Fabaceae	Lonchocarpus kerberi		VU
Fabaceae	Lonchocarpus lanceolatus		
Fabaceae	Lonchocarpus lanceolatus subsp. calciphilus		VU
Fabaceae	Lonchocarpus lasiotropis		EN
Fabaceae	Lonchocarpus latimarginatus	CR	VU
Fabaceae	Lonchocarpus lineatus		
Fabaceae	Lonchocarpus longistylus		
Fabaceae	Lonchocarpus luteomaculatus		
Fabaceae	Lonchocarpus major	EN	CR
Fabaceae	Lonchocarpus martinezii	VU	VU
Fabaceae	Lonchocarpus michelianus		VU
Fabaceae	Lonchocarpus minimiflorus		
Fabaceae	Lonchocarpus molinae		EN
Fabaceae	Lonchocarpus multifoliolatus	EN	VU
Fabaceae	Lonchocarpus mutans		
Fabaceae	Lonchocarpus necaxensis	EN	EN
Fabaceae	Lonchocarpus orizabensis	EN	VU
Fabaceae	Lonchocarpus parviflorus		
Fabaceae	Lonchocarpus phaseolifolius		
Fabaceae	Lonchocarpus punctatus		
Fabaceae	Lonchocarpus robustus	VU	
Fabaceae	Lonchocarpus rugosus		
Fabaceae	Lonchocarpus rugosus subsp. apricus		
Fabaceae	Lonchocarpus rugosus subsp. gillyi		
Fabaceae	Lonchocarpus rugosus subsp. rugosus		
Fabaceae	Lonchocarpus salvadorensis		
Fabaceae	Lonchocarpus sanctuarii		VU
Fabaceae	Lonchocarpus santarosanus	VU	
Fabaceae	Lonchocarpus schiedeanus		

Fabaceae	Lonchocarpus sericeus			
Fabaceae	Lonchocarpus sumiderensis	VU	CR	YES
Fabaceae	Lonchocarpus tuxtepecensis	EN	CR	YES
Fabaceae	Lonchocarpus verrucosus			
Fabaceae	Lonchocarpus violaceus			
Fabaceae	Lonchocarpus wendtii	VU	EN	YES
Fabaceae	Lupinus aschenbornii			
Fabaceae	Lupinus chiapensis		VU	
Fabaceae	Lupinus ehrenbergii var. ehrenbergii		EN	
Fabaceae	Lupinus elegans			
Fabaceae	Lupinus elegans var. elegans			
Fabaceae	Lupinus montanus			
Fabaceae	Lupinus montanus var. nelsonii		EN	
Fabaceae	Lupinus simulans			
Fabaceae	Lupinus splendens			
Fabaceae	Lupinus versicolor		VU	
Fabaceae	Lysiloma acapulcense			
Fabaceae	Lysiloma auritum			
Fabaceae	Lysiloma divaricatum			
Fabaceae	Lysiloma latisiliquum			
Fabaceae	Lysiloma tergeminum			
Fabaceae	Machaerium × salvadorense			
Fabaceae	Machaerium arboreum			
Fabaceae	Machaerium biovulatum			
Fabaceae	Machaerium chiapense			
Fabaceae	Machaerium cirrhiferum			
Fabaceae	Machaerium cobanense			
Fabaceae	Machaerium conzattii	EN	CR	YES
Fabaceae	Machaerium falciforme			
Fabaceae	Machaerium floribundum			
Fabaceae	Machaerium floribundum var. floribundum		EN	
Fabaceae	Machaerium isadelphum			
Fabaceae	Machaerium kegelii			
Fabaceae	Machaerium lunatum			

Fabaceae	Machaerium pittieri	
Fabaceae	Machaerium riparium	
Fabaceae	Machaerium seemannii	
Fabaceae	Macroptilium atropurpureum	
Fabaceae	Macroptilium gibbosifolium	
Fabaceae	Macroptilium gracile	
Fabaceae	Macroptilium lathyroides	
Fabaceae	Macroptilium longepedunculatum	
Fabaceae	Marina diffusa	
Fabaceae	Marina ghiesbreghtii	VU
Fabaceae	Marina neglecta	
Fabaceae	Marina nutans	
Fabaceae	Marina scopa	
Fabaceae	Marina spiciformis	
Fabaceae	Mariosousa acatlensis	
Fabaceae	Mariosousa centralis	
Fabaceae	Mariosousa coulteri	
Fabaceae	Mariosousa dolichostachya	
Fabaceae	Mariosousa durangensis	
Fabaceae	Mariosousa mammifera	
Fabaceae	Mariosousa millefolia	
Fabaceae	Mariosousa salazarii	
Fabaceae	Mariosousa usumacintensis	
Fabaceae	Medicago lupulina	
Fabaceae	Medicago polymorpha	
Fabaceae	Medicago sativa	
Fabaceae	Melilotus albus	
Fabaceae	Melilotus indicus	
Fabaceae	Microlobius foetidus	
Fabaceae	Mimosa acantholoba	
Fabaceae	Mimosa acantholoba var. acantholoba	
Fabaceae	Mimosa acantholoba var. eurycarpa	
Fabaceae	Mimosa acantholoba var. molinarum	EN
Fabaceae	Mimosa aculeaticarpa	

Fabaceae	Mimosa aculeaticarpa var. aculeaticarpa	
Fabaceae	Mimosa aculeaticarpa var. biuncifera	
Fabaceae	Mimosa adenantheroides	
Fabaceae	Mimosa affinis	
Fabaceae	Mimosa albida	
Fabaceae	Mimosa albida var. albida	
Fabaceae	Mimosa albida var. glabrior	
Fabaceae	Mimosa albida var. willdenowii	
Fabaceae	Mimosa antioquensis	
Fabaceae	Mimosa arenosa	
Fabaceae	Mimosa argillotropha	EN
Fabaceae	Mimosa bahamensis	
Fabaceae	Mimosa benthamii	
Fabaceae	Mimosa caerulea	
Fabaceae	Mimosa calcicola	
Fabaceae	Mimosa candollei	
Fabaceae	Mimosa casta	EN
Fabaceae	Mimosa deamii	
Fabaceae	Mimosa depauperata	
Fabaceae	Mimosa diffusa	VU
Fabaceae	Mimosa diplotricha	
Fabaceae	Mimosa diplotricha var. diplotricha	
Fabaceae	Mimosa distachya	
Fabaceae	Mimosa distachya var. distachya	
Fabaceae	Mimosa ervendbergii	
Fabaceae	Mimosa galeottii	
Fabaceae	Mimosa goldmanii	
Fabaceae	Mimosa guilandinae	EN
Fabaceae	Mimosa guilandinae var. extensissima	EN
Fabaceae	Mimosa hondurana	
Fabaceae	Mimosa invisa	
Fabaceae	Mimosa lacerata	
Fabaceae	Mimosa lactiflua	
Fabaceae	Mimosa leptocarpa	

Fabaceae	Mimosa leucaenoides	
Fabaceae	Mimosa luisana	
Fabaceae	Mimosa mellii	
Fabaceae	Mimosa monancistra	
Fabaceae	Mimosa occidentalis	
Fabaceae	Mimosa occidentalis var. occidentalis	VU
Fabaceae	Mimosa pellita	
Fabaceae	Mimosa pigra	
Fabaceae	Mimosa pigra var. asperata	
Fabaceae	Mimosa pigra var. pigra	
Fabaceae	Mimosa platycarpa	
Fabaceae	Mimosa polyantha	
Fabaceae	Mimosa polydactyla	EN
Fabaceae	Mimosa psilocarpa	
Fabaceae	Mimosa pudica	
Fabaceae	Mimosa pudica var. tetrandra	VU
Fabaceae	Mimosa pudica var. unijuga	
Fabaceae	Mimosa purpusii	
Fabaceae	Mimosa quadrivalvis	
Fabaceae	Mimosa quadrivalvis var. quadrivalvis	VU
Fabaceae	Mimosa rhodocarpa	
Fabaceae	Mimosa robusta	
Fabaceae	Mimosa rosei	
Fabaceae	Mimosa skinneri	
Fabaceae	Mimosa skinneri var. skinneri	
Fabaceae	Mimosa somnians	
Fabaceae	Mimosa somnians var. somnians	VU
Fabaceae	Mimosa sousae	EN
Fabaceae	Mimosa tarda	VU
Fabaceae	Mimosa teledactyla	EN
Fabaceae	Mimosa tenuiflora	
Fabaceae	Mimosa torresiae	EN
Fabaceae	Mimosa tricephala	
Fabaceae	Mimosa tricephala var. nelsonii	

Fabaceae	Mimosa tricephala var. xanti				
Fabaceae	Mimosa ursina			VU	
Fabaceae	Mimosa velloziana				
Fabaceae	Mimosa watsonii				
Fabaceae	Mimosa weddelliana				
Fabaceae	Mucuna argyrophylla				
Fabaceae	Mucuna holtonii				
Fabaceae	Mucuna jarocha			CR	YES
Fabaceae	Mucuna mollis			EN	
Fabaceae	Mucuna pruriens				
Fabaceae	Mucuna sloanei				
Fabaceae	Mucuna urens			VU	
Fabaceae	Muellera torrensis		EN	EN	
Fabaceae	Muellera unifoliolata				
Fabaceae	Myrospermum frutescens				
Fabaceae	Myroxylon balsamum				
Fabaceae	Neptunia pubescens var. pubescens				
Fabaceae	Nesphostylis lanceolata			VU	
Fabaceae	Neustanthus phaseoloides				
Fabaceae	Nissolia brasiliensis				
Fabaceae	Nissolia chiapensis				
Fabaceae	Nissolia fruticosa				
Fabaceae	Nissolia leiogyne				
Fabaceae	Nissolia microptera				
Fabaceae	Nissolia platycarpa				
Fabaceae	Nissolia pringlei				
Fabaceae	Nissolia vincentina				
Fabaceae	Orbexilum melanocarpum				
Fabaceae	Orbexilum oliganthum				
Fabaceae	Ormosia coccinea			CR	
Fabaceae	Ormosia isthmensis	EN			
Fabaceae	Ormosia macrocalyx	EN			
Fabaceae	Ormosia oaxacana		VU	VU	
Fabaceae	Ormosia panamensis		VU	VU	

Fabaceae	Ormosia velutina				
Fabaceae	Oxyrhynchus trinervius			VU	
Fabaceae	Oxyrhynchus volubilis				
Fabaceae	Pachyrhizus erosus				
Fabaceae	Pachyrhizus ferrugineus				
Fabaceae	Painteria elachistophylla				
Fabaceae	Painteria leptophylla				
Fabaceae	Parasenegalia visco				
Fabaceae	Paraserianthes lophantha subsp. lophantha			EN	
Fabaceae	Parkinsonia aculeata				
Fabaceae	Parkinsonia florida subsp. florida				
Fabaceae	Parkinsonia praecox				
Fabaceae	Parkinsonia texana				
Fabaceae	Paubrasilia echinata		EN		
Fabaceae	Pediomelum rhombifolium				
Fabaceae	Peltogyne mexicana	VU		VU	
Fabaceae	Peltophorum dubium			EN	
Fabaceae	Phanera retusa			CR	YES
Fabaceae	Phaseolus chiapasanus		EN		
Fabaceae	Phaseolus esperanzae		VU		
Fabaceae	Phaseolus esquincensis			EN	
Fabaceae	Phaseolus glabellus				
Fabaceae	Phaseolus leptostachyus				
Fabaceae	Phaseolus maculatus subsp. maculatus				
Fabaceae	Phaseolus macvaughii				
Fabaceae	Phaseolus marechalii			VU	
Fabaceae	Phaseolus microcarpus				
Fabaceae	Phaseolus neglectus				
Fabaceae	Phaseolus nelsonii		VU		
Fabaceae	Phaseolus oligospermus		VU		
Fabaceae	Phaseolus parvifolius				
Fabaceae	Phaseolus pauciflorus				
Fabaceae	Phaseolus pedicellatus				
Fabaceae	Phaseolus pedicellatus var. pedicellatus				

Fabaceae	Phaseolus tuerckheimii			
Fabaceae	Phaseolus vulcanicus			
Fabaceae	Phaseolus xanthotrichus			
Fabaceae	Phaseolus xolocotzii		VU	
Fabaceae	Phaseolus zimapanensis			
Fabaceae	Philenoptera cyanescens		CR	YES
Fabaceae	Piptadenia flava			
Fabaceae	Piscidia carthagenensis			
Fabaceae	Piscidia grandifolia			
Fabaceae	Piscidia piscipula			
Fabaceae	Pithecellobium dulce			
Fabaceae	Pithecellobium furcatum			
Fabaceae	Pithecellobium hymenaeifolium			
Fabaceae	Pithecellobium lanceolatum			
Fabaceae	Pithecellobium macrandrium			
Fabaceae	Pithecellobium unguis-cati			
Fabaceae	Pityrocarpa obliqua			
Fabaceae	Pityrocarpa obliqua subsp. obliqua			
Fabaceae	Platymiscium dimorphandrum			
Fabaceae	Platymiscium jejunum		VU	
Fabaceae	Platymiscium pinnatum			
Fabaceae	Platymiscium yucatanum			
Fabaceae	Poeppigia procera			
Fabaceae	Poiretia punctata			
Fabaceae	Prosopis glandulosa			
Fabaceae	Prosopis juliflora			
Fabaceae	Prosopis laevigata			
Fabaceae	Prosopis tamaulipana			
Fabaceae	Pseudosamanea guachapele			
Fabaceae	Pterocarpus acapulcensis	VU		
Fabaceae	Pterocarpus amphymenium			
Fabaceae	Pterocarpus belizensis		VU	
Fabaceae	Pterocarpus orbiculatus			
Fabaceae	Pterocarpus rohrii			

Fabaceae	Ramirezella crassa		
Fabaceae	Ramirezella lozanii		
Fabaceae	Ramirezella micrantha	VU	
Fabaceae	Ramirezella nitida	VU	
Fabaceae	Ramirezella penduliflora	CR	YES
Fabaceae	Ramirezella strobilophora		
Fabaceae	Ramirezella strobilophora var. buseri		
Fabaceae	Rhynchosia amabilis		
Fabaceae	Rhynchosia americana		
Fabaceae	Rhynchosia difformis	EN	
Fabaceae	Rhynchosia discolor		
Fabaceae	Rhynchosia edulis		
Fabaceae	Rhynchosia erythrinoides		
Fabaceae	Rhynchosia latifolia	EN	
Fabaceae	Rhynchosia longeracemosa		
Fabaceae	Rhynchosia macrocarpa		
Fabaceae	Rhynchosia minima		
Fabaceae	Rhynchosia minima var. minima		
Fabaceae	Rhynchosia monticola	EN	
Fabaceae	Rhynchosia nelsonii	VU	
Fabaceae	Rhynchosia phaseoloides		
Fabaceae	Rhynchosia precatoria		
Fabaceae	Rhynchosia pyramidalis		
Fabaceae	Rhynchosia reticulata		
Fabaceae	Rhynchosia senna		
Fabaceae	Rhynchosia tamaulipensis	VU	
Fabaceae	Rhynchosia yucatanensis		
Fabaceae	Samanea saman		
Fabaceae	Schizolobium parahyba		
Fabaceae	Schnella glabra		
Fabaceae	Schnella guianensis	EN	
Fabaceae	Schnella herrerae		
Fabaceae	Schnella microstachya	VU	
Fabaceae	Senegalia aristeguietana	CR	YES

Fabaceae	Senegalia berlandieri		
Fabaceae	Senegalia hayesii		
Fabaceae	Senegalia loretensis	EN	
Fabaceae	Senegalia micrantha		
Fabaceae	Senegalia mirandae	VU	
Fabaceae	Senegalia picachensis		
Fabaceae	Senegalia polyphylla		
Fabaceae	Senegalia purpusii		
Fabaceae	Senegalia riparia		
Fabaceae	Senegalia roemeriana		
Fabaceae	Senegalia subangulata		
Fabaceae	Senegalia tenuifolia		
Fabaceae	Senegalia tucumanensis		
Fabaceae	Senna × floribunda		
Fabaceae	Senna alata		
Fabaceae	Senna andrieuxii		
Fabaceae	Senna apiculata		
Fabaceae	Senna atomaria		
Fabaceae	Senna bacillaris		
Fabaceae	Senna bacillaris var. bacillaris	EN	
Fabaceae	Senna bicapsularis		
Fabaceae	Senna bicapsularis var. bicapsularis		
Fabaceae	Senna centranthera		
Fabaceae	Senna cobanensis		
Fabaceae	Senna demissa		
Fabaceae	Senna didymobotrya		
Fabaceae	Senna foetidissima		
Fabaceae	Senna foetidissima var. grandiflora		
Fabaceae	Senna fruticosa		
Fabaceae	Senna galeottiana		
Fabaceae	Senna guatemalensis		
Fabaceae	Senna guatemalensis var. calcarea	EN	YES
Fabaceae	Senna guatemalensis var. chiapensis		
Fabaceae	Senna guatemalensis var. oligantha	EN	

Fabaceae	Senna hayesiana		
Fabaceae	Senna hirsuta		
Fabaceae	Senna hirsuta var. glaberrima		
Fabaceae	Senna hirsuta var. hirsuta		
Fabaceae	Senna hirsuta var. hirta		
Fabaceae	Senna hirsuta var. leptocarpa		
Fabaceae	Senna holwayana		
Fabaceae	Senna holwayana var. holwayana		
Fabaceae	Senna incarnata	VU	
Fabaceae	Senna leiophylla		
Fabaceae	Senna lindheimeriana		
Fabaceae	Senna mexicana	VU	
Fabaceae	Senna mollissima		
Fabaceae	Senna mollissima var. glabrata		
Fabaceae	Senna multifoliolata var. mimetes	VU	
Fabaceae	Senna multiglandulosa		
Fabaceae	Senna multijuga		
Fabaceae	Senna multijuga subsp. doylei		
Fabaceae	Senna nicaraguensis		
Fabaceae	Senna obtusifolia		
Fabaceae	Senna occidentalis		
Fabaceae	Senna oxyphylla	VU	
Fabaceae	Senna pallida		
Fabaceae	Senna pallida var. brachyrrhachis		
Fabaceae	Senna pallida var. delgadoana	EN	
Fabaceae	Senna pallida var. foliolosa	CR	YES
Fabaceae	Senna pallida var. geminiflora		
Fabaceae	Senna pallida var. isthmica		
Fabaceae	Senna pallida var. lemniscata	VU	
Fabaceae	Senna pallida var. pallida		
Fabaceae	Senna pallida var. palmeri		
Fabaceae	Senna pallida var. quiedondilla		
Fabaceae	Senna pallida var. trichocraspedon		
Fabaceae	Senna pallida var. triquetripes		

Fabaceae	Senna papillosa	
Fabaceae	Senna papillosa var. papillosa	
Fabaceae	Senna pendula	
Fabaceae	Senna pendula var. indecora	
Fabaceae	Senna pendula var. ovalifolia	
Fabaceae	Senna pendula var. pendula	
Fabaceae	Senna pentagonia	VU
Fabaceae	Senna pentagonia var. pentagonia	VU
Fabaceae	Senna peralteana	
Fabaceae	Senna pilifera	
Fabaceae	Senna polyantha	
Fabaceae	Senna quinquangulata	
Fabaceae	Senna quinquangulata var. quinquangulata	
Fabaceae	Senna racemosa	
Fabaceae	Senna racemosa var. coalcomanica	VU
Fabaceae	Senna racemosa var. liebmannii	
Fabaceae	Senna racemosa var. moctezumae	
Fabaceae	Senna racemosa var. racemosa	
Fabaceae	Senna reticulata	
Fabaceae	Senna septemtrionalis	
Fabaceae	Senna skinneri	
Fabaceae	Senna spectabilis	
Fabaceae	Senna spectabilis var. spectabilis	
Fabaceae	Senna tonduzii	
Fabaceae	Senna tora	
Fabaceae	Senna undulata	
Fabaceae	Senna uniflora	
Fabaceae	Senna unijuga	
Fabaceae	Senna villosa	
Fabaceae	Senna wislizeni	
Fabaceae	Senna wislizeni var. pringlei	
Fabaceae	Sesbania emerus	
Fabaceae	Sesbania herbacea	
Fabaceae	Sigmoidotropis elegans	

Fabaceae	Sigmoidotropis speciosa			
Fabaceae	Stylosanthes erecta		CR	YES
Fabaceae	Stylosanthes guianensis			
Fabaceae	Stylosanthes guianensis subsp. dissitiflora			
Fabaceae	Stylosanthes humilis			
Fabaceae	Stylosanthes macrocarpa			
Fabaceae	Stylosanthes viscosa			
Fabaceae	Styphnolobium burseroides	VU		
Fabaceae	Styphnolobium parviflorum	EN	EN	
Fabaceae	Styphnolobium sporadicum		EN	YES
Fabaceae	Swartzia cubensis			
Fabaceae	Swartzia cubensis var. cubensis			
Fabaceae	Swartzia guatemalensis			
Fabaceae	Swartzia mexicana	VU	EN	
Fabaceae	Swartzia myrtifolia		VU	
Fabaceae	Swartzia myrtifolia var. myrtifolia		EN	
Fabaceae	Swartzia myrtifolia var. standleyi		VU	
Fabaceae	Swartzia simplex			
Fabaceae	Swartzia simplex var. continentalis			
Fabaceae	Swartzia standleyi		VU	
Fabaceae	Tamarindus indica			
Fabaceae	Tara cacalaco			
Fabaceae	Tephrosia belizensis			
Fabaceae	Tephrosia cinerea			
Fabaceae	Tephrosia conzattii			
Fabaceae	Tephrosia crassifolia			
Fabaceae	Tephrosia diversifolia			
Fabaceae	Tephrosia domingensis			
Fabaceae	Tephrosia lanata			
Fabaceae	Tephrosia langlassei			
Fabaceae	Tephrosia leiocarpa			
Fabaceae	Tephrosia major			
Fabaceae	Tephrosia multifolia			
Fabaceae	Tephrosia nicaraguensis			

Fabaceae	Tephrosia nitens	
Fabaceae	Tephrosia potosina	
Fabaceae	Tephrosia pringlei	
Fabaceae	Tephrosia purpurea subsp. purpurea	EN
Fabaceae	Tephrosia rhodantha	
Fabaceae	Tephrosia sinapou	
Fabaceae	Tephrosia vicioides	
Fabaceae	Teramnus labialis	
Fabaceae	Teramnus uncinatus	
Fabaceae	Teramnus volubilis	EN
Fabaceae	Trifolium amabile	
Fabaceae	Trifolium amabile var. hemsleyi	VU
Fabaceae	Trifolium amabile var. mexicanum	
Fabaceae	Trifolium pratense	
Fabaceae	Trifolium repens	
Fabaceae	Trifolium rhombeum	
Fabaceae	Vachellia californica	
Fabaceae	Vachellia campeachiana	
Fabaceae	Vachellia chiapensis	
Fabaceae	Vachellia collinsii	
Fabaceae	Vachellia constricta	
Fabaceae	Vachellia cookii	VU
Fabaceae	Vachellia cornigera	
Fabaceae	Vachellia farnesiana	
Fabaceae	Vachellia gentlei	
Fabaceae	Vachellia globulifera	
Fabaceae	Vachellia hindsii	
Fabaceae	Vachellia janzenii	VU
Fabaceae	Vachellia macracantha	
Fabaceae	Vachellia mayana	
Fabaceae	Vachellia pacensis	
Fabaceae	Vachellia pennatula	
Fabaceae	Vachellia pennatula var. parvicephala	
Fabaceae	Vachellia pennatula var. pennatula	

Fabaceae	Vachellia pringlei			
Fabaceae	Vachellia rigidula			
Fabaceae	Vachellia schaffneri			
Fabaceae	Vachellia tortuosa			
Fabaceae	Vatairea lundellii	EN		
Fabaceae	Vicia faba			
Fabaceae	Vicia humilis			
Fabaceae	Vicia ludoviciana			
Fabaceae	Vicia pulchella			
Fabaceae	Vicia sativa			
Fabaceae	Vigna luteola			
Fabaceae	Vigna mungo		EN	YES
Fabaceae	Vigna umbellata			
Fabaceae	Vigna unguiculata			
Fabaceae	Vigna unguiculata subsp. unguiculata			
Fabaceae	Vigna vexillata			
Fabaceae	Zapoteca formosa			
Fabaceae	Zapoteca formosa subsp. formosa			
Fabaceae	Zapoteca formosa subsp. rosei			
Fabaceae	Zapoteca lambertiana			
Fabaceae	Zapoteca portoricensis			
Fabaceae	Zapoteca portoricensis subsp. flavida			
Fabaceae	Zapoteca portoricensis subsp. portoricensis			
Fabaceae	Zapoteca ravenii		VU	
Fabaceae	Zapoteca tehuana		VU	
Fabaceae	Zapoteca tetragona			
Fabaceae	Zornia diphylla			
Fabaceae	Zornia laevis		EN	
Fabaceae	Zornia reticulata			
Fabaceae	Zornia thymifolia			
Fabaceae	Zygia cognata			
Fabaceae	Zygia conzattii			
Fabaceae	Zygia latifolia			
Fabaceae	Zygia longifolia			

Fabaceae	Zygia paucijugata				
Fabaceae	Zygia peckii				
Fabaceae	Zygia turneri			EN	
Fabaceae	Zygia unifoliolata			VU	
Fagaceae	Fagus grandifolia				
Fagaceae	Fagus grandifolia subsp. mexicana	VU			
Fagaceae	Quercus × dysophylla				
Fagaceae	Quercus acatenangensis				
Fagaceae	Quercus acherdophylla				
Fagaceae	Quercus acutifolia		VU		
Fagaceae	Quercus affinis				
Fagaceae	Quercus aristata				
Fagaceae	Quercus augustini			CR	YES
Fagaceae	Quercus benthamii				
Fagaceae	Quercus brenesii			EN	
Fagaceae	Quercus calophylla				
Fagaceae	Quercus canbyi				
Fagaceae	Quercus castanea				
Fagaceae	Quercus chihuahuensis				
Fagaceae	Quercus chinantlensis			EN	
Fagaceae	Quercus conzattii				
Fagaceae	Quercus corrugata				
Fagaceae	Quercus cortesii				
Fagaceae	Quercus crassifolia				
Fagaceae	Quercus crassipes				
Fagaceae	Quercus crispifolia				
Fagaceae	Quercus crispipilis				
Fagaceae	Quercus depressa				
Fagaceae	Quercus deserticola				
Fagaceae	Quercus diversifolia		EN		
Fagaceae	Quercus eduardi				
Fagaceae	Quercus elliptica				
Fagaceae	Quercus frutex				
Fagaceae	Quercus fulva				

Fagaceae	Quercus furfuracea	VU	
Fagaceae	Quercus germana		
Fagaceae	Quercus ghiesbreghtii		VU
Fagaceae	Quercus glabrescens		
Fagaceae	Quercus glaucescens		
Fagaceae	Quercus glaucoides		
Fagaceae	Quercus grahamii		
Fagaceae	Quercus greggii		
Fagaceae	Quercus grisea		
Fagaceae	Quercus hirtifolia	EN	
Fagaceae	Quercus insignis	EN	
Fagaceae	Quercus jonesii		
Fagaceae	Quercus laeta		
Fagaceae	Quercus lancifolia		
Fagaceae	Quercus laurifolia		
Fagaceae	Quercus laurina		
Fagaceae	Quercus liebmannii		
Fagaceae	Quercus magnoliifolia		
Fagaceae	Quercus mexicana		
Fagaceae	Quercus microphylla		
Fagaceae	Quercus obtusata		
Fagaceae	Quercus oleoides		
Fagaceae	Quercus opaca		
Fagaceae	Quercus paxtalensis		
Fagaceae	Quercus peduncularis		
Fagaceae	Quercus pinnativenulosa		
Fagaceae	Quercus planipocula		
Fagaceae	Quercus polymorpha		
Fagaceae	Quercus purulhana		
Fagaceae	Quercus repanda		
Fagaceae	Quercus rugosa		
Fagaceae	Quercus rysophylla		
Fagaceae	Quercus salicifolia		
Fagaceae	Quercus sapotifolia		

Fagaceae	Quercus sartorii			
Fagaceae	Quercus scytophylla			
Fagaceae	Quercus sebifera			
Fagaceae	Quercus seemannii			
Fagaceae	Quercus segoviensis			
Fagaceae	Quercus sideroxyla			
Fagaceae	Quercus skinneri			
Fagaceae	Quercus sororia			
Fagaceae	Quercus subspathulata			
Fagaceae	Quercus tinkhamii			
Fagaceae	Quercus urbani			
Fagaceae	Quercus vicentensis		VU	
Fagaceae	Quercus xalapensis			
Fouquieriaceae	Fouquieria fasciculata	VU	VU	
Fouquieriaceae	Fouquieria formosa			
Fouquieriaceae	Fouquieria macdougalii			
Garryaceae	Garrya glaberrima			
Garryaceae	Garrya goldmanii			
Garryaceae	Garrya laurifolia			
Garryaceae	Garrya laurifolia subsp. laurifolia			
Garryaceae	Garrya laurifolia subsp. macrophylla			
Garryaceae	Garrya laurifolia subsp. quichensis			
Garryaceae	Garrya longifolia			
Garryaceae	Garrya ovata			
Gelsemiaceae	Gelsemium sempervirens			
Gentianaceae	Coutoubea spicata			
Gentianaceae	Eustoma exaltatum			
Gentianaceae	Geniostemon rotundifolius			EN
Gentianaceae	Gentiana arisanensis			EN
Gentianaceae	Gentiana bicuspidata			
Gentianaceae	Gentiana ovatiloba			
Gentianaceae	Gentiana spathacea	NT		
Gentianaceae	Gentianella amarella			
Gentianaceae	Gyrandra brachycalyx			

Gentianaceae	Gyrandra pauciflora	VU	
Gentianaceae	Gyrandra pterocaulis		
Gentianaceae	Halenia alata	EN	
Gentianaceae	Halenia brevicornis		
Gentianaceae	Halenia conzattii		
Gentianaceae	Halenia decumbens		
Gentianaceae	Halenia plantaginea		
Gentianaceae	Helia acutangula	VU	
Gentianaceae	Helia alata		
Gentianaceae	Helia grandiflora	EN	
Gentianaceae	Lisianthius axillaris		
Gentianaceae	Lisianthius brevidentatus		
Gentianaceae	Lisianthius cuspidatus	VU	
Gentianaceae	Lisianthius nigrescens		
Gentianaceae	Lisianthius oreopolus		
Gentianaceae	Lisianthius perkinsiae	EN	
Gentianaceae	Lisianthius quichensis		
Gentianaceae	Lisianthius saponarioides		
Gentianaceae	Lisianthius viscidiflorus	VU	
Gentianaceae	Schultesia brachyptera		
Gentianaceae	Schultesia guianensis		
Gentianaceae	Voyria aphylla		
Gentianaceae	Voyria aurantiaca	CR	YES
Gentianaceae	Voyria corymbosa subsp. alba	EN	
Gentianaceae	Voyria flavescens	EN	
Gentianaceae	Voyria parasitica		
Gentianaceae	Voyria tenella		
Gentianaceae	Voyria truncata	EN	
Gentianaceae	Xestaea lisianthoides	VU	
Gentianaceae	Zeltnera quitensis		
Gentianaceae	Zeltnera stricta		
Geraniaceae	Erodium cicutarium		
Geraniaceae	Erodium moschatum		
Geraniaceae	Geranium alpicola		

Geraniaceae	Geranium andicola		
Geraniaceae	Geranium bellum		
Geraniaceae	Geranium campanulatum	VU	
Geraniaceae	Geranium deltoideum		
Geraniaceae	Geranium goldmanii		
Geraniaceae	Geranium hernandesii		
Geraniaceae	Geranium lilacinum		
Geraniaceae	Geranium mexicanum		
Geraniaceae	Geranium monanthum		
Geraniaceae	Geranium oaxacanum		
Geraniaceae	Geranium potentillifolium		
Geraniaceae	Geranium schiedeanum		
Geraniaceae	Geranium seemannii		
Geraniaceae	Geranium seemannii subsp. repens		
Geraniaceae	Pelargonium × hybridum		
Geraniaceae	Pelargonium peltatum		
Geraniaceae	Pelargonium tomentosum	CR	YES
Geraniaceae	Pelargonium zonale		
Gesneriaceae	Achimenes admirabilis	VU	
Gesneriaceae	Achimenes antirrhina		
Gesneriaceae	Achimenes candida		
Gesneriaceae	Achimenes cettoana		
Gesneriaceae	Achimenes dulcis		
Gesneriaceae	Achimenes erecta		
Gesneriaceae	Achimenes flava		
Gesneriaceae	Achimenes grandiflora		
Gesneriaceae	Achimenes heterophylla		
Gesneriaceae	Achimenes longiflora		
Gesneriaceae	Achimenes misera		
Gesneriaceae	Achimenes obscura		
Gesneriaceae	Achimenes pedunculata		
Gesneriaceae	Achimenes skinneri	VU	
Gesneriaceae	Achimenes woodii	VU	
Gesneriaceae	Alsobia chiapensis	EN	

Gesneriaceae	Alsobia punctata	VU	
Gesneriaceae	Amalophyllon parviflorum	EN	
Gesneriaceae	Amalophyllon repens	EN	
Gesneriaceae	Amalophyllon rupestre		
Gesneriaceae	Besleria conspecta	VU	
Gesneriaceae	Besleria cyrtanthemum		
Gesneriaceae	Besleria glabra		
Gesneriaceae	Besleria laxiflora		
Gesneriaceae	Chrysothemis pulchella		
Gesneriaceae	Codonanthopsis crassifolia		
Gesneriaceae	Codonanthopsis uleana		
Gesneriaceae	Columnea erythrophaea		
Gesneriaceae	Columnea guatemalensis		
Gesneriaceae	Columnea hirta	EN	
Gesneriaceae	Columnea nervosa		
Gesneriaceae	Columnea purpurata	EN	
Gesneriaceae	Columnea purpusii		
Gesneriaceae	Columnea schiedeana		
Gesneriaceae	Columnea sulfurea		
Gesneriaceae	Diastema racemiferum		
Gesneriaceae	Diastema rupestre	EN	
Gesneriaceae	Drymonia oinochrophylla		
Gesneriaceae	Drymonia serrulata		
Gesneriaceae	Drymonia strigosa		
Gesneriaceae	Episcia cupreata		
Gesneriaceae	Eucodonia andrieuxii		
Gesneriaceae	Eucodonia verticillata		
Gesneriaceae	Glossoloma cucullatum	VU	
Gesneriaceae	Glossoloma tetragonum	VU	
Gesneriaceae	Kohleria rugata		
Gesneriaceae	Kohleria spicata		
Gesneriaceae	Kohleria tigridia	EN	
Gesneriaceae	Moussonia adpressipilosa	CR	YES
Gesneriaceae	Moussonia deppeana		

Gesneriaceae	Moussonia elegans		
Gesneriaceae	Moussonia fruticosa		
Gesneriaceae	Moussonia hirsutissima	VU	
Gesneriaceae	Moussonia rupicola		
Gesneriaceae	Moussonia skutchii	VU	
Gesneriaceae	Napeanthus apodemus	EN	
Gesneriaceae	Napeanthus bracteatus	VU	
Gesneriaceae	Niphaea mexicana	VU	
Gesneriaceae	Phinaea multiflora		
Gesneriaceae	Rhynchoglossum azureum		
Gesneriaceae	Sinningia incarnata		
Gesneriaceae	Smithiantha aurantiaca	EN	
Gesneriaceae	Smithiantha cinnabarina		
Gesneriaceae	Smithiantha multiflora		
Gesneriaceae	Smithiantha zebrina		
Gesneriaceae	Solenophora chiapasensis	VU	
Gesneriaceae	Solenophora glomerata		
Gesneriaceae	Solenophora insignis		
Gesneriaceae	Solenophora modesta	CR	YES
Gesneriaceae	Solenophora obscura	VU	
Gesneriaceae	Solenophora pirana	EN	
Gesneriaceae	Solenophora purpusii		
Gesneriaceae	Solenophora wilsonii	EN	
Gesneriaceae	Trichodrymonia congesta	EN	
Gleicheniaceae	Dicranopteris flexuosa		
Gleicheniaceae	Diplopterygium bancroftii		
Gleicheniaceae	Gleichenella pectinata		
Gleicheniaceae	Sticherus bifidus		
Gleicheniaceae	Sticherus ferrugineus	EN	
Gleicheniaceae	Sticherus fulvus		
Gleicheniaceae	Sticherus furcatus		
Gleicheniaceae	Sticherus underwoodianus		
Goodeniaceae	Scaevola plumieri		
Grossulariaceae	Ribes affine		

Grossulariaceae	Ribes ciliatum				
Grossulariaceae	Ribes microphyllum				
Grossulariaceae	Ribes orizabae			CR	YES
Guamatelaceae	Guamatela tuerckheimii				
Gunneraceae	Gunnera insignis			VU	
Gunneraceae	Gunnera killipiana			VU	
Gunneraceae	Gunnera mexicana				
Haemodoraceae	Xiphidium caeruleum				
Haloragaceae	Proserpinaca palustris			VU	
Hamamelidaceae	Hamamelis virginiana			VU	
Hamamelidaceae	Hamamelis virginiana var. mexicana			EN	
Hamamelidaceae	Matudaea trinervia	VU	VU		
Heliconiaceae	Heliconia adflexa				
Heliconiaceae	Heliconia aemygdiana			EN	
Heliconiaceae	Heliconia aurantiaca				
Heliconiaceae	Heliconia bihai				
Heliconiaceae	Heliconia bourgaeana				
Heliconiaceae	Heliconia collinsiana				
Heliconiaceae	Heliconia collinsiana var. collinsiana				
Heliconiaceae	Heliconia collinsiana var. velutina			EN	
Heliconiaceae	Heliconia dielsiana			EN	
Heliconiaceae	Heliconia latispatha				
Heliconiaceae	Heliconia librata				
Heliconiaceae	Heliconia longiflora			CR	YES
Heliconiaceae	Heliconia mathiasiae				
Heliconiaceae	Heliconia pendula			CR	YES
Heliconiaceae	Heliconia psittacorum				
Heliconiaceae	Heliconia rostrata				
Heliconiaceae	Heliconia schiedeana				
Heliconiaceae	Heliconia spissa				
Heliconiaceae	Heliconia stricta			VU	
Heliconiaceae	Heliconia subulata			EN	
Heliconiaceae	Heliconia tortuosa			VU	
Heliconiaceae	Heliconia uxpanapensis				

Heliconiaceae	Heliconia vaginalis			
Heliconiaceae	Heliconia wagneriana			
Hernandiaceae	Gyrocarpus americanus			
Hernandiaceae	Gyrocarpus jatrophifolius			
Hernandiaceae	Gyrocarpus mocinoi		VU	
Hernandiaceae	Hernandia didymantha			EN
Hernandiaceae	Hernandia sonora			VU
Hernandiaceae	Hernandia stenura			EN
Hernandiaceae	Hernandia wendtii			EN
Hernandiaceae	Sparattanthelium amazonum			
Hydrangeaceae	Deutzia mexicana			VU
Hydrangeaceae	Hydrangea albostellata			EN
Hydrangeaceae	Hydrangea macrophylla			
Hydrangeaceae	Hydrangea nebulicola	EN		
Hydrangeaceae	Hydrangea steyermarkii			
Hydrangeaceae	Philadelphus affinis			
Hydrangeaceae	Philadelphus coulteri			
Hydrangeaceae	Philadelphus mexicanus			
Hydrocharitaceae	Elodea densa			
Hydrocharitaceae	Najas guadalupensis			
Hydrocharitaceae	Najas guadalupensis subsp. guadalupensis			
Hydrocharitaceae	Najas wrightiana			
Hydroleaceae	Hydrolea spinosa			
Hymenophyllaceae	Hymenophyllum abruptum			EN
Hymenophyllaceae	Hymenophyllum asplenioides			VU
Hymenophyllaceae	Hymenophyllum brevistipes			EN
Hymenophyllaceae	Hymenophyllum crispum			EN
Hymenophyllaceae	Hymenophyllum ectocarpon			EN
Hymenophyllaceae	Hymenophyllum fendlerianum			VU
Hymenophyllaceae	Hymenophyllum fragile			
Hymenophyllaceae	Hymenophyllum fucoides			
Hymenophyllaceae	Hymenophyllum hirsutum			
Hymenophyllaceae	Hymenophyllum lanatum			VU
Hymenophyllaceae	Hymenophyllum maxonii			VU

Hymenophyllaceae	Hymenophyllum microcarpum		
Hymenophyllaceae	Hymenophyllum myriocarpum		
Hymenophyllaceae	Hymenophyllum paucicarpum	EN	
Hymenophyllaceae	Hymenophyllum polyanthos		
Hymenophyllaceae	Hymenophyllum pulchellum		
Hymenophyllaceae	Hymenophyllum tegularis		
Hymenophyllaceae	Hymenophyllum trapezoidale		
Hymenophyllaceae	Hymenophyllum tunbrigense		
Hymenophyllaceae	Hymenophyllum undulatum	VU	
Hymenophyllaceae	Trichomanes capillaceum		
Hymenophyllaceae	Trichomanes collariatum		
Hymenophyllaceae	Trichomanes crispum		
Hymenophyllaceae	Trichomanes diversifrons		
Hymenophyllaceae	Trichomanes ekmanii	EN	
Hymenophyllaceae	Trichomanes galeottii		
Hymenophyllaceae	Trichomanes godmanii	EN	
Hymenophyllaceae	Trichomanes holopterum	CR	YES
Hymenophyllaceae	Trichomanes hymenoides		
Hymenophyllaceae	Trichomanes krausii		
Hymenophyllaceae	Trichomanes petersii	EN	
Hymenophyllaceae	Trichomanes pinnatum		
Hymenophyllaceae	Trichomanes polypodioides		
Hymenophyllaceae	Trichomanes pyxidiferum	EN	
Hymenophyllaceae	Trichomanes radicans		
Hymenophyllaceae	Trichomanes reptans		
Hymenophyllaceae	Trichomanes rigidum		
Hypericaceae	Hypericum calcicola	EN	
Hypericaceae	Hypericum epigeium	EN	
Hypericaceae	Hypericum erythreae	CR	
Hypericaceae	Hypericum gymnanthum	EN	
Hypericaceae	Hypericum hypericoides		
Hypericaceae	Hypericum hypericoides subsp. hypericoides		
Hypericaceae	Hypericum hypericoides subsp. multicaule	EN	
Hypericaceae	Hypericum matudae	EN	

Hypericaceae	Hypericum moranense			
Hypericaceae	Hypericum mutilum			
Hypericaceae	Hypericum pauciflorum			
Hypericaceae	Hypericum philonotis			
Hypericaceae	Hypericum pratense			
Hypericaceae	Hypericum pumilum subsp. diffusum		VU	
Hypericaceae	Hypericum silenoides			
Hypericaceae	Hypericum silenoides subsp. silenoides			
Hypericaceae	Hypericum thesiifolium			
Hypericaceae	Vismia baccifera			
Hypericaceae	Vismia baccifera subsp. baccifera			
Hypericaceae	Vismia baccifera subsp. dealbata		CR	
Hypericaceae	Vismia camparaguey			
Hypericaceae	Vismia ferruginea		EN	
Hypoxidaceae	Curculigo scorzonerifolia			
Hypoxidaceae	Hypoxis decumbens			
Hypoxidaceae	Hypoxis humilis			
Hypoxidaceae	Hypoxis mexicana			
Hypoxidaceae	Hypoxis potosina			
Hypoxidaceae	Hypoxis tepicensis			
Icacinaceae	Mappia multiflora		EN	
Icacinaceae	Mappia racemosa	VU	VU	
Iridaceae	Alophia drummondii			
Iridaceae	Cipura campanulata			
Iridaceae	Cipura paludosa			
Iridaceae	Crocosmia × crocosmiiflora			
Iridaceae	Crocosmia aurea		EN	
Iridaceae	Eleutherine bulbosa			
Iridaceae	Eleutherine latifolia			
Iridaceae	Gladiolus dalenii subsp. dalenii		EN	
Iridaceae	Gladiolus liliaceus		CR	YES
Iridaceae	Iris × germanica			
Iridaceae	Larentia mexicana			
Iridaceae	Orthrosanthus chimboracensis			

Iridaceae	Orthrosanthus exsertus			
Iridaceae	Orthrosanthus monadelphus			
Iridaceae	Sisyrinchium angustifolium			
Iridaceae	Sisyrinchium angustissimum			
Iridaceae	Sisyrinchium arguellesiae		EN	
Iridaceae	Sisyrinchium bellum			
Iridaceae	Sisyrinchium biforme			
Iridaceae	Sisyrinchium cernuum			
Iridaceae	Sisyrinchium convolutum			
Iridaceae	Sisyrinchium exalatum		VU	
Iridaceae	Sisyrinchium johnstonii		VU	
Iridaceae	Sisyrinchium macrophyllum			
Iridaceae	Sisyrinchium micranthum			
Iridaceae	Sisyrinchium scabrum			
Iridaceae	Sisyrinchium schaffneri			
Iridaceae	Sisyrinchium serrulatum			
Iridaceae	Sisyrinchium subalpinum		CR	YES
Iridaceae	Sisyrinchium tenuifolium			
Iridaceae	Sisyrinchium tinctorium			
Iridaceae	Sisyrinchium tolucense			
Iridaceae	Sisyrinchium vaginatum subsp. vaginatum		VU	
Iridaceae	Tigridia alpestris		VU	
Iridaceae	Tigridia ehrenbergii			
Iridaceae	Tigridia ehrenbergii subsp. flaviglandifera		VU	
Iridaceae	Tigridia galanthoides			
Iridaceae	Tigridia hallbergii			
Iridaceae	Tigridia hallbergii subsp. hallbergii		VU	
Iridaceae	Tigridia heliantha			
Iridaceae	Tigridia immaculata		VU	
Iridaceae	Tigridia inusitata	NT		
Iridaceae	Tigridia multiflora			
Iridaceae	Tigridia oaxacana	EW		
Iridaceae	Tigridia orthantha	NT		
Iridaceae	Tigridia pavonia			

Iridaceae	Tigridia rzedowskiana			EN
Iridaceae	Tigridia seleriana			VU
Iridaceae	Tigridia vanhouttei			
Iridaceae	Tigridia vanhouttei subsp. roldanii			VU
Iridaceae	Trimezia gracilis			
Iridaceae	Trimezia martinicensis			
Iridaceae	Trimezia steyermarkii			
Iridaceae	Trimezia variegata			
Isoetaceae	Isoetes mexicana			
Iteaceae	Pterostemon mexicanus			
Iteaceae	Pterostemon rotundifolius			
Juglandaceae	Alfaroa mexicana	NT	VU	
Juglandaceae	Carya illinoinensis			
Juglandaceae	Carya myristiciformis			
Juglandaceae	Carya ovata			
Juglandaceae	Carya ovata var. mexicana			
Juglandaceae	Carya palmeri		VU	
Juglandaceae	Juglans mollis			
Juglandaceae	Juglans pyriformis	VU	EN	
Juglandaceae	Oreomunnea mexicana	VU		
Juncaceae	Juncus acuminatus			
Juncaceae	Juncus aemulans			
Juncaceae	Juncus arcticus			
Juncaceae	Juncus bufonius			
Juncaceae	Juncus chiapasensis			EN
Juncaceae	Juncus debilis			VU
Juncaceae	Juncus dichotomus			
Juncaceae	Juncus ebracteatus			
Juncaceae	Juncus effusus			
Juncaceae	Juncus liebmannii			
Juncaceae	Juncus liebmannii var. polycephalus			VU
Juncaceae	Juncus marginatus			
Juncaceae	Juncus microcephalus			
Juncaceae	Juncus tenuis			

Juncaceae	Luzula caricina		
Juncaceae	Luzula denticulata		
Koeberliniaceae	Koeberlinia spinosa		
Krameriaceae	Krameria cytisoides		
Krameriaceae	Krameria ixine		
Krameriaceae	Krameria pauciflora		
Krameriaceae	Krameria revoluta		
Krameriaceae	Krameria secundiflora		
Lacistemataceae	Lacistema aggregatum		
Lamiaceae	Aegiphila costaricensis		
Lamiaceae	Aegiphila deppeana		
Lamiaceae	Aegiphila elata		
Lamiaceae	Aegiphila falcata		EN
Lamiaceae	Aegiphila fasciculata	VU	EN
Lamiaceae	Aegiphila monstrosa	VU	
Lamiaceae	Aegiphila skutchii	VU	VU
Lamiaceae	Aegiphila wigandioides	EN	VU
Lamiaceae	Agastache mexicana		
Lamiaceae	Asterohyptis stellulata		
Lamiaceae	Callicarpa acuminata		
Lamiaceae	Callicarpa acuminata var. pringlei		
Lamiaceae	Cantinoa americana		
Lamiaceae	Cantinoa mutabilis		
Lamiaceae	Catoferia capitata		
Lamiaceae	Catoferia chiapensis		
Lamiaceae	Clerodendrum × speciosum		VU
Lamiaceae	Clerodendrum bungei		
Lamiaceae	Clerodendrum chinense		
Lamiaceae	Clerodendrum japonicum		
Lamiaceae	Clerodendrum thomsoniae		
Lamiaceae	Clerodendrum umbellatum		EN
Lamiaceae	Clinopodium brownei		
Lamiaceae	Clinopodium macrostemum		
Lamiaceae	Clinopodium mexicanum		

Lamiaceae	Clinopodium selerianum		
Lamiaceae	Coleus amboinicus		
Lamiaceae	Coleus scutellarioides		
Lamiaceae	Condea albida		
Lamiaceae	Condea subtilis		
Lamiaceae	Condea tomentosa		
Lamiaceae	Condea verticillata		
Lamiaceae	Cornutia pyramidata		
Lamiaceae	Cunila leucantha	VU	
Lamiaceae	Cunila polyantha		
Lamiaceae	Gmelina arborea		
Lamiaceae	Hedeoma acinoides	VU	
Lamiaceae	Hedeoma costata		
Lamiaceae	Hedeoma costata var. costata		
Lamiaceae	Hedeoma drummondii		
Lamiaceae	Hedeoma palmeri		
Lamiaceae	Holmskioldia sanguinea		
Lamiaceae	Hyptis atrorubens		
Lamiaceae	Hyptis brevipes		
Lamiaceae	Hyptis capitata		
Lamiaceae	Hyptis conferta		
Lamiaceae	Hyptis intermedia	CR	YES
Lamiaceae	Hyptis lantanifolia		
Lamiaceae	Hyptis recurvata		
Lamiaceae	Leonotis nepetifolia		
Lamiaceae	Leonurus japonicus		
Lamiaceae	Leonurus sibiricus		
Lamiaceae	Lepechinia caulescens		
Lamiaceae	Lepechinia mexicana		
Lamiaceae	Lepechinia schiedeana		
Lamiaceae	Marrubium vulgare		
Lamiaceae	Marsypianthes chamaedrys		
Lamiaceae	Melissa officinalis	VU	
Lamiaceae	Mentha × piperita		

Lamiaceae	Mentha × rotundifolia		
Lamiaceae	Mentha aquatica		
Lamiaceae	Mentha arvensis		
Lamiaceae	Mentha spicata		
Lamiaceae	Mentha spicata subsp. spicata		
Lamiaceae	Mesosphaerum oblongifolium		
Lamiaceae	Mesosphaerum pectinatum		
Lamiaceae	Mesosphaerum suaveolens		
Lamiaceae	Mesosphaerum urticoides		
Lamiaceae	Monarda bartlettii		
Lamiaceae	Monarda fistulosa		
Lamiaceae	Ocimum × africanum	EN	
Lamiaceae	Ocimum basilicum		
Lamiaceae	Ocimum campechianum		
Lamiaceae	Ocimum carnosum		
Lamiaceae	Origanum majorana		
Lamiaceae	Origanum vulgare		
Lamiaceae	Pogostemon cablin	VU	
Lamiaceae	Poliomintha marifolia		
Lamiaceae	Prunella vulgaris		
Lamiaceae	Salvia adenophora		
Lamiaceae	Salvia albiflora		
Lamiaceae	Salvia altimitrata	CR	YES
Lamiaceae	Salvia anastomosans		
Lamiaceae	Salvia assurgens		
Lamiaceae	Salvia axillaris		
Lamiaceae	Salvia biserrata	VU	
Lamiaceae	Salvia blepharophylla		
Lamiaceae	Salvia breviflora		
Lamiaceae	Salvia buchananii	EN	
Lamiaceae	Salvia cacaliifolia		
Lamiaceae	Salvia candicans		
Lamiaceae	Salvia carnea		
Lamiaceae	Salvia carranzae	EN	

Lamiaceae	Salvia caudata	VU	
Lamiaceae	Salvia chamaedryoides		
Lamiaceae	Salvia chiapensis		
Lamiaceae	Salvia cinnabarina		
Lamiaceae	Salvia circinnata		
Lamiaceae	Salvia coccinea		
Lamiaceae	Salvia collinsii	VU	
Lamiaceae	Salvia compsostachys		
Lamiaceae	Salvia concolor		
Lamiaceae	Salvia connivens		
Lamiaceae	Salvia dichlamys		
Lamiaceae	Salvia divinorum		
Lamiaceae	Salvia duripes	EN	
Lamiaceae	Salvia eizi-matudae	EN	
Lamiaceae	Salvia elegans		
Lamiaceae	Salvia filicifolia	VU	
Lamiaceae	Salvia filipes		
Lamiaceae	Salvia flaccida	VU	
Lamiaceae	Salvia flaccidifolia		
Lamiaceae	Salvia fluviatilis		
Lamiaceae	Salvia fulgens		
Lamiaceae	Salvia gesneriiflora		
Lamiaceae	Salvia gracilipes		
Lamiaceae	Salvia guevarae	CR	YES
Lamiaceae	Salvia helianthemifolia		
Lamiaceae	Salvia herbacea		
Lamiaceae	Salvia hidalgensis	EN	
Lamiaceae	Salvia hispanica		
Lamiaceae	Salvia holwayi		
Lamiaceae	Salvia inconspicua		
Lamiaceae	Salvia infuscata		
Lamiaceae	Salvia involucrata		
Lamiaceae	Salvia iodantha		
Lamiaceae	Salvia ionocalyx	EN	

Lamiaceae	Salvia karwinskii	
Lamiaceae	Salvia keerlii	
Lamiaceae	Salvia kellermanii	EN
Lamiaceae	Salvia laevis	
Lamiaceae	Salvia languidula	
Lamiaceae	Salvia lasiantha	
Lamiaceae	Salvia lasiocephala	
Lamiaceae	Salvia lavanduloides	
Lamiaceae	Salvia leptostachys	
Lamiaceae	Salvia leucantha	
Lamiaceae	Salvia leucophylla	
Lamiaceae	Salvia lineata	
Lamiaceae	Salvia littae	
Lamiaceae	Salvia longispicata	
Lamiaceae	Salvia longistyla	
Lamiaceae	Salvia melissodora	
Lamiaceae	Salvia mexicana	
Lamiaceae	Salvia microphylla	
Lamiaceae	Salvia miniata	
Lamiaceae	Salvia misella	
Lamiaceae	Salvia mocinoi	
Lamiaceae	Salvia nervata	
Lamiaceae	Salvia oaxacana	
Lamiaceae	Salvia occidentalis	
Lamiaceae	Salvia pansamalensis	VU
Lamiaceae	Salvia patens	
Lamiaceae	Salvia plurispicata	
Lamiaceae	Salvia polystachia	
Lamiaceae	Salvia protracta	
Lamiaceae	Salvia prunelloides	
Lamiaceae	Salvia puberula	
Lamiaceae	Salvia pubescens	
Lamiaceae	Salvia pulchella	
Lamiaceae	Salvia purpurea	

Lamiaceae	Salvia pusilla		
Lamiaceae	Salvia recurva		
Lamiaceae	Salvia regla		
Lamiaceae	Salvia reptans		
Lamiaceae	Salvia richardsonii	CR	YES
Lamiaceae	Salvia rosmarinus		
Lamiaceae	Salvia rypara		
Lamiaceae	Salvia sacculus		
Lamiaceae	Salvia semiatrata		
Lamiaceae	Salvia setulosa		
Lamiaceae	Salvia shannonii		
Lamiaceae	Salvia sharpii		
Lamiaceae	Salvia splendens		
Lamiaceae	Salvia stolonifera		
Lamiaceae	Salvia subrubens	CR	YES
Lamiaceae	Salvia thymoides		
Lamiaceae	Salvia thyrsiflora		
Lamiaceae	Salvia tiliifolia		
Lamiaceae	Salvia tonalensis		
Lamiaceae	Salvia tricuspidata		
Lamiaceae	Salvia tubifera		
Lamiaceae	Salvia uliginosa		
Lamiaceae	Salvia univerticillata	EN	
Lamiaceae	Salvia urica		
Lamiaceae	Salvia urolepis		
Lamiaceae	Salvia vitifolia		
Lamiaceae	Salvia wagneriana		
Lamiaceae	Salvia whitefoordiae	EN	
Lamiaceae	Salvia xalapensis		
Lamiaceae	Scutellaria aurea	EN	
Lamiaceae	Scutellaria chalicophila		
Lamiaceae	Scutellaria cordifolia		
Lamiaceae	Scutellaria dumetorum		
Lamiaceae	Scutellaria formosa		

Lamiaceae	Scutellaria fruticetorum	VU
Lamiaceae	Scutellaria guatemalensis	
Lamiaceae	Scutellaria havanensis	EN
Lamiaceae	Scutellaria longifolia	VU
Lamiaceae	Scutellaria lutea	EN
Lamiaceae	Scutellaria macra	VU
Lamiaceae	Scutellaria orichalcea	
Lamiaceae	Scutellaria orizabensis	EN
Lamiaceae	Scutellaria pseudocoerulea	
Lamiaceae	Scutellaria purpurascens	VU
Lamiaceae	Scutellaria racemosa	
Lamiaceae	Scutellaria seleriana	
Lamiaceae	Scutellaria vitifolia	
Lamiaceae	Stachys agraria	
Lamiaceae	Stachys albotomentosa	
Lamiaceae	Stachys boraginoides	
Lamiaceae	Stachys coccinea	
Lamiaceae	Stachys costaricensis	
Lamiaceae	Stachys eriantha	
Lamiaceae	Stachys keerlii	
Lamiaceae	Stachys lindenii	
Lamiaceae	Stachys pilosissima	
Lamiaceae	Stachys radicans	
Lamiaceae	Stachys rotundifolia	
Lamiaceae	Stachys sanchezii	
Lamiaceae	Stachys torresii	EN
Lamiaceae	Tectona grandis	
Lamiaceae	Teucrium cubense	
Lamiaceae	Teucrium vesicarium	
Lamiaceae	Thymus vulgaris	
Lamiaceae	Vitex gaumeri	
Lamiaceae	Vitex hemsleyi	
Lamiaceae	Vitex mollis	
Lamiaceae	Volkameria ligustrina	

Lauraceae	Aiouea areolata	VU		
Lauraceae	Aiouea bractefoliacea	VU		
Lauraceae	Aiouea breedlovei	EN	VU	
Lauraceae	Aiouea brenesii		CR	
Lauraceae	Aiouea chiapensis	VU		
Lauraceae	Aiouea cinnamomoidea		EN	
Lauraceae	Aiouea effusa			
Lauraceae	Aiouea grisebachii			
Lauraceae	Aiouea maya		VU	
Lauraceae	Aiouea montana			
Lauraceae	Aiouea pachypoda			
Lauraceae	Aiouea salicifolia	EN		
Lauraceae	Aiouea zapatae	VU		
Lauraceae	Beilschmiedia anay	EN		
Lauraceae	Beilschmiedia hondurensis			
Lauraceae	Beilschmiedia mexicana			
Lauraceae	Beilschmiedia ovalioides	VU	VU	
Lauraceae	Beilschmiedia ovalis			
Lauraceae	Beilschmiedia pendula		VU	
Lauraceae	Beilschmiedia riparia			
Lauraceae	Beilschmiedia steyermarkii	EN	CR	YES
Lauraceae	Beilschmiedia zapoteoides		EN	YES
Lauraceae	Cassytha filiformis			
Lauraceae	Cinnamomum verum			
Lauraceae	Damburneya ambigens			
Lauraceae	Damburneya colorata		VU	
Lauraceae	Damburneya coriacea			
Lauraceae	Damburneya gentlei			
Lauraceae	Damburneya inconspicua			
Lauraceae	Damburneya leucocome	EN		
Lauraceae	Damburneya longicaudata			
Lauraceae	Damburneya martinicensis			
Lauraceae	Damburneya matudae	EN		
Lauraceae	Damburneya nitida			

Lauraceae	Damburneya rudis		VU	
Lauraceae	Damburneya salicifolia			
Lauraceae	Licaria alata			
Lauraceae	Licaria areolata			EN
Lauraceae	Licaria campechiana			
Lauraceae	Licaria capitata			
Lauraceae	Licaria caudata			
Lauraceae	Licaria cervantesii			
Lauraceae	Licaria excelsa			
Lauraceae	Licaria glaberrima			
Lauraceae	Licaria misantlae			
Lauraceae	Licaria multinervis			EN
Lauraceae	Licaria peckii			
Lauraceae	Licaria triandra			
Lauraceae	Licaria velutina		VU	VU
Lauraceae	Litsea glaucescens	EN		
Lauraceae	Litsea muellerorum			
Lauraceae	Litsea parvifolia			
Lauraceae	Litsea pringlei			
Lauraceae	Mespilodaphne macrophylla			
Lauraceae	Mespilodaphne veraguensis			
Lauraceae	Nectandra cissiflora			EN
Lauraceae	Nectandra cuspidata			
Lauraceae	Nectandra globosa			
Lauraceae	Nectandra hihua			
Lauraceae	Nectandra membranacea			
Lauraceae	Nectandra rubriflora			
Lauraceae	Nectandra sanguinea			
Lauraceae	Nectandra turbacensis			
Lauraceae	Nectandra villosa			
Lauraceae	Ocotea acuminatissima			
Lauraceae	Ocotea bernoulliana			
Lauraceae	Ocotea betazensis			
Lauraceae	Ocotea botrantha			

Lauraceae	Ocotea bourgeauviana		
Lauraceae	Ocotea chiapensis		
Lauraceae	Ocotea chrysobalanoides		EN
Lauraceae	Ocotea congregata		VU
Lauraceae	Ocotea effusa		
Lauraceae	Ocotea heydeana		
Lauraceae	Ocotea klotzschiana		
Lauraceae	Ocotea laetevirens		
Lauraceae	Ocotea leptobotra		
Lauraceae	Ocotea leucoxylon		
Lauraceae	Ocotea macrophylla		
Lauraceae	Ocotea magnifolia		VU
Lauraceae	Ocotea matudae		VU
Lauraceae	Ocotea parvula		VU
Lauraceae	Ocotea pittieri		EN
Lauraceae	Ocotea psychotrioides		
Lauraceae	Ocotea puberula		
Lauraceae	Ocotea purpurea		
Lauraceae	Ocotea rovirosae		VU
Lauraceae	Ocotea salvinii		VU
Lauraceae	Ocotea sinuata		
Lauraceae	Ocotea standleyi		
Lauraceae	Ocotea stenoneura		EN
Lauraceae	Ocotea tampicensis		
Lauraceae	Ocotea tonii		
Lauraceae	Ocotea truncata		
Lauraceae	Ocotea uxpanapana	VU	
Lauraceae	Ocotea vanderwerffii	VU	
Lauraceae	Persea albida	EN	
Lauraceae	Persea americana		
Lauraceae	Persea caerulea		VU
Lauraceae	Persea chamissonis	EN	
Lauraceae	Persea cinerascens	EN	
Lauraceae	Persea donnell-smithii	VU	

Lauraceae	Persea hintonii		VU		
Lauraceae	Persea liebmannii				
Lauraceae	Persea longipes		EN		
Lauraceae	Persea pallescens		EN		
Lauraceae	Persea purpusii				
Lauraceae	Persea rufescens		EN		
Lauraceae	Persea schiedeana		EN		
Lauraceae	Persea standleyi			EN	
Lauraceae	Persea vesticula				
Lauraceae	Phoebe mollis		EN	EN	
Lecythidaceae	Eschweilera mexicana		VU	EN	
Lentibulariaceae	Pinguicula calderoniae			EN	
Lentibulariaceae	Pinguicula crenatiloba				
Lentibulariaceae	Pinguicula emarginata			VU	
Lentibulariaceae	Pinguicula gigantea			CR	YES
Lentibulariaceae	Pinguicula gracilis				
Lentibulariaceae	Pinguicula heterophylla				
Lentibulariaceae	Pinguicula laueana			EN	
Lentibulariaceae	Pinguicula laxifolia			EN	
Lentibulariaceae	Pinguicula lilacina				
Lentibulariaceae	Pinguicula moranensis				
Lentibulariaceae	Pinguicula moranensis var. moranensis				
Lentibulariaceae	Pinguicula parvifolia				
Lentibulariaceae	Pinguicula robertiana			EN	YES
Lentibulariaceae	Pinguicula sharpii			EN	
Lentibulariaceae	Utricularia foliosa				
Lentibulariaceae	Utricularia gibba				
Lentibulariaceae	Utricularia jamesoniana			CR	YES
Lentibulariaceae	Utricularia pusilla				
Lentibulariaceae	Utricularia subulata				
Liliaceae	Calochortus balsensis				
Liliaceae	Calochortus barbatus				
Liliaceae	Calochortus ghiesbreghtii				
Liliaceae	Calochortus nigrescens	NT			

Liliaceae	Lilium longiflorum	EN
Linaceae	Linum guatemalense	EN
Linaceae	Linum lasiocarpum	
Linaceae	Linum mexicanum	
Linaceae	Linum nelsonii	
Linaceae	Linum orizabae	
Linaceae	Linum pringlei	
Linaceae	Linum rupestre	
Linaceae	Linum scabrellum	
Linaceae	Linum schiedeanum	
Linaceae	Linum tenellum	
Linaceae	Linum usitatissimum	
Linderniaceae	Bonnaya antipoda	
Linderniaceae	Lindernia dubia	
Linderniaceae	Torenia fournieri	
Linderniaceae	Vandellia diffusa	VU
Lindsaeaceae	Lindsaea arcuata	VU
Lindsaeaceae	Lindsaea lancea	
Lindsaeaceae	Lindsaea portoricensis	VU
Lindsaeaceae	Lindsaea quadrangularis subsp. antillensis	EN
Lindsaeaceae	Lindsaea stricta	VU
Lindsaeaceae	Lindsaea subalata	EN
Lindsaeaceae	Odontosoria schlechtendalii	
Lindsaeaceae	Sphenomeris clavata	EN
Loasaceae	Cevallia sinuata	
Loasaceae	Eucnide bartonioides	
Loasaceae	Eucnide chiapasana	VU
Loasaceae	Eucnide grandiflora	
Loasaceae	Eucnide hirta	
Loasaceae	Gronovia scandens	
Loasaceae	Klaprothia fasciculata	
Loasaceae	Mentzelia arborescens	
Loasaceae	Mentzelia aspera	
Loasaceae	Mentzelia conzattii	VU

Loasaceae	Mentzelia hispida	
Loasaceae	Nasa triphylla	
Loasaceae	Nasa triphylla subsp. rudis	
Loasaceae	Schismocarpus matudae	VU
Loganiaceae	Mitreola petiolata	
Loganiaceae	Spigelia anthelmia	
Loganiaceae	Spigelia carnosa	VU
Loganiaceae	Spigelia chiapensis	EN
Loganiaceae	Spigelia coelostylioides	
Loganiaceae	Spigelia humboldtiana	
Loganiaceae	Spigelia longiflora	
Loganiaceae	Spigelia palmeri	
Loganiaceae	Spigelia pygmaea	
Loganiaceae	Spigelia speciosa	
Loganiaceae	Spigelia splendens	
Loganiaceae	Strychnos brachistantha	
Loganiaceae	Strychnos nigricans	
Loganiaceae	Strychnos panamensis	
Loganiaceae	Strychnos tabascana	
Lonchitidaceae	Lonchitis hirsuta	
Loranthaceae	Cladocolea andrieuxii	
Loranthaceae	Cladocolea diversifolia	
Loranthaceae	Cladocolea glauca	
Loranthaceae	Cladocolea grahamii	
Loranthaceae	Cladocolea pedicellata	
Loranthaceae	Oryctanthus cordifolius	
Loranthaceae	Passovia pyrifolia	VU
Loranthaceae	Peristethium phaneroneurum	EN
Loranthaceae	Psittacanthus angustifolius	VU
Loranthaceae	Psittacanthus auriculatus	
Loranthaceae	Psittacanthus calyculatus	
Loranthaceae	Psittacanthus mayanus	
Loranthaceae	Psittacanthus minor	CR
Loranthaceae	Psittacanthus palmeri	

Loranthaceae	Psittacanthus ramiflorus			
Loranthaceae	Psittacanthus rhynchanthus			
Loranthaceae	Psittacanthus schiedeanus			
Loranthaceae	Struthanthus capitatus			
Loranthaceae	Struthanthus cassythoides			
Loranthaceae	Struthanthus crassipes			
Loranthaceae	Struthanthus deppeanus			
Loranthaceae	Struthanthus hartwegii			
Loranthaceae	Struthanthus interruptus			
Loranthaceae	Struthanthus macrostachyus		VU	
Loranthaceae	Struthanthus marginatus			
Loranthaceae	Struthanthus orbicularis			
Loranthaceae	Struthanthus quercicola			
Loranthaceae	Struthanthus tacanensis		VU	
Lycopodiaceae	Huperzia cuernavacensis			
Lycopodiaceae	Huperzia dichotoma	VU		
Lycopodiaceae	Huperzia eversa			
Lycopodiaceae	Huperzia hippuridea		VU	
Lycopodiaceae	Huperzia linifolia			
Lycopodiaceae	Huperzia myrsinites			
Lycopodiaceae	Huperzia orizabae			
Lycopodiaceae	Huperzia pithyoides			
Lycopodiaceae	Huperzia pringlei			
Lycopodiaceae	Huperzia reflexa			
Lycopodiaceae	Huperzia taxifolia			
Lycopodiaceae	Huperzia verticillata		VU	
Lycopodiaceae	Lycopodiella alopecuroides		EN	YES
Lycopodiaceae	Lycopodiella andicola		CR	YES
Lycopodiaceae	Lycopodiella cernua			
Lycopodiaceae	Lycopodium clavatum			
Lycopodiaceae	Lycopodium thyoides			
Lythraceae	Adenaria floribunda			
Lythraceae	Ammannia auriculata			
Lythraceae	Ammannia coccinea			

Lythraceae	Ammannia robusta	
Lythraceae	Cuphea aequipetala	
Lythraceae	Cuphea angustifolia	
Lythraceae	Cuphea appendiculata	
Lythraceae	Cuphea avigera	
Lythraceae	Cuphea calaminthifolia	
Lythraceae	Cuphea calophylla	
Lythraceae	Cuphea carthagenensis	
Lythraceae	Cuphea cyanea	
Lythraceae	Cuphea decandra	
Lythraceae	Cuphea elliptica	
Lythraceae	Cuphea glossostoma	
Lythraceae	Cuphea hookeriana	
Lythraceae	Cuphea hyssopifolia	
Lythraceae	Cuphea ignea	
Lythraceae	Cuphea intermedia	
Lythraceae	Cuphea jorullensis	
Lythraceae	Cuphea laminuligera	
Lythraceae	Cuphea lanceolata	
Lythraceae	Cuphea leptopoda	
Lythraceae	Cuphea llavea	
Lythraceae	Cuphea micropetala	
Lythraceae	Cuphea mimuloides	VU
Lythraceae	Cuphea nitidula	
Lythraceae	Cuphea nudicostata	
Lythraceae	Cuphea pinetorum	
Lythraceae	Cuphea procumbens	
Lythraceae	Cuphea racemosa	
Lythraceae	Cuphea retroscabra	VU
Lythraceae	Cuphea salicifolia	
Lythraceae	Cuphea salvadorensis	
Lythraceae	Cuphea schumannii	
Lythraceae	Cuphea subuligera	
Lythraceae	Cuphea tetrapetala	VU

Lythraceae	Cuphea tolucana				
Lythraceae	Cuphea utriculosa				
Lythraceae	Cuphea vesiculigera				
Lythraceae	Cuphea wrightii				
Lythraceae	Ginoria nudiflora		VU		
Lythraceae	Heimia salicifolia				
Lythraceae	Lafoensia punicifolia				
Lythraceae	Lagerstroemia indica				
Lythraceae	Lawsonia inermis				
Lythraceae	Lythrum alatum				
Lythraceae	Lythrum alatum var. lanceolatum				
Lythraceae	Lythrum californicum				
Lythraceae	Lythrum gracile				
Lythraceae	Lythrum vulneraria				
Lythraceae	Punica granatum				
Lythraceae	Rotala ramosior				
Magnoliaceae	Magnolia grandiflora				
Magnoliaceae	Magnolia krusei		EN	EN	
Magnoliaceae	Magnolia lacandonica		CR	VU	
Magnoliaceae	Magnolia macrophylla			EN	
Magnoliaceae	Magnolia macrophylla var. dealbata	VU			
Magnoliaceae	Magnolia mayae		CR	EN	YES
Magnoliaceae	Magnolia mexicana	VU	VU		
Magnoliaceae	Magnolia montebelloensis			CR	YES
Magnoliaceae	Magnolia perezfarrerae		EN	CR	YES
Magnoliaceae	Magnolia poasana				
Magnoliaceae	Magnolia rzedowskiana		EN	CR	YES
Magnoliaceae	Magnolia schiedeana	VU	VU		
Magnoliaceae	Magnolia sharpii		EN		
Magnoliaceae	Magnolia tamaulipana		EN		
Magnoliaceae	Magnolia yoroconte		VU	VU	
Malpighiaceae	Adelphia hiraea				
Malpighiaceae	Aspicarpa hirtella				
Malpighiaceae	Banisteriopsis caapi			CR	

Malpighiaceae	Banisteriopsis elegans	EN	
Malpighiaceae	Banisteriopsis muricata		
Malpighiaceae	Bunchosia biocellata		
Malpighiaceae	Bunchosia breedlovei	EN	
Malpighiaceae	Bunchosia canescens		
Malpighiaceae	Bunchosia caroli		
Malpighiaceae	Bunchosia gracilis		
Malpighiaceae	Bunchosia guatemalensis		
Malpighiaceae	Bunchosia lancifolia	EN	
Malpighiaceae	Bunchosia lindeniana		
Malpighiaceae	Bunchosia macrophylla	EN	
Malpighiaceae	Bunchosia matudae		
Malpighiaceae	Bunchosia montana		
Malpighiaceae	Bunchosia nitida		
Malpighiaceae	Bunchosia odorata	VU	
Malpighiaceae	Bunchosia palmeri		
Malpighiaceae	Bunchosia swartziana		
Malpighiaceae	Byrsonima crassifolia		
Malpighiaceae	Byrsonima roigii		
Malpighiaceae	Callaeum chiapense	EN	
Malpighiaceae	Callaeum macropterum		
Malpighiaceae	Callaeum malpighioides		
Malpighiaceae	Callaeum nicaraguense	EN	
Malpighiaceae	Callaeum septentrionale		
Malpighiaceae	Carolus sinemariensis		
Malpighiaceae	Diplopterys mexicana	CR	YES
Malpighiaceae	Echinopterys eglandulosa		
Malpighiaceae	Galphimia glauca		
Malpighiaceae	Galphimia gracilis		
Malpighiaceae	Galphimia paniculata		
Malpighiaceae	Galphimia speciosa		
Malpighiaceae	Gaudichaudia albida		
Malpighiaceae	Gaudichaudia cynanchoides		
Malpighiaceae	Gaudichaudia galeottiana		

Malpighiaceae	Gaudichaudia hexandra	EN	
Malpighiaceae	Heteropterys brachiata		
Malpighiaceae	Heteropterys cotinifolia		
Malpighiaceae	Heteropterys laurifolia		
Malpighiaceae	Heteropterys macrostachya	EN	
Malpighiaceae	Heteropterys panamensis	EN	
Malpighiaceae	Hiraea barclayana		
Malpighiaceae	Hiraea fagifolia		
Malpighiaceae	Hiraea obovata		
Malpighiaceae	Hiraea quapara	VU	
Malpighiaceae	Hiraea reclinata		
Malpighiaceae	Hiraea smilacina		
Malpighiaceae	Hiraea ternifolia	EN	
Malpighiaceae	Lasiocarpus ferrugineus		
Malpighiaceae	Lasiocarpus ovalifolius		
Malpighiaceae	Lasiocarpus salicifolius		
Malpighiaceae	Malpighia emarginata		
Malpighiaceae	Malpighia galeottiana		
Malpighiaceae	Malpighia glabra		
Malpighiaceae	Malpighia incana	CR	YES
Malpighiaceae	Malpighia leticiana	EN	
Malpighiaceae	Malpighia lundellii		
Malpighiaceae	Malpighia macrocarpa	VU	
Malpighiaceae	Malpighia mexicana		
Malpighiaceae	Malpighia mexicana subsp. guadalajarensis	EN	
Malpighiaceae	Malpighia novogaliciana		
Malpighiaceae	Malpighia ovata		
Malpighiaceae	Malpighia romeroana		
Malpighiaceae	Malpighia sessilifolia	VU	
Malpighiaceae	Malpighia souzae		
Malpighiaceae	Malpighia wendtii	VU	
Malpighiaceae	Mascagnia sepium		
Malpighiaceae	Mascagnia vacciniifolia		
Malpighiaceae	Niedenzuella sericea	EN	

Malpighiaceae	Psychopterys dipholiphylla			
Malpighiaceae	Psychopterys multiflora			
Malpighiaceae	Psychopterys ornata		EN	YES
Malpighiaceae	Psychopterys polycarpa			
Malpighiaceae	Psychopterys rivularis			
Malpighiaceae	Stigmaphyllon cordatum		EN	YES
Malpighiaceae	Stigmaphyllon dichotomum			
Malpighiaceae	Stigmaphyllon ellipticum			
Malpighiaceae	Stigmaphyllon lindenianum			
Malpighiaceae	Stigmaphyllon pseudopuberum			
Malpighiaceae	Stigmaphyllon retusum			
Malpighiaceae	Stigmaphyllon selerianum		VU	
Malpighiaceae	Tetrapterys andersonii		CR	YES
Malpighiaceae	Tetrapterys arcana			
Malpighiaceae	Tetrapterys argentea		VU	
Malpighiaceae	Tetrapterys discolor			
Malpighiaceae	Tetrapterys heterophylla			
Malpighiaceae	Tetrapterys mexicana			
Malpighiaceae	Tetrapterys nelsonii		EN	
Malpighiaceae	Tetrapterys schiedeana			
Malpighiaceae	Tetrapterys seleriana			
Malpighiaceae	Tetrapterys tinifolia			
Malvaceae	Abelmoschus esculentus			
Malvaceae	Abelmoschus manihot		VU	
Malvaceae	Abelmoschus moschatus			
Malvaceae	Abutilon andrieuxii			
Malvaceae	Abutilon bracteosum			
Malvaceae	Abutilon divaricatum			
Malvaceae	Abutilon dugesii			
Malvaceae	Abutilon fruticosum			
Malvaceae	Abutilon giganteum		VU	
Malvaceae	Abutilon grandidentatum	EN	VU	
Malvaceae	Abutilon hulseanum		VU	
Malvaceae	Abutilon hypoleucum			

Malvaceae	Abutilon incanum			
Malvaceae	Abutilon malacum			
Malvaceae	Abutilon mollicomum			
Malvaceae	Abutilon palmeri			
Malvaceae	Abutilon permolle			
Malvaceae	Abutilon procerum		EN	YES
Malvaceae	Abutilon reventum			
Malvaceae	Abutilon theophrasti			
Malvaceae	Abutilon trisulcatum			
Malvaceae	Abutilon viscosum			
Malvaceae	Alcea rosea			
Malvaceae	Allosidastrum hilarianum			
Malvaceae	Allosidastrum pyramidatum			
Malvaceae	Allowissadula pringlei			
Malvaceae	Anoda acerifolia			
Malvaceae	Anoda crenatiflora			
Malvaceae	Anoda cristata			
Malvaceae	Anoda paniculata			
Malvaceae	Anoda pedunculosa			
Malvaceae	Apeiba tibourbou			
Malvaceae	Ayenia aculeata			
Malvaceae	Ayenia berlandieri			
Malvaceae	Ayenia catalpifolia			
Malvaceae	Ayenia glabra			
Malvaceae	Ayenia magna			
Malvaceae	Ayenia micrantha			
Malvaceae	Ayenia pusilla			
Malvaceae	Ayenia sidifolia			
Malvaceae	Ayenia standleyi			
Malvaceae	Bakeridesia bakeriana	EN		
Malvaceae	Bakeridesia ferruginea			
Malvaceae	Bakeridesia gloriosa	VU		
Malvaceae	Bakeridesia integerrima			
Malvaceae	Bakeridesia nelsonii	VU	EN	

Malvaceae	Bakeridesia notolophium		EN		
Malvaceae	Bakeridesia pittieri				
Malvaceae	Bastardiastrum gracile				
Malvaceae	Bastardiastrum tricarpellatum				
Malvaceae	Bernoullia flammea				
Malvaceae	Briquetia spicata				
Malvaceae	Callianthe pachecoana		VU	CR	YES
Malvaceae	Callianthe picta				
Malvaceae	Callianthe purpusii				
Malvaceae	Callianthe striata				
Malvaceae	Callianthe tridens		EN	VU	
Malvaceae	Callianthe vexillaria			EN	
Malvaceae	Carpodiptera cubensis				
Malvaceae	Ceiba acuminata				
Malvaceae	Ceiba aesculifolia				
Malvaceae	Ceiba aesculifolia subsp. parvifolia				
Malvaceae	Ceiba pentandra				
Malvaceae	Ceiba schottii				
Malvaceae	Chiranthodendron pentadactylon	VU			
Malvaceae	Christiana africana			VU	
Malvaceae	Corchorus aestuans				
Malvaceae	Corchorus hirsutus			VU	
Malvaceae	Corchorus hirtus				
Malvaceae	Corchorus orinocensis				
Malvaceae	Corchorus siliquosus				
Malvaceae	Dendrosida batesii	VU	VU	EN	
Malvaceae	Dendrosida breedlovei	VU			
Malvaceae	Dendrosida sharpiana				
Malvaceae	Dombeya wallichii				
Malvaceae	Fuertesimalva jacens				
Malvaceae	Fuertesimalva limensis				
Malvaceae	Gaya hermannioides				
Malvaceae	Gaya minutiflora				
Malvaceae	Gaya occidentalis				

Malvaceae	Gossypium aridum	NT	VU	
Malvaceae	Gossypium barbadense			
Malvaceae	Gossypium gossypioides	NT	VU	
Malvaceae	Gossypium hirsutum	NT	VU	
Malvaceae	Gossypium irenaeum			EN
Malvaceae	Guazuma ulmifolia			
Malvaceae	Hampea integerrima			
Malvaceae	Hampea longipes			
Malvaceae	Hampea mexicana			
Malvaceae	Hampea montebellensis	VU	EN	
Malvaceae	Hampea nutricia			
Malvaceae	Hampea rovirosae			
Malvaceae	Hampea stipitata			
Malvaceae	Hampea tomentosa			
Malvaceae	Hampea trilobata			
Malvaceae	Helicteres baruensis			
Malvaceae	Helicteres guazumifolia			
Malvaceae	Heliocarpus americanus			
Malvaceae	Heliocarpus appendiculatus			
Malvaceae	Heliocarpus donnellsmithii			
Malvaceae	Heliocarpus mexicanus			
Malvaceae	Heliocarpus occidentalis			
Malvaceae	Heliocarpus pallidus			
Malvaceae	Heliocarpus terebinthinaceus			
Malvaceae	Heliocarpus velutinus			
Malvaceae	Herissantia crispa			
Malvaceae	Hibiscus acetosella			VU
Malvaceae	Hibiscus bifurcatus			
Malvaceae	Hibiscus clypeatus			
Malvaceae	Hibiscus costatus			
Malvaceae	Hibiscus furcellatus			
Malvaceae	Hibiscus lavateroides			
Malvaceae	Hibiscus longifilus			
Malvaceae	Hibiscus mutabilis			

Malvaceae	Hibiscus peripteroides		
Malvaceae	Hibiscus phoeniceus		
Malvaceae	Hibiscus poeppigii		
Malvaceae	Hibiscus purpusii		
Malvaceae	Hibiscus rosa-sinensis		
Malvaceae	Hibiscus sabdariffa		
Malvaceae	Hibiscus schizopetalus		
Malvaceae	Hibiscus spiralis	VU	
Malvaceae	Hibiscus tiliaceus		
Malvaceae	Hibiscus tiliaceus var. pernambucensis		
Malvaceae	Hibiscus uncinellus		
Malvaceae	Hochreutinera amplexifolia		
Malvaceae	Horsfordia rotundifolia		
Malvaceae	Kearnemalvastrum lacteum		
Malvaceae	Kearnemalvastrum subtriflorum		
Malvaceae	Kosteletzkya depressa		
Malvaceae	Kosteletzkya flavicentrum		EN
Malvaceae	Kosteletzkya tubiflora		
Malvaceae	Luehea candida		
Malvaceae	Luehea seemannii		
Malvaceae	Luehea speciosa		
Malvaceae	Malachra alceifolia		
Malvaceae	Malachra capitata		
Malvaceae	Malachra fasciata		
Malvaceae	Malva assurgentiflora		
Malvaceae	Malva neglecta		
Malvaceae	Malva nicaeensis		
Malvaceae	Malva parviflora		
Malvaceae	Malva pusilla		
Malvaceae	Malva sylvestris		
Malvaceae	Malvastrum americanum		
Malvaceae	Malvastrum corchorifolium		
Malvaceae	Malvastrum coromandelianum		
Malvaceae	Malvaviscus achanioides		

Malvaceae	Malvaviscus arboreus				
Malvaceae	Malvaviscus arboreus var. drummondii				
Malvaceae	Malvaviscus lanceolatus				
Malvaceae	Malvaviscus oaxacanus				
Malvaceae	Malvaviscus penduliflorus				
Malvaceae	Malvella leprosa				
Malvaceae	Melochia hirsuta				
Malvaceae	Melochia lupulina				
Malvaceae	Melochia manducata				
Malvaceae	Melochia nodiflora				
Malvaceae	Melochia nudiflora				
Malvaceae	Melochia oaxacana		VU		
Malvaceae	Melochia pyramidata				
Malvaceae	Melochia tomentosa				
Malvaceae	Melochia villosa				
Malvaceae	Modiola caroliniana				
Malvaceae	Mortoniodendron guatemalense	EN			
Malvaceae	Mortoniodendron hirsutum			CR	
Malvaceae	Mortoniodendron ocotense		EN	CR	YES
Malvaceae	Mortoniodendron palaciosii		CR	VU	
Malvaceae	Mortoniodendron pentagonum			EN	
Malvaceae	Mortoniodendron ruizii		CR	EN	
Malvaceae	Mortoniodendron sulcatum		VU	VU	
Malvaceae	Mortoniodendron vestitum				
Malvaceae	Neobrittonia acerifolia				
Malvaceae	Ochroma pyramidale				
Malvaceae	Pachira aquatica				
Malvaceae	Pavonia candida				
Malvaceae	Pavonia integrifolia			VU	
Malvaceae	Pavonia macdougallii				
Malvaceae	Pavonia malacophylla				
Malvaceae	Pavonia monticola				
Malvaceae	Pavonia paniculata				
Malvaceae	Pavonia purpusii				

Malvaceae	Pavonia schiedeana				
Malvaceae	Pavonia sidifolia				
Malvaceae	Pavonia spinifex				
Malvaceae	Pavonia uniflora				
Malvaceae	Phymosia anomala			EN	
Malvaceae	Phymosia rosea	NT			
Malvaceae	Phymosia umbellata				
Malvaceae	Physodium corymbosum				
Malvaceae	Pseudabutilon ellipticum				
Malvaceae	Pseudabutilon scabrum				
Malvaceae	Pseudobombax ellipticoideum			VU	
Malvaceae	Pseudobombax ellipticum				
Malvaceae	Pseudobombax palmeri				
Malvaceae	Quararibea asterolepis			EN	
Malvaceae	Quararibea funebris				
Malvaceae	Quararibea gentlei			EN	
Malvaceae	Quararibea yunckeri		CR		
Malvaceae	Quararibea yunckeri subsp. sessiliflora				
Malvaceae	Quararibea yunckeri subsp. veracruzana			CR	YES
Malvaceae	Reevesia clarkii			VU	
Malvaceae	Reevesia pubescens			CR	YES
Malvaceae	Robinsonella brevituba		EN		
Malvaceae	Robinsonella cordata				
Malvaceae	Robinsonella discolor				
Malvaceae	Robinsonella glabrifolia		CR	VU	
Malvaceae	Robinsonella lindeniana				
Malvaceae	Robinsonella lindeniana subsp. divergens				
Malvaceae	Robinsonella mirandae		EN		
Malvaceae	Robinsonella pilosissima		EN	VU	
Malvaceae	Robinsonella samaricarpa		EN		
Malvaceae	Robinsonella speciosa				
Malvaceae	Sida abutifolia				
Malvaceae	Sida acuta				
Malvaceae	Sida aggregata				

Malvaceae	Sida angustifolia		
Malvaceae	Sida barclayi		
Malvaceae	Sida brachystemon		VU
Malvaceae	Sida ciliaris		
Malvaceae	Sida cordifolia		
Malvaceae	Sida cuspidata		
Malvaceae	Sida elliottii		
Malvaceae	Sida glabra		
Malvaceae	Sida glutinosa		
Malvaceae	Sida haenkeana		
Malvaceae	Sida hirsutissima		
Malvaceae	Sida hyssopifolia		
Malvaceae	Sida jussiaeana		
Malvaceae	Sida lindheimeri		
Malvaceae	Sida linearifolia		
Malvaceae	Sida linearis		
Malvaceae	Sida linifolia		
Malvaceae	Sida rhombifolia		
Malvaceae	Sida spinosa		
Malvaceae	Sida turneroides		
Malvaceae	Sida ulmifolia		
Malvaceae	Sida urens		
Malvaceae	Sida viarum		EN
Malvaceae	Sidastrum paniculatum		
Malvaceae	Sphaeralcea angustifolia		
Malvaceae	Sphaeralcea hastulata		
Malvaceae	Sterculia apetala		
Malvaceae	Sterculia mexicana		
Malvaceae	Sterculia xolocotzii	CR	VU
Malvaceae	Tarasa geranioides		VU
Malvaceae	Theobroma bicolor		
Malvaceae	Theobroma cacao		
Malvaceae	Theobroma mammosum		EN
Malvaceae	Tilia americana		

Malvaceae	Tilia mexicana		
Malvaceae	Trichospermum galeottii		
Malvaceae	Trichospermum lessertianum		
Malvaceae	Trichospermum mexicanum		
Malvaceae	Triumfetta acahuizotlanensis	CR	CR
Malvaceae	Triumfetta acracantha		
Malvaceae	Triumfetta bogotensis		
Malvaceae	Triumfetta brevipes		
Malvaceae	Triumfetta columnaris		
Malvaceae	Triumfetta dioica		VU
Malvaceae	Triumfetta falcifera		
Malvaceae	Triumfetta galeottiana		
Malvaceae	Triumfetta goldmanii		
Malvaceae	Triumfetta grandiflora		
Malvaceae	Triumfetta heliocarpoides		
Malvaceae	Triumfetta lappula		
Malvaceae	Triumfetta paniculata		
Malvaceae	Triumfetta polyandra		
Malvaceae	Triumfetta semitriloba		
Malvaceae	Triumfetta simplicifolia		
Malvaceae	Triumfetta speciosa		
Malvaceae	Urena lobata		
Malvaceae	Waltheria acapulcensis		VU
Malvaceae	Waltheria albicans		
Malvaceae	Waltheria conzattii		
Malvaceae	Waltheria detonsa		
Malvaceae	Waltheria fryxellii		VU
Malvaceae	Waltheria glomerata		
Malvaceae	Waltheria indica		
Malvaceae	Waltheria rotundifolia		
Malvaceae	Wissadula amplissima		
Malvaceae	Wissadula excelsior		
Malvaceae	Wissadula hernandioides		
Malvaceae	Wissadula periplocifolia		

Marantaceae	Calathea crotalifera			
Marantaceae	Calathea lutea			
Marantaceae	Goeppertia allouia			
Marantaceae	Goeppertia atropurpurea			
Marantaceae	Goeppertia coccinea			
Marantaceae	Goeppertia leonoriae		CR	YES
Marantaceae	Goeppertia lietzei		EN	
Marantaceae	Goeppertia macrosepala			
Marantaceae	Goeppertia matudae		EN	YES
Marantaceae	Goeppertia micans			
Marantaceae	Goeppertia microcephala			
Marantaceae	Goeppertia misantlensis		EN	
Marantaceae	Goeppertia ornata		EN	
Marantaceae	Goeppertia ovandensis			
Marantaceae	Goeppertia soconuscum			
Marantaceae	Goeppertia sousandradeana		EN	
Marantaceae	Goeppertia violacea		EN	
Marantaceae	Goeppertia zebrina		EN	
Marantaceae	Ischnosiphon polyphyllus		EN	
Marantaceae	Maranta arundinacea			
Marantaceae	Maranta divaricata			
Marantaceae	Maranta gibba			
Marantaceae	Maranta leuconeura			
Marantaceae	Stromanthe macrochlamys			
Marantaceae	Stromanthe tonckat		VU	
Marantaceae	Thalia geniculata			
Marattiaceae	Danaea elliptica			
Marattiaceae	Danaea moritziana		VU	
Marattiaceae	Danaea nodosa			
Marattiaceae	Marattia laxa	NT		
Marattiaceae	Marattia weinmanniifolia	NT		
Marcgraviaceae	Marcgravia brownei			
Marcgraviaceae	Marcgravia mexicana			
Marcgraviaceae	Marcgravia nepenthoides		VU	

Marcgraviaceae	Marcgravia pittieri		CR
Marcgraviaceae	Marcgravia rectiflora		VU
Marcgraviaceae	Marcgravia stonei		
Marcgraviaceae	Ruyschia enervia		
Marcgraviaceae	Souroubea exauriculata		
Marcgraviaceae	Souroubea loczyi		
Martyniaceae	Martynia annua		
Martyniaceae	Proboscidea triloba		
Mayacaceae	Mayaca fluviatilis	NT	
Melanthiaceae	Anticlea frigida		
Melanthiaceae	Anticlea hintoniorum		
Melanthiaceae	Anticlea neglecta		EN
Melanthiaceae	Anticlea virescens	NT	
Melanthiaceae	Schoenocaulon ghiesbreghtii		
Melanthiaceae	Schoenocaulon madidorum		VU
Melanthiaceae	Schoenocaulon officinale		
Melanthiaceae	Schoenocaulon tenuifolium		
Melastomataceae	Aciotis indecora		
Melastomataceae	Acisanthera quadrata		
Melastomataceae	Adelobotrys adscendens		
Melastomataceae	Arthrostemma alatum		
Melastomataceae	Arthrostemma ciliatum		
Melastomataceae	Arthrostemma parvifolium		
Melastomataceae	Arthrostemma primaevum		
Melastomataceae	Bellucia grossularioides		
Melastomataceae	Bellucia pentamera		
Melastomataceae	Blakea calycularis		
Melastomataceae	Blakea laevigata		
Melastomataceae	Blakea purpusii		VU
Melastomataceae	Blakea watsonii		EN
Melastomataceae	Centradenia grandifolia		
Melastomataceae	Centradenia grandifolia subsp. grandifolia		
Melastomataceae	Centradenia inaequilateralis		
Melastomataceae	Chaetogastra longifolia		

Melastomataceae	Chaetogastra naudiniana			
Melastomataceae	Chaetogastra rufipilis			
Melastomataceae	Chaetogastra schiedeana			
Melastomataceae	Chaetogastra tortuosa			
Melastomataceae	Clidemia costaricensis		CR	
Melastomataceae	Clidemia cymifera		CR	YES
Melastomataceae	Clidemia dentata			
Melastomataceae	Clidemia donnell-smithii		VU	
Melastomataceae	Clidemia fulva			
Melastomataceae	Clidemia gracilipes		VU	
Melastomataceae	Clidemia inopinata		EN	
Melastomataceae	Clidemia laxiflora			
Melastomataceae	Clidemia matudae			
Melastomataceae	Clidemia monantha		VU	
Melastomataceae	Clidemia petiolaris			
Melastomataceae	Clidemia rubra			
Melastomataceae	Clidemia sericea			
Melastomataceae	Clidemia setosa			
Melastomataceae	Clidemia submontana			
Melastomataceae	Clidemia urceolata		EN	
Melastomataceae	Conostegia arborea			
Melastomataceae	Conostegia brenesii		CR	YES
Melastomataceae	Conostegia caelestis			
Melastomataceae	Conostegia fulvostellata			
Melastomataceae	Conostegia icosandra			
Melastomataceae	Conostegia montana		EN	
Melastomataceae	Conostegia oligocephala		VU	
Melastomataceae	Conostegia superba	VU		
Melastomataceae	Conostegia volcanalis			
Melastomataceae	Conostegia xalapensis			
Melastomataceae	Graffenrieda latifolia		CR	
Melastomataceae	Henriettea seemannii		EN	
Melastomataceae	Henriettea succosa			
Melastomataceae	Heterocentron elegans			

Melastomataceae	Heterocentron muricatum		VU	
Melastomataceae	Heterocentron subtriplinervium			
Melastomataceae	Leandra cornoides			
Melastomataceae	Leandra dichotoma		EN	
Melastomataceae	Leandra longicoma		CR	YES
Melastomataceae	Leandra melanodesma			
Melastomataceae	Leandra mexicana			
Melastomataceae	Leandra multiplinervis			
Melastomataceae	Leandra subseriata			
Melastomataceae	Meriania speciosa		CR	YES
Melastomataceae	Meriania umbellata	EN	CR	YES
Melastomataceae	Miconia aeruginosa			
Melastomataceae	Miconia affinis			
Melastomataceae	Miconia albicans			
Melastomataceae	Miconia ampla		VU	
Melastomataceae	Miconia anisotricha			
Melastomataceae	Miconia argentea			
Melastomataceae	Miconia baillonii			
Melastomataceae	Miconia barbinervis		VU	
Melastomataceae	Miconia bubalina			
Melastomataceae	Miconia calvescens			
Melastomataceae	Miconia caudata		VU	
Melastomataceae	Miconia chinantlana			
Melastomataceae	Miconia chrysoneura			
Melastomataceae	Miconia chrysophylla		VU	
Melastomataceae	Miconia ciliata			
Melastomataceae	Miconia costaricensis			
Melastomataceae	Miconia crenata			
Melastomataceae	Miconia decurrens		EN	
Melastomataceae	Miconia dependens			
Melastomataceae	Miconia desmantha			
Melastomataceae	Miconia dodecandra			
Melastomataceae	Miconia elata			
Melastomataceae	Miconia fulvostellata			

Melastomataceae	Miconia glaberrima	
Melastomataceae	Miconia globulifera	
Melastomataceae	Miconia gracilis	
Melastomataceae	Miconia guatemalensis	
Melastomataceae	Miconia hemenostigma	
Melastomataceae	Miconia holosericea	
Melastomataceae	Miconia hondurensis	VU
Melastomataceae	Miconia hyperprasina	
Melastomataceae	Miconia ibaguensis	
Melastomataceae	Miconia impetiolaris	
Melastomataceae	Miconia insularis	EN
Melastomataceae	Miconia lacera	
Melastomataceae	Miconia laevigata	
Melastomataceae	Miconia lateriflora	VU
Melastomataceae	Miconia liebmannii	
Melastomataceae	Miconia livida	
Melastomataceae	Miconia lonchophylla	
Melastomataceae	Miconia longifolia	VU
Melastomataceae	Miconia matthaei	
Melastomataceae	Miconia mexicana	
Melastomataceae	Miconia minutiflora	
Melastomataceae	Miconia moorei	
Melastomataceae	Miconia nervosa	EN
Melastomataceae	Miconia nutans	VU
Melastomataceae	Miconia octona	
Melastomataceae	Miconia oinochrophylla	
Melastomataceae	Miconia oligocephala	VU
Melastomataceae	Miconia oligotricha	
Melastomataceae	Miconia prasina	
Melastomataceae	Miconia punctata	VU
Melastomataceae	Miconia ravenii	VU
Melastomataceae	Miconia reducens	EN
Melastomataceae	Miconia rubescens	
Melastomataceae	Miconia rubra	

Melastomataceae	Miconia schlechtendalii		
Melastomataceae	Miconia septuplinervia	EN	
Melastomataceae	Miconia serrulata		
Melastomataceae	Miconia smaragdina	VU	
Melastomataceae	Miconia splendens	EN	
Melastomataceae	Miconia stenostachya		
Melastomataceae	Miconia subhirsuta		
Melastomataceae	Miconia sylvatica		
Melastomataceae	Miconia tomentosa	VU	
Melastomataceae	Miconia trinervia		
Melastomataceae	Miconia triplinervis		
Melastomataceae	Miconia tuerckheimii		
Melastomataceae	Miconia virescens	VU	
Melastomataceae	Miconia xalapensis		
Melastomataceae	Monochaetum alpestre		
Melastomataceae	Monochaetum deppeanum		
Melastomataceae	Monochaetum floribundum		
Melastomataceae	Monochaetum pulchrum		
Melastomataceae	Monochaetum rubescens	VU	
Melastomataceae	Monochaetum tenellum	EN	
Melastomataceae	Mouriri exilis	VU	
Melastomataceae	Mouriri gardneri	VU	
Melastomataceae	Mouriri gleasoniana		
Melastomataceae	Mouriri myrtilloides		
Melastomataceae	Mouriri myrtilloides subsp. parvifolia		
Melastomataceae	Pleroma galeottianum		
Melastomataceae	Pleroma heteromallum	EN	
Melastomataceae	Pleroma monticola		
Melastomataceae	Pleroma scabriusculum		
Melastomataceae	Pleroma tuberosum	CR	YES
Melastomataceae	Pleroma urvilleanum		
Melastomataceae	Pterolepis trichotoma		
Melastomataceae	Rhynchanthera mexicana		
Melastomataceae	Schwackaea cupheoides	VU	

Melastomataceae	Tibouchina breedlovei			EN
Melastomataceae	Tibouchina congestiflora			VU
Melastomataceae	Tibouchina longisepala			
Melastomataceae	Triolena scorpioides			
Meliaceae	Azadirachta indica			
Meliaceae	Cedrela oaxacensis		EN	
Meliaceae	Cedrela odorata	NT	VU	
Meliaceae	Cedrela salvadorensis			
Meliaceae	Cedrela tonduzii			
Meliaceae	Guarea bijuga			
Meliaceae	Guarea bullata			VU
Meliaceae	Guarea glabra			
Meliaceae	Guarea glabra subsp. excelsa			
Meliaceae	Guarea glabra subsp. glabrescens			
Meliaceae	Guarea glabra subsp. rovirosae			
Meliaceae	Guarea glabra subsp. tuerckheimii			
Meliaceae	Guarea guidonia			
Meliaceae	Guarea macrophylla			
Meliaceae	Guarea macrophylla subsp. pachycarpa			CR
Meliaceae	Guarea megantha			
Meliaceae	Guarea mexicana		EN	EN
Meliaceae	Guarea tonduzii			
Meliaceae	Melia azedarach			
Meliaceae	Swietenia humilis		EN	
Meliaceae	Swietenia macrophylla		VU	
Meliaceae	Trichilia americana			
Meliaceae	Trichilia breviflora		EN	
Meliaceae	Trichilia chirriactensis		CR	EN
Meliaceae	Trichilia erythrocarpa		EN	
Meliaceae	Trichilia glabra			
Meliaceae	Trichilia havanensis			
Meliaceae	Trichilia hirta			
Meliaceae	Trichilia martiana			
Meliaceae	Trichilia minutiflora		VU	

Meliaceae	Trichilia moschata		
Meliaceae	Trichilia moschata subsp. matudae		EN
Meliaceae	Trichilia pallida		
Meliaceae	Trichilia pleeana		
Meliaceae	Trichilia quadrijuga		EN
Meliaceae	Trichilia quadrijuga subsp. cinerascens		CR
Meliaceae	Trichilia tomentosa		VU
Meliaceae	Trichilia trifolia		
Meliaceae	Trichilia trifolia subsp. palmeri		
Menispermaceae	Abuta chiapasensis		
Menispermaceae	Abuta panamensis		
Menispermaceae	Abuta steyermarkii		VU
Menispermaceae	Cissampelos fasciculata		
Menispermaceae	Cissampelos grandifolia		
Menispermaceae	Cissampelos pareira		
Menispermaceae	Cissampelos tropaeolifolia		
Menispermaceae	Cocculus diversifolius		
Menispermaceae	Disciphania calocarpa		
Menispermaceae	Hyperbaena ilicifolia		
Menispermaceae	Hyperbaena mexicana		
Menispermaceae	Hyperbaena standleyi		EN
Menispermaceae	Menispermum canadense		VU
Menispermaceae	Odontocarya mexicana		VU
Menyanthaceae	Liparophyllum exaltatum		VU
Menyanthaceae	Nymphoides fallax		
Menyanthaceae	Nymphoides indica		
Metteniusaceae	Calatola costaricensis		
Metteniusaceae	Calatola laevigata	EN	
Metteniusaceae	Calatola mollis	CR	
Metteniusaceae	Calatola uxpanapensis		VU
Metteniusaceae	Oecopetalum greenmanii		
Metteniusaceae	Oecopetalum mexicanum		
Molluginaceae	Glinus radiatus		
Molluginaceae	Mollugo verticillata		

Monimiaceae	Mollinedia butleriana	
Monimiaceae	Mollinedia pallida	
Monimiaceae	Mollinedia viridiflora	
Montiaceae	Calandrinia ciliata	
Montiaceae	Claytonia perfoliata	
Montiaceae	Claytonia perfoliata subsp. mexicana	
Montiaceae	Montia fontana	VU
Moraceae	Artocarpus altilis	
Moraceae	Artocarpus heterophyllus	
Moraceae	Brosimum alicastrum	
Moraceae	Brosimum costaricanum	VU
Moraceae	Brosimum guianense	
Moraceae	Brosimum lactescens	
Moraceae	Castilla elastica	
Moraceae	Clarisia biflora	
Moraceae	Clarisia mexicana	
Moraceae	Clarisia racemosa	EN
Moraceae	Dorstenia contrajerva	
Moraceae	Dorstenia drakena	
Moraceae	Dorstenia lindeniana	
Moraceae	Dorstenia uxpanapana	VU
Moraceae	Ficus adhatodifolia	VU
Moraceae	Ficus americana	
Moraceae	Ficus apollinaris	
Moraceae	Ficus aurea	
Moraceae	Ficus benjamina	
Moraceae	Ficus cahuitensis	EN
Moraceae	Ficus calyculata	
Moraceae	Ficus carica	
Moraceae	Ficus citrifolia	
Moraceae	Ficus colubrinae	
Moraceae	Ficus costaricana	
Moraceae	Ficus cotinifolia	
Moraceae	Ficus crassinervia	

Moraceae	Ficus crocata			
Moraceae	Ficus goldmanii			
Moraceae	Ficus hispida		EN	
Moraceae	Ficus insipida			
Moraceae	Ficus lapathifolia	VU		
Moraceae	Ficus macrosyce		EN	YES
Moraceae	Ficus maxima			
Moraceae	Ficus mexicana			
Moraceae	Ficus microcarpa			
Moraceae	Ficus obtusifolia			
Moraceae	Ficus paraensis		VU	
Moraceae	Ficus pertusa			
Moraceae	Ficus petiolaris			
Moraceae	Ficus pringlei			
Moraceae	Ficus pumila			
Moraceae	Ficus retusa			
Moraceae	Ficus rzedowskiana			
Moraceae	Ficus segoviae			
Moraceae	Ficus trigonata			
Moraceae	Ficus turrialbana			
Moraceae	Ficus velutina			
Moraceae	Ficus yoponensis			
Moraceae	Maclura tinctoria			
Moraceae	Morus alba			
Moraceae	Morus celtidifolia			
Moraceae	Poulsenia armata			
Moraceae	Pseudolmedia glabrata			
Moraceae	Pseudolmedia spuria			
Moraceae	Sorocea affinis		EN	
Moraceae	Sorocea trophoides		EN	
Moraceae	Trophis cuspidata			
Moraceae	Trophis mexicana			
Moraceae	Trophis racemosa			
Muntingiaceae	Muntingia calabura			

Musaceae	Musa × paradisiaca		
Musaceae	Musa acuminata		
Musaceae	Musa coccinea	EN	EN
Musaceae	Musa ornata		
Myricaceae	Myrica cerifera		
Myricaceae	Myrica lindeniana		
Myricaceae	Myrica pringlei		
Myristicaceae	Compsoneura mexicana		
Myristicaceae	Compsoneura sprucei		
Myristicaceae	Virola guatemalensis		
Myrtaceae	Calycorectes mexicanus	EN	
Myrtaceae	Calyptranthes chytraculia		
Myrtaceae	Calyptranthes chytraculia var. americana		
Myrtaceae	Calyptranthes fluviatilis		
Myrtaceae	Calyptranthes williamsii		
Myrtaceae	Chamguava schippii		VU
Myrtaceae	Corymbia ficifolia		EN
Myrtaceae	Eucalyptus camaldulensis		
Myrtaceae	Eucalyptus cinerea		
Myrtaceae	Eucalyptus globulus		
Myrtaceae	Eucalyptus tereticornis		VU
Myrtaceae	Eugenia acapulcensis		
Myrtaceae	Eugenia aeruginea		
Myrtaceae	Eugenia alnifolia	EN	EN
Myrtaceae	Eugenia amatenangensis	VU	
Myrtaceae	Eugenia avicenniae		
Myrtaceae	Eugenia axillaris		
Myrtaceae	Eugenia biflora		
Myrtaceae	Eugenia breedlovei	EN	
Myrtaceae	Eugenia bumelioides		EN
Myrtaceae	Eugenia buxifolia		
Myrtaceae	Eugenia capuli		
Myrtaceae	Eugenia capulioides		
Myrtaceae	Eugenia chiapensis		VU

Myrtaceae	Eugenia choapamensis		
Myrtaceae	Eugenia citroides		VU
Myrtaceae	Eugenia coetzalensis		EN
Myrtaceae	Eugenia crenularis		
Myrtaceae	Eugenia farameoides		
Myrtaceae	Eugenia galalonensis		
Myrtaceae	Eugenia gaumeri		
Myrtaceae	Eugenia guatemalensis		
Myrtaceae	Eugenia hypargyrea		
Myrtaceae	Eugenia inirebensis	VU	VU
Myrtaceae	Eugenia karwinskyana		
Myrtaceae	Eugenia koepperi		VU
Myrtaceae	Eugenia letreroana		EN
Myrtaceae	Eugenia liebmannii		
Myrtaceae	Eugenia macrocarpa		
Myrtaceae	Eugenia nigrita	VU	
Myrtaceae	Eugenia octopleura		EN
Myrtaceae	Eugenia oerstediana		
Myrtaceae	Eugenia percivalii		EN
Myrtaceae	Eugenia pleurocarpa		
Myrtaceae	Eugenia pueblana	EN	VU
Myrtaceae	Eugenia ravenii		VU
Myrtaceae	Eugenia rhombea		
Myrtaceae	Eugenia riograndis		EN
Myrtaceae	Eugenia rubella		EN
Myrtaceae	Eugenia salamensis	EN	
Myrtaceae	Eugenia savannarum		VU
Myrtaceae	Eugenia siltepecana	VU	
Myrtaceae	Eugenia standleyi	EN	
Myrtaceae	Eugenia teapensis	EN	VU
Myrtaceae	Eugenia tikalana		
Myrtaceae	Eugenia trikii		
Myrtaceae	Eugenia trunciflora		
Myrtaceae	Eugenia uliginosa		

Myrtaceae	Eugenia uniflora			
Myrtaceae	Eugenia uxpanapensis	EN	EN	
Myrtaceae	Eugenia venezuelensis			
Myrtaceae	Eugenia vesca		EN	
Myrtaceae	Eugenia winzerlingii			
Myrtaceae	Eugenia xalapensis			
Myrtaceae	Eugenia xilitlensis		EN	
Myrtaceae	Eugenia yunckeri			
Myrtaceae	Melaleuca citrina			
Myrtaceae	Melaleuca huegelii		CR	
Myrtaceae	Mosiera ehrenbergii			
Myrtaceae	Myrcia amazonica		EN	
Myrtaceae	Myrcia chiapensis			
Myrtaceae	Myrcia chytraculia			
Myrtaceae	Myrcia karlingii			
Myrtaceae	Myrcia macrantha		EN	YES
Myrtaceae	Myrcia millspaughii			
Myrtaceae	Myrcia neohernandezii		CR	YES
Myrtaceae	Myrcia neovenulosa	EN	EN	
Myrtaceae	Myrcia paxillata			
Myrtaceae	Myrcia pendens			
Myrtaceae	Myrcia perlaevigata		VU	
Myrtaceae	Myrcia quitarensis		CR	YES
Myrtaceae	Myrcia salamensis	CR	CR	YES
Myrtaceae	Myrcia schlechtendaliana			
Myrtaceae	Myrcia splendens			
Myrtaceae	Myrcia tenuipes			
Myrtaceae	Myrcia tonii		VU	
Myrtaceae	Myrcianthes fragrans			
Myrtaceae	Myrciaria floribunda			
Myrtaceae	Pimenta dioica			
Myrtaceae	Psidium friedrichsthalianum			
Myrtaceae	Psidium guajava			
Myrtaceae	Psidium guineense			

Myrtaceae	Psidium oligospermum		
Myrtaceae	Psidium salutare		
Myrtaceae	Psidium salutare var. salutare		
Myrtaceae	Syzygium jambos		
Myrtaceae	Syzygium malaccense	VU	
Myrtaceae	Ugni myricoides		
Nyctaginaceae	Allionia choisyi		
Nyctaginaceae	Boerhavia anisophylla		
Nyctaginaceae	Boerhavia coccinea		
Nyctaginaceae	Boerhavia diffusa		
Nyctaginaceae	Boerhavia erecta		
Nyctaginaceae	Bougainvillea × buttiana		
Nyctaginaceae	Bougainvillea glabra		
Nyctaginaceae	Bougainvillea spectabilis		
Nyctaginaceae	Commicarpus scandens		
Nyctaginaceae	Guapira costaricana		
Nyctaginaceae	Guapira petenensis		
Nyctaginaceae	Guapira potosina	EN	
Nyctaginaceae	Mirabilis albida		
Nyctaginaceae	Mirabilis glabrifolia		
Nyctaginaceae	Mirabilis jalapa		
Nyctaginaceae	Mirabilis longiflora		
Nyctaginaceae	Mirabilis nyctaginea		
Nyctaginaceae	Mirabilis violacea		
Nyctaginaceae	Mirabilis viscosa		
Nyctaginaceae	Neea amplifolia	VU	
Nyctaginaceae	Neea chiapensis	CR	YES
Nyctaginaceae	Neea psychotrioides		
Nyctaginaceae	Neea spruceana	CR	
Nyctaginaceae	Neea stenophylla		
Nyctaginaceae	Neea tenuis		
Nyctaginaceae	Neea turbinata	EN	
Nyctaginaceae	Okenia hypogaea		
Nyctaginaceae	Pisonia aculeata		

Nyctaginaceae	Pisonia donnellsmithii		VU	VU
Nyctaginaceae	Pisonia fasciculata			
Nyctaginaceae	Pisonia macranthocarpa			
Nyctaginaceae	Salpianthus arenarius			
Nyctaginaceae	Salpianthus macrodontus			
Nyctaginaceae	Salpianthus purpurascens			
Nymphaeaceae	Nuphar advena			
Nymphaeaceae	Nuphar lutea	VU		VU
Nymphaeaceae	Nymphaea ampla			
Nymphaeaceae	Nymphaea elegans			
Nymphaeaceae	Nymphaea jamesoniana			
Nymphaeaceae	Nymphaea pulchella			
Nyssaceae	Nyssa sylvatica			
Ochnaceae	Ouratea crassinervia			VU
Ochnaceae	Ouratea insulae		VU	
Ochnaceae	Ouratea jurgensenii		VU	VU
Ochnaceae	Ouratea lucens			
Ochnaceae	Ouratea madrensis		EN	EN
Ochnaceae	Ouratea mexicana			
Ochnaceae	Ouratea nitida			
Ochnaceae	Ouratea theophrasta		CR	
Ochnaceae	Sauvagesia erecta			
Olacaceae	Heisteria acuminata			VU
Olacaceae	Heisteria media			
Olacaceae	Ximenia americana			
Olacaceae	Ximenia parviflora			
Oleaceae	Cartrema americana			
Oleaceae	Chionanthus domingensis			
Oleaceae	Chionanthus ligustrinus			VU
Oleaceae	Chionanthus oblanceolatus			
Oleaceae	Chionanthus panamensis			VU
Oleaceae	Chrysojasminum humile			EN
Oleaceae	Forestiera angustifolia			
Oleaceae	Forestiera phillyreoides			

Oleaceae	Forestiera racemosa		
Oleaceae	Forestiera reticulata		
Oleaceae	Forestiera rhamnifolia		
Oleaceae	Forestiera rotundifolia		
Oleaceae	Fraxinus albicans		
Oleaceae	Fraxinus berlandieriana		
Oleaceae	Fraxinus dubia		
Oleaceae	Fraxinus pringlei		
Oleaceae	Fraxinus purpusii		
Oleaceae	Fraxinus purpusii var. purpusii	CR	YES
Oleaceae	Fraxinus purpusii var. vellerea	VU	
Oleaceae	Fraxinus uhdei		
Oleaceae	Jasminum grandiflorum		
Oleaceae	Jasminum multiflorum		
Oleaceae	Jasminum sambac		
Oleaceae	Jasminum simplicifolium subsp. australiense	EN	
Oleaceae	Ligustrum lucidum		
Oleaceae	Ligustrum vulgare		
Oleaceae	Menodora helianthemoides		
Onagraceae	Epilobium ciliatum		
Onagraceae	Fuchsia × hybrida		
Onagraceae	Fuchsia arborescens		
Onagraceae	Fuchsia boliviana	VU	
Onagraceae	Fuchsia encliandra		
Onagraceae	Fuchsia encliandra subsp. tetradactyla		
Onagraceae	Fuchsia magellanica		
Onagraceae	Fuchsia microphylla		
Onagraceae	Fuchsia microphylla subsp. aprica		
Onagraceae	Fuchsia microphylla subsp. quercetorum		
Onagraceae	Fuchsia paniculata		
Onagraceae	Fuchsia paniculata subsp. mixensis	EN	
Onagraceae	Fuchsia parviflora		
Onagraceae	Fuchsia splendens		
Onagraceae	Fuchsia thymifolia		

Onagraceae	Fuchsia thymifolia subsp. minimiflora	
Onagraceae	Gongylocarpus rubricaulis	
Onagraceae	Hauya elegans	
Onagraceae	Hauya elegans subsp. barcenae	
Onagraceae	Hauya elegans subsp. cornuta	
Onagraceae	Lopezia grandiflora subsp. macrophylla	EN
Onagraceae	Lopezia hirsuta	
Onagraceae	Lopezia langmaniae	
Onagraceae	Lopezia miniata	
Onagraceae	Lopezia racemosa	
Onagraceae	Lopezia racemosa subsp. moelchenensis	VU
Onagraceae	Ludwigia decurrens	
Onagraceae	Ludwigia erecta	
Onagraceae	Ludwigia foliobracteolata	EN
Onagraceae	Ludwigia inclinata	
Onagraceae	Ludwigia leptocarpa	
Onagraceae	Ludwigia nervosa	
Onagraceae	Ludwigia octovalvis	
Onagraceae	Ludwigia palustris	
Onagraceae	Ludwigia peploides	
Onagraceae	Ludwigia peruviana	
Onagraceae	Ludwigia repens	
Onagraceae	Ludwigia suffruticosa	
Onagraceae	Ludwigia torulosa	VU
Onagraceae	Oenothera anomala	
Onagraceae	Oenothera biennis	
Onagraceae	Oenothera deserticola	
Onagraceae	Oenothera elata subsp. hookeri	VU
Onagraceae	Oenothera epilobiifolia	
Onagraceae	Oenothera epilobiifolia subsp. cuprea	
Onagraceae	Oenothera hexandra	
Onagraceae	Oenothera kunthiana	
Onagraceae	Oenothera laciniata	
Onagraceae	Oenothera multicaulis	VU

Onagraceae	Oenothera pubescens			
Onagraceae	Oenothera rosea			
Onagraceae	Oenothera speciosa			
Onagraceae	Oenothera suffrutescens			
Onagraceae	Oenothera tetraptera			
Onagraceae	Oenothera versicolor			
Ophioglossaceae	Botrychium decompositum			
Ophioglossaceae	Botrychium jenmanii		CR	YES
Ophioglossaceae	Botrychium schaffneri			
Ophioglossaceae	Botrychium virginianum			
Ophioglossaceae	Botrychium virginianum subsp. virginianum		EN	
Ophioglossaceae	Ophioglossum engelmannii			
Ophioglossaceae	Ophioglossum palmatum		EN	
Ophioglossaceae	Ophioglossum reticulatum			
Opiliaceae	Agonandra obtusifolia			
Opiliaceae	Agonandra racemosa			
Orchidaceae	Acianthera angustifolia			
Orchidaceae	Acianthera angustisepala		EN	
Orchidaceae	Acianthera breedlovei		VU	
Orchidaceae	Acianthera circumplexa			
Orchidaceae	Acianthera erinacea		EN	
Orchidaceae	Acianthera hondurensis		EN	
Orchidaceae	Acianthera obscura		EN	
Orchidaceae	Acianthera pubescens		VU	
Orchidaceae	Acianthera sotoana		EN	
Orchidaceae	Acianthera tikalensis		VU	
Orchidaceae	Acineta barkeri	VU		
Orchidaceae	Acineta densa		EN	
Orchidaceae	Alamania punicea			
Orchidaceae	Anathallis abbreviata		VU	
Orchidaceae	Anathallis barbulata		VU	
Orchidaceae	Anathallis inversa		CR	YES
Orchidaceae	Anathallis lewisiae		VU	
Orchidaceae	Anathallis minutalis			

Orchidaceae	Anathallis sertularioides			
Orchidaceae	Arpophyllum giganteum			
Orchidaceae	Arpophyllum giganteum subsp. alpinum			
Orchidaceae	Arpophyllum giganteum subsp. giganteum		VU	
Orchidaceae	Arpophyllum giganteum subsp. medium			
Orchidaceae	Arpophyllum laxiflorum			
Orchidaceae	Arpophyllum spicatum			
Orchidaceae	Artorima erubescens			
Orchidaceae	Aspidogyne clavigera		VU	
Orchidaceae	Aspidogyne querceticola			
Orchidaceae	Aspidogyne stictophylla	NT	VU	
Orchidaceae	Aspidogyne tuerckheimii		CR	YES
Orchidaceae	Aspidogyne venustula		CR	YES
Orchidaceae	Aulosepalum hemichrea			
Orchidaceae	Aulosepalum nelsonii			
Orchidaceae	Aulosepalum pulchrum		VU	
Orchidaceae	Aulosepalum pyramidale			
Orchidaceae	Aulosepalum ramentaceum		VU	
Orchidaceae	Barkeria lindleyana			
Orchidaceae	Barkeria lindleyana subsp. vanneriana			
Orchidaceae	Barkeria melanocaulon	VU	VU	
Orchidaceae	Barkeria naevosa			
Orchidaceae	Barkeria obovata			
Orchidaceae	Barkeria scandens	NT		
Orchidaceae	Barkeria skinneri	NT		
Orchidaceae	Barkeria spectabilis			
Orchidaceae	Barkeria uniflora			
Orchidaceae	Beloglottis costaricensis			
Orchidaceae	Beloglottis mexicana			
Orchidaceae	Bletia adenocarpa			
Orchidaceae	Bletia campanulata			
Orchidaceae	Bletia greenmaniana		VU	
Orchidaceae	Bletia lilacina			
Orchidaceae	Bletia neglecta			

Orchidaceae	Bletia nelsonii		VU	
Orchidaceae	Bletia parkinsonii			
Orchidaceae	Bletia purpurata			
Orchidaceae	Bletia purpurea			
Orchidaceae	Bletia reflexa			
Orchidaceae	Bletia roezlii			
Orchidaceae	Bletia tenuifolia			
Orchidaceae	Brachystele guayanensis		EN	
Orchidaceae	Brassavola cucullata			
Orchidaceae	Brassavola nodosa			
Orchidaceae	Brassia caudata			
Orchidaceae	Brassia maculata			
Orchidaceae	Brassia verrucosa			
Orchidaceae	Bulbophyllum aristatum			
Orchidaceae	Bulbophyllum bracteolatum		CR	YES
Orchidaceae	Bulbophyllum oerstedii			
Orchidaceae	Bulbophyllum pinelianum		VU	
Orchidaceae	Bulbophyllum sordidum		VU	
Orchidaceae	Calanthe calanthoides			
Orchidaceae	Calopogon tuberosus		EN	
Orchidaceae	Campylocentrum hondurense		EN	
Orchidaceae	Campylocentrum micranthum			
Orchidaceae	Campylocentrum schiedei			
Orchidaceae	Catasetum integerrimum			
Orchidaceae	Catasetum laminatum			
Orchidaceae	Catasetum lemosii		EN	
Orchidaceae	Catasetum maculatum		EN	
Orchidaceae	Chysis aurea		EN	
Orchidaceae	Chysis bractescens	VU		
Orchidaceae	Chysis laevis			
Orchidaceae	Chysis tricostata		CR	YES
Orchidaceae	Clowesia dodsoniana			
Orchidaceae	Clowesia rosea	VU		
Orchidaceae	Clowesia russelliana			

Orchidaceae	Cochleanthes flabelliformis	NT	CR	YES
Orchidaceae	Coelia bella			
Orchidaceae	Coelia densiflora	NT	EN	
Orchidaceae	Coelia guatemalensis			
Orchidaceae	Coelia macrostachya			
Orchidaceae	Coelia triptera			
Orchidaceae	Coenoemersa limosa			
Orchidaceae	Comparettia falcata			
Orchidaceae	Comparettia tuerckheimii	VU		
Orchidaceae	Corallorhiza bulbosa			
Orchidaceae	Corallorhiza maculata			
Orchidaceae	Corallorhiza maculata var. mexicana			
Orchidaceae	Corallorhiza striata var. vreelandii			
Orchidaceae	Coryanthes picturata			
Orchidaceae	Corymborkis forcipigera			
Orchidaceae	Cranichis apiculata			
Orchidaceae	Cranichis candida		CR	YES
Orchidaceae	Cranichis ciliilabia		VU	
Orchidaceae	Cranichis cochleata			
Orchidaceae	Cranichis muscosa		VU	
Orchidaceae	Cranichis subumbellata			
Orchidaceae	Cranichis sylvatica			
Orchidaceae	Cranichis tenuis		CR	YES
Orchidaceae	Cranichis wageneri		VU	
Orchidaceae	Cryptarrhena lunata	NT	VU	
Orchidaceae	Cuitlauzina convallarioides		EN	
Orchidaceae	Cuitlauzina egertonii			
Orchidaceae	Cuitlauzina pulchella			
Orchidaceae	Cyclopogon comosus			
Orchidaceae	Cyclopogon elatus			
Orchidaceae	Cyclopogon luteoalbus			
Orchidaceae	Cyclopogon prasophyllus		VU	
Orchidaceae	Cyclopogon saccatus			
Orchidaceae	Cycnoches egertonianum			

Orchidaceae	Cycnoches ventricosum	VU		
Orchidaceae	Cypripedium irapeanum	VU	VU	
Orchidaceae	Cypripedium molle			
Orchidaceae	Cyrtochiloides ochmatochila	VU		VU
Orchidaceae	Cyrtopodium macrobulbon			
Orchidaceae	Cyrtopodium paniculatum			
Orchidaceae	Cyrtopodium punctatum			
Orchidaceae	Deiregyne diaphana			
Orchidaceae	Deiregyne eriophora			
Orchidaceae	Deiregyne pallens			EN
Orchidaceae	Deiregyne pseudopyramidalis			
Orchidaceae	Deiregyne rhombilabia			
Orchidaceae	Deiregyne tenorioi			
Orchidaceae	Dichaea glauca			
Orchidaceae	Dichaea graminoides			
Orchidaceae	Dichaea intermedia			VU
Orchidaceae	Dichaea morrisii			
Orchidaceae	Dichaea muricatoides			
Orchidaceae	Dichaea neglecta			
Orchidaceae	Dichaea panamensis			
Orchidaceae	Dichaea squarrosa			
Orchidaceae	Dichaea trichocarpa			
Orchidaceae	Dichromanthus aurantiacus			
Orchidaceae	Dichromanthus cinnabarinus			
Orchidaceae	Dichromanthus michuacanus			
Orchidaceae	Dimerandra emarginata			
Orchidaceae	Dinema polybulbon			
Orchidaceae	Domingoa purpurea			
Orchidaceae	Dracula pusilla	NT		EN
Orchidaceae	Dryadella guatemalensis	NT		VU
Orchidaceae	Dryadella linearifolia			
Orchidaceae	Dryadella simula			EN
Orchidaceae	Elleanthus capitatus			
Orchidaceae	Elleanthus caricoides			

Orchidaceae	Elleanthus graminifolius			
Orchidaceae	Elleanthus hymenophorus	VU	EN	
Orchidaceae	Encyclia aenicta			
Orchidaceae	Encyclia alata			
Orchidaceae	Encyclia alata subsp. virella			
Orchidaceae	Encyclia ambigua			
Orchidaceae	Encyclia asperula		VU	
Orchidaceae	Encyclia bractescens			
Orchidaceae	Encyclia calderoniae		VU	
Orchidaceae	Encyclia candollei			
Orchidaceae	Encyclia ceratistes		VU	
Orchidaceae	Encyclia chiapasensis		EN	
Orchidaceae	Encyclia chloroleuca		EN	
Orchidaceae	Encyclia cordigera			
Orchidaceae	Encyclia diota			
Orchidaceae	Encyclia dressleri		CR	YES
Orchidaceae	Encyclia gravida		VU	
Orchidaceae	Encyclia hanburyi			
Orchidaceae	Encyclia incumbens			
Orchidaceae	Encyclia parviflora			
Orchidaceae	Encyclia peraltensis		CR	YES
Orchidaceae	Encyclia selligera			
Orchidaceae	Encyclia tuerckheimii	NT	VU	
Orchidaceae	Epidendrum acunae		EN	
Orchidaceae	Epidendrum alabastrialatum	NT		
Orchidaceae	Epidendrum albopropinquum		EN	
Orchidaceae	Epidendrum alticola	VU	EN	
Orchidaceae	Epidendrum alvarezdeltoroi		EN	
Orchidaceae	Epidendrum anceps			
Orchidaceae	Epidendrum angustilobum		CR	YES
Orchidaceae	Epidendrum anisatum			
Orchidaceae	Epidendrum arbuscula			
Orchidaceae	Epidendrum atroscriptum			
Orchidaceae	Epidendrum carchiense		CR	YES

Orchidaceae	Epidendrum cardiochilum			
Orchidaceae	Epidendrum cardiophorum			
Orchidaceae	Epidendrum caroli		VU	
Orchidaceae	Epidendrum carpophorum		CR	YES
Orchidaceae	Epidendrum centropetalum		VU	
Orchidaceae	Epidendrum cerinum	NT	VU	
Orchidaceae	Epidendrum chloe	NT	EN	
Orchidaceae	Epidendrum chlorocorymbos			
Orchidaceae	Epidendrum ciliare			
Orchidaceae	Epidendrum cilioccidentale		EN	
Orchidaceae	Epidendrum citrosmum			
Orchidaceae	Epidendrum cnemidophorum	VU		
Orchidaceae	Epidendrum conopseum			
Orchidaceae	Epidendrum coriifolium			
Orchidaceae	Epidendrum coronatum	NT	EN	
Orchidaceae	Epidendrum criniferum		VU	
Orchidaceae	Epidendrum cristatum			
Orchidaceae	Epidendrum culmiforme		EN	
Orchidaceae	Epidendrum cystosum	NT	EN	
Orchidaceae	Epidendrum difforme			
Orchidaceae	Epidendrum diffusum			
Orchidaceae	Epidendrum erectifolium			
Orchidaceae	Epidendrum eustirum			
Orchidaceae	Epidendrum eximium			
Orchidaceae	Epidendrum falcatum			
Orchidaceae	Epidendrum flexuosum			
Orchidaceae	Epidendrum fruticosum		EN	
Orchidaceae	Epidendrum galeottianum			
Orchidaceae	Epidendrum ibaguense			
Orchidaceae	Epidendrum incomptoides	NT	VU	
Orchidaceae	Epidendrum incomptum		EN	
Orchidaceae	Epidendrum isomerum			
Orchidaceae	Epidendrum isthmi	NT	EN	
Orchidaceae	Epidendrum laucheanum			

Orchidaceae	Epidendrum longipetalum			
Orchidaceae	Epidendrum macdougallii			
Orchidaceae	Epidendrum macroclinium			
Orchidaceae	Epidendrum magnificum		VU	
Orchidaceae	Epidendrum martinezii			
Orchidaceae	Epidendrum melistagum			
Orchidaceae	Epidendrum mesocarpum		EN	
Orchidaceae	Epidendrum microcharis		VU	
Orchidaceae	Epidendrum mixtum			
Orchidaceae	Epidendrum mocinoi		EN	
Orchidaceae	Epidendrum myodes		VU	
Orchidaceae	Epidendrum myrianthum			
Orchidaceae	Epidendrum nitens			
Orchidaceae	Epidendrum nocturnum			
Orchidaceae	Epidendrum nubium		EN	
Orchidaceae	Epidendrum pachyceras		CR	YES
Orchidaceae	Epidendrum pachyrachis		EN	
Orchidaceae	Epidendrum paniculatum			
Orchidaceae	Epidendrum pansamalae	VU	EN	
Orchidaceae	Epidendrum paranthicum		EN	
Orchidaceae	Epidendrum parkinsonianum			
Orchidaceae	Epidendrum peperomia		EN	
Orchidaceae	Epidendrum physodes		EN	
Orchidaceae	Epidendrum polyanthum			
Orchidaceae	Epidendrum porpax		VU	
Orchidaceae	Epidendrum propinquum			
Orchidaceae	Epidendrum pseudoramosum			
Orchidaceae	Epidendrum radicans			
Orchidaceae	Epidendrum radioferens			
Orchidaceae	Epidendrum ramosum			
Orchidaceae	Epidendrum repens			
Orchidaceae	Epidendrum rigidum			
Orchidaceae	Epidendrum roseoscriptum		EN	
Orchidaceae	Epidendrum santaclarense		EN	

Orchidaceae	Epidendrum schlechterianum		VU	
Orchidaceae	Epidendrum sculptum		EN	
Orchidaceae	Epidendrum singuliflorum		VU	
Orchidaceae	Epidendrum skutchii	NT	EN	
Orchidaceae	Epidendrum sobralioides	VU	EN	
Orchidaceae	Epidendrum stamfordianum			
Orchidaceae	Epidendrum strobiliferum			
Orchidaceae	Epidendrum trachythece		VU	
Orchidaceae	Epidendrum tziscaoense		EN	
Orchidaceae	Epidendrum vandifolium			
Orchidaceae	Epidendrum veroscriptum			
Orchidaceae	Epidendrum verrucosum			
Orchidaceae	Eriopsis biloba		EN	YES
Orchidaceae	Erycina crista-galli	NT		
Orchidaceae	Erycina echinata			
Orchidaceae	Erycina pumilio	NT	EN	YES
Orchidaceae	Erycina pusilla			
Orchidaceae	Eulophia alta			
Orchidaceae	Eurystyles ananassocomos		EN	
Orchidaceae	Fernandezia mexicana	NT	EN	
Orchidaceae	Funkiella hyemalis			
Orchidaceae	Funkiella parasitica			
Orchidaceae	Funkiella valerioi		EN	
Orchidaceae	Galeoglossum tubulosum			
Orchidaceae	Galeottiella sarcoglossa	NT		
Orchidaceae	Gongora galeata			
Orchidaceae	Gongora galeottiana		VU	
Orchidaceae	Gongora leucochila			
Orchidaceae	Gongora quinquenervis			
Orchidaceae	Gongora truncata			
Orchidaceae	Gongora unicolor			
Orchidaceae	Goodyera major		EN	
Orchidaceae	Goodyera striata			
Orchidaceae	Govenia alba			

Orchidaceae	Govenia bella		EN	
Orchidaceae	Govenia dressleriana			
Orchidaceae	Govenia lagenophora			
Orchidaceae	Govenia liliacea			
Orchidaceae	Govenia matudae		VU	
Orchidaceae	Govenia praecox		VU	
Orchidaceae	Govenia purpusii			
Orchidaceae	Govenia superba			
Orchidaceae	Govenia utriculata			
Orchidaceae	Greenwoodiella micrantha			
Orchidaceae	Greenwoodiella micrantha var. garayana		VU	
Orchidaceae	Guarianthe aurantiaca			
Orchidaceae	Guarianthe bowringiana		CR	YES
Orchidaceae	Guarianthe skinneri	VU		
Orchidaceae	Habenaria alata			
Orchidaceae	Habenaria bractescens			
Orchidaceae	Habenaria brevilabiata		EN	
Orchidaceae	Habenaria clypeata			
Orchidaceae	Habenaria crassicornis			
Orchidaceae	Habenaria distans			
Orchidaceae	Habenaria entomantha			
Orchidaceae	Habenaria eustachya		VU	
Orchidaceae	Habenaria floribunda			
Orchidaceae	Habenaria guadalajarana			
Orchidaceae	Habenaria ixtlanensis		EN	
Orchidaceae	Habenaria jaliscana			
Orchidaceae	Habenaria lactiflora			
Orchidaceae	Habenaria lucaecapensis			
Orchidaceae	Habenaria macroceratitis			
Orchidaceae	Habenaria monorrhiza			
Orchidaceae	Habenaria novemfida	NT		
Orchidaceae	Habenaria pringlei			
Orchidaceae	Habenaria quinqueseta			
Orchidaceae	Habenaria repens			

Orchidaceae	Habenaria spithamaea		CR	YES
Orchidaceae	Habenaria strictissima			
Orchidaceae	Habenaria tetranema		VU	
Orchidaceae	Habenaria trifida			
Orchidaceae	Habenaria tuerckheimii		VU	
Orchidaceae	Habenaria virens			
Orchidaceae	Habenaria wercklei		EN	
Orchidaceae	Hexalectris grandiflora			
Orchidaceae	Homalopetalum pumilio			
Orchidaceae	Homalopetalum pumilum			
Orchidaceae	Ionopsis satyrioides	NT	EN	
Orchidaceae	Ionopsis utricularioides			
Orchidaceae	Isochilus alatus			
Orchidaceae	Isochilus aurantiacus			
Orchidaceae	Isochilus carnosiflorus			
Orchidaceae	Isochilus chiriquensis			
Orchidaceae	Isochilus latibracteatus			
Orchidaceae	Isochilus linearis			
Orchidaceae	Isochilus major			
Orchidaceae	Isochilus unilateralis			
Orchidaceae	Jacquiniella cobanensis			
Orchidaceae	Jacquiniella equitantifolia			
Orchidaceae	Jacquiniella gigantea	NT	EN	
Orchidaceae	Jacquiniella globosa			
Orchidaceae	Jacquiniella leucomelana			
Orchidaceae	Jacquiniella teretifolia			
Orchidaceae	Kionophyton sawyeri			
Orchidaceae	Kionophyton seminuda			
Orchidaceae	Lacaena bicolor	VU	EN	
Orchidaceae	Laelia albida			
Orchidaceae	Laelia anceps			
Orchidaceae	Laelia anceps subsp. anceps		EN	
Orchidaceae	Laelia autumnalis	NT		
Orchidaceae	Laelia furfuracea	NT		

Orchidaceae	Laelia rubescens			
Orchidaceae	Laelia speciosa	NT		
Orchidaceae	Laelia superbiens	VU		
Orchidaceae	Leochilus carinatus			
Orchidaceae	Leochilus labiatus		VU	
Orchidaceae	Leochilus oncidioides			
Orchidaceae	Leochilus scriptus			
Orchidaceae	Lepanthes acuminata			
Orchidaceae	Lepanthes acuminata subsp. acuminata		VU	
Orchidaceae	Lepanthes ancylopetala	NT	CR	YES
Orchidaceae	Lepanthes appendiculata		VU	
Orchidaceae	Lepanthes avis		VU	
Orchidaceae	Lepanthes breedlovei		CR	YES
Orchidaceae	Lepanthes disticha			
Orchidaceae	Lepanthes erythroxantha		EN	
Orchidaceae	Lepanthes guatemalensis	NT	EN	
Orchidaceae	Lepanthes johnsonii		EN	YES
Orchidaceae	Lepanthes maxima		EN	YES
Orchidaceae	Lepanthes minima		CR	YES
Orchidaceae	Lepanthes moorei		EN	
Orchidaceae	Lepanthes oreocharis		EN	
Orchidaceae	Lepanthes oreophila		CR	YES
Orchidaceae	Lepanthes papillipetala		EN	
Orchidaceae	Lepanthes parvula	NT	EN	YES
Orchidaceae	Lepanthes pristidis		VU	
Orchidaceae	Lepanthes rekoi		EN	
Orchidaceae	Lepanthes samacensis		EN	
Orchidaceae	Lepanthes schiedei		EN	
Orchidaceae	Lepanthes schultesii		EN	
Orchidaceae	Lepanthes scopula		VU	
Orchidaceae	Lepanthes stenophylla		EN	
Orchidaceae	Lepanthes suarezii		EN	
Orchidaceae	Lepanthes tecpanica		EN	
Orchidaceae	Lepanthes tenuiloba			

Orchidaceae	Lepanthes thurstoniorum		EN	
Orchidaceae	Lepanthes totontepecensis		CR	YES
Orchidaceae	Lepanthes turialvae		EN	YES
Orchidaceae	Lepanthes williamsii		CR	
Orchidaceae	Lepanthes yunckeri		CR	YES
Orchidaceae	Lepanthopsis floripecten	NT	EN	
Orchidaceae	Liparis alata		EN	
Orchidaceae	Liparis cordiformis			
Orchidaceae	Liparis fantastica		EN	
Orchidaceae	Liparis lindeniana		EN	
Orchidaceae	Liparis nervosa subsp. nervosa		VU	
Orchidaceae	Lockhartia oerstedii			
Orchidaceae	Lycaste aromatica			
Orchidaceae	Lycaste bradeorum		EN	YES
Orchidaceae	Lycaste cochleata			
Orchidaceae	Lycaste consobrina			
Orchidaceae	Lycaste cruenta			
Orchidaceae	Lycaste deppei			
Orchidaceae	Lycaste virginalis			
Orchidaceae	Macradenia brassavolae	NT	VU	
Orchidaceae	Macroclinium bicolor		VU	
Orchidaceae	Malaxis aurea			
Orchidaceae	Malaxis brachyrrhynchos			
Orchidaceae	Malaxis brachystachys			
Orchidaceae	Malaxis excavata			
Orchidaceae	Malaxis fastigiata			
Orchidaceae	Malaxis greenwoodiana	NT	EN	
Orchidaceae	Malaxis histionantha			
Orchidaceae	Malaxis lepanthiflora			
Orchidaceae	Malaxis lepidota			
Orchidaceae	Malaxis macrostachya			
Orchidaceae	Malaxis maianthemifolia			
Orchidaceae	Malaxis maxonii		EN	
Orchidaceae	Malaxis streptopetala			

Orchidaceae	Malaxis triangularis		EN	
Orchidaceae	Malaxis unifolia			
Orchidaceae	Masdevallia floribunda			
Orchidaceae	Masdevallia tubuliflora		CR	YES
Orchidaceae	Masdevallia tuerckheimii			
Orchidaceae	Maxillaria aciantha			
Orchidaceae	Maxillaria acutifolia		EN	
Orchidaceae	Maxillaria alba	NT	EN	
Orchidaceae	Maxillaria anceps			
Orchidaceae	Maxillaria cobanensis		VU	
Orchidaceae	Maxillaria crassifolia			
Orchidaceae	Maxillaria cucullata			
Orchidaceae	Maxillaria curtipes		VU	
Orchidaceae	Maxillaria densa			
Orchidaceae	Maxillaria discolor		CR	YES
Orchidaceae	Maxillaria egertoniana			
Orchidaceae	Maxillaria elata		CR	YES
Orchidaceae	Maxillaria elatior			
Orchidaceae	Maxillaria friedrichsthalii			
Orchidaceae	Maxillaria hagsateriana			
Orchidaceae	Maxillaria hedwigiae			
Orchidaceae	Maxillaria histrionica		EN	
Orchidaceae	Maxillaria lineolata			
Orchidaceae	Maxillaria lutescens		EN	YES
Orchidaceae	Maxillaria macleei			
Orchidaceae	Maxillaria maleolens		VU	
Orchidaceae	Maxillaria meleagris			
Orchidaceae	Maxillaria nagelii			
Orchidaceae	Maxillaria nasuta	NT	EN	
Orchidaceae	Maxillaria parviflora			
Orchidaceae	Maxillaria pauciflora		EN	
Orchidaceae	Maxillaria praestans		EN	
Orchidaceae	Maxillaria pulchra			
Orchidaceae	Maxillaria ringens			

Orchidaceae	Maxillaria sotoana		EN	YES
Orchidaceae	Maxillaria tenuifolia			
Orchidaceae	Maxillaria tonsoniae	NT		
Orchidaceae	Maxillaria uncata			
Orchidaceae	Maxillaria variabilis			
Orchidaceae	Meiracyllium gemma			
Orchidaceae	Meiracyllium trinasutum			
Orchidaceae	Mesadenella petenensis			
Orchidaceae	Mesadenella tonduzii		EN	
Orchidaceae	Mesadenus lucayanus			
Orchidaceae	Mesadenus polyanthus			
Orchidaceae	Microchilus lunifer		VU	
Orchidaceae	Microthelys minutiflora			
Orchidaceae	Microthelys rubrocalosa			
Orchidaceae	Mormodes aromatica		VU	
Orchidaceae	Mormodes dayana		CR	YES
Orchidaceae	Mormodes ignea		EN	
Orchidaceae	Mormodes lineata			
Orchidaceae	Mormodes maculata			
Orchidaceae	Mormodes maculata var. unicolor	VU		
Orchidaceae	Mormodes nagelii		VU	
Orchidaceae	Mormodes sotoana	EN	EN	YES
Orchidaceae	Mormodes tezontle		EN	
Orchidaceae	Mormodes tuxtlensis		VU	
Orchidaceae	Muscarella fimbriata	NT	EN	
Orchidaceae	Muscarella marginata		VU	
Orchidaceae	Muscarella segregatifolia		VU	
Orchidaceae	Myoxanthus congestus		VU	
Orchidaceae	Myrmecophila galeottiana			
Orchidaceae	Myrmecophila grandiflora			
Orchidaceae	Myrmecophila tibicinis			
Orchidaceae	Nemaconia glomerata			
Orchidaceae	Nemaconia graminifolia			
Orchidaceae	Nemaconia striata			

Orchidaceae	Nidema boothii			
Orchidaceae	Notylia barkeri			
Orchidaceae	Notylia leucantha		CR	YES
Orchidaceae	Notylia orbicularis			
Orchidaceae	Notylia orbicularis subsp. orbicularis			
Orchidaceae	Notylia trisepala		VU	
Orchidaceae	Oeceoclades maculata			
Orchidaceae	Oestlundia distantiflora	NT	VU	
Orchidaceae	Oestlundia luteorosea			
Orchidaceae	Oncidium altissimum		VU	
Orchidaceae	Oncidium ansiferum		EN	
Orchidaceae	Oncidium cheirophorum		EN	
Orchidaceae	Oncidium endocharis	VU	VU	
Orchidaceae	Oncidium graminifolium			
Orchidaceae	Oncidium hintonii			
Orchidaceae	Oncidium incurvum	VU		
Orchidaceae	Oncidium laeve			
Orchidaceae	Oncidium lepidum		EN	
Orchidaceae	Oncidium leucochilum	VU		
Orchidaceae	Oncidium maculatum			
Orchidaceae	Oncidium oblongatum			
Orchidaceae	Oncidium oliganthum			
Orchidaceae	Oncidium ornithorhynchum			
Orchidaceae	Oncidium pergameneum		EN	
Orchidaceae	Oncidium poikilostalix	EN	EN	
Orchidaceae	Oncidium reflexum			
Orchidaceae	Oncidium sotoanum			
Orchidaceae	Oncidium sphacelatum			
Orchidaceae	Oncidium stenoglossum		VU	
Orchidaceae	Oncidium tenuipes			
Orchidaceae	Oncidium wentworthianum	NT		
Orchidaceae	Ornithocephalus bicornis		EN	
Orchidaceae	Ornithocephalus dolabratus		EN	
Orchidaceae	Ornithocephalus inflexus			

Orchidaceae	Ornithocephalus iridifolius			VU	
Orchidaceae	Ornithocephalus obergiae			EN	
Orchidaceae	Ornithocephalus tripterus				
Orchidaceae	Pelexia adnata			VU	
Orchidaceae	Pelexia funckiana				
Orchidaceae	Pelexia gutturosa				
Orchidaceae	Phloeophila peperomioides			CR	YES
Orchidaceae	Phragmipedium caudatum		EN	CR	YES
Orchidaceae	Phragmipedium humboldtii var. exstaminodium			EN	
Orchidaceae	Platanthera brevifolia				
Orchidaceae	Platystele caudatisepala	VU		EN	YES
Orchidaceae	Platystele compacta			VU	
Orchidaceae	Platystele jungermannioides	VU		EN	YES
Orchidaceae	Platystele lancilabris			EN	
Orchidaceae	Platystele minimiflora			VU	
Orchidaceae	Platystele ornata			VU	
Orchidaceae	Platystele ovatilabia				
Orchidaceae	Platystele oxyglossa			VU	
Orchidaceae	Platystele pedicellaris			CR	YES
Orchidaceae	Platystele stenostachya				
Orchidaceae	Pleurothallis bivalvis				
Orchidaceae	Pleurothallis cardiothallis				
Orchidaceae	Pleurothallis correllii			VU	
Orchidaceae	Pleurothallis homalantha			EN	
Orchidaceae	Pleurothallis leucantha				
Orchidaceae	Pleurothallis matudana				
Orchidaceae	Pleurothallis oncoglossa			CR	YES
Orchidaceae	Pleurothallis pansamalae				
Orchidaceae	Pleurothallis quadrifida				
Orchidaceae	Pleurothallis sanchoi			EN	
Orchidaceae	Pleurothallis tipuloides			VU	
Orchidaceae	Polystachya cerea				
Orchidaceae	Polystachya clavata				
Orchidaceae	Polystachya concreta			EN	

Orchidaceae	Polystachya foliosa			
Orchidaceae	Polystachya lineata		VU	
Orchidaceae	Polystachya masayensis		CR	YES
Orchidaceae	Ponera juncifolia			
Orchidaceae	Ponthieva ephippium			
Orchidaceae	Ponthieva mexicana			
Orchidaceae	Ponthieva parvula			
Orchidaceae	Ponthieva racemosa			
Orchidaceae	Ponthieva rinconii		EN	
Orchidaceae	Ponthieva schaffneri			
Orchidaceae	Ponthieva triloba		EN	
Orchidaceae	Ponthieva trilobata			
Orchidaceae	Prescottia stachyodes			
Orchidaceae	Prosthechea baculus			
Orchidaceae	Prosthechea boothiana			
Orchidaceae	Prosthechea brassavolae			
Orchidaceae	Prosthechea chacaoensis			
Orchidaceae	Prosthechea chondylobulbon			
Orchidaceae	Prosthechea citrina	NT		
Orchidaceae	Prosthechea cochleata			
Orchidaceae	Prosthechea cochleata var. cochleata			
Orchidaceae	Prosthechea concolor			
Orchidaceae	Prosthechea glauca			
Orchidaceae	Prosthechea guttata			
Orchidaceae	Prosthechea ionophlebia		VU	
Orchidaceae	Prosthechea livida			
Orchidaceae	Prosthechea mariae	VU		
Orchidaceae	Prosthechea michuacana			
Orchidaceae	Prosthechea neurosa	NT		
Orchidaceae	Prosthechea obpiribulbon			
Orchidaceae	Prosthechea ochracea			
Orchidaceae	Prosthechea panthera			
Orchidaceae	Prosthechea pseudopygmaea			
Orchidaceae	Prosthechea pterocarpa			

Orchidaceae	Prosthechea pygmaea			
Orchidaceae	Prosthechea radiata			
Orchidaceae	Prosthechea rhynchophora			
Orchidaceae	Prosthechea varicosa			
Orchidaceae	Prosthechea vitellina	NT		
Orchidaceae	Pseudogoodyera wrightii	NT	VU	
Orchidaceae	Psilochilus macrophyllus			
Orchidaceae	Psychilis atropurpurea		CR	YES
Orchidaceae	Pteroglossa roseoalba		VU	
Orchidaceae	Restrepia muscifera			
Orchidaceae	Restrepia trichoglossa	VU	VU	
Orchidaceae	Restrepiella ophiocephala			
Orchidaceae	Rhyncholaelia glauca			
Orchidaceae	Rhynchostele bictoniensis			
Orchidaceae	Rhynchostele candidula			
Orchidaceae	Rhynchostele cervantesii	VU		
Orchidaceae	Rhynchostele cordata	VU		
Orchidaceae	Rhynchostele ehrenbergii	VU		
Orchidaceae	Rhynchostele maculata			
Orchidaceae	Rhynchostele majalis	EN	EN	
Orchidaceae	Rhynchostele pygmaea	NT	VU	
Orchidaceae	Rhynchostele rossii	VU		
Orchidaceae	Rhynchostele stellata			
Orchidaceae	Rossioglossum grande	EN	EN	
Orchidaceae	Rossioglossum williamsianum	EN	EN	
Orchidaceae	Sacoila lanceolata			
Orchidaceae	Sarcoglottis acaulis			
Orchidaceae	Sarcoglottis assurgens			
Orchidaceae	Sarcoglottis cerina	NT	VU	
Orchidaceae	Sarcoglottis rosulata			
Orchidaceae	Sarcoglottis sceptrodes			
Orchidaceae	Sarcoglottis schaffneri			
Orchidaceae	Scaphosepalum microdactylum		VU	
Orchidaceae	Scaphyglottis behrii			

Orchidaceae	Scaphyglottis cernua		CR	YES
Orchidaceae	Scaphyglottis confusa			
Orchidaceae	Scaphyglottis crurigera			
Orchidaceae	Scaphyglottis fasciculata			
Orchidaceae	Scaphyglottis graminifolia		VU	
Orchidaceae	Scaphyglottis imbricata			
Orchidaceae	Scaphyglottis leucantha			
Orchidaceae	Scaphyglottis lindeniana			
Orchidaceae	Scaphyglottis livida			
Orchidaceae	Scaphyglottis longicaulis		CR	YES
Orchidaceae	Scaphyglottis minutiflora			
Orchidaceae	Scaphyglottis prolifera		VU	
Orchidaceae	Schiedeella affinis			
Orchidaceae	Schiedeella crenulata		VU	
Orchidaceae	Schiedeella saltensis			
Orchidaceae	Schiedeella trilineata		VU	
Orchidaceae	Schiedeella violacea			
Orchidaceae	Sobralia crispissima		CR	YES
Orchidaceae	Sobralia decora			
Orchidaceae	Sobralia fragrans			
Orchidaceae	Sobralia galeottiana			
Orchidaceae	Sobralia lindleyana	NT	EN	YES
Orchidaceae	Sobralia macdougallii		VU	
Orchidaceae	Sobralia macrantha			
Orchidaceae	Sobralia mucronata	NT	EN	YES
Orchidaceae	Sobralia warszewiczii		EN	
Orchidaceae	Sobralia xantholeuca			
Orchidaceae	Specklinia blancoi		CR	YES
Orchidaceae	Specklinia brighamii			
Orchidaceae	Specklinia corniculata		EN	
Orchidaceae	Specklinia digitale	VU	VU	
Orchidaceae	Specklinia endotrachys			
Orchidaceae	Specklinia fuegi			
Orchidaceae	Specklinia grobyi			

Orchidaceae	Specklinia lanceola		EN	
Orchidaceae	Specklinia microphylla		EN	
Orchidaceae	Specklinia picta			
Orchidaceae	Specklinia pisinna			
Orchidaceae	Specklinia tribuloides			
Orchidaceae	Specklinia vittariifolia		CR	YES
Orchidaceae	Specklinia yucatanensis			
Orchidaceae	Spiranthes nebulorum		VU	
Orchidaceae	Stanhopea dodsoniana			
Orchidaceae	Stanhopea ecornuta	VU	VU	
Orchidaceae	Stanhopea graveolens			
Orchidaceae	Stanhopea hernandezii			
Orchidaceae	Stanhopea oculata	VU		
Orchidaceae	Stanhopea ruckeri			
Orchidaceae	Stanhopea saccata			
Orchidaceae	Stanhopea tigrina	VU		
Orchidaceae	Stanhopea whittenii		EN	
Orchidaceae	Stelis aemula		EN	
Orchidaceae	Stelis chihobensis	NT	EN	
Orchidaceae	Stelis ciliaris			
Orchidaceae	Stelis cobanensis	NT		
Orchidaceae	Stelis deregularis	NT	EN	
Orchidaceae	Stelis despectans		EN	
Orchidaceae	Stelis emarginata			
Orchidaceae	Stelis gracilis			
Orchidaceae	Stelis hymenantha			
Orchidaceae	Stelis immersa			
Orchidaceae	Stelis jalapensis		EN	
Orchidaceae	Stelis martinezii		VU	
Orchidaceae	Stelis megachlamys			
Orchidaceae	Stelis microchila		VU	
Orchidaceae	Stelis nagelii		VU	
Orchidaceae	Stelis oaxacana		VU	
Orchidaceae	Stelis ovatilabia		VU	

Orchidaceae	Stelis oxypetala			
Orchidaceae	Stelis pachyglossa			
Orchidaceae	Stelis pardipes			
Orchidaceae	Stelis parvula			
Orchidaceae	Stelis platystylis			
Orchidaceae	Stelis poasensis			
Orchidaceae	Stelis punctulata		EN	
Orchidaceae	Stelis purpurascens			
Orchidaceae	Stelis rubens			
Orchidaceae	Stelis sclerophylla		CR	
Orchidaceae	Stelis segoviensis		VU	
Orchidaceae	Stelis superbiens		VU	
Orchidaceae	Stelis tenuissima		VU	
Orchidaceae	Stelis tridentata		CR	YES
Orchidaceae	Stelis veracrucensis		VU	
Orchidaceae	Stelis villosa			
Orchidaceae	Stenorrhynchos speciosum			
Orchidaceae	Stenotyla helleri		EN	
Orchidaceae	Stenotyla lendyana			
Orchidaceae	Teuscheria pickiana	NT	EN	
Orchidaceae	Tolumnia guttata		EN	
Orchidaceae	Trichocentrum andreanum			
Orchidaceae	Trichocentrum ascendens			
Orchidaceae	Trichocentrum bicallosum			
Orchidaceae	Trichocentrum biorbiculare			
Orchidaceae	Trichocentrum brachyphyllum			
Orchidaceae	Trichocentrum candidum			
Orchidaceae	Trichocentrum capistratum		CR	YES
Orchidaceae	Trichocentrum carthagenense			
Orchidaceae	Trichocentrum cavendishianum			
Orchidaceae	Trichocentrum cebolleta			
Orchidaceae	Trichocentrum cosymbephorum			
Orchidaceae	Trichocentrum lindenii			
Orchidaceae	Trichocentrum luridum			

Orchidaceae	Trichocentrum microchilum				
Orchidaceae	Trichocentrum morenoi			CR	
Orchidaceae	Trichocentrum oerstedii				
Orchidaceae	Trichocentrum stramineum	EN			
Orchidaceae	Trichocentrum undulatum			EN	
Orchidaceae	Trichopilia galeottiana	EN		EN	
Orchidaceae	Trichopilia tortilis				
Orchidaceae	Trichosalpinx blaisdellii				
Orchidaceae	Trichosalpinx ciliaris				
Orchidaceae	Trichosalpinx dura			VU	
Orchidaceae	Trichosalpinx memor			VU	
Orchidaceae	Trichosalpinx trachystoma			CR	YES
Orchidaceae	Triphora debilis			VU	
Orchidaceae	Triphora gentianoides				
Orchidaceae	Triphora trianthophoros subsp. mexicana				
Orchidaceae	Triphora wagneri			EN	
Orchidaceae	Tropidia polystachya				
Orchidaceae	Vanilla cribbiana		CR	VU	
Orchidaceae	Vanilla inodora		EN		
Orchidaceae	Vanilla mexicana			EN	
Orchidaceae	Vanilla odorata		EN		
Orchidaceae	Vanilla planifolia	NT	EN		
Orchidaceae	Vanilla pompona		EN		
Orchidaceae	Warrea costaricensis	VU		EN	YES
Orchidaceae	Wullschlaegelia aphylla			EN	
Orchidaceae	Xylobium elongatum				
Orchidaceae	Xylobium foveatum			VU	
Orchidaceae	Xylobium sulfurinum				
Orobanchaceae	Agalinis maritima			VU	
Orobanchaceae	Agalinis maritima var. grandiflora				
Orobanchaceae	Agalinis peduncularis				
Orobanchaceae	Agalinis purpurea			EN	
Orobanchaceae	Aureolaria greggii				
Orobanchaceae	Buchnera obliqua				

Orobanchaceae	Buchnera palustris	CR	YES
Orobanchaceae	Buchnera pusilla		
Orobanchaceae	Castilleja arvensis		
Orobanchaceae	Castilleja auriculata		
Orobanchaceae	Castilleja integra		
Orobanchaceae	Castilleja integrifolia		
Orobanchaceae	Castilleja integrifolia var. chiapensis		
Orobanchaceae	Castilleja longiflora	VU	
Orobanchaceae	Castilleja moranensis		
Orobanchaceae	Castilleja moranensis var. cinerascens	EN	
Orobanchaceae	Castilleja nitricola	VU	
Orobanchaceae	Castilleja nivibractea	EN	YES
Orobanchaceae	Castilleja porphyrosceptron	CR	YES
Orobanchaceae	Castilleja schaffneri	VU	
Orobanchaceae	Castilleja scorzonerifolia		
Orobanchaceae	Castilleja tenuiflora		
Orobanchaceae	Castilleja tenuifolia		
Orobanchaceae	Castilleja zempoaltepetlensis	VU	
Orobanchaceae	Conopholis alpina		
Orobanchaceae	Conopholis americana		
Orobanchaceae	Epifagus virginiana	EN	
Orobanchaceae	Escobedia crassipes		
Orobanchaceae	Escobedia grandiflora		
Orobanchaceae	Escobedia guatemalensis	EN	
Orobanchaceae	Escobedia laevis		
Orobanchaceae	Lamourouxia dasyantha		
Orobanchaceae	Lamourouxia lanceolata		
Orobanchaceae	Lamourouxia longiflora		
Orobanchaceae	Lamourouxia longiflora var. integerrima	EN	
Orobanchaceae	Lamourouxia macrantha		
Orobanchaceae	Lamourouxia microphylla	VU	
Orobanchaceae	Lamourouxia multifida		
Orobanchaceae	Lamourouxia nelsonii		
Orobanchaceae	Lamourouxia paneroi	VU	

Orobanchaceae	Lamourouxia pringlei	
Orobanchaceae	Lamourouxia rhinanthifolia	
Orobanchaceae	Lamourouxia viscosa	
Orobanchaceae	Lamourouxia xalapensis	
Orobanchaceae	Melasma melampyroides	EN
Orobanchaceae	Melasma physalodes	
Orobanchaceae	Orobanche ludoviciana	
Orobanchaceae	Pedicularis angustifolia	
Orobanchaceae	Pedicularis canadensis	
Orobanchaceae	Seymeria decurva	
Orobanchaceae	Seymeria laciniata	
Orobanchaceae	Silviella prostrata	
Osmundaceae	Osmunda regalis	
Osmundaceae	Osmundastrum cinnamomeum	VU
Oxalidaceae	Averrhoa carambola	
Oxalidaceae	Biophytum cowanii	EN
Oxalidaceae	Biophytum dendroides	
Oxalidaceae	Biophytum sensitivum	EN
Oxalidaceae	Oxalis acuminata	
Oxalidaceae	Oxalis albicans	
Oxalidaceae	Oxalis alpina	
Oxalidaceae	Oxalis bipartita	VU
Oxalidaceae	Oxalis clematodes	VU
Oxalidaceae	Oxalis corniculata	
Oxalidaceae	Oxalis decaphylla	
Oxalidaceae	Oxalis dimidiata	
Oxalidaceae	Oxalis divergens	
Oxalidaceae	Oxalis drummondii	
Oxalidaceae	Oxalis frutescens	
Oxalidaceae	Oxalis frutescens subsp. angustifolia	
Oxalidaceae	Oxalis latifolia	
Oxalidaceae	Oxalis latifolia subsp. vespertilionis	EN
Oxalidaceae	Oxalis lunulata	
Oxalidaceae	Oxalis microcarpa	VU

Oxalidaceae	Oxalis nelsonii		
Oxalidaceae	Oxalis pilosa		
Oxalidaceae	Oxalis rhombifolia		
Oxalidaceae	Oxalis sellowiana		VU
Oxalidaceae	Oxalis sepium		EN
Oxalidaceae	Oxalis spiralis		EN
Oxalidaceae	Oxalis stricta		
Oxalidaceae	Oxalis tetraphylla		
Oxalidaceae	Oxalis tuberosa		EN
Oxalidaceae	Oxalis violacea		
Papaveraceae	Argemone echinata		
Papaveraceae	Argemone grandiflora		
Papaveraceae	Argemone mexicana		
Papaveraceae	Argemone ochroleuca		
Papaveraceae	Argemone platyceras		
Papaveraceae	Bocconia arborea		
Papaveraceae	Bocconia frutescens		
Papaveraceae	Bocconia glaucifolia	VU	EN
Papaveraceae	Bocconia gracilis	VU	
Papaveraceae	Bocconia hintoniorum	EN	EN
Papaveraceae	Bocconia integrifolia		VU
Papaveraceae	Bocconia vulcanica	EN	
Papaveraceae	Corydalis pseudomicrantha		
Papaveraceae	Eschscholzia californica		
Papaveraceae	Papaver rhoeas		
Passifloraceae	Erblichia odorata		
Passifloraceae	Passiflora adenopoda		
Passifloraceae	Passiflora alata		
Passifloraceae	Passiflora ambigua		
Passifloraceae	Passiflora apetala		EN
Passifloraceae	Passiflora biflora		
Passifloraceae	Passiflora bryonioides		
Passifloraceae	Passiflora caerulea		
Passifloraceae	Passiflora ciliata		

Passifloraceae	Passiflora cobanensis	
Passifloraceae	Passiflora complanata	VU
Passifloraceae	Passiflora conzattiana	
Passifloraceae	Passiflora coriacea	
Passifloraceae	Passiflora costaricensis	
Passifloraceae	Passiflora dolichocarpa	EN
Passifloraceae	Passiflora edulis	
Passifloraceae	Passiflora exsudans	
Passifloraceae	Passiflora filipes	
Passifloraceae	Passiflora foetida	
Passifloraceae	Passiflora goniosperma	
Passifloraceae	Passiflora guatemalensis	
Passifloraceae	Passiflora hahnii	
Passifloraceae	Passiflora helleri	
Passifloraceae	Passiflora hibiscifolia	
Passifloraceae	Passiflora holosericea	
Passifloraceae	Passiflora incarnata	
Passifloraceae	Passiflora jorullensis	
Passifloraceae	Passiflora jorullensis var. salvadorensis	
Passifloraceae	Passiflora karwinskii	
Passifloraceae	Passiflora lancearia	EN
Passifloraceae	Passiflora lancetillensis	EN
Passifloraceae	Passiflora ligularis	
Passifloraceae	Passiflora manantlanensis	VU
Passifloraceae	Passiflora membranacea	
Passifloraceae	Passiflora mexicana	
Passifloraceae	Passiflora microstipula	EN
Passifloraceae	Passiflora nelsonii	VU
Passifloraceae	Passiflora oerstedii	
Passifloraceae	Passiflora ornithoura	VU
Passifloraceae	Passiflora ornithoura var. chiapasensis	VU
Passifloraceae	Passiflora pallida	
Passifloraceae	Passiflora pendens	EN
Passifloraceae	Passiflora pilosa subsp. dimidiata	VU

Passifloraceae	Passiflora platyloba		EN	
Passifloraceae	Passiflora porophylla		CR	YES
Passifloraceae	Passiflora porphyretica			
Passifloraceae	Passiflora rovirosae			
Passifloraceae	Passiflora serratifolia			
Passifloraceae	Passiflora sexflora			
Passifloraceae	Passiflora sexocellata			
Passifloraceae	Passiflora sicyoides			
Passifloraceae	Passiflora standleyi			
Passifloraceae	Passiflora suberosa			
Passifloraceae	Passiflora suberosa subsp. litoralis			
Passifloraceae	Passiflora subpeltata			
Passifloraceae	Passiflora viridiflora			
Passifloraceae	Passiflora vitifolia			
Passifloraceae	Piriqueta cistoides			
Passifloraceae	Piriqueta mexicana		EN	
Passifloraceae	Turnera coerulea			
Passifloraceae	Turnera curassavica		VU	
Passifloraceae	Turnera diffusa			
Passifloraceae	Turnera scabra			
Passifloraceae	Turnera ulmifolia			
Passifloraceae	Turnera velutina			
Passifloraceae	Turnera violacea		EN	
Pentaphylacaceae	Cleyera theoides			
Pentaphylacaceae	Freziera candicans		VU	
Pentaphylacaceae	Freziera grisebachii		EN	
Pentaphylacaceae	Freziera guatemalensis			
Pentaphylacaceae	Symplococarpon flavifolium	EN	VU	
Pentaphylacaceae	Symplococarpon purpusii			
Pentaphylacaceae	Ternstroemia huasteca	VU		
Pentaphylacaceae	Ternstroemia lineata			
Pentaphylacaceae	Ternstroemia lineata subsp. chalicophila			
Pentaphylacaceae	Ternstroemia sylvatica			
Pentaphylacaceae	Ternstroemia tepezapote			

Peraceae	Pera arborea		CR	
Peraceae	Pera barbellata	EN		
Petenaeaceae	Petenaea cordata	EN		
Petiveriaceae	Petiveria alliacea			
Petiveriaceae	Rivina humilis			
Petiveriaceae	Trichostigma octandrum			
Phrymaceae	Erythranthe geyeri			
Phrymaceae	Erythranthe glabrata			
Phrymaceae	Erythranthe orizabae		VU	
Phrymaceae	Hemichaena fruticosa			
Phrymaceae	Hemichaena levigata			
Phrymaceae	Leucocarpus perfoliatus			
Phyllanthaceae	Astrocasia neurocarpa			
Phyllanthaceae	Astrocasia peltata			
Phyllanthaceae	Astrocasia tremula			
Phyllanthaceae	Hieronyma oblonga			
Phyllanthaceae	Margaritaria nobilis			
Phyllanthaceae	Meineckia neogranatensis		EN	
Phyllanthaceae	Phyllanthus acuminatus			
Phyllanthaceae	Phyllanthus adenodiscus			
Phyllanthaceae	Phyllanthus amarus			
Phyllanthaceae	Phyllanthus barbarae			
Phyllanthaceae	Phyllanthus brasiliensis			
Phyllanthaceae	Phyllanthus caroliniensis			
Phyllanthaceae	Phyllanthus coalcomanensis	EN		
Phyllanthaceae	Phyllanthus compressus			
Phyllanthaceae	Phyllanthus galeottianus			
Phyllanthaceae	Phyllanthus grandifolius			
Phyllanthaceae	Phyllanthus graveolens subsp. micrandrus			
Phyllanthaceae	Phyllanthus heterotrichus		CR	YES
Phyllanthaceae	Phyllanthus hexadactylus		VU	
Phyllanthaceae	Phyllanthus hyssopifolioides		EN	
Phyllanthaceae	Phyllanthus laxiflorus			
Phyllanthaceae	Phyllanthus liebmannianus			

Phyllanthaceae	Phyllanthus liebmannianus subsp. liebmannianus				
Phyllanthaceae	Phyllanthus mcvaughii				
Phyllanthaceae	Phyllanthus niruri				
Phyllanthaceae	Phyllanthus niruri subsp. lathyroides				
Phyllanthaceae	Phyllanthus purpusii				
Phyllanthaceae	Phyllanthus stipulatus				
Phyllanthaceae	Phyllanthus tuerckheimii			VU	
Phyllanthaceae	Phyllanthus urinaria				
Phyllanthaceae	Savia sessiliflora				
Phyllonomaceae	Phyllonoma laticuspis				
Phyllonomaceae	Phyllonoma ruscifolia			EN	
Phytolaccaceae	Agdestis clematidea				
Phytolaccaceae	Phytolacca americana				
Phytolaccaceae	Phytolacca dodecandra			CR	YES
Phytolaccaceae	Phytolacca icosandra				
Phytolaccaceae	Phytolacca octandra				
Phytolaccaceae	Phytolacca rivinoides				
Phytolaccaceae	Phytolacca rugosa				
Picramniaceae	Alvaradoa amorphoides				
Picramniaceae	Picramnia antidesma				
Picramniaceae	Picramnia antidesma subsp. fessonia				
Picramniaceae	Picramnia deflexa		EN		
Picramniaceae	Picramnia guerrerensis		EN		
Picramniaceae	Picramnia hirsuta		VU		
Picramniaceae	Picramnia latifolia			EN	
Picramniaceae	Picramnia matudae				
Picramniaceae	Picramnia polyantha				
Picramniaceae	Picramnia teapensis				
Picramniaceae	Picramnia xalapensis		VU		
Pinaceae	Abies durangensis				
Pinaceae	Abies flinckii				
Pinaceae	Abies guatemalensis	EN	EN		
Pinaceae	Abies hickelii	EN	EN		
Pinaceae	Abies religiosa				

Pinaceae	Abies vejarii	VU		
Pinaceae	Pinus ayacahuite			
Pinaceae	Pinus caribaea			
Pinaceae	Pinus cembroides			
Pinaceae	Pinus cembroides subsp. orizabensis			
Pinaceae	Pinus chiapensis			
Pinaceae	Pinus devoniana			
Pinaceae	Pinus douglasiana			
Pinaceae	Pinus greggii		VU	
Pinaceae	Pinus hartwegii			
Pinaceae	Pinus herrerae			
Pinaceae	Pinus lawsonii			
Pinaceae	Pinus leiophylla			
Pinaceae	Pinus maximinoi			
Pinaceae	Pinus montezumae			
Pinaceae	Pinus nelsonii	EN	EN	
Pinaceae	Pinus oocarpa			
Pinaceae	Pinus patula			
Pinaceae	Pinus pringlei			
Pinaceae	Pinus pseudostrobus			
Pinaceae	Pinus pseudostrobus var. apulcensis			
Pinaceae	Pinus rzedowskii	EN	VU	VU
Pinaceae	Pinus strobus			
Pinaceae	Pinus tecunumanii		VU	
Pinaceae	Pinus teocote			
Piperaceae	Peperomia acuminata			VU
Piperaceae	Peperomia adscendens			
Piperaceae	Peperomia alata			
Piperaceae	Peperomia alpina			
Piperaceae	Peperomia angularis			
Piperaceae	Peperomia angustata			
Piperaceae	Peperomia arboricola			
Piperaceae	Peperomia asarifolia			
Piperaceae	Peperomia berlandieri			

Piperaceae	Peperomia blanda			
Piperaceae	Peperomia botterii		VU	
Piperaceae	Peperomia bracteata			
Piperaceae	Peperomia camptotricha		VU	
Piperaceae	Peperomia castilloi		CR	YES
Piperaceae	Peperomia chimboana		EN	YES
Piperaceae	Peperomia clavigera			
Piperaceae	Peperomia claytonioides		VU	
Piperaceae	Peperomia coatzacoalcosensis		EN	
Piperaceae	Peperomia cobana			
Piperaceae	Peperomia cocleana		CR	YES
Piperaceae	Peperomia consoquitlana			
Piperaceae	Peperomia cordovana		EN	
Piperaceae	Peperomia cyclophylla			
Piperaceae	Peperomia dendrophila			
Piperaceae	Peperomia deppeana			
Piperaceae	Peperomia distachyos		CR	YES
Piperaceae	Peperomia donaguiana			
Piperaceae	Peperomia dorstenioides		VU	
Piperaceae	Peperomia edulis		VU	
Piperaceae	Peperomia emarginata		EN	
Piperaceae	Peperomia emarginella		VU	
Piperaceae	Peperomia emiliana		VU	
Piperaceae	Peperomia epidendron		VU	
Piperaceae	Peperomia galioides			
Piperaceae	Peperomia glabella			
Piperaceae	Peperomia glandulosa	CR	CR	YES
Piperaceae	Peperomia granulosa			
Piperaceae	Peperomia griggsii		CR	YES
Piperaceae	Peperomia guatemalensis			
Piperaceae	Peperomia hernandiifolia			
Piperaceae	Peperomia heterodoxa			
Piperaceae	Peperomia heterophylla			
Piperaceae	Peperomia hirta			

Piperaceae	Peperomia hirtipetiola	CR	
Piperaceae	Peperomia hispidula		
Piperaceae	Peperomia hispiduliformis		
Piperaceae	Peperomia hobbitoides	EN	
Piperaceae	Peperomia hoffmannii		
Piperaceae	Peperomia hondoana	VU	
Piperaceae	Peperomia humilis		
Piperaceae	Peperomia lanceolatopeltata		
Piperaceae	Peperomia lancifolia		
Piperaceae	Peperomia lenticularis		
Piperaceae	Peperomia leptophylla		
Piperaceae	Peperomia liebmannii		
Piperaceae	Peperomia limana	EN	
Piperaceae	Peperomia lindeniana		
Piperaceae	Peperomia macrandra		
Piperaceae	Peperomia macrostachyos		
Piperaceae	Peperomia maculosa		
Piperaceae	Peperomia magnoliifolia		
Piperaceae	Peperomia magnoliifolia var. alata	CR	YES
Piperaceae	Peperomia matlalucaensis		
Piperaceae	Peperomia mexicana		
Piperaceae	Peperomia microphylla	EN	YES
Piperaceae	Peperomia naranjoana	VU	
Piperaceae	Peperomia non-alata	CR	YES
Piperaceae	Peperomia nopalana	CR	YES
Piperaceae	Peperomia obtusifolia		
Piperaceae	Peperomia olivacea		
Piperaceae	Peperomia parastriata	EN	YES
Piperaceae	Peperomia pedicellata		
Piperaceae	Peperomia pellucida		
Piperaceae	Peperomia peltilimba		
Piperaceae	Peperomia peltoidea	CR	
Piperaceae	Peperomia pereskiifolia		
Piperaceae	Peperomia pernambucensis	EN	

Piperaceae	Peperomia petrophila		
Piperaceae	Peperomia portobellensis		
Piperaceae	Peperomia pseudoalpina		
Piperaceae	Peperomia pseudopereskiifolia		
Piperaceae	Peperomia pubilimba	CR	YES
Piperaceae	Peperomia quadrifolia		
Piperaceae	Peperomia reflexa		
Piperaceae	Peperomia rhexiifolia		
Piperaceae	Peperomia rotundifolia		
Piperaceae	Peperomia san-carlosiana		
Piperaceae	Peperomia san-joseana		
Piperaceae	Peperomia santa-helenae	VU	
Piperaceae	Peperomia serpens		
Piperaceae	Peperomia subblanda		
Piperaceae	Peperomia succulenta		
Piperaceae	Peperomia suchitanensis	EN	
Piperaceae	Peperomia sylvestris	VU	
Piperaceae	Peperomia tenerrima		
Piperaceae	Peperomia tenuifolia	CR	
Piperaceae	Peperomia tetraphylla		
Piperaceae	Peperomia tuerckheimii		
Piperaceae	Peperomia tuisana		
Piperaceae	Peperomia umbilicata		
Piperaceae	Peperomia urocarpa		
Piperaceae	Peperomia xalana	EN	YES
Piperaceae	Peperomia zongolicana	CR	YES
Piperaceae	Piper aduncum		
Piperaceae	Piper aequale		
Piperaceae	Piper aereum	CR	YES
Piperaceae	Piper aeruginosibaccum		
Piperaceae	Piper amalago		
Piperaceae	Piper angustifolium	VU	
Piperaceae	Piper arboreum		
Piperaceae	Piper arieianum	EN	

Piperaceae	Piper atlantidanum	CR	YES
Piperaceae	Piper augustum	VU	
Piperaceae	Piper auritum		
Piperaceae	Piper berlandieri		
Piperaceae	Piper berteroanum	EN	
Piperaceae	Piper biauritum	EN	
Piperaceae	Piper bisasperatum		
Piperaceae	Piper bredemeyeri	VU	
Piperaceae	Piper brevipedicellatum		
Piperaceae	Piper carrilloanum	EN	
Piperaceae	Piper cayoense	VU	
Piperaceae	Piper chamissonis		
Piperaceae	Piper chinantlense		
Piperaceae	Piper commutatum		
Piperaceae	Piper cordovanum	VU	
Piperaceae	Piper crassicaule	CR	YES
Piperaceae	Piper cubilquitzianum		
Piperaceae	Piper curtispicum	EN	
Piperaceae	Piper curvatipes		
Piperaceae	Piper decipiens	VU	
Piperaceae	Piper decurrens	EN	
Piperaceae	Piper dilatatum		
Piperaceae	Piper disjunctum	VU	
Piperaceae	Piper divaricatum	EN	
Piperaceae	Piper donnell-smithii	EN	
Piperaceae	Piper dryadum	CR	
Piperaceae	Piper elasmophyllum	EN	
Piperaceae	Piper epigynium	EN	
Piperaceae	Piper evulsipilosum	VU	
Piperaceae	Piper flavidum		
Piperaceae	Piper fortunyoanum		
Piperaceae	Piper fraguanum		
Piperaceae	Piper georginum	EN	
Piperaceae	Piper gibbosum	EN	

Piperaceae	Piper glabratum	EN	
Piperaceae	Piper glabrescens		
Piperaceae	Piper grande	EN	
Piperaceae	Piper grandilimbum		
Piperaceae	Piper heydei	VU	
Piperaceae	Piper hispidum		
Piperaceae	Piper instabilipes	VU	
Piperaceae	Piper interruptum	EN	
Piperaceae	Piper irazuanum		
Piperaceae	Piper jacquemontianum		
Piperaceae	Piper jalapense	VU	
Piperaceae	Piper karwinskianum		
Piperaceae	Piper kerberi		
Piperaceae	Piper lacunosum		
Piperaceae	Piper lanceolatum	EN	
Piperaceae	Piper lapathifolium		
Piperaceae	Piper liebmannii	EN	
Piperaceae	Piper lindenianum	EN	
Piperaceae	Piper linearifolium		
Piperaceae	Piper luxii		
Piperaceae	Piper malpasoense	EN	
Piperaceae	Piper marginatum		
Piperaceae	Piper martensianum		
Piperaceae	Piper matudae	EN	
Piperaceae	Piper maxonii	VU	
Piperaceae	Piper melastomoides		
Piperaceae	Piper michelianum		
Piperaceae	Piper misantlense		
Piperaceae	Piper muelleri	VU	
Piperaceae	Piper neesianum		
Piperaceae	Piper nigrum	VU	
Piperaceae	Piper obliquum		
Piperaceae	Piper oradendron	CR	YES
Piperaceae	Piper orizabanum	EN	

Piperaceae	Piper otophorum	EN
Piperaceae	Piper papantlense	VU
Piperaceae	Piper patulum	
Piperaceae	Piper peltatum	
Piperaceae	Piper pergamentifolium	VU
Piperaceae	Piper phaeophyllum	EN
Piperaceae	Piper pinetorum	EN
Piperaceae	Piper populifolium	EN
Piperaceae	Piper pseudoasperifolium	
Piperaceae	Piper pseudofuligineum	
Piperaceae	Piper pseudolindenii	
Piperaceae	Piper psilorhachis	
Piperaceae	Piper purpusianum	VU
Piperaceae	Piper reticulatum	VU
Piperaceae	Piper sancti-felicis	
Piperaceae	Piper sanctum	
Piperaceae	Piper schiedeanum	
Piperaceae	Piper schippianum	VU
Piperaceae	Piper schlechtendalii	
Piperaceae	Piper stipulaceum	
Piperaceae	Piper subaequilaterum	VU
Piperaceae	Piper subeburneum	EN
Piperaceae	Piper subsessilifolium	
Piperaceae	Piper tecutlanum	VU
Piperaceae	Piper tenuifolium	VU
Piperaceae	Piper terrabanum	EN
Piperaceae	Piper tuberculatum	
Piperaceae	Piper tuerckheimii	
Piperaceae	Piper udimontanum	EN
Piperaceae	Piper uhdei	
Piperaceae	Piper umbellatum	
Piperaceae	Piper umbricola	VU
Piperaceae	Piper unguiculatum	
Piperaceae	Piper uspantanense	

Piperaceae	Piper usumacintense	EN	
Piperaceae	Piper veraguense		
Piperaceae	Piper vergelense	VU	
Piperaceae	Piper villiramulum		
Piperaceae	Piper virginicum	EN	
Piperaceae	Piper xanthostachyum	VU	
Piperaceae	Piper yousei	CR	YES
Piperaceae	Piper yucatanense		
Piperaceae	Piper yzabalanum		
Plantaginaceae	Angelonia angustifolia		
Plantaginaceae	Antirrhinum majus		
Plantaginaceae	Bacopa lacertosa		
Plantaginaceae	Bacopa monnieri		
Plantaginaceae	Bacopa repens		
Plantaginaceae	Bacopa salzmannii		
Plantaginaceae	Bacopa sessiliflora	EN	
Plantaginaceae	Conobea scoparioides		
Plantaginaceae	Cymbalaria muralis		
Plantaginaceae	Digitalis purpurea		
Plantaginaceae	Gambelia glabrata		
Plantaginaceae	Gratiola oresbia		
Plantaginaceae	Linaria vulgaris	VU	
Plantaginaceae	Lophospermum erubescens		
Plantaginaceae	Lophospermum purpusii		
Plantaginaceae	Lophospermum scandens	VU	
Plantaginaceae	Maurandella antirrhiniflora		
Plantaginaceae	Maurandya scandens		
Plantaginaceae	Mecardonia procumbens		
Plantaginaceae	Penstemon barbatus		
Plantaginaceae	Penstemon campanulatus		
Plantaginaceae	Penstemon gentianoides		
Plantaginaceae	Penstemon hartwegii		
Plantaginaceae	Penstemon hidalgensis		
Plantaginaceae	Penstemon isophyllus		

Plantaginaceae	Penstemon miniatus subsp. apateticus	
Plantaginaceae	Penstemon perfoliatus	VU
Plantaginaceae	Penstemon roseus	
Plantaginaceae	Plantago alismatifolia	
Plantaginaceae	Plantago australis	
Plantaginaceae	Plantago australis subsp. hirtella	
Plantaginaceae	Plantago floccosa	
Plantaginaceae	Plantago lanceolata	
Plantaginaceae	Plantago linearis	
Plantaginaceae	Plantago major	
Plantaginaceae	Plantago nivea	
Plantaginaceae	Plantago pachyneura	
Plantaginaceae	Plantago virginica	
Plantaginaceae	Rhodochiton atrosanguineus	
Plantaginaceae	Russelia acuminata	VU
Plantaginaceae	Russelia campechiana	
Plantaginaceae	Russelia chiapensis	
Plantaginaceae	Russelia coccinea	
Plantaginaceae	Russelia contrerasii	VU
Plantaginaceae	Russelia cuneata	
Plantaginaceae	Russelia equisetiformis	
Plantaginaceae	Russelia floribunda	
Plantaginaceae	Russelia maculosa	VU
Plantaginaceae	Russelia multiflora	
Plantaginaceae	Russelia obtusata	
Plantaginaceae	Russelia polyedra	
Plantaginaceae	Russelia purpusii	
Plantaginaceae	Russelia retrorsa	
Plantaginaceae	Russelia sarmentosa	
Plantaginaceae	Russelia syringifolia	
Plantaginaceae	Russelia tenuis	
Plantaginaceae	Russelia teres	VU
Plantaginaceae	Russelia ternifolia	
Plantaginaceae	Russelia tetraptera	

Plantaginaceae	Russelia verticillata		
Plantaginaceae	Schistophragma mexicanum		
Plantaginaceae	Schistophragma pusillum		
Plantaginaceae	Scoparia dulcis		
Plantaginaceae	Sibthorpia repens		
Plantaginaceae	Stemodia durantifolia		
Plantaginaceae	Stemodia macrantha		
Plantaginaceae	Stemodia peduncularis		
Plantaginaceae	Stemodia schottii		
Plantaginaceae	Stemodia verticillata		
Plantaginaceae	Tetranema roseum		
Plantaginaceae	Uroskinnera almedae	CR	YES
Plantaginaceae	Uroskinnera flavida	VU	
Plantaginaceae	Uroskinnera hirtiflora		
Plantaginaceae	Uroskinnera hirtiflora var. breviloba		
Plantaginaceae	Veronica arvensis		
Plantaginaceae	Veronica peregrina		
Plantaginaceae	Veronica persica		
Platanaceae	Platanus lindeniana		
Platanaceae	Platanus mexicana		
Platanaceae	Platanus occidentalis		
Platanaceae	Platanus rzedowskii		
Plumbaginaceae	Plumbago auriculata		
Plumbaginaceae	Plumbago pulchella		
Plumbaginaceae	Plumbago zeylanica		
Poaceae	Aakia tuerckheimii		
Poaceae	Acroceras zizanioides		
Poaceae	Agrostis aucklandica	VU	
Poaceae	Agrostis bourgaei		
Poaceae	Agrostis ghiesbreghtii		
Poaceae	Agrostis laxissima	EN	
Poaceae	Agrostis microphylla	EN	
Poaceae	Agrostis perennans		
Poaceae	Agrostis subpatens		

Poaceae	Agrostis tolucensis	
Poaceae	Alloteropsis cimicina	
Poaceae	Andropogon bicornis	
Poaceae	Andropogon bourgaei	VU
Poaceae	Andropogon gayanus	
Poaceae	Andropogon glomeratus	
Poaceae	Andropogon gyrans	VU
Poaceae	Andropogon leucostachyus	
Poaceae	Andropogon liebmannii	
Poaceae	Andropogon virgatus	
Poaceae	Andropogon virginicus	
Poaceae	Anthenantia lanata	
Poaceae	Anthephora hermaphrodita	
Poaceae	Anthoxanthum mexicanum	
Poaceae	Arberella glaberrima	
Poaceae	Aristida adscensionis	
Poaceae	Aristida appressa	
Poaceae	Aristida arizonica	
Poaceae	Aristida capillacea	
Poaceae	Aristida curvifolia	
Poaceae	Aristida divaricata	
Poaceae	Aristida fendleriana	
Poaceae	Aristida gibbosa	
Poaceae	Aristida glauca	
Poaceae	Aristida jorullensis	
Poaceae	Aristida laxa	
Poaceae	Aristida mexicana	VU
Poaceae	Aristida pansa	
Poaceae	Aristida purpurascens	EN
Poaceae	Aristida purpurea	
Poaceae	Aristida schiedeana	
Poaceae	Aristida tenuifolia	EN
Poaceae	Aristida ternipes	
Poaceae	Aristida wrightii	

Poaceae	Arthraxon hispidus		
Poaceae	Arthrostylidium excelsum		
Poaceae	Arundinella berteroniana		
Poaceae	Arundinella deppeana		
Poaceae	Arundinella hispida		
Poaceae	Arundo donax		
Poaceae	Aulonemia laxa		
Poaceae	Avena fatua		
Poaceae	Avena sativa		
Poaceae	Axonopus aureus	VU	
Poaceae	Axonopus centralis		
Poaceae	Axonopus compressus		
Poaceae	Axonopus fissifolius		
Poaceae	Axonopus poiophyllus		
Poaceae	Axonopus purpusii		
Poaceae	Bambusa amplexicaulis	CR	YES
Poaceae	Bambusa oldhamii		
Poaceae	Bambusa vulgaris		
Poaceae	Bothriochloa alta		
Poaceae	Bothriochloa barbinodis		
Poaceae	Bothriochloa hirtifolia		
Poaceae	Bothriochloa ischaemum		
Poaceae	Bothriochloa laguroides		
Poaceae	Bothriochloa longipaniculata		
Poaceae	Bothriochloa pertusa		
Poaceae	Bothriochloa saccharoides		
Poaceae	Bothriochloa torreyana		
Poaceae	Bouteloua alamosana		
Poaceae	Bouteloua barbata		
Poaceae	Bouteloua chondrosioides		
Poaceae	Bouteloua curtipendula		
Poaceae	Bouteloua curtipendula var. caespitosa		
Poaceae	Bouteloua curtipendula var. curtipendula		
Poaceae	Bouteloua curtipendula var. tenuis		

Poaceae	Bouteloua dactyloides
Poaceae	Bouteloua dimorpha
Poaceae	Bouteloua distans
Poaceae	Bouteloua diversispicula
Poaceae	Bouteloua elata
Poaceae	Bouteloua gracilis
Poaceae	Bouteloua hirsuta
Poaceae	Bouteloua media
Poaceae	Bouteloua parryi
Poaceae	Bouteloua pedicellata
Poaceae	Bouteloua purpurea
Poaceae	Bouteloua radicosa
Poaceae	Bouteloua reederorum
Poaceae	Bouteloua repens
Poaceae	Bouteloua scabra
Poaceae	Bouteloua scorpioides
Poaceae	Bouteloua simplex
Poaceae	Bouteloua triaena
Poaceae	Bouteloua trifida
Poaceae	Bouteloua uniflora
Poaceae	Bouteloua varia
Poaceae	Brachypodium mexicanum
Poaceae	Briza minor
Poaceae	Bromus anomalus
Poaceae	Bromus attenuatus
Poaceae	Bromus carinatus
Poaceae	Bromus diandrus
Poaceae	Bromus dolichocarpus
Poaceae	Bromus exaltatus
Poaceae	Bromus marginatus
Poaceae	Cenchrus americanus
Poaceae	Cenchrus brownii
Poaceae	Cenchrus ciliaris
Poaceae	Cenchrus clandestinus

Poaceae	Cenchrus complanatus	
Poaceae	Cenchrus distachyus	
Poaceae	Cenchrus echinatus	
Poaceae	Cenchrus michoacanus	
Poaceae	Cenchrus multiflorus	
Poaceae	Cenchrus nervosus	
Poaceae	Cenchrus pilosus	
Poaceae	Cenchrus preslii	
Poaceae	Cenchrus prolificus	
Poaceae	Cenchrus purpureus	
Poaceae	Cenchrus setaceus	
Poaceae	Cenchrus setosus	
Poaceae	Cenchrus spinifex	
Poaceae	Chaetium bromoides	
Poaceae	Chascolytrum subaristatum	
Poaceae	Chloris × subdolichostachya	
Poaceae	Chloris barbata	
Poaceae	Chloris gayana	
Poaceae	Chloris rufescens	
Poaceae	Chloris submutica	
Poaceae	Chloris virgata	
Poaceae	Chusquea circinata	
Poaceae	Chusquea coronalis	VU
Poaceae	Chusquea foliosa	VU
Poaceae	Chusquea glauca	
Poaceae	Chusquea lanceolata	
Poaceae	Chusquea liebmannii	
Poaceae	Chusquea longifolia	EN
Poaceae	Chusquea mulleri	
Poaceae	Chusquea nelsonii	
Poaceae	Chusquea repens	
Poaceae	Chusquea serrulata	EN
Poaceae	Chusquea simpliciflora	VU
Poaceae	Coelorachis ramosa	

Poaceae	Coix lacryma-jobi		
Poaceae	Coleataenia rigidula		
Poaceae	Cortaderia selloana		
Poaceae	Cryptochloa strictiflora		
Poaceae	Cryptochloa variana	CR	
Poaceae	Cymbopogon citratus		
Poaceae	Cynodon dactylon		
Poaceae	Cynodon nlemfuensis		
Poaceae	Cynodon plectostachyus		
Poaceae	Dactylis glomerata		
Poaceae	Dactyloctenium aegyptium		
Poaceae	Deschampsia elongata		
Poaceae	Deschampsia liebmanniana		
Poaceae	Dichanthelium aciculare		
Poaceae	Dichanthelium acuminatum		
Poaceae	Dichanthelium arenicoloides	EN	
Poaceae	Dichanthelium boreale	CR	YES
Poaceae	Dichanthelium boscii	EN	
Poaceae	Dichanthelium columbianum	EN	
Poaceae	Dichanthelium commutatum		
Poaceae	Dichanthelium dichotomum		
Poaceae	Dichanthelium laxiflorum		
Poaceae	Dichanthelium leucothrix	EN	
Poaceae	Dichanthelium oligosanthes		
Poaceae	Dichanthelium ovale	EN	
Poaceae	Dichanthelium sphaerocarpon		
Poaceae	Dichanthelium strigosum		
Poaceae	Dichanthelium tenue		
Poaceae	Dichanthelium transiens		
Poaceae	Dichanthelium villosissimum		
Poaceae	Dichanthelium viscidellum		
Poaceae	Dichanthium annulatum		
Poaceae	Dichanthium aristatum		
Poaceae	Diectomis fastigiata		

Poaceae	Digitaria argillacea		
Poaceae	Digitaria badia		
Poaceae	Digitaria bakeri	VU	
Poaceae	Digitaria bicornis		
Poaceae	Digitaria ciliaris		
Poaceae	Digitaria cognata		
Poaceae	Digitaria curtigluma		
Poaceae	Digitaria eriantha		
Poaceae	Digitaria filiformis		
Poaceae	Digitaria horizontalis		
Poaceae	Digitaria insularis		
Poaceae	Digitaria leucites		
Poaceae	Digitaria nuda		
Poaceae	Digitaria sanguinalis		
Poaceae	Digitaria sellowii		
Poaceae	Digitaria setigera		
Poaceae	Digitaria setigera var. setigera	EN	
Poaceae	Digitaria simpsonii	CR	YES
Poaceae	Digitaria ternata		
Poaceae	Digitaria villosa		
Poaceae	Diplachne fusca subsp. uninervia		
Poaceae	Disakisperma dubium		
Poaceae	Echinochloa crus-galli		
Poaceae	Echinochloa crus-pavonis		
Poaceae	Echinochloa pyramidalis		
Poaceae	Eleusine indica		
Poaceae	Eleusine tristachya		
Poaceae	Elionurus barbiculmis		
Poaceae	Elionurus ciliaris		
Poaceae	Elionurus tripsacoides		
Poaceae	Elymus canadensis		
Poaceae	Elymus longifolius		
Poaceae	Elymus violaceus		
Poaceae	Enneapogon desvauxii		

Poaceae	Eragrostis acutiflora		
Poaceae	Eragrostis atrovirens		
Poaceae	Eragrostis bahiensis		
Poaceae	Eragrostis barrelieri		
Poaceae	Eragrostis cilianensis		
Poaceae	Eragrostis ciliaris		
Poaceae	Eragrostis cumingii	CR	YES
Poaceae	Eragrostis guatemalensis	EN	
Poaceae	Eragrostis hypnoides		
Poaceae	Eragrostis intermedia		
Poaceae	Eragrostis japonica		
Poaceae	Eragrostis lugens		
Poaceae	Eragrostis maypurensis		
Poaceae	Eragrostis mexicana		
Poaceae	Eragrostis pectinacea		
Poaceae	Eragrostis pilosa		
Poaceae	Eragrostis polytricha	VU	
Poaceae	Eragrostis secundiflora		
Poaceae	Eragrostis silveana		
Poaceae	Eragrostis simpliciflora	CR	YES
Poaceae	Eragrostis swallenii		
Poaceae	Eragrostis tenuifolia		
Poaceae	Eragrostis tephrosanthos		
Poaceae	Eragrostis trichocolea		
Poaceae	Eragrostis viscosa		
Poaceae	Eriochloa acuminata		
Poaceae	Eriochloa aristata		
Poaceae	Eriochloa nelsonii		
Poaceae	Eriochloa punctata		
Poaceae	Eriochrysis cayennensis		
Poaceae	Erioneuron avenaceum		
Poaceae	Erioneuron avenaceum var. avenaceum		
Poaceae	Euclasta condylotricha		
Poaceae	Festuca aguana	VU	

Poaceae	Festuca amplissima			
Poaceae	Festuca bromoides			
Poaceae	Festuca hephaestophila			
Poaceae	Festuca myuros			
Poaceae	Festuca orizabensis			
Poaceae	Festuca rosei			
Poaceae	Gigantochloa levis		CR	YES
Poaceae	Gouinia papillosa		EN	
Poaceae	Gouinia virgata			
Poaceae	Guadua aculeata			
Poaceae	Guadua amplexifolia			
Poaceae	Guadua angustifolia			
Poaceae	Guadua longifolia	EN		
Poaceae	Guadua paniculata			
Poaceae	Guadua refracta		VU	
Poaceae	Guadua velutina			
Poaceae	Gynerium sagittatum			
Poaceae	Hackelochloa granularis			
Poaceae	Hemarthria altissima			
Poaceae	Heteropogon contortus			
Poaceae	Heteropogon melanocarpus			
Poaceae	Hilaria cenchroides			
Poaceae	Hilaria ciliata			
Poaceae	Holcus lanatus			
Poaceae	Homolepis aturensis			
Poaceae	Homolepis glutinosa			
Poaceae	Hymenachne amplexicaulis			
Poaceae	Hyparrhenia bracteata			
Poaceae	Hyparrhenia rufa			
Poaceae	Hyperthelia dissoluta			
Poaceae	Ichnanthus nemorosus			
Poaceae	Ichnanthus pallens			
Poaceae	Ichnanthus pallens var. pallens			
Poaceae	Ichnanthus tenuis			

Poaceae	Imperata brasiliensis	
Poaceae	Imperata contracta	
Poaceae	Isachne arundinacea	
Poaceae	Isachne pubescens	
Poaceae	Isachne pulchella	
Poaceae	Ischaemum latifolium	
Poaceae	Ixophorus unisetus	
Poaceae	Jarava ichu	
Poaceae	Jouvea pilosa	
Poaceae	Koeleria macrantha	
Poaceae	Koeleria pyramidata	
Poaceae	Lasiacis divaricata	
Poaceae	Lasiacis divaricata var. divaricata	
Poaceae	Lasiacis divaricata var. leptostachya	
Poaceae	Lasiacis grisebachii	
Poaceae	Lasiacis grisebachii var. grisebachii	
Poaceae	Lasiacis linearis	VU
Poaceae	Lasiacis maculata	
Poaceae	Lasiacis nigra	
Poaceae	Lasiacis oaxacensis	
Poaceae	Lasiacis oaxacensis var. maxonii	
Poaceae	Lasiacis oaxacensis var. oaxacensis	
Poaceae	Lasiacis procerrima	
Poaceae	Lasiacis rhizophora	
Poaceae	Lasiacis rugelii	
Poaceae	Lasiacis ruscifolia	
Poaceae	Lasiacis ruscifolia var. ruscifolia	
Poaceae	Lasiacis ruscifolia var. velutina	EN
Poaceae	Lasiacis scabrior	
Poaceae	Lasiacis sloanei	
Poaceae	Lasiacis standleyi	
Poaceae	Leersia hexandra	
Poaceae	Leersia ligularis	
Poaceae	Leersia ligularis var. breviligulata	

Poaceae	Leersia ligularis var. grandiflora		
Poaceae	Leersia ligularis var. ligularis		
Poaceae	Leersia monandra		
Poaceae	Leptochloa mucronata		
Poaceae	Leptochloa panicea subsp. brachiata		
Poaceae	Leptochloa scabra		
Poaceae	Leptochloa virgata		
Poaceae	Leymus condensatus		
Poaceae	Lithachne pauciflora		
Poaceae	Lolium arundinaceum	VU	
Poaceae	Luziola bahiensis	CR	YES
Poaceae	Megastachya mucronata	CR	YES
Poaceae	Megathyrsus maximus		
Poaceae	Melinis minutiflora		
Poaceae	Melinis repens		
Poaceae	Melinis repens subsp. repens		
Poaceae	Merostachys mexicana	CR	YES
Poaceae	Metcalfia mexicana		
Poaceae	Microchloa kunthii		
Poaceae	Morronea arundinariae		
Poaceae	Morronea cayoensis		
Poaceae	Morronea guatemalensis	EN	
Poaceae	Morronea parviglumis		
Poaceae	Morronea trichidiachnis		
Poaceae	Muhlenbergia breviligula		
Poaceae	Muhlenbergia breviseta		
Poaceae	Muhlenbergia capillaris		
Poaceae	Muhlenbergia cenchroides		
Poaceae	Muhlenbergia ciliata		
Poaceae	Muhlenbergia distichophylla		
Poaceae	Muhlenbergia diversiglumis		
Poaceae	Muhlenbergia dubia		
Poaceae	Muhlenbergia gigantea		
Poaceae	Muhlenbergia implicata		

Poaceae	Muhlenbergia laxa	EN
Poaceae	Muhlenbergia macroura	
Poaceae	Muhlenbergia microsperma	
Poaceae	Muhlenbergia minutissima	
Poaceae	Muhlenbergia mutica	VU
Poaceae	Muhlenbergia nigra	
Poaceae	Muhlenbergia pereilema	
Poaceae	Muhlenbergia peruviana	
Poaceae	Muhlenbergia phleoides	
Poaceae	Muhlenbergia plumbea	
Poaceae	Muhlenbergia plumiseta	
Poaceae	Muhlenbergia pubescens	
Poaceae	Muhlenbergia quadridentata	
Poaceae	Muhlenbergia ramulosa	
Poaceae	Muhlenbergia repens	
Poaceae	Muhlenbergia rigida	
Poaceae	Muhlenbergia robusta	
Poaceae	Muhlenbergia schmitzii	
Poaceae	Muhlenbergia schreberi	
Poaceae	Muhlenbergia setarioides	
Poaceae	Muhlenbergia spiciformis	
Poaceae	Muhlenbergia stricta	
Poaceae	Muhlenbergia tenella	
Poaceae	Muhlenbergia tenuifolia	
Poaceae	Muhlenbergia trifida	
Poaceae	Muhlenbergia uniseta	
Poaceae	Muhlenbergia utilis	
Poaceae	Muhlenbergia vaginata	
Poaceae	Muhlenbergia versicolor	
Poaceae	Muhlenbergia virletii	
Poaceae	Muhlenbergia xanthodas	EN
Poaceae	Nassella caespitosa	
Poaceae	Nassella mucronata	
Poaceae	Nassella neesiana	

Poaceae	Nassella tenuissima			
Poaceae	Ocellochloa biglandularis			
Poaceae	Ocellochloa craterifera			
Poaceae	Ocellochloa pulchella		VU	
Poaceae	Ocellochloa stolonifera			
Poaceae	Oedochloa lanceolata			
Poaceae	Oedochloa standleyi		VU	
Poaceae	Olmeca recta	EN		
Poaceae	Olmeca reflexa	EN		
Poaceae	Olyra latifolia			
Poaceae	Ophiuros exaltatus			
Poaceae	Oplismenus burmanni			
Poaceae	Oplismenus compositus			
Poaceae	Oplismenus hirtellus			
Poaceae	Orthoclada laxa			
Poaceae	Oryza latifolia			
Poaceae	Oryza sativa			
Poaceae	Otatea acuminata			
Poaceae	Otatea fimbriata			
Poaceae	Otatea rzedowskiorum		EN	YES
Poaceae	Panicum alatum			
Poaceae	Panicum bartlettii			
Poaceae	Panicum capillare			
Poaceae	Panicum capillarioides			
Poaceae	Panicum cayennense			
Poaceae	Panicum coloratum		VU	
Poaceae	Panicum dichotomiflorum			
Poaceae	Panicum fluviicola			
Poaceae	Panicum ghiesbreghtii			
Poaceae	Panicum hallii			
Poaceae	Panicum hirsutum			
Poaceae	Panicum hirticaule			
Poaceae	Panicum hispidifolium		EN	
Poaceae	Panicum miliaceum			

Poaceae	Panicum millegrana	
Poaceae	Panicum parcum	
Poaceae	Panicum plenum	
Poaceae	Panicum sellowii	
Poaceae	Panicum sublaeve	VU
Poaceae	Panicum tamaulipense	
Poaceae	Panicum trichanthum	
Poaceae	Panicum trichoides	
Poaceae	Panicum virgatum	
Poaceae	Parodiophyllochloa cordovensis	
Poaceae	Paspalum adoperiens	
Poaceae	Paspalum affine	
Poaceae	Paspalum arsenei	
Poaceae	Paspalum blodgettii	
Poaceae	Paspalum botterii	
Poaceae	Paspalum caespitosum	
Poaceae	Paspalum campylostachyum	
Poaceae	Paspalum candidum	
Poaceae	Paspalum chiapense	EN
Poaceae	Paspalum clavuliferum	
Poaceae	Paspalum conjugatum	
Poaceae	Paspalum conspersum	
Poaceae	Paspalum convexum	
Poaceae	Paspalum costaricense	
Poaceae	Paspalum crassum	
Poaceae	Paspalum cymbiforme	
Poaceae	Paspalum decumbens	VU
Poaceae	Paspalum denticulatum	
Poaceae	Paspalum dilatatum	
Poaceae	Paspalum distichum	
Poaceae	Paspalum hartwegianum	
Poaceae	Paspalum humboldtianum	
Poaceae	Paspalum jaliscanum	
Poaceae	Paspalum langei	

Poaceae	Paspalum lentiginosum	
Poaceae	Paspalum malacophyllum	
Poaceae	Paspalum minus	
Poaceae	Paspalum notatum	
Poaceae	Paspalum orbiculatum	VU
Poaceae	Paspalum paniculatum	
Poaceae	Paspalum pilosum	VU
Poaceae	Paspalum plenum	
Poaceae	Paspalum plicatulum	
Poaceae	Paspalum pubiflorum	
Poaceae	Paspalum repens	
Poaceae	Paspalum scrobiculatum	EN
Poaceae	Paspalum setaceum	
Poaceae	Paspalum setaceum var. setaceum	
Poaceae	Paspalum squamulatum	
Poaceae	Paspalum tinctum	
Poaceae	Paspalum unispicatum	
Poaceae	Paspalum vaginatum	
Poaceae	Paspalum variabile	
Poaceae	Paspalum virgatum	
Poaceae	Peyritschia deyeuxioides	
Poaceae	Peyritschia koelerioides	
Poaceae	Peyritschia pinetorum	EN
Poaceae	Peyritschia pringlei	
Poaceae	Phalaris minor	
Poaceae	Pharus lappulaceus	
Poaceae	Pharus latifolius	
Poaceae	Pharus mezii	
Poaceae	Pharus parvifolius	
Poaceae	Pharus vittatus	CR
Poaceae	Phragmites australis	
Poaceae	Phyllostachys reticulata	VU
Poaceae	Piptochaetium angustifolium	
Poaceae	Piptochaetium fimbriatum	

Poaceae	Piptochaetium panicoides	EN	
Poaceae	Piptochaetium virescens		
Poaceae	Poa annua		
Poaceae	Poa scaberula		
Poaceae	Poa seleri	EN	
Poaceae	Polypogon elongatus		
Poaceae	Polypogon viridis		
Poaceae	Pseudechinolaena polystachya		
Poaceae	Pseudoeriocoma constricta		
Poaceae	Pseudoeriocoma editorum		
Poaceae	Pseudoeriocoma eminens		
Poaceae	Rehia nervata	CR	YES
Poaceae	Rhipidocladum bartlettii		
Poaceae	Rhipidocladum pittieri		
Poaceae	Rhipidocladum racemiflorum		
Poaceae	Rhipidocladum zoqueorum	CR	YES
Poaceae	Rottboellia aurita	VU	
Poaceae	Rottboellia cochinchinensis		
Poaceae	Rugoloa hylaeica		
Poaceae	Rugoloa pilosa		
Poaceae	Rugoloa polygonata		
Poaceae	Saccharum contortum	EN	
Poaceae	Saccharum officinarum		
Poaceae	Saccharum villosum		
Poaceae	Sacciolepis myuros		
Poaceae	Schizachyrium brevifolium		
Poaceae	Schizachyrium condensatum		
Poaceae	Schizachyrium gaumeri	VU	
Poaceae	Schizachyrium gracile	EN	YES
Poaceae	Schizachyrium malacostachyum		
Poaceae	Schizachyrium microstachyum		
Poaceae	Schizachyrium salzmannii		
Poaceae	Schizachyrium sanguineum		
Poaceae	Schizachyrium scoparium		

Poaceae	Schizachyrium tenerum	
Poaceae	Scleropogon brevifolius	
Poaceae	Setaria adhaerens	
Poaceae	Setaria geminata	
Poaceae	Setaria grisebachii	
Poaceae	Setaria leucopila	
Poaceae	Setaria liebmannii	
Poaceae	Setaria longipila	
Poaceae	Setaria macrostachya	
Poaceae	Setaria magna	VU
Poaceae	Setaria palmifolia	
Poaceae	Setaria parviflora	
Poaceae	Setaria poiretiana	
Poaceae	Setaria pumila	
Poaceae	Setaria scandens	
Poaceae	Setaria sulcata	EN
Poaceae	Setaria tenax	
Poaceae	Setaria viridis	
Poaceae	Setariopsis auriculata	
Poaceae	Setariopsis latiglumis	
Poaceae	Sorghastrum brunneum	
Poaceae	Sorghastrum incompletum	
Poaceae	Sorghastrum nutans	
Poaceae	Sorghastrum setosum	
Poaceae	Sorghastrum stipoides	VU
Poaceae	Sorghum bicolor	
Poaceae	Sorghum halepense	
Poaceae	Sorghum trichocladum	
Poaceae	Sporobolus airoides	
Poaceae	Sporobolus atrovirens	
Poaceae	Sporobolus buckleyi	
Poaceae	Sporobolus capillaris	EN
Poaceae	Sporobolus coromandelianus	
Poaceae	Sporobolus erectus	

Poaceae	Sporobolus indicus			
Poaceae	Sporobolus junceus			
Poaceae	Sporobolus macrospermus			
Poaceae	Sporobolus purpurascens			
Poaceae	Sporobolus pyramidalis			
Poaceae	Sporobolus pyramidatus			
Poaceae	Sporobolus spartinae			
Poaceae	Sporobolus tenuissimus			
Poaceae	Sporobolus trichodes			
Poaceae	Sporobolus wrightii			
Poaceae	Stapfochloa ciliata			
Poaceae	Steinchisma hians			
Poaceae	Steinchisma laxum			
Poaceae	Stenotaphrum secundatum			
Poaceae	Streptochaeta sodiroana			VU
Poaceae	Streptochaeta spicata			
Poaceae	Trachypogon spicatus			
Poaceae	Tragus berteronianus			
Poaceae	Tridens texanus			
Poaceae	Triniochloa stipoides			
Poaceae	Tripsacum andersonii			EN
Poaceae	Tripsacum dactyloides			
Poaceae	Tripsacum intermedium		EN	
Poaceae	Tripsacum jalapense			
Poaceae	Tripsacum lanceolatum			
Poaceae	Tripsacum latifolium			
Poaceae	Tripsacum laxum			
Poaceae	Tripsacum maizar	VU	EN	
Poaceae	Tripsacum manisuroides			
Poaceae	Tripsacum peruvianum		EN	VU
Poaceae	Tripsacum pilosum			
Poaceae	Tripsacum zopilotense	NT	EN	
Poaceae	Trisetum irazuense			
Poaceae	Trisetum pringlei			VU

Poaceae	Trisetum spicatum			
Poaceae	Trisetum virletii			
Poaceae	Triticum aestivum			
Poaceae	Uniola pittieri			
Poaceae	Urochloa brizantha			
Poaceae	Urochloa eminii			
Poaceae	Urochloa fusca			
Poaceae	Urochloa mutica			
Poaceae	Urochloa plantaginea			
Poaceae	Urochloa polystachya			EN
Poaceae	Urochloa reptans			
Poaceae	Zeugites americanus			
Poaceae	Zuloagaea bulbosa			
Podocarpaceae	Podocarpus guatemalensis			
Podocarpaceae	Podocarpus matudae	NT	VU	
Podocarpaceae	Podocarpus matudae subsp. matudae			
Podocarpaceae	Podocarpus oleifolius			
Podocarpaceae	Podocarpus oleifolius subsp. costaricensis			EN
Podostemaceae	Marathrum foeniculaceum			
Podostemaceae	Marathrum schiedeanum			
Podostemaceae	Marathrum tenue			
Podostemaceae	Noveloa coulteriana			
Podostemaceae	Podostemum ceratophyllum			VU
Podostemaceae	Podostemum rutifolium subsp. ricciiforme			EN
Podostemaceae	Tristicha trifaria			
Polemoniaceae	Bonplandia geminiflora			
Polemoniaceae	Cobaea pachysepala			
Polemoniaceae	Cobaea paneroi			EN
Polemoniaceae	Cobaea pringlei			
Polemoniaceae	Cobaea scandens			
Polemoniaceae	Cobaea stipularis			
Polemoniaceae	Giliastrum incisum			
Polemoniaceae	Loeselia amplectens			
Polemoniaceae	Loeselia ciliata			

Polemoniaceae	Loeselia coerulea		
Polemoniaceae	Loeselia glandulosa		
Polemoniaceae	Loeselia mexicana		
Polemoniaceae	Loeselia pumila		
Polemoniaceae	Loeselia rupestris	VU	
Polemoniaceae	Phlox paniculata	EN	YES
Polemoniaceae	Polemonium grandiflorum		
Polygalaceae	Asemeia floribunda	VU	
Polygalaceae	Asemeia grandiflora	VU	
Polygalaceae	Asemeia hondurana	VU	
Polygalaceae	Bredemeyera lucida	VU	
Polygalaceae	Hebecarpa barbeyana		
Polygalaceae	Hebecarpa costaricensis		
Polygalaceae	Hebecarpa obscura		
Polygalaceae	Hebecarpa rivinifolia		
Polygalaceae	Hebecarpa tehuacana	VU	
Polygalaceae	Monnina bifurcata	VU	
Polygalaceae	Monnina ciliolata		
Polygalaceae	Monnina euonymoides		
Polygalaceae	Monnina guatemalensis		
Polygalaceae	Monnina schlechtendaliana		
Polygalaceae	Monnina sylvatica		
Polygalaceae	Monnina xalapensis		
Polygalaceae	Polygala adenophora	VU	
Polygalaceae	Polygala alba		
Polygalaceae	Polygala aparinoides		
Polygalaceae	Polygala appendiculata		
Polygalaceae	Polygala berlandieri		
Polygalaceae	Polygala chiapensis	EN	
Polygalaceae	Polygala chinensis	VU	
Polygalaceae	Polygala compacta		
Polygalaceae	Polygala conferta		
Polygalaceae	Polygala cuspidulata		
Polygalaceae	Polygala dolichocarpa		

Polygalaceae	Polygala evolvulacea	EN
Polygalaceae	Polygala galeottii	EN
Polygalaceae	Polygala glochidiata	
Polygalaceae	Polygala gracillima	
Polygalaceae	Polygala jamaicensis	
Polygalaceae	Polygala longicaulis	
Polygalaceae	Polygala major	
Polygalaceae	Polygala mexicana	
Polygalaceae	Polygala microtricha	VU
Polygalaceae	Polygala paniculata	
Polygalaceae	Polygala salviniana	EN
Polygalaceae	Polygala scoparia	
Polygalaceae	Polygala scoparioides	
Polygalaceae	Polygala tenuis	EN
Polygalaceae	Polygala trichosperma	
Polygalaceae	Polygala velata	EN
Polygalaceae	Rhinotropis nitida var. lithophila	VU
Polygalaceae	Rhinotropis purpusii	
Polygalaceae	Securidaca diversifolia	
Polygalaceae	Securidaca sylvestris	
Polygonaceae	Antigonon cinerascens	
Polygonaceae	Antigonon flavescens	
Polygonaceae	Antigonon guatimalense	
Polygonaceae	Antigonon leptopus	
Polygonaceae	Coccoloba acapulcensis	
Polygonaceae	Coccoloba barbadensis	
Polygonaceae	Coccoloba belizensis	
Polygonaceae	Coccoloba caracasana	
Polygonaceae	Coccoloba chiapensis	VU
Polygonaceae	Coccoloba cozumelensis	
Polygonaceae	Coccoloba diversifolia	
Polygonaceae	Coccoloba escuintlensis	
Polygonaceae	Coccoloba floresii	EN
Polygonaceae	Coccoloba floribunda	

Polygonaceae	Coccoloba hirtella		
Polygonaceae	Coccoloba hondurensis		
Polygonaceae	Coccoloba humboldtii		
Polygonaceae	Coccoloba lehmannii		
Polygonaceae	Coccoloba liebmannii		
Polygonaceae	Coccoloba lindaviana	CR	EN
Polygonaceae	Coccoloba lindeniana	CR	VU
Polygonaceae	Coccoloba montana		
Polygonaceae	Coccoloba orizabae		EN
Polygonaceae	Coccoloba spicata		
Polygonaceae	Coccoloba tuerckheimii		
Polygonaceae	Coccoloba tunii		VU
Polygonaceae	Enneatypus ramiflorus		
Polygonaceae	Fagopyrum esculentum		EN
Polygonaceae	Gymnopodium floribundum		
Polygonaceae	Muehlenbeckia platyclada		VU
Polygonaceae	Muehlenbeckia tamnifolia		
Polygonaceae	Persicaria amphibia		
Polygonaceae	Persicaria bungeana		
Polygonaceae	Persicaria capitata		
Polygonaceae	Persicaria chinensis		
Polygonaceae	Persicaria decipiens		
Polygonaceae	Persicaria glabra		
Polygonaceae	Persicaria hispida		EN
Polygonaceae	Persicaria hydropiper		VU
Polygonaceae	Persicaria hydropiperoides		
Polygonaceae	Persicaria lapathifolia		
Polygonaceae	Persicaria punctata		
Polygonaceae	Persicaria segetum		
Polygonaceae	Persicaria stagnina		
Polygonaceae	Peutalis punctata		
Polygonaceae	Podopterus cordifolius		
Polygonaceae	Podopterus mexicanus		
Polygonaceae	Polygonum longiocreatum		VU

Polygonaceae	Rumex acetosella		
Polygonaceae	Rumex conglomeratus		
Polygonaceae	Rumex crispus		
Polygonaceae	Rumex mexicanus		
Polygonaceae	Rumex obtusifolius		
Polygonaceae	Rumex obtusifolius subsp. obtusifolius		VU
Polygonaceae	Rumex pulcher		
Polygonaceae	Ruprechtia chiapensis		
Polygonaceae	Ruprechtia costata		
Polygonaceae	Ruprechtia fusca		
Polygonaceae	Ruprechtia pallida		
Polygonaceae	Triplaris melaenodendron		
Polypodiaceae	Arachniodes denticulata		
Polypodiaceae	Bolbitis aliena		VU
Polypodiaceae	Bolbitis hastata		
Polypodiaceae	Bolbitis portoricensis		
Polypodiaceae	Bolbitis serratifolia		EN
Polypodiaceae	Bolbitis umbrosa		EN
Polypodiaceae	Campyloneurum amphostenon		
Polypodiaceae	Campyloneurum angustifolium		
Polypodiaceae	Campyloneurum crassifolium		
Polypodiaceae	Campyloneurum ensifolium		
Polypodiaceae	Campyloneurum phyllitidis	VU	
Polypodiaceae	Campyloneurum repens		
Polypodiaceae	Campyloneurum serpentinum		
Polypodiaceae	Campyloneurum tenuipes		
Polypodiaceae	Campyloneurum xalapense		
Polypodiaceae	Ctenitis bullata		EN
Polypodiaceae	Ctenitis equestris		
Polypodiaceae	Ctenitis excelsa		
Polypodiaceae	Ctenitis hemsleyana		
Polypodiaceae	Ctenitis interjecta		VU
Polypodiaceae	Ctenitis melanosticta		
Polypodiaceae	Ctenitis mexicana		EN

Polypodiaceae	Ctenitis microchlaena	EN	YES
Polypodiaceae	Ctenitis nigrovenia		
Polypodiaceae	Ctenitis salvinii	VU	
Polypodiaceae	Ctenitis sloanei	EN	
Polypodiaceae	Ctenitis strigilosa	EN	
Polypodiaceae	Ctenitis submarginalis	VU	
Polypodiaceae	Ctenitis thelypteroides	CR	YES
Polypodiaceae	Cyclopeltis semicordata		
Polypodiaceae	Didymochlaena truncatula		
Polypodiaceae	Dryopteris cinnamomea		
Polypodiaceae	Dryopteris filix-mas		
Polypodiaceae	Dryopteris futura	VU	
Polypodiaceae	Dryopteris maxonii		
Polypodiaceae	Dryopteris nubigena	EN	
Polypodiaceae	Dryopteris patula		
Polypodiaceae	Dryopteris wallichiana		
Polypodiaceae	Elaphoglossum apodum	CR	YES
Polypodiaceae	Elaphoglossum auricomum	VU	
Polypodiaceae	Elaphoglossum davidsei		
Polypodiaceae	Elaphoglossum decursivum	EN	
Polypodiaceae	Elaphoglossum ellipticifolium	EN	
Polypodiaceae	Elaphoglossum erinaceum		
Polypodiaceae	Elaphoglossum glaucum		
Polypodiaceae	Elaphoglossum gratum		
Polypodiaceae	Elaphoglossum guatemalense		
Polypodiaceae	Elaphoglossum latifolium		
Polypodiaceae	Elaphoglossum leebrowniae	EN	
Polypodiaceae	Elaphoglossum lindenii		
Polypodiaceae	Elaphoglossum lonchophyllum		
Polypodiaceae	Elaphoglossum mcvaughii	EN	
Polypodiaceae	Elaphoglossum mesoamericanum	VU	
Polypodiaceae	Elaphoglossum minutum		
Polypodiaceae	Elaphoglossum muscosum		
Polypodiaceae	Elaphoglossum obovatum	CR	YES

Polypodiaceae	Elaphoglossum obscurum	VU	
Polypodiaceae	Elaphoglossum paleaceum		
Polypodiaceae	Elaphoglossum pallidum	EN	
Polypodiaceae	Elaphoglossum parduei	EN	
Polypodiaceae	Elaphoglossum peltatum		
Polypodiaceae	Elaphoglossum petiolatum		
Polypodiaceae	Elaphoglossum piloselloides		
Polypodiaceae	Elaphoglossum pilosius	EN	
Polypodiaceae	Elaphoglossum pusillum	EN	
Polypodiaceae	Elaphoglossum revolutum		
Polypodiaceae	Elaphoglossum rubescens	EN	
Polypodiaceae	Elaphoglossum sartorii		
Polypodiaceae	Elaphoglossum setigerum		
Polypodiaceae	Elaphoglossum setosum	VU	
Polypodiaceae	Elaphoglossum squamipes		
Polypodiaceae	Elaphoglossum tambillense	VU	
Polypodiaceae	Elaphoglossum tectum	VU	
Polypodiaceae	Elaphoglossum tenuifolium	VU	
Polypodiaceae	Elaphoglossum vestitum		
Polypodiaceae	Elaphoglossum viride	VU	
Polypodiaceae	Grammitis anfractuosa	VU	
Polypodiaceae	Grammitis apiculata	EN	
Polypodiaceae	Grammitis basiattenuata	VU	
Polypodiaceae	Grammitis cultrata		
Polypodiaceae	Grammitis delicatula	VU	
Polypodiaceae	Grammitis ecostata	EN	
Polypodiaceae	Grammitis elastica	EN	
Polypodiaceae	Grammitis firma	EN	
Polypodiaceae	Grammitis leptostoma		
Polypodiaceae	Grammitis limula	CR	YES
Polypodiaceae	Grammitis linearifolia		
Polypodiaceae	Grammitis mitchelliae	VU	
Polypodiaceae	Grammitis moniliformis		
Polypodiaceae	Grammitis pilosissima		

Polypodiaceae	Grammitis prionodes			
Polypodiaceae	Grammitis rostrata			
Polypodiaceae	Grammitis serrulata			
Polypodiaceae	Grammitis taenifolia		VU	
Polypodiaceae	Grammitis trichomanoides		CR	YES
Polypodiaceae	Grammitis xiphopteroides			
Polypodiaceae	Lepisorus longifolius		EN	
Polypodiaceae	Lomariopsis maxonii		CR	YES
Polypodiaceae	Lomariopsis mexicana		VU	
Polypodiaceae	Lomariopsis recurvata			
Polypodiaceae	Lomariopsis vestita		EN	
Polypodiaceae	Loxogramme mexicana			
Polypodiaceae	Megalastrum galeottii			
Polypodiaceae	Megalastrum gompholepis		EN	
Polypodiaceae	Megalastrum lunense		VU	
Polypodiaceae	Megalastrum pulverulentum			
Polypodiaceae	Megalastrum sparsipilosum		VU	
Polypodiaceae	Megalastrum subincisum			
Polypodiaceae	Mickelia bernoullii			
Polypodiaceae	Mickelia hemiotis		VU	
Polypodiaceae	Mickelia pergamentacea			
Polypodiaceae	Microgramma brunei		EN	
Polypodiaceae	Microgramma lycopodioides			
Polypodiaceae	Microgramma nitida			
Polypodiaceae	Microgramma percussa			
Polypodiaceae	Microgramma reptans		EN	
Polypodiaceae	Microsorum sarawakense			
Polypodiaceae	Neocheiropteris ensata		EN	
Polypodiaceae	Nephrolepis biserrata			
Polypodiaceae	Nephrolepis brownii			
Polypodiaceae	Nephrolepis cordifolia	EN		
Polypodiaceae	Nephrolepis exaltata			
Polypodiaceae	Nephrolepis hirsutula			
Polypodiaceae	Nephrolepis obtusiloba		EN	YES

Polypodiaceae	Nephrolepis pectinata		
Polypodiaceae	Nephrolepis pendula		
Polypodiaceae	Nephrolepis rivularis	VU	
Polypodiaceae	Nephrolepis undulata		
Polypodiaceae	Olfersia cervina	VU	
Polypodiaceae	Parapolystichum effusum		
Polypodiaceae	Parapolystichum effusum var. divergens		
Polypodiaceae	Parapolystichum effusum var. effusum		
Polypodiaceae	Pecluma alfredii		
Polypodiaceae	Pecluma atra		
Polypodiaceae	Pecluma bourgeauana	EN	
Polypodiaceae	Pecluma consimilis	VU	
Polypodiaceae	Pecluma dispersa		
Polypodiaceae	Pecluma divaricata		
Polypodiaceae	Pecluma dulcis	VU	
Polypodiaceae	Pecluma ferruginea		
Polypodiaceae	Pecluma hartwegiana		
Polypodiaceae	Pecluma hygrometrica	VU	
Polypodiaceae	Pecluma longepinnulata		
Polypodiaceae	Pecluma paradiseae	CR	YES
Polypodiaceae	Pecluma pectinata	EN	
Polypodiaceae	Pecluma plumula		
Polypodiaceae	Pecluma rhachipterygia		
Polypodiaceae	Pecluma sursumcurrens		
Polypodiaceae	Phanerophlebia gastonyi	VU	
Polypodiaceae	Phanerophlebia juglandifolia		
Polypodiaceae	Phanerophlebia macrosora		
Polypodiaceae	Phanerophlebia nobilis		
Polypodiaceae	Phanerophlebia remotispora		
Polypodiaceae	Phanerophlebia umbonata		
Polypodiaceae	Phlebodium areolatum		
Polypodiaceae	Phlebodium aureum		
Polypodiaceae	Phlebodium decumanum		
Polypodiaceae	Pleopeltis × sordidula	EN	

Polypodiaceae	Pleopeltis angusta	
Polypodiaceae	Pleopeltis astrolepis	
Polypodiaceae	Pleopeltis bradeorum	
Polypodiaceae	Pleopeltis collinsii	
Polypodiaceae	Pleopeltis conzattii	
Polypodiaceae	Pleopeltis crassinervata	
Polypodiaceae	Pleopeltis cryptocarpos	
Polypodiaceae	Pleopeltis fallax	
Polypodiaceae	Pleopeltis furfuracea	
Polypodiaceae	Pleopeltis guttata	
Polypodiaceae	Pleopeltis lepidotricha	
Polypodiaceae	Pleopeltis lindeniana	
Polypodiaceae	Pleopeltis macrocarpa	
Polypodiaceae	Pleopeltis madrensis	
Polypodiaceae	Pleopeltis mexicana	
Polypodiaceae	Pleopeltis michauxiana	
Polypodiaceae	Pleopeltis montigena	
Polypodiaceae	Pleopeltis platylepis	
Polypodiaceae	Pleopeltis plebeia	
Polypodiaceae	Pleopeltis polylepis	
Polypodiaceae	Pleopeltis polylepis var. interjecta	
Polypodiaceae	Pleopeltis polypodioides	
Polypodiaceae	Pleopeltis pyrrholepis	
Polypodiaceae	Pleopeltis rosei	
Polypodiaceae	Pleopeltis sanctae-rosae	
Polypodiaceae	Pleopeltis thyssanolepis	
Polypodiaceae	Pleopeltis villagranii	VU
Polypodiaceae	Polybotrya caudata	VU
Polypodiaceae	Polybotrya polybotryoides	VU
Polypodiaceae	Polypodium arcanum	
Polypodiaceae	Polypodium chiapense	EN
Polypodiaceae	Polypodium colpodes	
Polypodiaceae	Polypodium conterminans	
Polypodiaceae	Polypodium eatonii	

Polypodiaceae	Polypodium echinolepis		
Polypodiaceae	Polypodium eperopeutes	EN	
Polypodiaceae	Polypodium fissidens	VU	
Polypodiaceae	Polypodium fraternum		
Polypodiaceae	Polypodium hispidulum		
Polypodiaceae	Polypodium martensii		
Polypodiaceae	Polypodium plesiosorum		
Polypodiaceae	Polypodium pleurosorum		
Polypodiaceae	Polypodium puberulum		
Polypodiaceae	Polypodium rhodopleuron		
Polypodiaceae	Polypodium subpetiolatum		
Polypodiaceae	Polystichum aculeatum	EN	
Polypodiaceae	Polystichum distans		
Polypodiaceae	Polystichum erythrosorum		
Polypodiaceae	Polystichum fournieri		
Polypodiaceae	Polystichum muricatum		
Polypodiaceae	Polystichum ordinatum		
Polypodiaceae	Polystichum platyphyllum	VU	
Polypodiaceae	Polystichum speciosissimum		
Polypodiaceae	Pyrrosia longifolia		
Polypodiaceae	Pyrrosia tricholepis	CR	YES
Polypodiaceae	Selliguea enervis	EN	
Polypodiaceae	Serpocaulon attenuatum		
Polypodiaceae	Serpocaulon dissimile	EN	
Polypodiaceae	Serpocaulon falcaria		
Polypodiaceae	Serpocaulon fraxinifolium	VU	
Polypodiaceae	Serpocaulon loriceum		
Polypodiaceae	Serpocaulon sessilifolium	VU	
Polypodiaceae	Serpocaulon triseriale		
Polypodiaceae	Stigmatopteris longicaudata		
Polypodiaceae	Stigmatopteris sordida	VU	
Polypodiaceae	Tectaria cicutaria	EN	
Polypodiaceae	Tectaria heracleifolia		
Polypodiaceae	Tectaria incisa		

Polypodiaceae	Tectaria incisa f. vivipara		EN
Polypodiaceae	Tectaria mexicana		
Polypodiaceae	Tectaria panamensis		
Polypodiaceae	Tectaria transiens		
Pontederiaceae	Heteranthera dubia		
Pontederiaceae	Heteranthera limosa		
Pontederiaceae	Heteranthera peduncularis		
Pontederiaceae	Heteranthera reniformis		
Pontederiaceae	Heteranthera rotundifolia		
Pontederiaceae	Heteranthera seubertiana	EN	EN
Pontederiaceae	Pontederia cordata		
Pontederiaceae	Pontederia crassipes		
Pontederiaceae	Pontederia sagittata		
Portulacaceae	Portulaca grandiflora		
Portulacaceae	Portulaca granulatostellulata		VU
Portulacaceae	Portulaca mexicana		
Portulacaceae	Portulaca oleracea		
Portulacaceae	Portulaca pilosa		
Portulacaceae	Portulaca rubricaulis		
Portulacaceae	Portulaca umbraticola		
Potamogetonaceae	Potamogeton foliosus		
Potamogetonaceae	Potamogeton illinoensis		
Potamogetonaceae	Potamogeton nodosus		
Potamogetonaceae	Potamogeton pusillus		
Potamogetonaceae	Stuckenia pectinata		
Primulaceae	Ardisia bracteosa		
Primulaceae	Ardisia breedlovei		VU
Primulaceae	Ardisia chiapensis		EN
Primulaceae	Ardisia compressa		
Primulaceae	Ardisia escallonioides		
Primulaceae	Ardisia fendleri		
Primulaceae	Ardisia hintonii		
Primulaceae	Ardisia hyalina		VU
Primulaceae	Ardisia liebmannii		

Primulaceae	Ardisia liebmannii subsp. jalapensis		VU	
Primulaceae	Ardisia mexicana subsp. siltepecana			
Primulaceae	Ardisia nigrescens			
Primulaceae	Ardisia paschalis			
Primulaceae	Ardisia pellucida			
Primulaceae	Ardisia rarescens			
Primulaceae	Ardisia revoluta			
Primulaceae	Ardisia staminosa			
Primulaceae	Ardisia tacanensis		VU	
Primulaceae	Ardisia thyrsiflora			
Primulaceae	Ardisia tuerckheimii			
Primulaceae	Ardisia venosa			
Primulaceae	Ardisia venosissima			
Primulaceae	Ardisia verapazensis			
Primulaceae	Ardisia verapazensis subsp. cucullata			
Primulaceae	Ardisia verdisepala	EN	EN	YES
Primulaceae	Bonellia macrocarpa			
Primulaceae	Bonellia macrocarpa subsp. pungens			
Primulaceae	Ctenardisia ovandensis	EN	VU	
Primulaceae	Deherainia matudae	VU		
Primulaceae	Deherainia smaragdina			
Primulaceae	Deherainia smaragdina subsp. occidentalis		VU	
Primulaceae	Jacquinia armillaris		CR	
Primulaceae	Lysimachia arvensis			
Primulaceae	Lysimachia arvensis var. caerulea			
Primulaceae	Lysimachia congestiflora		EN	
Primulaceae	Myrsine coriacea			
Primulaceae	Myrsine cubana			
Primulaceae	Myrsine floridana		VU	
Primulaceae	Myrsine guianensis			
Primulaceae	Myrsine juergensenii			
Primulaceae	Myrsine pellucidopunctata		EN	
Primulaceae	Myrsine penduliflora			
Primulaceae	Parathesis belizensis			

Primulaceae	Parathesis bracteolata	EN	
Primulaceae	Parathesis breedlovei	VU	
Primulaceae	Parathesis calophylla	EN	
Primulaceae	Parathesis chiapensis		
Primulaceae	Parathesis cintalapana	EN	
Primulaceae	Parathesis columnaris		
Primulaceae	Parathesis cubana		
Primulaceae	Parathesis donnell-smithii		
Primulaceae	Parathesis gracilis	EN	
Primulaceae	Parathesis hondurensis	VU	
Primulaceae	Parathesis lanceolata		
Primulaceae	Parathesis latifolia	CR	YES
Primulaceae	Parathesis lenticellata		
Primulaceae	Parathesis leptopa		
Primulaceae	Parathesis macronema		
Primulaceae	Parathesis melanosticta		
Primulaceae	Parathesis mexicana	CR	
Primulaceae	Parathesis microcalyx	CR	
Primulaceae	Parathesis minutiflora		
Primulaceae	Parathesis navarretei	CR	YES
Primulaceae	Parathesis neei	VU	
Primulaceae	Parathesis oerstediana		
Primulaceae	Parathesis papillosa	EN	
Primulaceae	Parathesis parvissima	VU	
Primulaceae	Parathesis psychotrioides		
Primulaceae	Parathesis reflexa	VU	
Primulaceae	Parathesis rekoi	VU	
Primulaceae	Parathesis rubriflora	VU	
Primulaceae	Parathesis rufa	VU	
Primulaceae	Parathesis serrulata		
Primulaceae	Parathesis sessilifolia		
Primulaceae	Parathesis subulata		
Primulaceae	Parathesis tenuis		
Primulaceae	Parathesis trichogyne	EN	

Primulaceae	Parathesis villalobosii			EN	
Primulaceae	Parathesis villosa				
Primulaceae	Samolus ebracteatus				
Primulaceae	Samolus parviflorus				
Primulaceae	Stylogyne turbacensis			VU	
Primulaceae	Stylogyne turbacensis subsp. laevis				
Proteaceae	Grevillea robusta				
Proteaceae	Macadamia ternifolia		EN	EN	
Proteaceae	Roupala mexicana		EN	EN	
Proteaceae	Roupala montana				
Psilotaceae	Psilotum complanatum	VU			
Psilotaceae	Psilotum nudum				
Pteridaceae	Acrostichum aureum				
Pteridaceae	Acrostichum danaeifolium				
Pteridaceae	Adiantopsis seemannii			VU	
Pteridaceae	Adiantum alan-smithii			CR	YES
Pteridaceae	Adiantum amplum				
Pteridaceae	Adiantum andicola				
Pteridaceae	Adiantum braunii				
Pteridaceae	Adiantum capillus-veneris				
Pteridaceae	Adiantum caryotideum			CR	YES
Pteridaceae	Adiantum concinnum				
Pteridaceae	Adiantum dolosum			EN	YES
Pteridaceae	Adiantum feei				
Pteridaceae	Adiantum fructuosum			VU	
Pteridaceae	Adiantum latifolium				
Pteridaceae	Adiantum lunulatum				
Pteridaceae	Adiantum macrophyllum				
Pteridaceae	Adiantum oaxacanum			VU	
Pteridaceae	Adiantum obliquum				
Pteridaceae	Adiantum petiolatum				
Pteridaceae	Adiantum poiretii				
Pteridaceae	Adiantum pulverulentum				
Pteridaceae	Adiantum raddianum				

Pteridaceae	Adiantum seemannii	EN	
Pteridaceae	Adiantum tenerum		
Pteridaceae	Adiantum tetraphyllum		
Pteridaceae	Adiantum trapeziforme		
Pteridaceae	Adiantum trichochlaenum	VU	
Pteridaceae	Adiantum tricholepis		
Pteridaceae	Adiantum villosum		
Pteridaceae	Adiantum wilesianum		
Pteridaceae	Adiantum wilsonii	VU	
Pteridaceae	Ananthacorus angustifolius		
Pteridaceae	Anogramma leptophylla		
Pteridaceae	Hecistopteris pumila	CR	YES
Pteridaceae	Hemionitis achariorum	EN	
Pteridaceae	Hemionitis aemula		
Pteridaceae	Hemionitis angustifolia		
Pteridaceae	Hemionitis atropurpurea		
Pteridaceae	Hemionitis beitelii		
Pteridaceae	Hemionitis bonariensis		
Pteridaceae	Hemionitis bradburyi		
Pteridaceae	Hemionitis cairon		
Pteridaceae	Hemionitis candida		
Pteridaceae	Hemionitis cartilaginea		
Pteridaceae	Hemionitis complanata	CR	YES
Pteridaceae	Hemionitis crassifolia		
Pteridaceae	Hemionitis cucullans		
Pteridaceae	Hemionitis decomposita		
Pteridaceae	Hemionitis doryopteris	EN	
Pteridaceae	Hemionitis eatonii		
Pteridaceae	Hemionitis ehrenbergiana		
Pteridaceae	Hemionitis emperatricella		
Pteridaceae	Hemionitis farinosa		
Pteridaceae	Hemionitis formosa		
Pteridaceae	Hemionitis galeottii		
Pteridaceae	Hemionitis hirsuta		

Pteridaceae	Hemionitis horridula	
Pteridaceae	Hemionitis incana	
Pteridaceae	Hemionitis integerrima	
Pteridaceae	Hemionitis intramarginalis	
Pteridaceae	Hemionitis kaulfussii	
Pteridaceae	Hemionitis lendigera	
Pteridaceae	Hemionitis levyi	VU
Pteridaceae	Hemionitis lozanoi	
Pteridaceae	Hemionitis marginata	
Pteridaceae	Hemionitis meifolia	
Pteridaceae	Hemionitis membranacea	VU
Pteridaceae	Hemionitis myriophylla	
Pteridaceae	Hemionitis notholaenoides	
Pteridaceae	Hemionitis nudiuscula	
Pteridaceae	Hemionitis ovata	
Pteridaceae	Hemionitis palmata	
Pteridaceae	Hemionitis pedata	
Pteridaceae	Hemionitis persica	
Pteridaceae	Hemionitis pteridioides	
Pteridaceae	Hemionitis purpusii	EN
Pteridaceae	Hemionitis radiata	
Pteridaceae	Hemionitis rufa	EN
Pteridaceae	Hemionitis sinuata	
Pteridaceae	Hemionitis sulphurea	
Pteridaceae	Hemionitis ternifolia	
Pteridaceae	Hemionitis urglae	VU
Pteridaceae	Hemionitis villosa	
Pteridaceae	Hemionitis vooshvazool	
Pteridaceae	Jamesonia flexuosa	VU
Pteridaceae	Llavea cordifolia	
Pteridaceae	Pellaea cordifolia	
Pteridaceae	Pityrogramma calomelanos	
Pteridaceae	Pityrogramma dealbata	
Pteridaceae	Pityrogramma ebenea	

Pteridaceae	Pityrogramma pearcei	EN	
Pteridaceae	Pityrogramma trifoliata		
Pteridaceae	Polytaenium cajenense	EN	
Pteridaceae	Polytaenium chlorosporum	EN	
Pteridaceae	Polytaenium feei		
Pteridaceae	Polytaenium guayanense	EN	YES
Pteridaceae	Polytaenium jenmanii	CR	YES
Pteridaceae	Polytaenium lineatum		
Pteridaceae	Pteris altissima		
Pteridaceae	Pteris biaurita		
Pteridaceae	Pteris chiapensis	VU	
Pteridaceae	Pteris cretica		
Pteridaceae	Pteris fallax		
Pteridaceae	Pteris grandifolia		
Pteridaceae	Pteris longifolia		
Pteridaceae	Pteris muricata	VU	
Pteridaceae	Pteris muricella		
Pteridaceae	Pteris orizabae		
Pteridaceae	Pteris plumieri	EN	
Pteridaceae	Pteris podophylla		
Pteridaceae	Pteris propinqua	VU	
Pteridaceae	Pteris pulchra		
Pteridaceae	Pteris pungens		
Pteridaceae	Pteris quadriaurita		
Pteridaceae	Pteris vittata		
Pteridaceae	Pterozonium brevifrons	VU	
Pteridaceae	Radiovittaria stipitata	EN	
Pteridaceae	Scoliosorus ensiformis		
Pteridaceae	Vittaria bradeorum	EN	
Pteridaceae	Vittaria flavicosta		
Pteridaceae	Vittaria graminifolia		
Pteridaceae	Vittaria lineata		
Putranjivaceae	Drypetes brownii		
Putranjivaceae	Drypetes gentryi		

Putranjivaceae	Drypetes lateriflora		
Ranunculaceae	Anemone berlandieri		
Ranunculaceae	Clematis acapulcensis		
Ranunculaceae	Clematis affinis		EN
Ranunculaceae	Clematis caleoides		
Ranunculaceae	Clematis dioica		
Ranunculaceae	Clematis drummondii		
Ranunculaceae	Clematis grahamii		
Ranunculaceae	Clematis grossa		
Ranunculaceae	Clematis haenkeana		
Ranunculaceae	Clematis pitcheri		
Ranunculaceae	Clematis polygama		
Ranunculaceae	Clematis rhodocarpa		
Ranunculaceae	Delphinium bicornutum		
Ranunculaceae	Delphinium bicornutum subsp. oaxacanum		VU
Ranunculaceae	Delphinium pedatisectum		
Ranunculaceae	Knowltonia mexicana		
Ranunculaceae	Ranunculus dichotomus		
Ranunculaceae	Ranunculus gouanii		
Ranunculaceae	Ranunculus macranthus		
Ranunculaceae	Ranunculus ophioglossifolius		
Ranunculaceae	Ranunculus petiolaris		
Ranunculaceae	Ranunculus praemorsus		
Ranunculaceae	Ranunculus sierrae-orientalis		
Ranunculaceae	Thalictrum deamii		EN
Ranunculaceae	Thalictrum fendleri		
Ranunculaceae	Thalictrum gibbosum		
Ranunculaceae	Thalictrum guatemalense		
Ranunculaceae	Thalictrum hernandezii		
Ranunculaceae	Thalictrum lanatum		
Ranunculaceae	Thalictrum pubigerum		
Ranunculaceae	Thalictrum standleyi		EN
Ranunculaceae	Thalictrum strigillosum		
Resedaceae	Forchhammeria hintonii	EN	VU

Resedaceae	Forchhammeria matudae		VU
Resedaceae	Forchhammeria pallida		
Resedaceae	Forchhammeria trifoliata		
Resedaceae	Reseda luteola		
Rhamnaceae	Adolphia infesta		
Rhamnaceae	Berchemia scandens		
Rhamnaceae	Ceanothus buxifolius		
Rhamnaceae	Ceanothus caeruleus		
Rhamnaceae	Ceanothus pauciflorus		
Rhamnaceae	Ceanothus perplexans		
Rhamnaceae	Ceanothus tomentosus		
Rhamnaceae	Colubrina angustior		VU
Rhamnaceae	Colubrina arborescens		
Rhamnaceae	Colubrina celtidifolia		
Rhamnaceae	Colubrina ehrenbergii		
Rhamnaceae	Colubrina elliptica		
Rhamnaceae	Colubrina greggii		
Rhamnaceae	Colubrina johnstonii	EN	VU
Rhamnaceae	Colubrina macrocarpa		
Rhamnaceae	Colubrina spinosa		EN
Rhamnaceae	Colubrina triflora		
Rhamnaceae	Colubrina viridis		
Rhamnaceae	Condalia mexicana		
Rhamnaceae	Condalia spathulata		
Rhamnaceae	Frangula breedlovei	EN	VU
Rhamnaceae	Frangula capreifolia		
Rhamnaceae	Frangula capreifolia var. grandifolia		
Rhamnaceae	Frangula caroliniana		EN
Rhamnaceae	Frangula discolor		
Rhamnaceae	Frangula longistyla		
Rhamnaceae	Frangula mcvaughii	EN	
Rhamnaceae	Frangula mucronata		
Rhamnaceae	Frangula sphaerosperma		
Rhamnaceae	Gouania conzattii		

Rhamnaceae	Gouania eurycarpa		
Rhamnaceae	Gouania guiengolensis		VU
Rhamnaceae	Gouania lupuloides		
Rhamnaceae	Gouania obamana		
Rhamnaceae	Gouania polygama		
Rhamnaceae	Gouania rosei		
Rhamnaceae	Gouania stipularis		
Rhamnaceae	Karwinskia calderonii		
Rhamnaceae	Karwinskia humboldtiana		
Rhamnaceae	Karwinskia mollis		
Rhamnaceae	Krugiodendron ferreum		
Rhamnaceae	Rhamnus serrata		
Rhamnaceae	Sageretia elegans		
Rhamnaceae	Sarcomphalus amole		
Rhamnaceae	Sarcomphalus mexicanus		
Rhamnaceae	Sarcomphalus obtusifolius		
Rhizophoraceae	Cassipourea elliptica		
Rhizophoraceae	Cassipourea guianensis		
Rhizophoraceae	Rhizophora mangle	VU	
Rosaceae	Acaena elongata		
Rosaceae	Agrimonia gryposepala		
Rosaceae	Agrimonia pringlei		VU
Rosaceae	Alchemilla aphanoides		
Rosaceae	Alchemilla guatemalensis		
Rosaceae	Alchemilla orbiculata		
Rosaceae	Alchemilla orizabensis		EN
Rosaceae	Alchemilla pectinata		
Rosaceae	Alchemilla procumbens		
Rosaceae	Alchemilla sibbaldiifolia		
Rosaceae	Alchemilla vulcanica		
Rosaceae	Cercocarpus fothergilloides		
Rosaceae	Cercocarpus montanus		
Rosaceae	Crataegus gracilior		
Rosaceae	Crataegus mexicana		

Rosaceae	Crataegus rosei		
Rosaceae	Crataegus serratissima		EN
Rosaceae	Crataegus uniflora		EN
Rosaceae	Eriobotrya japonica		
Rosaceae	Fragaria vesca		
Rosaceae	Fragaria vesca subsp. bracteata		
Rosaceae	Holodiscus argenteus		
Rosaceae	Holodiscus fissus		
Rosaceae	Holodiscus velutinus		
Rosaceae	Lindleya mespiloides		
Rosaceae	Malacomeles denticulata		
Rosaceae	Malacomeles nervosa		
Rosaceae	Malacomeles paniculata		
Rosaceae	Malacomeles psilantha		
Rosaceae	Malus domestica		
Rosaceae	Photinia matudae	VU	
Rosaceae	Photinia mexicana		
Rosaceae	Photinia microcarpa		
Rosaceae	Photinia microcarpa subsp. hintonii		
Rosaceae	Potentilla candicans		
Rosaceae	Potentilla indica		
Rosaceae	Potentilla macdonaldii		EN
Rosaceae	Potentilla ranunculoides		
Rosaceae	Prunus axitliana	EN	EN
Rosaceae	Prunus barbata		
Rosaceae	Prunus brachybotrya		
Rosaceae	Prunus erythroxylon	EN	
Rosaceae	Prunus guatemalensis	EN	
Rosaceae	Prunus lundelliana	EN	
Rosaceae	Prunus matudae		VU
Rosaceae	Prunus myrtifolia		VU
Rosaceae	Prunus occidentalis		EN
Rosaceae	Prunus persica		
Rosaceae	Prunus rhamnoides		

Rosaceae	Prunus salasii	EN	VU	
Rosaceae	Prunus samydoides			
Rosaceae	Prunus serotina			
Rosaceae	Prunus serotina var. salicifolia			
Rosaceae	Prunus tetradenia			
Rosaceae	Prunus tuberculata	EN		
Rosaceae	Purshia plicata			
Rosaceae	Pyracantha crenulata		EN	
Rosaceae	Pyrus communis			
Rosaceae	Rosa × centifolia			
Rosaceae	Rosa carolina			
Rosaceae	Rosa chinensis			
Rosaceae	Rosa multiflora		VU	
Rosaceae	Rosa odorata		VU	
Rosaceae	Rubus × philyrophyllus		VU	
Rosaceae	Rubus adenotrichos			
Rosaceae	Rubus coriifolius			
Rosaceae	Rubus eriocarpus			
Rosaceae	Rubus fagifolius			
Rosaceae	Rubus flagellaris			
Rosaceae	Rubus glaucus			
Rosaceae	Rubus hadrocarpus			
Rosaceae	Rubus humistratus			
Rosaceae	Rubus idaeus			
Rosaceae	Rubus irasuensis		VU	
Rosaceae	Rubus miser		VU	
Rosaceae	Rubus occidentalis		EN	
Rosaceae	Rubus pringlei			
Rosaceae	Rubus sapidus			
Rosaceae	Rubus schiedeanus			
Rosaceae	Rubus trilobus			
Rosaceae	Rubus urticifolius			
Rosaceae	Spiraea × vanhouttei		CR	YES
Rosaceae	Spiraea cantoniensis			

Rosaceae	Vauquelinia australis	VU	
Rosaceae	Xerospiraea hartwegiana		
Rubiaceae	Alibertia edulis		
Rubiaceae	Alseis yucatanensis		
Rubiaceae	Amaioua glomerulata		
Rubiaceae	Appunia guatemalensis		
Rubiaceae	Arachnothryx bertieroides		EN
Rubiaceae	Arachnothryx bourgaei	VU	VU
Rubiaceae	Arachnothryx buddleioides		
Rubiaceae	Arachnothryx capitellata		
Rubiaceae	Arachnothryx capitellata subsp. capitellata		VU
Rubiaceae	Arachnothryx capitellata subsp. pringlei		
Rubiaceae	Arachnothryx chiapensis	VU	EN
Rubiaceae	Arachnothryx chimalaparum	EN	EN
Rubiaceae	Arachnothryx galeottii	CR	
Rubiaceae	Arachnothryx gonzaleoides		
Rubiaceae	Arachnothryx gracilispica		
Rubiaceae	Arachnothryx heteranthera		
Rubiaceae	Arachnothryx izabalensis	EN	EN
Rubiaceae	Arachnothryx laniflora		
Rubiaceae	Arachnothryx leucophylla		
Rubiaceae	Arachnothryx lineolata		EN
Rubiaceae	Arachnothryx linguiformis		VU
Rubiaceae	Arachnothryx myriantha	EN	EN
Rubiaceae	Arachnothryx nitida		
Rubiaceae	Arachnothryx ovandensis		
Rubiaceae	Arachnothryx pyramidalis		
Rubiaceae	Arachnothryx rufescens	EN	EN
Rubiaceae	Arachnothryx secundiflora		
Rubiaceae	Arachnothryx stachyoidea		
Rubiaceae	Arachnothryx thiemei	VU	EN
Rubiaceae	Arachnothryx tuxtlensis		
Rubiaceae	Arachnothryx uxpanapensis		VU
Rubiaceae	Arachnothryx villosa		

Rubiaceae	Arachnothryx wendtii		EN	EN
Rubiaceae	Arcytophyllum serpyllaceum			
Rubiaceae	Augusta rivalis			
Rubiaceae	Balmea stormiae		EN	
Rubiaceae	Bertiera guianensis			
Rubiaceae	Blepharidium guatemalense			
Rubiaceae	Bouvardia bouvardioides			
Rubiaceae	Bouvardia capitata	NT		
Rubiaceae	Bouvardia chrysantha			
Rubiaceae	Bouvardia cordifolia			
Rubiaceae	Bouvardia dictyoneura	NT		
Rubiaceae	Bouvardia erecta	VU		
Rubiaceae	Bouvardia glabra			
Rubiaceae	Bouvardia laevis			
Rubiaceae	Bouvardia langlassei	NT		
Rubiaceae	Bouvardia leiantha			
Rubiaceae	Bouvardia loeseneriana	NT		
Rubiaceae	Bouvardia longiflora			
Rubiaceae	Bouvardia multiflora			
Rubiaceae	Bouvardia quinquenervata			
Rubiaceae	Bouvardia tenuifolia			
Rubiaceae	Bouvardia ternifolia			
Rubiaceae	Bouvardia viminalis			
Rubiaceae	Bouvardia xestosperma			
Rubiaceae	Calycophyllum candidissimum			
Rubiaceae	Calycophyllum spruceanum			EN
Rubiaceae	Cephalanthus occidentalis			
Rubiaceae	Chiococca alba			
Rubiaceae	Chiococca belizensis			
Rubiaceae	Chiococca filipes			
Rubiaceae	Chiococca motleyana			
Rubiaceae	Chiococca oaxacana			
Rubiaceae	Chiococca pachyphylla			
Rubiaceae	Chiococca phaenostemon			

Rubiaceae	Chiococca rubriflora		VU
Rubiaceae	Chiococca sessilifolia	EN	
Rubiaceae	Chione venosa		
Rubiaceae	Chione venosa var. mexicana		
Rubiaceae	Chione venosa var. venosa		
Rubiaceae	Chomelia barbata	VU	
Rubiaceae	Chomelia brachypoda	EN	
Rubiaceae	Chomelia breedlovei	EN	EN
Rubiaceae	Chomelia longituba	CR	EN
Rubiaceae	Chomelia pringlei	VU	
Rubiaceae	Chomelia recordii		EN
Rubiaceae	Chomelia spinosa		
Rubiaceae	Chomelia tenuiflora		EN
Rubiaceae	Cinchona pubescens		EN
Rubiaceae	Coccocypselum cordifolium		
Rubiaceae	Coccocypselum guianense		
Rubiaceae	Coccocypselum herbaceum		
Rubiaceae	Coccocypselum hirsutum		
Rubiaceae	Coccocypselum hispidulum		
Rubiaceae	Coffea arabica	EN	
Rubiaceae	Coffea canephora		VU
Rubiaceae	Coffea liberica		EN
Rubiaceae	Cosmibuena matudae		
Rubiaceae	Cosmocalyx spectabilis	VU	
Rubiaceae	Coussarea chiapensis	CR	EN
Rubiaceae	Coussarea imitans	EN	EN
Rubiaceae	Coussarea impetiolaris		EN
Rubiaceae	Coussarea mexicana	EN	
Rubiaceae	Coutaportla ghiesbreghtiana		
Rubiaceae	Coutaportla guatemalensis	EN	
Rubiaceae	Coutarea hexandra		
Rubiaceae	Crusea calcicola		
Rubiaceae	Crusea calocephala		
Rubiaceae	Crusea coccinea		

Rubiaceae	Crusea coccinea var. breviloba		
Rubiaceae	Crusea diversifolia		
Rubiaceae	Crusea hispida		
Rubiaceae	Crusea hispida var. hispida		
Rubiaceae	Crusea longiflora		
Rubiaceae	Crusea megalocarpa		
Rubiaceae	Crusea parviflora		
Rubiaceae	Crusea psyllioides		
Rubiaceae	Crusea setosa		
Rubiaceae	Declieuxia fruticosa		CR
Rubiaceae	Dentella repens		VU
Rubiaceae	Deppea anisophylla		VU
Rubiaceae	Deppea cornifolia	VU	
Rubiaceae	Deppea erythrorhiza	CR	
Rubiaceae	Deppea grandiflora		
Rubiaceae	Deppea inaequalis		
Rubiaceae	Deppea microphylla		
Rubiaceae	Deppea obtusiflora	EN	
Rubiaceae	Deppea pubescens		
Rubiaceae	Deppea purpurascens		
Rubiaceae	Deppea purpusii		
Rubiaceae	Deppea splendens	EW	VU
Rubiaceae	Deppea tenuiflora		VU
Rubiaceae	Deppea umbellata		
Rubiaceae	Didymaea alsinoides		
Rubiaceae	Didymaea mexicana		
Rubiaceae	Donnellyanthus deamii		
Rubiaceae	Elaeagia utilis		CR
Rubiaceae	Eumachia microdon		
Rubiaceae	Exostema caribaeum		
Rubiaceae	Faramea brachysiphon		EN
Rubiaceae	Faramea glandulosa		
Rubiaceae	Faramea multiflora		VU
Rubiaceae	Faramea occidentalis		

Rubiaceae	Galianthe brasiliensis			
Rubiaceae	Galianthe brasiliensis subsp. angulata			
Rubiaceae	Galianthe brasiliensis subsp. brasiliensis		EN	
Rubiaceae	Galium aschenbornii			
Rubiaceae	Galium fuscum			
Rubiaceae	Galium fuscum subsp. hypadenium			
Rubiaceae	Galium hypocarpium			
Rubiaceae	Galium mexicanum			
Rubiaceae	Galium mexicanum subsp. mexicanum			
Rubiaceae	Galium microphyllum			
Rubiaceae	Galium orizabense			
Rubiaceae	Galium orizabense subsp. orizabense		CR	YES
Rubiaceae	Galium seatonii			
Rubiaceae	Galium triflorum			
Rubiaceae	Galium uncinulatum			
Rubiaceae	Gardenia jasminoides			
Rubiaceae	Genipa americana			
Rubiaceae	Geophila cordifolia		CR	
Rubiaceae	Geophila macropoda			
Rubiaceae	Geophila repens			
Rubiaceae	Glossostipula concinna			
Rubiaceae	Gonzalagunia panamensis			
Rubiaceae	Gonzalagunia tacanensis			
Rubiaceae	Gonzalagunia thyrsoidea	CR		
Rubiaceae	Guettarda camagueyensis		CR	YES
Rubiaceae	Guettarda combsii			
Rubiaceae	Guettarda elliptica			
Rubiaceae	Guettarda gaumeri			
Rubiaceae	Guettarda krugii		CR	YES
Rubiaceae	Guettarda macrocarpa		CR	YES
Rubiaceae	Guettarda macrosperma			
Rubiaceae	Guettarda subcapitata			
Rubiaceae	Guettarda tikalana	VU		
Rubiaceae	Hamelia axillaris			

Rubiaceae	Hamelia barbata		EN	
Rubiaceae	Hamelia calycosa			
Rubiaceae	Hamelia longipes			
Rubiaceae	Hamelia patens			
Rubiaceae	Hamelia rostrata			
Rubiaceae	Hamelia rovirosae	NT		
Rubiaceae	Hamelia xorullensis			
Rubiaceae	Hexasepalum apiculatum			
Rubiaceae	Hexasepalum sarmentosum			
Rubiaceae	Hexasepalum teres			
Rubiaceae	Hillia panamensis			VU
Rubiaceae	Hillia tetrandra			
Rubiaceae	Hindsia longiflora			EN
Rubiaceae	Hintonia latiflora			
Rubiaceae	Hintonia lumana			VU
Rubiaceae	Hintonia standleyana			
Rubiaceae	Hoffmannia altipetens			EN
Rubiaceae	Hoffmannia bullata			
Rubiaceae	Hoffmannia cauliflora		CR	EN
Rubiaceae	Hoffmannia conzattii			
Rubiaceae	Hoffmannia cryptoneura			
Rubiaceae	Hoffmannia cuneatissima			
Rubiaceae	Hoffmannia discolor			
Rubiaceae	Hoffmannia excelsa			
Rubiaceae	Hoffmannia ghiesbreghtii			
Rubiaceae	Hoffmannia huehueteca			VU
Rubiaceae	Hoffmannia ixtlanensis			VU
Rubiaceae	Hoffmannia longepetiolata			EN
Rubiaceae	Hoffmannia nicotianifolia			
Rubiaceae	Hoffmannia orizabensis			VU
Rubiaceae	Hoffmannia phoenicopoda			
Rubiaceae	Hoffmannia psychotriifolia	NT		
Rubiaceae	Hoffmannia regalis			
Rubiaceae	Hoffmannia rhizantha			EN

Rubiaceae	Hoffmannia rotata	
Rubiaceae	Hoffmannia steyermarkii	
Rubiaceae	Hoffmannia strigillosa	EN
Rubiaceae	Houstonia humifusa	EN
Rubiaceae	Houstonia sharpii	VU
Rubiaceae	Houstonia wrightii	
Rubiaceae	Ixora coccinea	
Rubiaceae	Ixora finlaysoniana	
Rubiaceae	Machaonia acuminata	
Rubiaceae	Machaonia erythrocarpa	EN
Rubiaceae	Machaonia erythrocarpa subsp. hondurensis	
Rubiaceae	Machaonia erythrocarpa subsp. parvifolia	
Rubiaceae	Macrocnemum roseum	EN
Rubiaceae	Manettia flexilis	VU
Rubiaceae	Manettia reclinata	
Rubiaceae	Martensianthus galeottii	
Rubiaceae	Mexotis latifolia	
Rubiaceae	Mitchella repens	
Rubiaceae	Mitracarpus breviflorus	
Rubiaceae	Mitracarpus hirtus	
Rubiaceae	Mitracarpus linearifolius	
Rubiaceae	Morinda citrifolia	
Rubiaceae	Morinda panamensis	
Rubiaceae	Mussaenda erythrophylla	VU
Rubiaceae	Neanotis latifolia	VU
Rubiaceae	Nertera granadensis	
Rubiaceae	Nertera granadensis var. granadensis	EN
Rubiaceae	Notopleura anomothyrsa	VU
Rubiaceae	Notopleura hondurensis	
Rubiaceae	Notopleura macrophylla	VU
Rubiaceae	Notopleura parasitica	VU
Rubiaceae	Notopleura tolimensis	VU
Rubiaceae	Notopleura uliginosa	
Rubiaceae	Oldenlandia lancifolia	

Rubiaceae	Oldenlandia latifolia		EN
Rubiaceae	Oldenlandia microtheca		
Rubiaceae	Oldenlandia ovata		
Rubiaceae	Oldenlandia pringlei		VU
Rubiaceae	Paederia ciliata		
Rubiaceae	Palicourea acuminata		
Rubiaceae	Palicourea angustifolia		EN
Rubiaceae	Palicourea axillaris		VU
Rubiaceae	Palicourea berteroana		
Rubiaceae	Palicourea breedlovei	EN	EN
Rubiaceae	Palicourea caerulea		VU
Rubiaceae	Palicourea chlorobotrya		
Rubiaceae	Palicourea crocea		
Rubiaceae	Palicourea cuspidata		
Rubiaceae	Palicourea cyanococca		EN
Rubiaceae	Palicourea deflexa		
Rubiaceae	Palicourea domingensis		
Rubiaceae	Palicourea elata		
Rubiaceae	Palicourea faxlucens		
Rubiaceae	Palicourea galeottiana		
Rubiaceae	Palicourea gardenioides		
Rubiaceae	Palicourea glomerulata		VU
Rubiaceae	Palicourea gracilenta		
Rubiaceae	Palicourea guianensis		
Rubiaceae	Palicourea hebeclada		
Rubiaceae	Palicourea hoffmannseggiana		
Rubiaceae	Palicourea luxurians		VU
Rubiaceae	Palicourea macrantha		
Rubiaceae	Palicourea megalantha		
Rubiaceae	Palicourea minarum		
Rubiaceae	Palicourea padifolia		
Rubiaceae	Palicourea patens		
Rubiaceae	Palicourea phanerandra		
Rubiaceae	Palicourea pubescens		

Rubiaceae	Palicourea racemosa			
Rubiaceae	Palicourea richardiana			
Rubiaceae	Palicourea seleri			
Rubiaceae	Palicourea simiarum			
Rubiaceae	Palicourea simiarum subsp. chiapensis			EN
Rubiaceae	Palicourea sousae		EN	EN
Rubiaceae	Palicourea stipulosa			EN
Rubiaceae	Palicourea tetragona			
Rubiaceae	Palicourea thornei			EN
Rubiaceae	Palicourea tomentosa			
Rubiaceae	Palicourea veracruzensis			
Rubiaceae	Palicourea violacea			EN
Rubiaceae	Palicourea winkleri			
Rubiaceae	Pentas lanceolata			
Rubiaceae	Pinarophyllon flavum	NT		
Rubiaceae	Pittoniotis protracta			
Rubiaceae	Placocarpa mexicana			CR
Rubiaceae	Plocaniophyllon flavum			
Rubiaceae	Pogonopus speciosus			
Rubiaceae	Posoqueria coriacea			
Rubiaceae	Posoqueria latifolia			
Rubiaceae	Pseudomiltemia filisepala			VU
Rubiaceae	Psychotria biaristata			EN
Rubiaceae	Psychotria calophylla			VU
Rubiaceae	Psychotria capensis			
Rubiaceae	Psychotria carthagenensis			
Rubiaceae	Psychotria chagrensis			VU
Rubiaceae	Psychotria clivorum			
Rubiaceae	Psychotria costivenia			
Rubiaceae	Psychotria costivenia subsp. altorum			
Rubiaceae	Psychotria costivenia subsp. costivenia			
Rubiaceae	Psychotria dwyeri		EN	EN
Rubiaceae	Psychotria erythrocarpa			
Rubiaceae	Psychotria flava			

Rubiaceae	Psychotria fruticetorum		
Rubiaceae	Psychotria grandis		
Rubiaceae	Psychotria hidalgensis	VU	
Rubiaceae	Psychotria horizontalis		
Rubiaceae	Psychotria limonensis		
Rubiaceae	Psychotria lorenciana	EN	EN
Rubiaceae	Psychotria lundellii	EN	
Rubiaceae	Psychotria marginata		
Rubiaceae	Psychotria mexiae		
Rubiaceae	Psychotria micrantha		VU
Rubiaceae	Psychotria mirandae		
Rubiaceae	Psychotria nervosa		
Rubiaceae	Psychotria panamensis		
Rubiaceae	Psychotria panamensis var. panamensis		
Rubiaceae	Psychotria papantlensis		
Rubiaceae	Psychotria pleuropoda		VU
Rubiaceae	Psychotria sarapiquensis		
Rubiaceae	Psychotria subsessilis		
Rubiaceae	Psychotria tenuifolia		
Rubiaceae	Psychotria trichotoma		
Rubiaceae	Psychotria viridis		EN
Rubiaceae	Randia aculeata		
Rubiaceae	Randia aculeata var. aculeata		
Rubiaceae	Randia armata		
Rubiaceae	Randia capitata		
Rubiaceae	Randia chiapensis		
Rubiaceae	Randia cinerea		
Rubiaceae	Randia cookii		
Rubiaceae	Randia echinocarpa		
Rubiaceae	Randia grandifolia		
Rubiaceae	Randia guerrerensis	EN	VU
Rubiaceae	Randia hidalgensis		
Rubiaceae	Randia laetevirens		
Rubiaceae	Randia laevigata		

Rubiaceae	Randia lanuginosa	EN	CR	YES
Rubiaceae	Randia longiloba			
Rubiaceae	Randia lonicerioides		EN	
Rubiaceae	Randia matudae			
Rubiaceae	Randia monantha			
Rubiaceae	Randia nelsonii			
Rubiaceae	Randia oaxacana	EN		
Rubiaceae	Randia obcordata			
Rubiaceae	Randia petenensis			
Rubiaceae	Randia pterocarpa			
Rubiaceae	Randia retroflexa			
Rubiaceae	Randia tetracantha			
Rubiaceae	Randia thurberi			
Rubiaceae	Randia vazquezii		EN	
Rubiaceae	Randia xalapensis			
Rubiaceae	Richardia scabra			
Rubiaceae	Richardia tricocca			
Rubiaceae	Rogiera amoena			
Rubiaceae	Rogiera cordata			
Rubiaceae	Rogiera edwardsii		VU	
Rubiaceae	Rogiera gratissima		VU	
Rubiaceae	Rogiera ligustroides	EN		
Rubiaceae	Rogiera stenosiphon			
Rubiaceae	Ronabea latifolia		EN	
Rubiaceae	Rondeletia belizensis	VU	EN	
Rubiaceae	Rovaeanthus strigosus			
Rubiaceae	Rovaeanthus suffrutescens		VU	
Rubiaceae	Rudgea citrifolia			
Rubiaceae	Rudgea cornifolia			
Rubiaceae	Sabicea mexicana			
Rubiaceae	Sabicea villosa			
Rubiaceae	Sherardia arvensis			
Rubiaceae	Simira rhodoclada			
Rubiaceae	Simira salvadorensis			

Rubiaceae	Solenandra mexicana			
Rubiaceae	Sommera arborescens			
Rubiaceae	Sommera chiapensis	EN		
Rubiaceae	Sommera grandis			
Rubiaceae	Sommera guatemalensis	VU		
Rubiaceae	Sommera parva	CR	EN	
Rubiaceae	Spermacoce confusa			
Rubiaceae	Spermacoce exilis		VU	
Rubiaceae	Spermacoce glabra			
Rubiaceae	Spermacoce laevis			
Rubiaceae	Spermacoce ocymifolia			
Rubiaceae	Spermacoce ocymoides			
Rubiaceae	Spermacoce ovalifolia			
Rubiaceae	Spermacoce prostrata			
Rubiaceae	Spermacoce remota			
Rubiaceae	Spermacoce suaveolens			
Rubiaceae	Spermacoce tenuior			
Rubiaceae	Spermacoce tetraquetra			
Rubiaceae	Spermacoce verticillata			
Rubiaceae	Stenaria nigricans			
Rubiaceae	Steyermarkia guatemalensis		EN	
Rubiaceae	Tessiera lithospermoides			
Rubiaceae	Warszewiczia uxpanapensis			
Rubiaceae	Warszewiczia uxpanapensis subsp. uxpanapensis		EN	
Ruppiaceae	Ruppia maritima			
Rutaceae	Amyris attenuata			
Rutaceae	Amyris balsamifera			
Rutaceae	Amyris elemifera			
Rutaceae	Amyris madrensis			
Rutaceae	Amyris maritima		CR	YES
Rutaceae	Amyris purpusii	EN	VU	
Rutaceae	Amyris rekoi	EN		
Rutaceae	Amyris staminosa		CR	
Rutaceae	Casimiroa calderoniae			

Rutaceae	Casimiroa edulis			
Rutaceae	Casimiroa microcarpa	EN	CR	YES
Rutaceae	Casimiroa tetrameria			
Rutaceae	Choisya ternata			
Rutaceae	Citrus × aurantiifolia			
Rutaceae	Citrus × aurantium			
Rutaceae	Citrus × limon			
Rutaceae	Citrus deliciosa		EN	
Rutaceae	Citrus maxima			
Rutaceae	Citrus medica			
Rutaceae	Decatropis bicolor			
Rutaceae	Decatropis paucijuga	VU		
Rutaceae	Decazyx esparzae	CR		
Rutaceae	Decazyx macrophyllus	EN	EN	
Rutaceae	Esenbeckia berlandieri	EN		
Rutaceae	Esenbeckia berlandieri subsp. litoralis			
Rutaceae	Esenbeckia bicolor	EN	EN	
Rutaceae	Esenbeckia feddemae	EN	EN	
Rutaceae	Esenbeckia macrantha	VU		
Rutaceae	Esenbeckia pentaphylla			
Rutaceae	Esenbeckia pentaphylla subsp. belizensis			
Rutaceae	Esenbeckia stephani	EN	EN	
Rutaceae	Helietta lucida			
Rutaceae	Helietta parvifolia			
Rutaceae	Megastigma skinneri			
Rutaceae	Murraya exotica		VU	
Rutaceae	Murraya paniculata			
Rutaceae	Peltostigma pteleoides			
Rutaceae	Pilocarpus racemosus			
Rutaceae	Polyaster boronioides			
Rutaceae	Ptelea trifoliata			
Rutaceae	Ruta chalepensis			
Rutaceae	Ruta graveolens			
Rutaceae	Stauranthus perforatus			

Rutaceae	Toxosiphon lindenii			
Rutaceae	Zanthoxylum acuminatum			
Rutaceae	Zanthoxylum acuminatum subsp. juniperinum			
Rutaceae	Zanthoxylum arborescens			
Rutaceae	Zanthoxylum caribaeum			
Rutaceae	Zanthoxylum ciliatum			
Rutaceae	Zanthoxylum ciliatum subsp. purpursii			
Rutaceae	Zanthoxylum clava-herculis			
Rutaceae	Zanthoxylum ekmanii		VU	
Rutaceae	Zanthoxylum fagara			
Rutaceae	Zanthoxylum fagara subsp. aguilarii			
Rutaceae	Zanthoxylum fagara subsp. culantrilo			
Rutaceae	Zanthoxylum flavum subsp. trichilioides		VU	
Rutaceae	Zanthoxylum foliolosum			
Rutaceae	Zanthoxylum limoncello			
Rutaceae	Zanthoxylum melanostictum			
Rutaceae	Zanthoxylum mollissimum			
Rutaceae	Zanthoxylum panamense	EN	EN	
Rutaceae	Zanthoxylum quassiifolium	CR	VU	
Rutaceae	Zanthoxylum rhoifolium			
Rutaceae	Zanthoxylum riedelianum			
Rutaceae	Zanthoxylum riedelianum subsp. kellermanii			
Rutaceae	Zanthoxylum riedelianum subsp. mayanum		VU	
Sabiaceae	Meliosma alba			
Sabiaceae	Meliosma dentata			
Sabiaceae	Meliosma grandifolia			
Sabiaceae	Meliosma idiopoda		VU	
Sabiaceae	Meliosma matudae	EN		
Sabiaceae	Meliosma mexicana	EN	EN	
Sabiaceae	Meliosma oaxacana		VU	
Sabiaceae	Meliosma seleriana	CR	EN	
Saccolomataceae	Saccoloma elegans		VU	
Saccolomataceae	Saccoloma elegans subsp. chartaceum		CR	YES
Saccolomataceae	Saccoloma galeottii		CR	YES

Saccolomataceae	Saccoloma inaequale			
Salicaceae	Abatia mexicana		VU	
Salicaceae	Bartholomaea sessiliflora		EN	EN
Salicaceae	Casearia aculeata			
Salicaceae	Casearia arborea			
Salicaceae	Casearia arguta			
Salicaceae	Casearia bartlettii			EN
Salicaceae	Casearia corymbosa			
Salicaceae	Casearia elegans			EN
Salicaceae	Casearia emarginata			
Salicaceae	Casearia guianensis			
Salicaceae	Casearia laetioides			
Salicaceae	Casearia mexicana			
Salicaceae	Casearia nitida			
Salicaceae	Casearia sylvestris			
Salicaceae	Casearia tacanensis			
Salicaceae	Casearia thamnia			
Salicaceae	Casearia tremula			
Salicaceae	Homalium racemosum			
Salicaceae	Homalium senarium			
Salicaceae	Lunania mexicana			
Salicaceae	Neopringlea integrifolia			
Salicaceae	Neopringlea viscosa			
Salicaceae	Olmediella betschleriana			
Salicaceae	Piparea dentata			
Salicaceae	Piparea multiflora			
Salicaceae	Pleuranthodendron lindenii			
Salicaceae	Populus alba			
Salicaceae	Populus fremontii			
Salicaceae	Populus mexicana			
Salicaceae	Populus simaroa	NT	VU	
Salicaceae	Prockia crucis			
Salicaceae	Salix bonplandiana			
Salicaceae	Salix cana		VU	

Salicaceae	Salix caroliniana	EN
Salicaceae	Salix chilensis	
Salicaceae	Salix discolor	
Salicaceae	Salix humboldtiana	
Salicaceae	Salix latifolia	
Salicaceae	Salix ledebouriana	VU
Salicaceae	Salix microphylla	
Salicaceae	Salix paradoxa	
Salicaceae	Salix taxifolia	
Salicaceae	Xylosma characantha	
Salicaceae	Xylosma chiapensis	
Salicaceae	Xylosma chlorantha	
Salicaceae	Xylosma cinerea	
Salicaceae	Xylosma flexuosa	
Salicaceae	Xylosma horrida	
Salicaceae	Xylosma intermedia	
Salicaceae	Xylosma oligandra	VU
Salicaceae	Xylosma panamensis	
Salicaceae	Xylosma quichensis	
Salicaceae	Xylosma velutina	
Santalaceae	Antidaphne viscoidea	VU
Santalaceae	Arceuthobium gillii	
Santalaceae	Arceuthobium globosum	
Santalaceae	Arceuthobium globosum subsp. grandicaule	
Santalaceae	Arceuthobium vaginatum	
Santalaceae	Dendrophthora costaricensis	EN
Santalaceae	Phoradendron aguilarii	VU
Santalaceae	Phoradendron annulatum	
Santalaceae	Phoradendron aurantiacum	EN
Santalaceae	Phoradendron bolleanum	
Santalaceae	Phoradendron brachystachyum	
Santalaceae	Phoradendron brevifolium	
Santalaceae	Phoradendron burgeri	EN
Santalaceae	Phoradendron carneum	

Santalaceae	Phoradendron chrysocladon	EN
Santalaceae	Phoradendron crassifolium	
Santalaceae	Phoradendron dipterum	
Santalaceae	Phoradendron falcatum	
Santalaceae	Phoradendron forestierae	
Santalaceae	Phoradendron galeottii	
Santalaceae	Phoradendron herbert-smithii	EN
Santalaceae	Phoradendron heydeanum	EN
Santalaceae	Phoradendron lanatum	
Santalaceae	Phoradendron lanceolatum	
Santalaceae	Phoradendron longifolium	
Santalaceae	Phoradendron minutifolium	
Santalaceae	Phoradendron mucronatum	
Santalaceae	Phoradendron naviculare	
Santalaceae	Phoradendron nervosum	
Santalaceae	Phoradendron oliverianum	VU
Santalaceae	Phoradendron pedicellatum	
Santalaceae	Phoradendron piperoides	
Santalaceae	Phoradendron puberulum	EN
Santalaceae	Phoradendron quadrangulare	
Santalaceae	Phoradendron racemosum	EN
Santalaceae	Phoradendron ramosissimum	EN
Santalaceae	Phoradendron reichenbachianum	
Santalaceae	Phoradendron robinsonii	
Santalaceae	Phoradendron robustissimum	
Santalaceae	Phoradendron rondeletiae	VU
Santalaceae	Phoradendron tonduzii	VU
Santalaceae	Phoradendron treleaseanum	
Santalaceae	Phoradendron undulatum	VU
Santalaceae	Phoradendron velutinum	
Santalaceae	Phoradendron wattii	
Santalaceae	Phoradendron wawrae	
Sapindaceae	Acer grandidentatum	
Sapindaceae	Acer negundo	

Sapindaceae	Acer negundo subsp. mexicanum		
Sapindaceae	Acer saccharinum		EN
Sapindaceae	Acer saccharum		
Sapindaceae	Acer skutchii	CR	
Sapindaceae	Allophylus camptostachys		
Sapindaceae	Allophylus cominia		
Sapindaceae	Allophylus psilospermus		
Sapindaceae	Billia hippocastanum		
Sapindaceae	Blomia prisca		
Sapindaceae	Cardiospermum corindum		
Sapindaceae	Cardiospermum grandiflorum		
Sapindaceae	Cardiospermum halicacabum		
Sapindaceae	Cupania belizensis		
Sapindaceae	Cupania dentata		
Sapindaceae	Cupania glabra		
Sapindaceae	Cupania guatemalensis		
Sapindaceae	Cupania juglandifolia		
Sapindaceae	Cupania mayana	EN	EN
Sapindaceae	Cupania mollis		
Sapindaceae	Cupania rufescens		
Sapindaceae	Cupania scrobiculata		
Sapindaceae	Cupania spectabilis	EN	
Sapindaceae	Dodonaea viscosa		
Sapindaceae	Exothea copalillo	EN	
Sapindaceae	Exothea paniculata		
Sapindaceae	Litchi chinensis		
Sapindaceae	Matayba apetala		
Sapindaceae	Matayba clavelligera		
Sapindaceae	Matayba glaberrima		VU
Sapindaceae	Matayba oppositifolia		
Sapindaceae	Melicoccus oliviformis		
Sapindaceae	Paullinia clavigera		
Sapindaceae	Paullinia costaricensis		
Sapindaceae	Paullinia costata		

Sapindaceae	Paullinia cururu		
Sapindaceae	Paullinia fuscescens		
Sapindaceae	Paullinia pinnata		
Sapindaceae	Paullinia tomentosa		
Sapindaceae	Paullinia turbacensis	EN	
Sapindaceae	Sapindus drummondii		
Sapindaceae	Sapindus saponaria		
Sapindaceae	Serjania acuta		
Sapindaceae	Serjania atrolineata		
Sapindaceae	Serjania brachycarpa		
Sapindaceae	Serjania caracasana		
Sapindaceae	Serjania cardiospermoides		
Sapindaceae	Serjania cystocarpa	CR	YES
Sapindaceae	Serjania depauperata	CR	YES
Sapindaceae	Serjania flaviflora		
Sapindaceae	Serjania goniocarpa		
Sapindaceae	Serjania grosii		
Sapindaceae	Serjania hispida	VU	
Sapindaceae	Serjania impressa	EN	
Sapindaceae	Serjania insignis	EN	
Sapindaceae	Serjania lobulata		
Sapindaceae	Serjania lundellii		
Sapindaceae	Serjania macrocarpa		
Sapindaceae	Serjania meridionalis	EN	
Sapindaceae	Serjania mexicana		
Sapindaceae	Serjania palmeri		
Sapindaceae	Serjania paniculata		
Sapindaceae	Serjania paucidentata		
Sapindaceae	Serjania polystachya	EN	
Sapindaceae	Serjania racemosa		
Sapindaceae	Serjania rachiptera		
Sapindaceae	Serjania rhombea	VU	
Sapindaceae	Serjania rufisepala	EN	
Sapindaceae	Serjania schiedeana		

Sapindaceae	Serjania subtriplinervis			VU	
Sapindaceae	Serjania triquetra				
Sapindaceae	Serjania yucatanensis				
Sapindaceae	Talisia macrophylla				
Sapindaceae	Thouinia acuminata				
Sapindaceae	Thouinia paucidentata				
Sapindaceae	Thouinia villosa				
Sapindaceae	Thouinidium decandrum				
Sapindaceae	Ungnadia speciosa				
Sapindaceae	Urvillea ulmacea				
Sapotaceae	Chrysophyllum argenteum				
Sapotaceae	Chrysophyllum cainito				
Sapotaceae	Chrysophyllum mexicanum				
Sapotaceae	Chrysophyllum oliviforme			VU	
Sapotaceae	Chrysophyllum venezuelanense				
Sapotaceae	Manilkara chicle				
Sapotaceae	Manilkara zapota				
Sapotaceae	Planchonella laetevirens				
Sapotaceae	Planchonella obovata			CR	YES
Sapotaceae	Pouteria amygdalina		VU		
Sapotaceae	Pouteria campechiana				
Sapotaceae	Pouteria durlandii				
Sapotaceae	Pouteria durlandii subsp. durlandii			VU	
Sapotaceae	Pouteria glomerata				
Sapotaceae	Pouteria glomerata subsp. glomerata				
Sapotaceae	Pouteria reticulata				
Sapotaceae	Pouteria rhynchocarpa		EN	EN	
Sapotaceae	Pouteria sapota				
Sapotaceae	Pouteria torta subsp. tuberculata			VU	
Sapotaceae	Sideroxylon americanum				
Sapotaceae	Sideroxylon capiri	VU			
Sapotaceae	Sideroxylon capiri subsp. tempisque				
Sapotaceae	Sideroxylon cartilagineum	EN			
Sapotaceae	Sideroxylon celastrinum				

Sapotaceae	Sideroxylon contrerasii			
Sapotaceae	Sideroxylon durifolium		VU	
Sapotaceae	Sideroxylon eucoriaceum	EN	CR	YES
Sapotaceae	Sideroxylon floribundum		EN	
Sapotaceae	Sideroxylon floribundum subsp. belizense			
Sapotaceae	Sideroxylon foetidissimum			
Sapotaceae	Sideroxylon foetidissimum subsp. gaumeri			
Sapotaceae	Sideroxylon obtusifolium			
Sapotaceae	Sideroxylon obtusifolium subsp. buxifolium			
Sapotaceae	Sideroxylon palmeri			
Sapotaceae	Sideroxylon persimile			
Sapotaceae	Sideroxylon persimile subsp. persimile			
Sapotaceae	Sideroxylon portoricense subsp. minutiflorum			
Sapotaceae	Sideroxylon salicifolium			
Sapotaceae	Sideroxylon stevensonii	VU	VU	
Sapotaceae	Sideroxylon tepicense			
Sapotaceae	Sideroxylon verruculosum			
Saxifragaceae	Heuchera longipetala var. orizabensis			
Saxifragaceae	Heuchera mexicana			
Schisandraceae	Illicium floridanum			
Schizaeaceae	Actinostachys pennula		EN	
Schizaeaceae	Anemia × paraphyllitidis		CR	YES
Schizaeaceae	Anemia × semihirsuta		EN	
Schizaeaceae	Anemia adiantifolia			
Schizaeaceae	Anemia cicutaria		EN	
Schizaeaceae	Anemia hirsuta			
Schizaeaceae	Anemia mexicana			
Schizaeaceae	Anemia oblongifolia			
Schizaeaceae	Anemia phyllitidis			
Schizaeaceae	Anemia speciosa		VU	
Schizaeaceae	Anemia tomentosa			
Schizaeaceae	Lygodium heterodoxum			
Schizaeaceae	Lygodium japonicum		EN	
Schizaeaceae	Lygodium venustum			

Schizaeaceae	Schizaea elegans	VU	
Schizaeaceae	Schizaea poeppigiana		EN
Schlegeliaceae	Gibsoniothamnus cornutus		
Schlegeliaceae	Schlegelia nicaraguensis		VU
Schlegeliaceae	Schlegelia parviflora		VU
Schoepfiaceae	Schoepfia schreberi		
Schoepfiaceae	Schoepfia vacciniiflora		
Scrophulariaceae	Alonsoa meridionalis		
Scrophulariaceae	Buddleja americana		
Scrophulariaceae	Buddleja cordata		
Scrophulariaceae	Buddleja crotonoides		
Scrophulariaceae	Buddleja nitida		
Scrophulariaceae	Buddleja parviflora		
Scrophulariaceae	Buddleja perfoliata		
Scrophulariaceae	Buddleja sessiliflora		
Scrophulariaceae	Buddleja skutchii		
Scrophulariaceae	Capraria biflora		
Scrophulariaceae	Capraria frutescens		
Scrophulariaceae	Capraria saxifragifolia		
Scrophulariaceae	Eremogeton grandiflorus		
Scrophulariaceae	Leucophyllum frutescens		
Scrophulariaceae	Leucophyllum pringlei		
Scrophulariaceae	Leucophyllum revolutum		
Scrophulariaceae	Leucophyllum zygophyllum		
Selaginellaceae	Selaginella apoda		
Selaginellaceae	Selaginella arsiclada		VU
Selaginellaceae	Selaginella chiapensis		VU
Selaginellaceae	Selaginella delicatissima		
Selaginellaceae	Selaginella eurynota		
Selaginellaceae	Selaginella extensa		
Selaginellaceae	Selaginella finitima		VU
Selaginellaceae	Selaginella flagellata		
Selaginellaceae	Selaginella flexuosa		VU
Selaginellaceae	Selaginella guatemalensis		

Selaginellaceae	Selaginella harrisii				
Selaginellaceae	Selaginella hoffmannii				
Selaginellaceae	Selaginella huehuetenangensis				
Selaginellaceae	Selaginella idiospora			CR	YES
Selaginellaceae	Selaginella illecebrosa			EN	
Selaginellaceae	Selaginella kunzeana			EN	
Selaginellaceae	Selaginella lepidophylla				
Selaginellaceae	Selaginella martensii				
Selaginellaceae	Selaginella mickelii				
Selaginellaceae	Selaginella mollis				
Selaginellaceae	Selaginella moritziana			EN	
Selaginellaceae	Selaginella mosorongensis			EN	
Selaginellaceae	Selaginella oaxacana				
Selaginellaceae	Selaginella orizabensis			CR	YES
Selaginellaceae	Selaginella pallescens				
Selaginellaceae	Selaginella pilifera				
Selaginellaceae	Selaginella popayanensis				
Selaginellaceae	Selaginella porphyrospora	EN			
Selaginellaceae	Selaginella pulcherrima				
Selaginellaceae	Selaginella reflexa				
Selaginellaceae	Selaginella sartorii				
Selaginellaceae	Selaginella schiedeana				
Selaginellaceae	Selaginella schizobasis				
Selaginellaceae	Selaginella sertata				
Selaginellaceae	Selaginella silvestris				
Selaginellaceae	Selaginella stellata				
Selaginellaceae	Selaginella stenophylla				
Selaginellaceae	Selaginella steyermarkii			VU	
Selaginellaceae	Selaginella subrugosa			VU	
Selaginellaceae	Selaginella tarapotensis				
Selaginellaceae	Selaginella tarda				
Selaginellaceae	Selaginella wrightii				
Simaroubaceae	Castela erecta				
Simaroubaceae	Castela retusa		EN		

Simaroubaceae	Picrasma mexicana	EN			
Simaroubaceae	Quassia amara				
Simaroubaceae	Recchia connaroides		VU		
Simaroubaceae	Recchia mexicana				
Simaroubaceae	Recchia simplicifolia		EN		
Simaroubaceae	Simarouba amara				
Simaroubaceae	Simarouba glauca				
Siparunaceae	Siparuna gesnerioides				
Siparunaceae	Siparuna grandiflora			EN	
Siparunaceae	Siparuna guianensis			CR	
Siparunaceae	Siparuna thecaphora				
Smilacaceae	Smilax aristolochiifolia				
Smilacaceae	Smilax bona-nox				
Smilacaceae	Smilax domingensis				
Smilacaceae	Smilax glauca				
Smilacaceae	Smilax laurifolia				
Smilacaceae	Smilax maritima			CR	YES
Smilacaceae	Smilax mollis				
Smilacaceae	Smilax moranensis				
Smilacaceae	Smilax obliquata			CR	YES
Smilacaceae	Smilax officinalis				
Smilacaceae	Smilax ornata				
Smilacaceae	Smilax spinosa				
Smilacaceae	Smilax subpubescens				
Smilacaceae	Smilax velutina				
Solanaceae	Bouchetia erecta				
Solanaceae	Brachistus nelsonii				
Solanaceae	Brachistus stramoniifolius				
Solanaceae	Browallia americana				
Solanaceae	Brugmansia × candida				
Solanaceae	Brugmansia arborea		EW		
Solanaceae	Brugmansia suaveolens		EW		
Solanaceae	Brugmansia versicolor		EW	EN	
Solanaceae	Capsicophysalis potosina			VU	

Solanaceae	Capsicum annuum			
Solanaceae	Capsicum chinense			
Solanaceae	Capsicum frutescens			
Solanaceae	Capsicum lanceolatum	EN		
Solanaceae	Capsicum pubescens			
Solanaceae	Capsicum rhomboideum			
Solanaceae	Cestrum anagyris			
Solanaceae	Cestrum aurantiacum			
Solanaceae	Cestrum benthamii			
Solanaceae	Cestrum contrerasianum		EN	
Solanaceae	Cestrum dumetorum			
Solanaceae	Cestrum elegans			
Solanaceae	Cestrum elegantissimum	EN		
Solanaceae	Cestrum endlicheri			
Solanaceae	Cestrum fasciculatum			
Solanaceae	Cestrum flavescens	CR		
Solanaceae	Cestrum fragile		EN	
Solanaceae	Cestrum fraternum		CR	YES
Solanaceae	Cestrum fulvescens	EN		
Solanaceae	Cestrum glanduliferum			
Solanaceae	Cestrum guatemalense	VU		
Solanaceae	Cestrum hartwegii		EN	
Solanaceae	Cestrum laxum			
Solanaceae	Cestrum luteovirescens			
Solanaceae	Cestrum megalophyllum			
Solanaceae	Cestrum nitidum	EN		
Solanaceae	Cestrum nocturnum			
Solanaceae	Cestrum oblongifolium			
Solanaceae	Cestrum racemosum			
Solanaceae	Cestrum roseum			
Solanaceae	Cestrum schlechtendalii			
Solanaceae	Cestrum tomentosum			
Solanaceae	Chamaesaracha cernua			
Solanaceae	Chamaesaracha rzedowskiana		EN	

Solanaceae	Cuatresia cuneata			
Solanaceae	Datura candida			
Solanaceae	Datura discolor			
Solanaceae	Datura innoxia			
Solanaceae	Datura metel			
Solanaceae	Datura pruinosa			
Solanaceae	Datura quercifolia			
Solanaceae	Datura stramonium			
Solanaceae	Jaltomata procumbens			
Solanaceae	Jaltomata repandidentata			
Solanaceae	Juanulloa mexicana			
Solanaceae	Lycianthes acapulcensis			
Solanaceae	Lycianthes anomala		VU	
Solanaceae	Lycianthes armentalis			
Solanaceae	Lycianthes arrazolensis			
Solanaceae	Lycianthes biflora		CR	YES
Solanaceae	Lycianthes breedlovei		CR	YES
Solanaceae	Lycianthes ceratocalycia	VU	EN	
Solanaceae	Lycianthes chiapensis			
Solanaceae	Lycianthes ciliolata			
Solanaceae	Lycianthes connata	VU		
Solanaceae	Lycianthes cuchumatanensis		VU	
Solanaceae	Lycianthes dejecta			
Solanaceae	Lycianthes geminiflora			
Solanaceae	Lycianthes gorgonea			
Solanaceae	Lycianthes heteroclita			
Solanaceae	Lycianthes hypoleuca	VU		
Solanaceae	Lycianthes inaequilatera			
Solanaceae	Lycianthes lenta			
Solanaceae	Lycianthes limitanea			
Solanaceae	Lycianthes nitida			
Solanaceae	Lycianthes ocellata		EN	
Solanaceae	Lycianthes orogenes		VU	
Solanaceae	Lycianthes pilifera	VU		

Solanaceae	Lycianthes pilosissima		
Solanaceae	Lycianthes purpusii		
Solanaceae	Lycianthes quichensis		
Solanaceae	Lycianthes rzedowskii		
Solanaceae	Lycianthes sideroxyloides		
Solanaceae	Lycianthes stephanocalyx		
Solanaceae	Lycianthes synanthera		
Solanaceae	Lycianthes tricolor		
Solanaceae	Lycium berlandieri		
Solanaceae	Nicandra physalodes		
Solanaceae	Nicotiana glauca		
Solanaceae	Nicotiana obtusifolia		
Solanaceae	Nicotiana plumbaginifolia		
Solanaceae	Nicotiana tabacum		
Solanaceae	Nierembergia angustifolia		
Solanaceae	Physalis angulata		
Solanaceae	Physalis arborescens		
Solanaceae	Physalis campanula		
Solanaceae	Physalis campechiana		
Solanaceae	Physalis cinerascens		
Solanaceae	Physalis cordata		
Solanaceae	Physalis glabra		
Solanaceae	Physalis gracilis		
Solanaceae	Physalis greenmanii	EN	
Solanaceae	Physalis hintonii		
Solanaceae	Physalis hirsuta		EN
Solanaceae	Physalis ignota		VU
Solanaceae	Physalis ixocarpa		
Solanaceae	Physalis lagascae		
Solanaceae	Physalis leptophylla		
Solanaceae	Physalis melanocystis		
Solanaceae	Physalis microcarpa		
Solanaceae	Physalis minuta		
Solanaceae	Physalis mollis		

Solanaceae	Physalis nicandroides			
Solanaceae	Physalis orizabae			
Solanaceae	Physalis patula			
Solanaceae	Physalis philadelphica			
Solanaceae	Physalis philippensis		CR	YES
Solanaceae	Physalis pruinosa			
Solanaceae	Physalis pubescens			
Solanaceae	Physalis solanacea			
Solanaceae	Physalis sordida			
Solanaceae	Physalis stapelioides			
Solanaceae	Physalis subrepens			
Solanaceae	Physalis tehuacanensis	CR	EN	
Solanaceae	Plowmania nyctaginoides		VU	
Solanaceae	Poortmannia speciosa		CR	
Solanaceae	Schraderanthus viscosus	VU		
Solanaceae	Schultesianthus coriaceus		CR	
Solanaceae	Schultesianthus leucanthus		EN	
Solanaceae	Schultesianthus uniflorus	EN	VU	
Solanaceae	Schwenckia americana			
Solanaceae	Schwenckia grandiflora		CR	YES
Solanaceae	Solandra grandiflora			
Solanaceae	Solandra guttata			
Solanaceae	Solandra maxima			
Solanaceae	Solandra nizandensis	VU		
Solanaceae	Solanum acerifolium			
Solanaceae	Solanum acutilobum		CR	YES
Solanaceae	Solanum aligerum			
Solanaceae	Solanum alternatopinnatum		EN	
Solanaceae	Solanum americanum			
Solanaceae	Solanum angustifolium			
Solanaceae	Solanum anomalum		EN	YES
Solanaceae	Solanum aphyodendron			
Solanaceae	Solanum appendiculatum			
Solanaceae	Solanum argentinum		VU	

Solanaceae	Solanum asperolanatum		
Solanaceae	Solanum asperum		
Solanaceae	Solanum aturense		
Solanaceae	Solanum betaceum		
Solanaceae	Solanum bicolor		
Solanaceae	Solanum bicorne		
Solanaceae	Solanum brevipedicellatum	VU	
Solanaceae	Solanum bulbocastanum		
Solanaceae	Solanum campechiense		
Solanaceae	Solanum candidum		
Solanaceae	Solanum capsicoides		VU
Solanaceae	Solanum chiapasense		
Solanaceae	Solanum chrysotrichum		
Solanaceae	Solanum clarum	VU	CR
Solanaceae	Solanum cordovense		
Solanaceae	Solanum corymbosum		
Solanaceae	Solanum crinitum		
Solanaceae	Solanum dasyanthum		
Solanaceae	Solanum deflexum		
Solanaceae	Solanum demissum		
Solanaceae	Solanum dimidiatum		VU
Solanaceae	Solanum diphyllum		
Solanaceae	Solanum diversifolium		
Solanaceae	Solanum douglasii		
Solanaceae	Solanum dulcamaroides		
Solanaceae	Solanum edinense		
Solanaceae	Solanum elaeagnifolium		
Solanaceae	Solanum erianthum		
Solanaceae	Solanum ferrugineum		
Solanaceae	Solanum glaucescens		
Solanaceae	Solanum hazenii		
Solanaceae	Solanum heterodoxum		
Solanaceae	Solanum hirtum		
Solanaceae	Solanum hjertingii		

Solanaceae	Solanum houstonii		
Solanaceae	Solanum ionidium		
Solanaceae	Solanum iopetalum		
Solanaceae	Solanum jamaicense		
Solanaceae	Solanum lanceifolium		
Solanaceae	Solanum lanceolatum		
Solanaceae	Solanum laxum		
Solanaceae	Solanum lepidotum		
Solanaceae	Solanum lesteri		VU
Solanaceae	Solanum lignescens		
Solanaceae	Solanum lycopersicum		
Solanaceae	Solanum mammosum		
Solanaceae	Solanum mauritianum		
Solanaceae	Solanum melongena		
Solanaceae	Solanum mitlense		
Solanaceae	Solanum morelliforme		
Solanaceae	Solanum myriacanthum		
Solanaceae	Solanum nakurense		VU
Solanaceae	Solanum narcoticosmum		VU
Solanaceae	Solanum nigrescens		
Solanaceae	Solanum nigricans		
Solanaceae	Solanum nigrum		
Solanaceae	Solanum nudum		
Solanaceae	Solanum oxycarpum	EN	
Solanaceae	Solanum pectinatum		
Solanaceae	Solanum pimpinellifolium		
Solanaceae	Solanum polyadenium		
Solanaceae	Solanum pruinosum		
Solanaceae	Solanum pseudocapsicum		
Solanaceae	Solanum pubigerum		
Solanaceae	Solanum pulverulentifolium	EN	VU
Solanaceae	Solanum quitoense		
Solanaceae	Solanum refractum		
Solanaceae	Solanum rostratum		

Solanaceae	Solanum rovirosanum			
Solanaceae	Solanum rude-pannum			
Solanaceae	Solanum rugosum			
Solanaceae	Solanum schenckii	EN		
Solanaceae	Solanum schlechtendalianum			
Solanaceae	Solanum seaforthianum			
Solanaceae	Solanum skutchii			
Solanaceae	Solanum sousae		EN	
Solanaceae	Solanum splendens			
Solanaceae	Solanum stoloniferum			
Solanaceae	Solanum suaveolens			
Solanaceae	Solanum tampicense			
Solanaceae	Solanum torvum			
Solanaceae	Solanum trizygum			
Solanaceae	Solanum tuberosum			
Solanaceae	Solanum tuerckheimii			
Solanaceae	Solanum umbellatum			
Solanaceae	Solanum verrucosum			
Solanaceae	Solanum viarum		VU	
Solanaceae	Solanum volubile			
Solanaceae	Solanum wendlandii			
Solanaceae	Solanum wrightii			
Solanaceae	Tzeltalia amphitricha		VU	
Solanaceae	Witheringia meiantha			
Solanaceae	Witheringia mexicana			
Solanaceae	Witheringia solanacea			
Solanaceae	Witheringia stellata	VU		
Staphyleaceae	Staphylea insignis			
Staphyleaceae	Staphylea tricornuta			
Staphyleaceae	Turpinia occidentalis			
Staphyleaceae	Turpinia occidentalis subsp. breviflora		VU	
Staphyleaceae	Turpinia parvifoliola		CR	YES
Styracaceae	Styrax argenteus			
Styracaceae	Styrax argenteus var. ramirezii			

Styracaceae	Styrax conterminus			EN	
Styracaceae	Styrax glabrescens				
Styracaceae	Styrax glabrescens var. pilosus				
Styracaceae	Styrax lanceolatus			EN	
Styracaceae	Styrax magnus		VU		
Styracaceae	Styrax warscewiczii				
Symplocaceae	Symplocos breedlovei		EN		
Symplocaceae	Symplocos citrea				
Symplocaceae	Symplocos coccinea	NT			
Symplocaceae	Symplocos excelsa	NT	EN		
Symplocaceae	Symplocos hartwegii				
Symplocaceae	Symplocos jurgensenii				
Symplocaceae	Symplocos limoncillo				
Symplocaceae	Symplocos pycnantha				
Symplocaceae	Symplocos schiedeana		VU		
Symplocaceae	Symplocos speciosa				
Talinaceae	Talinum fruticosum				
Talinaceae	Talinum paniculatum				
Taxaceae	Taxus globosa	NT	EN		
Tetrachondraceae	Polypremum procumbens				
Theaceae	Camellia sinensis			EN	
Theaceae	Gordonia brenesii				
Thymelaeaceae	Daphnopsis americana				
Thymelaeaceae	Daphnopsis americana subsp. guatemalensis			VU	
Thymelaeaceae	Daphnopsis brevifolia		EN		
Thymelaeaceae	Daphnopsis ficina				
Thymelaeaceae	Daphnopsis flavida		EN	VU	
Thymelaeaceae	Daphnopsis liebmannii		EN	VU	
Thymelaeaceae	Daphnopsis macrocarpa			CR	YES
Thymelaeaceae	Daphnopsis malacophylla		VU	VU	
Thymelaeaceae	Daphnopsis megacarpa		EN		
Thymelaeaceae	Daphnopsis mollis		VU		
Thymelaeaceae	Daphnopsis purpusii		VU		
Thymelaeaceae	Daphnopsis radiata		VU		

Thymelaeaceae	Daphnopsis selerorum			VU	
Thymelaeaceae	Daphnopsis tuerckheimiana			VU	
Ticodendraceae	Ticodendron incognitum				
Tovariaceae	Tovaria diffusa				
Tovariaceae	Tovaria pendula				
Trigoniaceae	Trigonia eriosperma			VU	
Trigoniaceae	Trigonia eriosperma subsp. membranacea				
Trigoniaceae	Trigonia rugosa				
Triuridaceae	Lacandonia schismatica	NT		VU	
Triuridaceae	Triuris brevistylis			CR	YES
Triuridaceae	Triuris hyalina			CR	YES
Tropaeolaceae	Tropaeolum emarginatum			VU	
Tropaeolaceae	Tropaeolum majus				
Tropaeolaceae	Tropaeolum moritzianum			CR	
Typhaceae	Typha angustifolia				
Typhaceae	Typha domingensis				
Typhaceae	Typha latifolia				
Ulmaceae	Ampelocera hottlei				
Ulmaceae	Phyllostylon rhamnoides				
Ulmaceae	Ulmus ismaelis			EN	
Ulmaceae	Ulmus mexicana				
Urticaceae	Boehmeria aspera			EN	
Urticaceae	Boehmeria bullata subsp. coriacea			EN	
Urticaceae	Boehmeria caudata				
Urticaceae	Boehmeria cylindrica				
Urticaceae	Boehmeria pavonii			EN	
Urticaceae	Boehmeria radiata				
Urticaceae	Boehmeria ramiflora				
Urticaceae	Boehmeria ulmifolia				
Urticaceae	Cecropia obtusifolia				
Urticaceae	Cecropia peltata				
Urticaceae	Coussapoa oligocephala		VU		
Urticaceae	Coussapoa purpusii		VU		
Urticaceae	Discocnide mexicana				

Urticaceae	Hemistylus odontophylla	VU	
Urticaceae	Laportea canadensis		
Urticaceae	Myriocarpa bifurca		
Urticaceae	Myriocarpa cordifolia		
Urticaceae	Myriocarpa cubilgueitzensis	EN	
Urticaceae	Myriocarpa densiflora		
Urticaceae	Myriocarpa heterospicata		
Urticaceae	Myriocarpa longipes		
Urticaceae	Myriocarpa obovata		
Urticaceae	Parietaria debilis		
Urticaceae	Parietaria pensylvanica		
Urticaceae	Phenax hirtus		
Urticaceae	Phenax laevigatus	CR	YES
Urticaceae	Phenax mexicanus		
Urticaceae	Phenax poiretii		
Urticaceae	Phenax rugosus		
Urticaceae	Phenax sonneratii	VU	
Urticaceae	Pilea acuminata		
Urticaceae	Pilea auriculata	VU	
Urticaceae	Pilea botterii	CR	YES
Urticaceae	Pilea cadierei		
Urticaceae	Pilea chiapensis	VU	
Urticaceae	Pilea costaricensis	EN	
Urticaceae	Pilea daguensis	EN	
Urticaceae	Pilea dauciodora		
Urticaceae	Pilea ecboliophylla	VU	
Urticaceae	Pilea falcata	EN	
Urticaceae	Pilea glabra		
Urticaceae	Pilea hyalina		
Urticaceae	Pilea imparifolia	EN	
Urticaceae	Pilea irrorata		
Urticaceae	Pilea killipiana	EN	YES
Urticaceae	Pilea matama	CR	YES
Urticaceae	Pilea mexicana		

Urticaceae	Pilea microphylla		
Urticaceae	Pilea pansamalana		
Urticaceae	Pilea parietaria		VU
Urticaceae	Pilea picta		EN
Urticaceae	Pilea pubescens		
Urticaceae	Pilea purulensis		
Urticaceae	Pilea quercifolia		
Urticaceae	Pilea riparia		
Urticaceae	Pilea serpyllifolia		EN
Urticaceae	Pilea tridentata		EN
Urticaceae	Pourouma mollis subsp. triloba		
Urticaceae	Pouzolzia guatemalana var. nivea		
Urticaceae	Pouzolzia occidentalis		
Urticaceae	Pouzolzia purpusii		
Urticaceae	Urera baccifera		
Urticaceae	Urera caracasana		
Urticaceae	Urera elata		
Urticaceae	Urera glabriuscula		
Urticaceae	Urera killipiana		
Urticaceae	Urera lianoides		EN
Urticaceae	Urera martiniana	EN	EN
Urticaceae	Urera simplex		
Urticaceae	Urera verrucosa		
Urticaceae	Urtica chamaedryoides		
Urticaceae	Urtica dioica		
Urticaceae	Urtica mexicana		
Urticaceae	Urtica spirealis		
Urticaceae	Urtica urens		
Verbenaceae	Aloysia chiapensis		
Verbenaceae	Aloysia citrodora		
Verbenaceae	Bouchea bifurca		
Verbenaceae	Bouchea nelsonii		
Verbenaceae	Bouchea prismatica		
Verbenaceae	Citharexylum affine		

Verbenaceae	Citharexylum berlandieri		
Verbenaceae	Citharexylum bourgeauanum		EN
Verbenaceae	Citharexylum caudatum		
Verbenaceae	Citharexylum crassifolium	EN	VU
Verbenaceae	Citharexylum donnell-smithii		
Verbenaceae	Citharexylum guatemalense		EN
Verbenaceae	Citharexylum hexangulare		
Verbenaceae	Citharexylum hidalgense		
Verbenaceae	Citharexylum hirtellum		
Verbenaceae	Citharexylum kerberi	EN	EN
Verbenaceae	Citharexylum ligustrifolium		VU
Verbenaceae	Citharexylum lucidum		VU
Verbenaceae	Citharexylum mexicanum	VU	
Verbenaceae	Citharexylum mocinoi		
Verbenaceae	Citharexylum oleinum		
Verbenaceae	Citharexylum schottii		
Verbenaceae	Citharexylum steyermarkii	EN	EN
Verbenaceae	Citharexylum tetramerum		
Verbenaceae	Duranta costaricensis		EN
Verbenaceae	Duranta erecta		
Verbenaceae	Lantana × urticoides		
Verbenaceae	Lantana achyranthifolia		
Verbenaceae	Lantana camara		
Verbenaceae	Lantana camara subsp. aculeata		
Verbenaceae	Lantana camara subsp. camara		
Verbenaceae	Lantana camara subsp. glandulosissima		
Verbenaceae	Lantana canescens		
Verbenaceae	Lantana chiapasensis		EN
Verbenaceae	Lantana hirsuta		
Verbenaceae	Lantana hirta		
Verbenaceae	Lantana horrida		
Verbenaceae	Lantana horrida subsp. horrida		
Verbenaceae	Lantana involucrata		
Verbenaceae	Lantana montevidensis		

Verbenaceae	Lantana scabrida	EN
Verbenaceae	Lantana trifolia	
Verbenaceae	Lantana velutina	
Verbenaceae	Lippia alba	
Verbenaceae	Lippia bracteosa	
Verbenaceae	Lippia cardiostegia	
Verbenaceae	Lippia chiapasensis	
Verbenaceae	Lippia dulcis	
Verbenaceae	Lippia inopinata	
Verbenaceae	Lippia myriocephala	
Verbenaceae	Lippia oaxacana	
Verbenaceae	Lippia origanoides	
Verbenaceae	Lippia stoechadifolia	
Verbenaceae	Lippia umbellata	
Verbenaceae	Petrea volubilis	
Verbenaceae	Petrea xolocotzia	VU
Verbenaceae	Phyla lanceolata	
Verbenaceae	Phyla nodiflora	
Verbenaceae	Phyla nodiflora var. reptans	
Verbenaceae	Priva aspera	
Verbenaceae	Priva grandiflora	
Verbenaceae	Priva lappulacea	
Verbenaceae	Priva mexicana	
Verbenaceae	Rehdera penninervia	EN
Verbenaceae	Stachytarpheta cayennensis	
Verbenaceae	Stachytarpheta frantzii	
Verbenaceae	Stachytarpheta indica	
Verbenaceae	Stachytarpheta jamaicensis	
Verbenaceae	Stachytarpheta mutabilis	
Verbenaceae	Tamonea curassavica	
Verbenaceae	Verbena bipinnatifida	
Verbenaceae	Verbena bipinnatifida var. nepetifolia	
Verbenaceae	Verbena canadensis	
Verbenaceae	Verbena canescens	

Verbenaceae	Verbena carolina			
Verbenaceae	Verbena delticola			
Verbenaceae	Verbena ehrenbergiana			
Verbenaceae	Verbena elegans			
Verbenaceae	Verbena gooddingii			
Verbenaceae	Verbena lasiostachys			
Verbenaceae	Verbena litoralis			
Verbenaceae	Verbena menthifolia			
Verbenaceae	Verbena recta			
Verbenaceae	Verbena scabra			
Verbenaceae	Verbena teucriifolia			
Viburnaceae	Sambucus canadensis			
Viburnaceae	Sambucus mexicana			
Viburnaceae	Sambucus nigra			
Viburnaceae	Sambucus peruviana		EN	
Viburnaceae	Viburnum acutifolium			
Viburnaceae	Viburnum blandum			
Viburnaceae	Viburnum caudatum	VU		
Viburnaceae	Viburnum ciliatum	VU		
Viburnaceae	Viburnum disjunctum		VU	
Viburnaceae	Viburnum elatum			
Viburnaceae	Viburnum hartwegii			
Viburnaceae	Viburnum jucundum			
Viburnaceae	Viburnum lautum			
Viburnaceae	Viburnum macdougallii	EN	VU	
Viburnaceae	Viburnum microcarpum			
Viburnaceae	Viburnum obtusatum	EN	VU	
Viburnaceae	Viburnum tiliifolium			
Violaceae	Calyptrion arboreum			
Violaceae	Hybanthus longipes		VU	
Violaceae	Hybanthus serrulatus			
Violaceae	Hybanthus thiemei			
Violaceae	Ixchelia mexicana			
Violaceae	Ixchelia uxpanapana		CR	YES

Violaceae	Orthion malpighiifolium		
Violaceae	Orthion montanum	EN	VU
Violaceae	Orthion oblanceolatum		
Violaceae	Orthion subsessile		
Violaceae	Orthion veracruzense	EN	VU
Violaceae	Pombalia glabra		
Violaceae	Pombalia oppositifolia		
Violaceae	Pombalia prunifolia		EN
Violaceae	Pombalia sylvicola		VU
Violaceae	Pombalia verbenacea		
Violaceae	Rinorea guatemalensis		
Violaceae	Rinorea hummelii		
Violaceae	Rinorea sylvatica		EN
Violaceae	Viola barroetana		
Violaceae	Viola grahamii		
Violaceae	Viola hookeriana		
Violaceae	Viola jalapaensis		
Violaceae	Viola nannei		
Violaceae	Viola odorata		
Violaceae	Viola painteri		
Violaceae	Viola scandens		
Vitaceae	Ampelocissus acapulcensis		
Vitaceae	Ampelocissus erdvendbergiana		
Vitaceae	Ampelocissus mesoamericana		VU
Vitaceae	Ampelopsis cordata		EN
Vitaceae	Ampelopsis denudata		
Vitaceae	Cissus alata		
Vitaceae	Cissus biformifolia		
Vitaceae	Cissus cacuminis		
Vitaceae	Cissus discolor		
Vitaceae	Cissus erosa		
Vitaceae	Cissus gossypiifolia		
Vitaceae	Cissus microcarpa		
Vitaceae	Cissus serrulatifolia		EN

Vitaceae	Cissus tiliacea				
Vitaceae	Cissus trianae				
Vitaceae	Cissus trifoliata				
Vitaceae	Cissus verticillata				
Vitaceae	Parthenocissus quinquefolia				
Vitaceae	Vitis aestivalis			VU	
Vitaceae	Vitis berlandieri				
Vitaceae	Vitis bourgaeana				
Vitaceae	Vitis cinerea				
Vitaceae	Vitis popenoei				
Vitaceae	Vitis rotundifolia				
Vitaceae	Vitis tiliifolia				
Vochysiaceae	Vochysia guatemalensis				
Vochysiaceae	Vochysia tabascana			EN	
Winteraceae	Drimys granadensis				
Winteraceae	Drimys granadensis var. mexicana				
Winteraceae	Drimys winteri				
Xyridaceae	Xyris jupicai				
Zamiaceae	Ceratozamia alvarezii	EN	EN	VU	
Zamiaceae	Ceratozamia becerrae	VU	EN	VU	
Zamiaceae	Ceratozamia brevifrons			EN	
Zamiaceae	Ceratozamia decumbens	EN		CR	YES
Zamiaceae	Ceratozamia euryphyllidia	EN	CR	VU	
Zamiaceae	Ceratozamia fuscoviridis		CR	VU	
Zamiaceae	Ceratozamia hildae	VU	EN	VU	
Zamiaceae	Ceratozamia kuesteriana	EN	CR	VU	
Zamiaceae	Ceratozamia latifolia	EN	EN		
Zamiaceae	Ceratozamia matudae	EN	EN		
Zamiaceae	Ceratozamia mexicana	VU	VU		
Zamiaceae	Ceratozamia miqueliana	EN	CR		
Zamiaceae	Ceratozamia mixeorum	EN	EN	VU	
Zamiaceae	Ceratozamia morettii	EN	EN	EN	
Zamiaceae	Ceratozamia robusta	VU	EN		
Zamiaceae	Ceratozamia santillanii			CR	YES

Zamiaceae	Ceratozamia vovidesii	EN	VU	VU
Zamiaceae	Ceratozamia zaragozae	EN	CR	VU
Zamiaceae	Ceratozamia zoquorum	EN	CR	EN
Zamiaceae	Dioon angustifolium	EN	VU	
Zamiaceae	Dioon edule	EN		
Zamiaceae	Dioon holmgrenii	EN	EN	VU
Zamiaceae	Dioon merolae	EN	VU	
Zamiaceae	Dioon rzedowskii	EN	EN	EN
Zamiaceae	Dioon spinulosum	EN	EN	
Zamiaceae	Zamia × katzeriana			
Zamiaceae	Zamia cremnophila	EN	EN	EN
Zamiaceae	Zamia fischeri	EN	EN	
Zamiaceae	Zamia furfuracea	EN	EN	
Zamiaceae	Zamia lacandona	EN	EN	VU
Zamiaceae	Zamia loddigesii	VU		
Zamiaceae	Zamia paucijuga	NT		
Zamiaceae	Zamia prasina		CR	
Zamiaceae	Zamia purpurea	NT	CR	
Zamiaceae	Zamia spartea	EN	CR	VU
Zamiaceae	Zamia variegata	NT	EN	EN
Zamiaceae	Zamia vazquezii	NT	CR	EN
Zamiaceae	Zamia verschaffeltii			
Zingiberaceae	Alpinia purpurata			
Zingiberaceae	Alpinia zerumbet			
Zingiberaceae	Curcuma longa			EN
Zingiberaceae	Etlingera elatior			
Zingiberaceae	Hedychium coronarium			
Zingiberaceae	Hedychium gardnerianum			VU
Zingiberaceae	Renealmia alpinia			
Zingiberaceae	Renealmia aromatica			
Zingiberaceae	Renealmia cernua			EN
Zingiberaceae	Renealmia mexicana			
Zingiberaceae	Renealmia pacifica			
Zingiberaceae	Zingiber officinale			

Zingiberaceae	Zingiber spectabile			
Zingiberaceae	Zingiber zerumbet			EN
Zygophyllaceae	Guaiacum coulteri	VU	VU	
Zygophyllaceae	Guaiacum sanctum	VU		
Zygophyllaceae	Kallstroemia maxima			
Zygophyllaceae	Kallstroemia pubescens			
Zygophyllaceae	Kallstroemia rosei			
Zygophyllaceae	Tribulus cistoides			
Zygophyllaceae	Tribulus terrestris			
