# Supplementary figures and images for: How diverse are the mountain karst forests of Mexico?
Source: PLoS One. 2023 Oct 4;18(10):e0292352. doi: 10.1371/journal.pone.0292352 (PMC10550121; doi:10.1371/journal.pone.0292352)

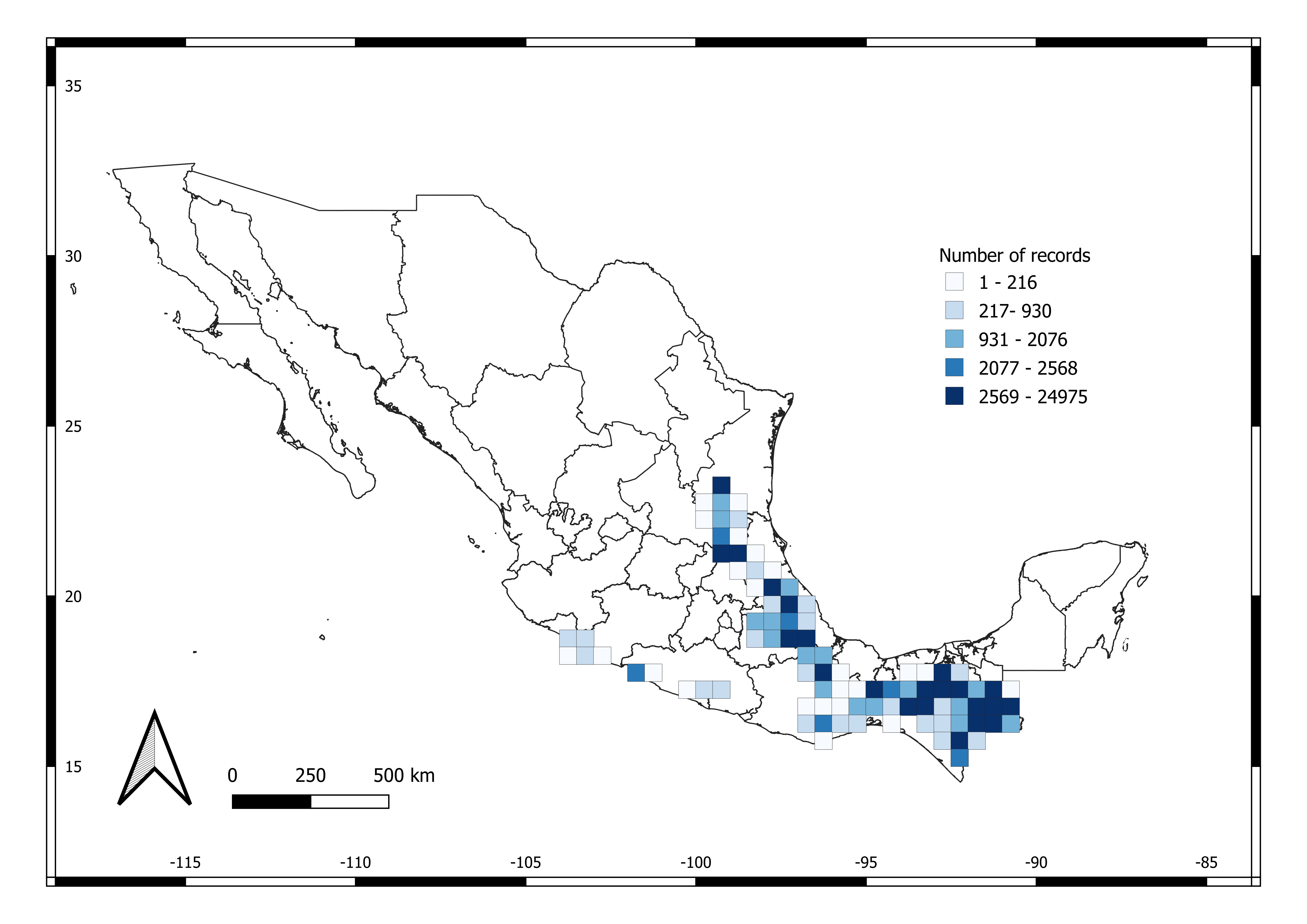

Supplement: S1 Fig — Background map: political division of Mexico provided by Comisión Nacional de Áreas Naturales Protegidas (CONANP) under CCBY 4.0 International License. (PNG) [file pone.0292352.s002.png]

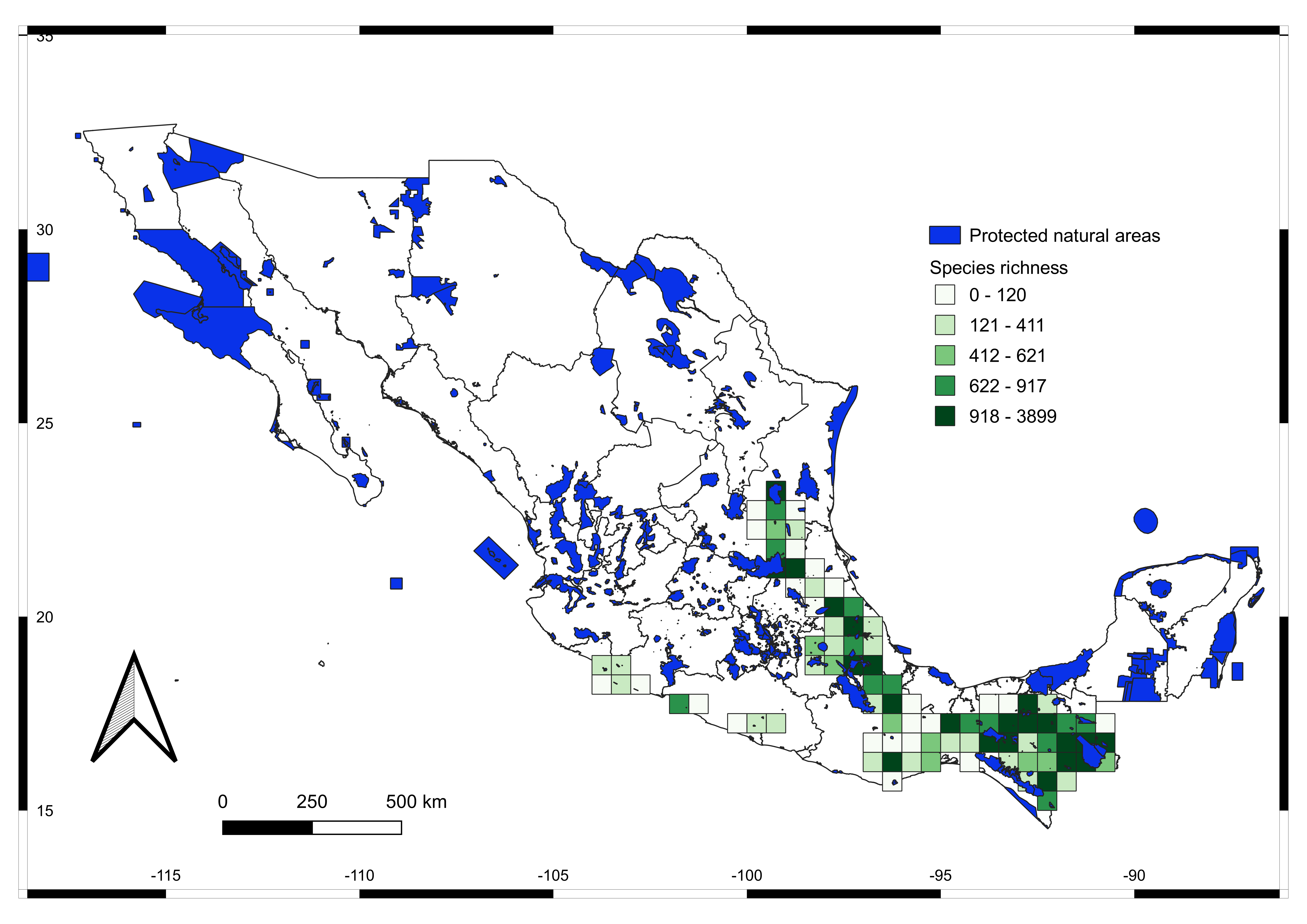

Supplement: S2 Fig — Background maps: political division of Mexico and protected natural areas provided by Comisión Nacional de Áreas Naturales Protegidas (CONANP) under CCBY 4.0 International License. (PNG) [file pone.0292352.s003.png]

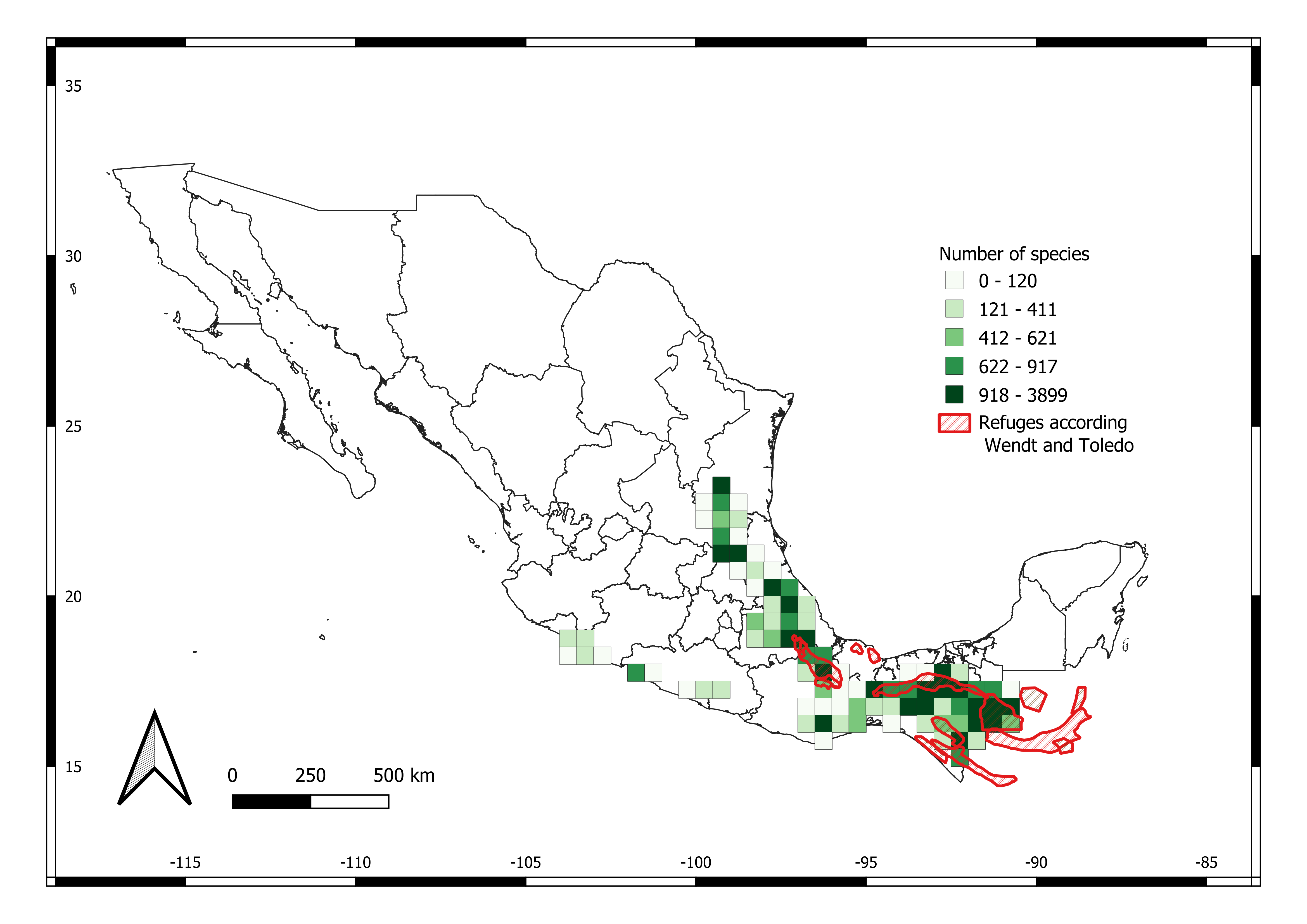

Supplement: S3 Fig — The colored polygons indicate the location of the floristic refuges in Mexico according to [2, 52]. Background map: political division of Mexico provided by Comisión Nacional de Áreas Naturales Protegidas (CONANP) under CCBY 4.0 International License. (PNG) [file pone.0292352.s004.png]

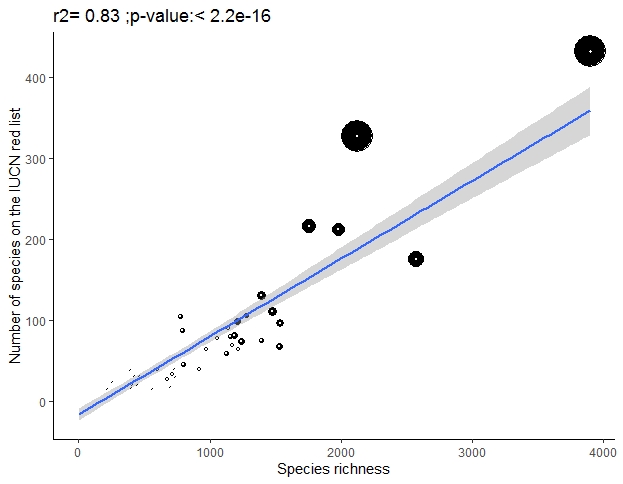

Supplement: S4 Fig — (JPEG) [file pone.0292352.s005.jpeg]

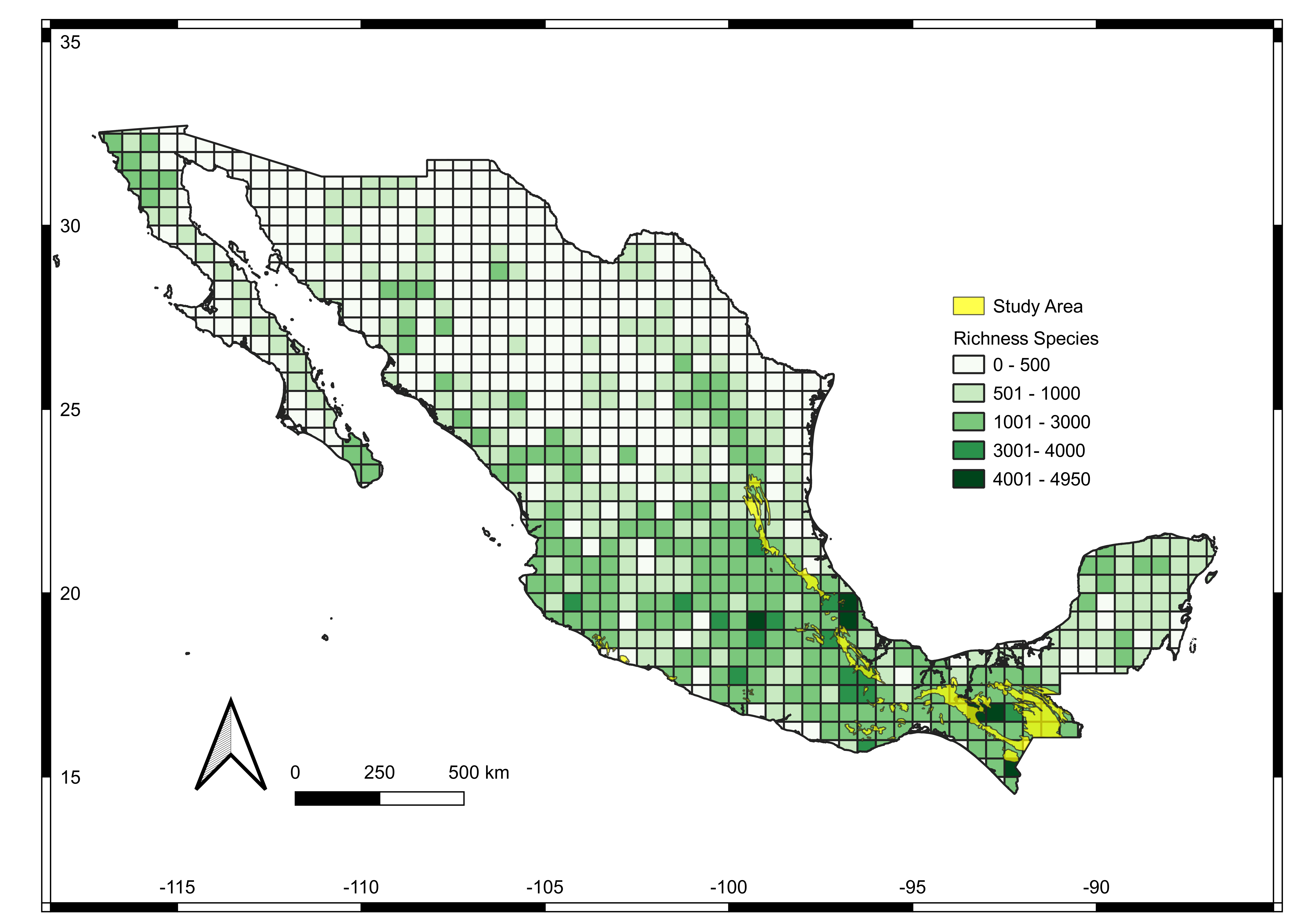

Supplement: S5 Fig — Background map: political division of Mexico provided by Comisión Nacional de Áreas Naturales Protegidas (CONANP) under CCBY 4.0 International License. (PNG) [file pone.0292352.s006.png]
